# T Cell Exhaustion in Cancer Immunotherapy: Heterogeneity, Mechanisms, and Therapeutic Opportunities

**DOI:** 10.1002/advs.202520634

**Published:** 2026-01-12

**Authors:** Yang Yu, Xiaoyu Yao, Qingyang Wang, Mengrui Yang, Runze Li, Jiangjiang Qin, Jing Zhuang, Changgang Sun

**Affiliations:** ^1^ Faculty of Chinese Medicine and State Key Laboratory of Mechanism and Quality of Chinese Medicine Macau University of Science and Technology Macau China; ^2^ School of Public Health Shandong Second Medical University Weifang China; ^3^ College of First Clinical Medicine Shandong University of Traditional Chinese Medicine Jinan China; ^4^ Antitumor Omics Laboratory of Traditional Chinese Medicine Weifang Traditional Chinese Hospital Weifang China; ^5^ State Key Laboratory of Traditional Chinese Medicine Syndrome/Chinese Medicine Guangdong Laboratory, The Second Affiliated Hospital of Guangzhou University of Chinese Medicine (Guangdong Provincial Hospital of Chinese Medicine) Guangzhou University of Chinese Medicine Guangdong China; ^6^ Hangzhou Institute of Medicine (HIM) Chinese Academy of Sciences Zhejiang China; ^7^ Department of Oncology Weifang Traditional Chinese Hospital Weifang China; ^8^ College of Traditional Chinese Medicine Shandong Second Medical University Weifang China

**Keywords:** cancer immunotherapy, combination therapy, immune checkpoint inhibitors, T cell exhaustion, tumor microenvironment

## Abstract

T cell exhaustion represents a pivotal mechanism of immune escape in cancer, with its inherent heterogeneity and dynamic plasticity being key determinants of the variable responses and resistance to immune checkpoint inhibitors (ICIs). This review comprehensively delineates the multifaceted heterogeneity of exhausted T (T_EX_) cells, tracing their developmental trajectory from precursor exhausted T (T_PEX_) cells to terminally differentiated exhausted T (T_EX_ _‐term_) cells. We highlight both distinct and shared exhaustion features across diverse cancer types and spatial niches within the tumor microenvironment. Furthermore, we examine the multi‐layer biomarkers that drive and define this state, including characteristic surface inhibitory receptors, core transcription factors, and metabolism‐associated molecules. Grounded in this mechanistic understanding, we discuss emerging therapeutic strategies aimed at reversing T cell exhaustion. These range from the optimized application of ICIs and rational combination therapies involving epigenetic or metabolic interventions, to next‐generation engineered cell therapies such as chimeric antigen receptor T cell (CAR‐T), T cell receptor‐engineered T cell (TCR‐T), and tumor‐infiltrating lymphocytes (TILs), alongside emerging modalities including oncolytic viruses and bispecific antibodies. Finally, we discuss prevailing challenges and future directions, emphasizing that deciphering the heterogeneous landscape of T_EX_ cells, identifying precise biomarkers, and developing temporally controlled combination regimens are imperative to effectively reverse T cell exhaustion and broaden the therapeutic efficacy of cancer immunotherapy.

## Graphical abstract

T cell exhaustion limits immunotherapy efficacy. This article delineates its progression from stem‐like to terminally exhausted states, governed by persistent antigen, transcription factors, epigenetics, and metabolism. It maps the exhaustion landscape in the TME and proposes integrated reversal strategies, providing a translational roadmap to overcome immunotherapy resistance. Abbreviation: TME, tumour microenvironment.

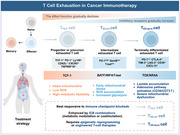

**Abbreviations**
ICI: immune checkpoint inhibitor
T_EX_ cell: exhausted T cell
T_PEX_ cell: precursor exhausted T cell
T_EX‐term_ cell: terminally differentiated exhausted T cell
ACT: adoptive cell therapy
PD‐1: programmed death protein 1
PD‐L1: programmed death‐ligand 1
CTLA‐4: cytotoxic T lymphocyte‐associated antigen 4
TME: tumour microenvironment
T_EX‐int_ cell: Intermediate exhausted T cell
LAG‐3: Lymphocyte‐activation gene 3
TIM‐3: T cell immunoglobulin and mucin‐domain containing‐3
TCF‐1: T cell factor 1
ICB: immune checkpoint blockade
TOX: thymocyte selection associated high mobility group box protein
NR4A: nuclear receptor subfamily 4, group A
OXPHOS: oxidative phosphorylation
TGF‐β: transforming growth factor‐β
PSR: proteotoxic stress response
HDACi: histone deacetylase inhibitor
DNMTi: DNA methyltransferase inhibitor
IDO: indoleamine 2,3‐dioxygenase
CAR‐T: chimeric antigen receptor T cell
TCR‐T: T cell receptor‐engineered T cell
TIL: tumor‐infiltrating lymphocyte
OV: oncolytic viruse
BsAb: bispecific antibody
ADC: antibody drug conjugate
LCMV: lymphocytic choriomeningitis virus
HIV: human immunodeficiency virus
HCV: hepatitis C virus
HBV: hepatitis B virus
TNF‐α: tumor necrosis factor‐alpha
IFN‐γ: interferon‐γ
TCR: T cell receptor
TIGIT: T cell immunoreceptor with Ig and ITIM domains
ITIM: immunoreceptor tyrosine‐based inhibitory motif
ITSM: immune receptor tyrosine‐based switching motif
SHP: src homology region 2 domain‐containing phosphatase
NFAT: nuclear factor of activated T cell
AP‐1: activator protein 1
MCT11: monocarboxylate transporter 11
Treg: regulatory T cell
AhR: aryl hydrocarbon receptor
MTA: 5’‐methylthioadenosine
SAM: S‐adenosylmethionine
ROS: reactive oxygen species
HIF‐1α: hypoxia‐inducible factor 1 alpha
TAM: tumor‐associated macrophage
MDSC: myeloid‐derived suppressor cell
ARG1: arginase‐1
SCFA: short chain fatty acid
GPCR: G protein‐coupled receptor
NHR: nuclear hormone receptor
EMD: extramedullary myeloma
CRC: colorectal cancer
SCLC: small cell lung cancer
TNBC: triple negative breast cancer
TWIST1: twist family BHLH transcription factor 1
TβRII: TGF‐β type II receptor
ICAM1: immune cell adhesion molecule 1
GBM: glioblastoma
TSH: thyroid‐stimulating hormone
TSHR: thyroid stimulating hormone receptor
MSI‐H: microsatellite instability‐high
B‐ALL: B‐cell acute lymphoblastic leukemia
MM: multiple myeloma
HCC: hepatocellular carcinoma
CESC: cervical cancer
HPV: human papillomavirus
TIME: tumor immune microenvironment
T_RM_ cell: T resident memory cell
T_fh_ cell: T follicular helper cell
MAIT cell: mucosal‐associated invariant T cell
CDK: cyclin‐dependent kinase
CK2: casein kinase II
NSCLC: non small cell lung cancer
FDA: Food and Drug Administration
GLIS1: GLIS family zinc finger 1
IgV: immunoglobulin variable
ITT: immunoreceptor tyrosine‐based tail
HNSCC: head and neck squamous cell carcinoma
MHC‐II: MHC class II molecule
APC: antigen‐presenting cell
FGL‐1: fibrinogen‐like protein 1
Gal‐9: galectin‐9
CEACAM‐1: carcinoembryonic antigen‐related cell adhesion molecule 1
PtdSer: phosphatidylserine
AML: acute myeloid leukemia
MDS: myelodysplastic syndromes
BATF: basic leucine zipper ATF‐like transcription factor
VEGF: vascular endothelial growth factor
A2aR: adenosine A2a receptor
IDO1: indoleamine 2,3‐dioxygenase 1
GCN2: general control nonderepressible 2
MSC: mesenchymal stem cell
OAd‐MSC: mesenchymal stem cell‐mediated delivery of oncolytic adenoviru
LPS: Lipopolysaccharide
PDAC: pancreatic ductal adenocarcinoma
PSGL‐1: P‐selectin glycoprotein ligand‐1
dMMR: mismatch repair‐deficient
Sema4A: semaphorin 4A
IGF‐IR: type I insulin‐like growth factor receptor
SCT: sodium citrate
LSD1: lysine specific demethylase 1
EV: extracellular vesicle
CPMV IT: cowpea mosaic virus‐based immunotherapy
YST‐OVH: tumor‐selective oncolytic herpesvirus vector
FasL: factor‐related apoptosis ligand
GzmB: granzyme B
DAMP: damage associated molecular pattern
MCT: metronomic chemotherapy
TET2: ten‐eleven translocation 2
CAR: chimeric antigen receptor
LBCL: large B‐cell lymphoma
DLBCL: diffuse large B‐cell lymphoma
CR: complete response
PR: partial response
PD: progressive disease
T_SCM_ cell: T stem cell memory
CRS: cytokine release syndrome
Bcl‐2: B cell lymphoma 2
C1QBP: complement component 1 Q subcomponent‐binding protein
GLUT1: glucose transporter1
LXRβ: liver X receptor β
SOCE: store‐operated calcium entry
ASS: argininosuccinate synthase
OTC: ornithine transcarbamylase
HDAC: histone deacetylase
Osr2: odd‐skipped related 2
ABL kinase: abelson tyrosine kinase
TBI: total body irradiation
SIN: synthetic immune niche
iPSC: induced pluripotent stem cell
BET: bromodomain and extra terminal domain
Multi‐Tvac: multi‐engineered T cell vaccine
LA: linoleic acid
HSV: herpes simplex virus
CRAd: conditionally replicating adenoviruse
RCC: renal cell carcinoma
G‐CSF: granulocyte colony‐stimulating factor
VV‐EpCAM BiTE: vaccinia virus encoding an EpCAM‐targeting bispecific T cell engager
VLV: virus‐like vesicle
B‐NHL: B‐cell non‐Hodgkin lymphoma
TsAb: Trispecific antibody
BiTE: bispecific T cell engager
EMT: epithelial‐mesenchymal transition
LNP: lipid nanoparticle

## 1 Introduction

The rapid advancement of cancer immunology has spurred the emergence of multiple breakthrough therapeutic strategies, with immune checkpoint inhibitors (ICIs) and adoptive cell therapy (ACT) being representative examples. These strategies have significantly enhanced the survival prognosis of patients with various advanced cancers. Therapies targeting immune checkpoints, such as programmed death protein 1 (PD‐1), programmed death‐ligand 1 (PD‐L1), and cytotoxic T lymphocyte‐associated antigen 4 (CTLA‐4), have exhibited durable clinical responses in a wide range of solid tumors and hematological malignancies. In some cases, patients have even achieved long‐term remission [1, 2]. Nonetheless, a substantial proportion of patients do not respond to treatment or ultimately develop resistance. This clinical challenge is, to a large extent, attributed to the dysfunctional state of T cells within the tumor microenvironment (TME), especially the critical state of T cell exhaustion [3, 4].

T cell exhaustion is a cellular state that occurs under the backdrop of persistent antigenic stimulation, such as during chronic infections or tumor progression. In this state, T cells gradually lose their effector functions, experience a marked decline in proliferation ability, and are accompanied by the sustained high expression of multiple inhibitory receptors, including PD‐1, lymphocyte‐activation gene 3 (LAG‐3), and T cell immunoglobulin and mucin‐domain containing‐3 (TIM‐3). This state not only impairs the tumor clearance ability mediated by T cells but also obstructs the formation of immune memory, thus significantly affecting the long‐term efficacy of immunotherapy [5]. Significantly, exhaustion does not signify cellular “ineptitude”. Instead, it represents a precisely regulated adaptive state. Underlying this state are unique transcriptomic, epigenetic, and metabolic reprogramming processes. These processes can maintain a certain level of pathogen or tumor control while simultaneously limiting immunopathology [6].

In recent years, the remarkable progress in single‐cell multi‐omics technologies and epigenetic analysis methods has significantly enhanced our comprehension of the biological nature of T cell exhaustion. Studies have clearly shown that exhausted T (T_EX_) cells exhibit a high degree of heterogeneity. They consist of distinct subpopulations, including precursor exhausted T (**T_PEX_**) cells, intermediate exhausted T (T_EX‐int_) cells, and terminally differentiated exhausted T (T_EX‐term_) cells, which form a well – defined and continuous differentiation pathway. Among these, T_PEX_ characterized by the expression of T cell factor 1 (TCF‐1), possess self‐renewal capacity and are sensitive to immune checkpoint blockade (ICB) therapy. In contrast, terminally exhausted cells display irreversible functional impairment and epigenetic “entrapment” [6, 7]. Crucial transcription factors such as thymocyte selection‐associated high mobility group box protein (TOX), TCF‐1, and nuclear receptor subfamily 4, group A (NR4A) have been identified as the central regulators of the exhaustion program. Through hierarchical epigenetic remodeling and metabolic – epigenetic cross – regulation, they jointly determine the fate of T cell exhaustion [8, 9, 10, 11]. Moreover, metabolic anomalies, such as impaired glycolysis/oxidative phosphorylation (OXPHOS) and mitochondrial dysfunction, along with immunosuppressive cytokines in the TME, such as Interleukin (IL)‐10 and transforming growth factor‐β (TGF‐β), have also been recognized as key determinants in maintaining and stabilizing the exhausted state [12, 13, 14, 15, 16]. Recent research has also disclosed that persistent antigenic signals, by driving chronic AKT activation, initiate a distinctive proteotoxic stress response (PSR). This response is characterized by an increase in global protein synthesis, a specific upregulation of the endoplasmic reticulum stress chaperone proteins BiP/gp96, and the accumulation of protein aggregates. These events collectively disrupt proteostasis, directly leading to the functional exhaustion of T cells [17]. Together, these mechanisms form a multi‐tiered regulatory network that drives the progressive loss of T cell function, culminating in an irreversible exhausted state under continuous antigen exposure.

Building upon these insights into the underlying mechanisms, novel therapeutic strategies are advancing at a rapid pace, transcending the traditional single ICB paradigm and moving towards a paradigm of combinatorial interventions and precise regulation. Specifically, the concurrent application of histone deacetylase inhibitors (HDACi) or DNA methyltransferase inhibitors (DNMTi) in conjunction with ICB holds the potential to reverse the epigenetic silencing of T cells, thereby rekindling their effector functions. This approach aims to modulate the epigenetic landscape of T cells, unlocking gene expression programs that were previously repressed and restoring their ability to mount an effective anti – tumor immune response [11, 18, 19].

Furthermore, targeting immune metabolic pathways or obstructing inhibitory signals, including adenosine, indoleamine 2,3‐dioxygenase (IDO), and TGF‐β, has demonstrated promising synergistic antitumor capabilities in both pre‐clinical investigations and clinical trials [5, 16, 20, 21, 22]. In the realm of cell therapy, gene editing technologies such as CRISPR‐Cas9 can effectively knock out molecules associated with T cell exhaustion or enhance the expression of functional promoters. These interventions have the potential to significantly improve the persistence, stem cell‐like characteristics, and anti‐tumor efficacy of chimeric antigen receptor T cell (CAR‐T), T cell receptor‐engineered T (TCR‐T), and tumor‐infiltrating lymphocytes (TILs) [4, 23, 24]. Furthermore, oncolytic viruses (OVs), bispecific antibodies (BsAbs), antibody drug conjugates (ADCs), and costimulatory agonists offer innovative strategies for reversing immune exhaustion and overcoming immunosuppression through various mechanisms [23, 25, 26].

This article systematically reviews the latest progress in the field of T cell exhaustion. Starting from its definition and core characteristics, the key molecular mechanisms regulating the exhaustion state, the heterogeneity of cell subsets, and new biomarkers are discussed. On this basis, we will focus on innovative therapeutic strategies to target T cell exhaustion, including the optimized application of existing ICIs, the development of combination therapy strategies, and the improvement of the next generation of cell therapy technology, in order to provide in‐depth theoretical insights and feasible translational directions for overcoming current immunotherapy resistance.

## 2 The Biological Basis of T Cell Exhaustion

### 2.1 Discovery and Definition of T Cell Exhaustion

T cell exhaustion refers to a critical cellular state characterized by the progressive loss of effector function in T cells due to persistent antigen exposure during chronic infections and within the tumor microenvironment. The conceptual foundation of this phenomenon originated from studies of a mouse model of chronic lymphocytic choriomeningitis virus (LCMV) infection conducted in the 1990s [27]. It was observed that, in contrast to the functional effector and memory T cells generated during acute infection, virus‐specific CD8^+^ T cells in chronically infected mice exhibited a gradual decline in functionality. These cells demonstrated significantly impaired proliferative capacity, reduced secretion of inflammatory cytokines, and diminished cytotoxic activity, ultimately resulting in a failure to clear the virus and increased susceptibility to apoptosis. This seminal study not only provided the first direct experimental evidence of T cell exhaustion but also established a classical animal model that remains widely used in immunological research today [28].

Since then, T cell dysfunction phenotypes, closely resembling those observed in the LCMV model, have been identified in patients with chronic viral infections, including human immunodeficiency virus (HIV), hepatitis C virus (HCV), and hepatitis B virus (HBV), as well as in TILs, thereby confirming the broad applicability and cross‐disease relevance of the T_EX_ cell state [29, 30, 31, 32, 33, 34]. Notably, these T_EX_ cells consistently exhibit elevated expression of multiple inhibitory receptors, with PD‐1 recognized as one of their most characteristic molecular markers [30, 35, 36]. These findings represent a pivotal transition in T cell exhaustion research—from experimental models to human disease contexts—and have established critical molecular targets for the development of ICB therapeutic interventions.

An important turning point in the field emerged from the systematic analysis of LCMV models conducted by the Wherry team. For the first time, they established a clear conceptual framework for T cell exhaustion and elucidated its core biological characteristics [37]. T_EX_ cells are defined as a distinct differentiation state characterized by progressive loss of effector function, co‐expression of multiple inhibitory receptors, a unique transcriptomic and epigenetic signature, and an inability to develop into functional memory cells under persistent antigenic and inflammatory stimulation [36, 38, 39, 40]. The dysfunction of these cells follows a hierarchical pattern: diminished proliferative capacity and loss of IL‐2 production occur early, followed by reduced secretion of tumor necrosis factor‐alpha (TNF‐α) and interferon‐γ (IFN‐γ) [41, 42]. Mechanistically, T_EX_ cells not only exhibit high expression of inhibitory receptors such as PD‐1, TIM‐3, LAG‐3, and CTLA‐4 [36, 43, 44], but also display a gene expression profile distinct from that of effector and memory T cells, as well as substantial alterations in chromatin accessibility. These epigenetic modifications are considered a fundamental mechanism underlying the irreversibility of the exhausted state [5]. This study not only formally established T cell exhaustion as a unique T cell differentiation lineage, but also provided a critical theoretical foundation for the development of ICB therapies.

**FIGURE 1:**
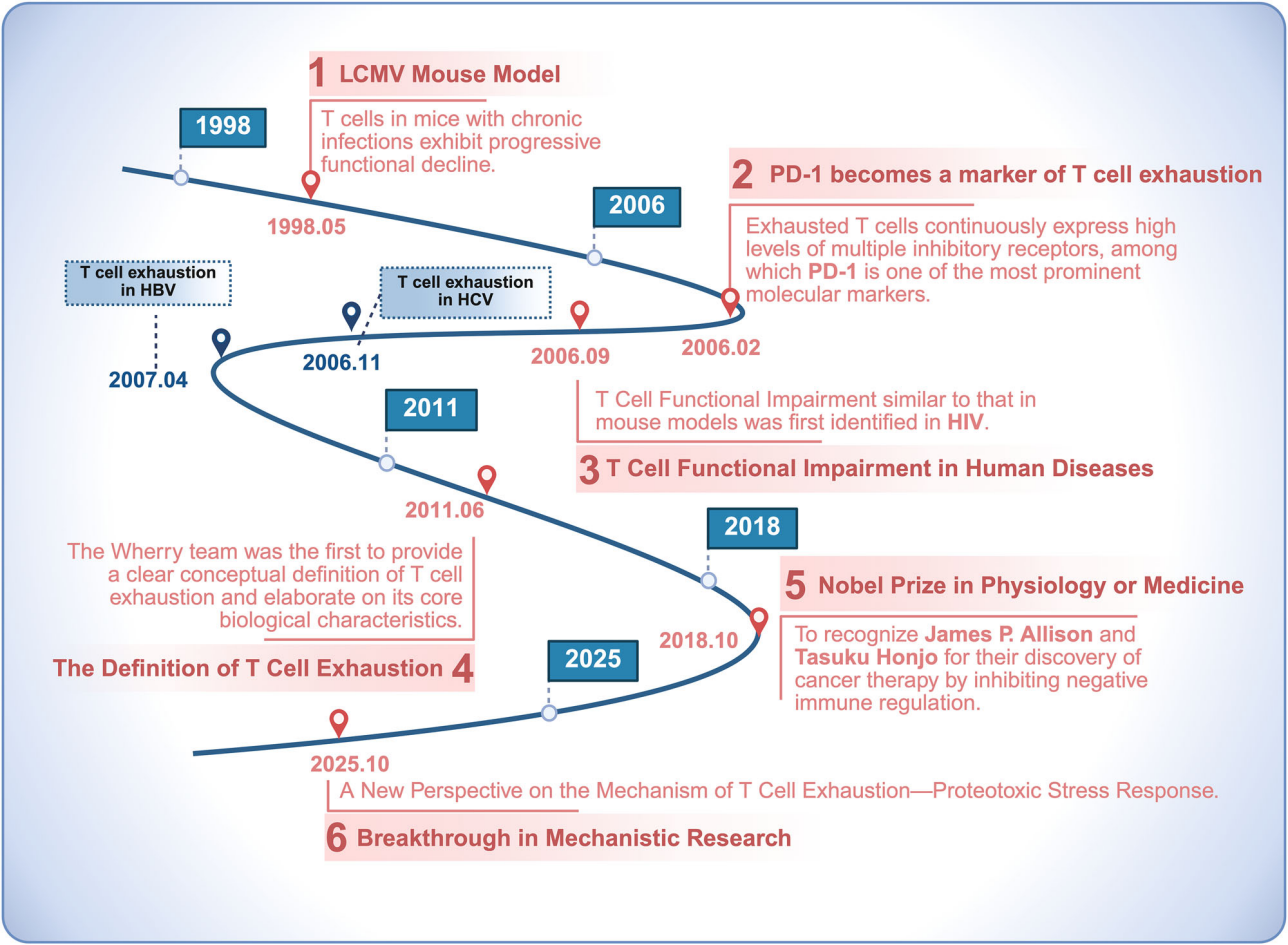
A timeline of research history and milestones of T cell exhaustion. Abbreviations: HBV, hepatitis B virus; HCV, hepatitis C virus; HIV, human immunodeficiency virus; LCMV, lymphocytic choriomeningitis virus; PD‐1, programmed death protein 1.

The clinical success of ICIs, such as those targeting PD‐1 and CTLA‐4, has established the reversal of T cell exhaustion as a viable therapeutic strategy, catalyzing a transformative shift in cancer treatment. For their pioneering contributions to the discovery of immune checkpoint regulation, James P. Allison and Tasuku Honjo were awarded the 2018 Nobel Prize in Physiology or Medicine [45, 46]. In recent years, extensive research has further demonstrated that the gene expression profile of T_EX_ cells is stabilized by durable epigenetic modifications, enabling their dysfunctional state to persist for prolonged periods even in the absence of antigenic stimulation. This mechanism elucidates the limitations of current ICB monotherapies in fully restoring the functionality of T_EX‐term_ cells [47], while also identifying molecular‐level regulators—acting as both “on‐switches” and “state stabilizers”—of the exhaustion program. These insights provide a critical foundation for the development of next‐generation immunotherapeutic approaches that extend beyond conventional ICB strategies (Figure 1).

### 2.2 Multifactorial Mechanisms Underlying T Cell Exhaustion

**FIGURE 2:**
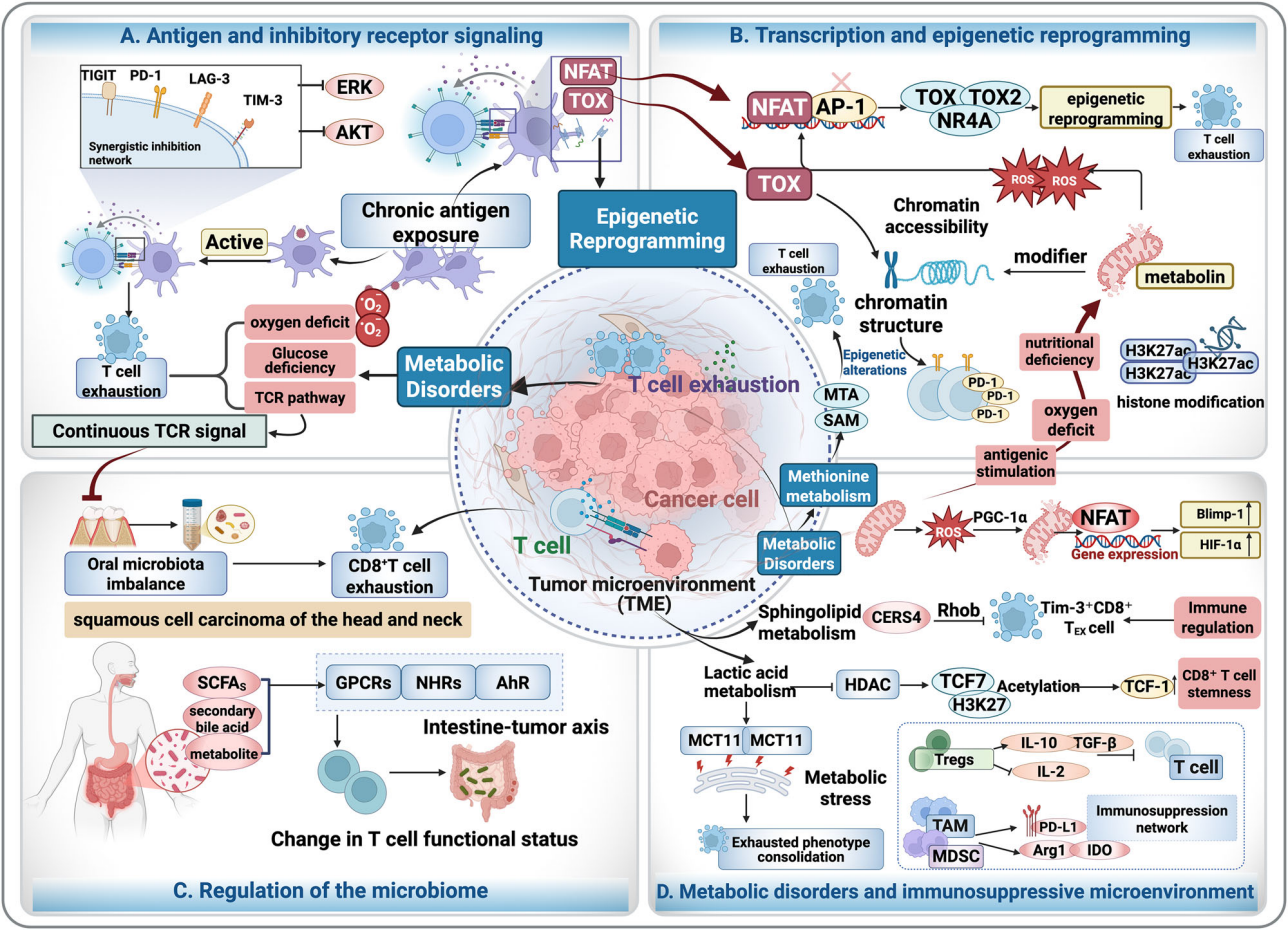
Regulatory mechanisms of T cell exhaustion. This figure systematically illustrates the multi‐level coordinated regulatory network underlying T cell exhaustion. (A) Persistent antigen exposure causes long‐term, low‐intensity activation of TCR signaling, leading to the gradual loss of T cell effector functions and activation of key transcription factors such as NFAT and TOX, thereby initiating epigenetic reprogramming. (B) Multiple epigenetic reprogramming mechanisms, including transcriptional regulators, dynamic changes in chromatin accessibility, histone modifications, and DNA methylation, collectively drive the transition of T cells to an exhausted phenotype. (C) Emerging regulatory factors highlight that dysbiosis of the intestinal microbiota and microbiota‐derived metabolites profoundly influence T cell exhaustion. (D) Metabolic disorders and the immunosuppressive microenvironment promote T cell exhaustion through multiple pathways. For example, mitochondrial damage induces excessive production of ROS, which in turn promotes NFAT activation. Immunosuppressive cells and their associated mediators cooperate to establish an inhibitory network. In addition, methionine metabolism generates high levels of SAM and MTA, leading to chromatin condensation in T cells and epigenetic remodeling, thereby consolidating exhaustion‐related gene expression profiles. Abbreviations: TCR, T cell receptor; NFAT, nuclear factor of activated T cell; TOX, thymocyte selection–associated high mobility group box protein; ROS, reactive oxygen species; SAM, S‐adenosylmethionine; MTA, 5‐methylthioadenosine.

The development of T cell exhaustion is a complex and dynamic process mediated by multiple signals within the TME. This phenomenon arises from multilayered and interconnected biological mechanisms, including persistent antigen stimulation, inhibitory receptor signaling, transcriptional and epigenetic reprogramming, metabolic dysregulation, and an immunosuppressive milieu. Collectively, these factors establish a positive feedback loop that progressively drives T cells from functional impairment to terminal exhaustion (Figure 2).

#### 2.2.1 Persistent Antigen and Inhibitory Receptor Signaling: Initiation and Reinforcement of Exhaustion

Continuous antigenic stimulation serves as the initiating trigger for the T cell exhaustion program. In contrast to the transient stimulation observed during acute infections, persistent antigen exposure in the context of tumors leads to prolonged, low‐intensity activation of T cell receptor (TCR) signaling [48]. This abnormal TCR signaling not only directly leads to the loss of T cell effector function but also induces the expression of inhibitory receptors by activating key transcription factors such as nuclear factor of activated T cells (NFAT) and TOX, and triggers global transcriptional and epigenetic reprogramming. See Section 2.2.2 for details. These changes persist even after antigen removal, establishing a durable “exhaustion signature” that is resistant to reversal and impedes the complete restoration of T‐cell function [49]. In addition, persistent TCR signaling acts synergistically with metabolic stresses in the tumor microenvironment—such as hypoxia and glucose deprivation—to rapidly induce mitochondrial dysfunction, impaired glycolysis, and reactive oxygen species (ROS) accumulation, thereby further promoting stabilization of the T_EX_ cell phenotype [14]. Metabolic disturbances not only restrict the energy supply to T cells but also impair the activities of histone‐modifying enzymes and DNA methyltransferases, contributing to the epigenetic stabilization of exhaustion‐associated genes [12]. For further details, refer to Section 2.2.3.

Inhibitory receptor networks play a central role in establishing and stabilizing T cell exhaustion. In normal immune responses, molecules like PD‐1, CTLA‐4, TIM‐3, LAG‐3, and T cell immunoreceptor with Ig and ITIM domains (TIGIT) are transiently expressed to maintain immune homeostasis [50, 51, 52, 53, 54, 55, 56]. However, under persistent antigen exposure in the TME, these receptors become stably and highly expressed. They form a synergistic inhibitory network that suppresses T cell function through multiple mechanisms. At the juxtamembrane level, they directly interfere with TCR signaling, disrupt the formation of immunological synapses, and attenuate the activation of key downstream kinases such as AKT and ERK [57, 58]. Moreover, the intracellular domains of these receptors contain immune receptor tyrosine‐based inhibitory motifs (ITIMs) or immune receptor tyrosine‐based switching motifs (ITSMs), which recruit phosphatases such as src homology region 2 domain‐containing phosphatase (SHP)‐1 and SHP‐2. These phosphatases subsequently dephosphorylate critical signaling molecules in the TCR and CD28 pathways, thereby dampening activation signals at their origin [59, 60, 61]. Additionally, this inhibitory network can exert direct effects in the nucleus by modulating gene expression programs, thereby promoting the establishment and stabilization of exhaustion‐associated transcriptional profiles [62, 63]. Sustained overactivation of this network collectively results in the loss of T cell cytotoxicity, impaired cytokine production, and complete abrogation of proliferative capacity [64, 65].

#### 2.2.2 Transcription and Epigenetic Reprogramming: Solidification of the Exhausted State

T cell exhaustion represents a progressive state driven by an aberrant transcriptional program and maintained through sustained epigenetic remodeling [66]. In the absence of costimulatory signals such as CD28, persistent antigenic stimulation leads to the activation of the transcription factor NFAT in a “chaperonless” conformation [67]. Without its canonical partner activator protein 1 (AP‐1), NFAT preferentially initiates a transcriptional program associated with T cell exhaustion and upregulates key transcription factors, including TOX/TOX2 and NR4A [68, 69]. These factors not only execute the exhaustion program but also function as “molecular architects” by orchestrating epigenetic changes. For instance, TOX modulates chromatin architecture by directing the recruitment of chromatin‐modifying complexes and sustaining the accessibility of gene loci encoding inhibitory receptors such as PD‐1, thereby reinforcing their sustained expression [70, 71].

At the epigenetic level, T_EX_ cells exhibit a chromatin landscape that is distinctly different from that of effector and memory T cells. Central to this distinction is the establishment of an “epigenetic scar” which serves as the core molecular mechanism underlying the irreversible nature of T cell exhaustion [72]. This remodeling process originates with widespread alterations in chromatin accessibility. Assay for transposase‐accessible chromatin using sequencing (ATAC‐seq) has revealed that open chromatin regions are highly enriched at gene loci associated with exhaustion markers such as PD‐1 and TIM‐3, whereas genes related to effector and memory functions display reduced chromatin accessibility [73, 74]. This differential accessibility pattern lays the foundation for subsequent stabilization through deeper epigenetic modifications. Concurrently, DNMT3A‐mediated de novo DNA methylation emerges as an early initiating event in the exhaustion program. It targets promoters of key effector and memory‐associated genes, including Tcf7 and Ifng, leading to their transcriptional silencing and stable inheritance across cell divisions [19, 75, 76]. The progressive accumulation and consolidation of these methylation events ultimately give rise to a durable and largely irreversible epigenetic methylation signature. This provides a mechanistic explanation for the limited efficacy of ICIs in fully restoring the functional capacity of terminally T_EX_ cells [11].

Subsequently, DNA methylation and histone modifications establish a tightly coordinated, self‐reinforcing synergistic network. Notably, repressive histone marks such as H3K27me3 can actively recruit the DNA methyltransferase DNMT3A to specific genomic loci, thereby facilitating the establishment of stable DNA methylation at these sites and enabling deep silencing of effector and memory‐associated genes [77, 78]. Conversely, DNA methylation can recruit methyl‐CpG‐binding domain proteins (MBDs), which in turn facilitate the recruitment of histone deacetylases (HDACs) and other repressive complexes, further reinforcing local transcriptional repression and forming a self‐sustaining feedback loop [79]. Importantly, this highly cooperative silencing network also harbors intrinsic counteracting mechanisms, such as the histone demethylase KDM6B, which may partially preserve the plasticity of T_PEX_ cells [80, 81]. However, in T_EX‐term_ cells, the aforementioned cooperative silencing mechanism becomes overwhelmingly dominant. This imbalance, combined with a state of “transcriptional arrest” imposed by bivalent chromatin domains marked by both H3K4me3 and H3K27me3, contributes to locking cells in a terminal state of severe functional impairment [80]. Critically, chromatin remodeling complexes such as mSWI/SNF act as ultimate executors of epigenetic information by regulating the three‐dimensional architecture of the genome and stabilizing covalent modifications within specific spatial conformations. This directly leads to the widespread phenomenon of “active mark–gene expression uncoupling” in T_EX‐term_ cells. Specifically, epigenomic analyses including ChIP‐seq and CUT&RUN have revealed that although many genomic regions retain active enhancer marks such as H3K27ac, the physical constraints imposed by closed chromatin conformation and the absence of essential transcription factors prevent effective activation of the associated gene expression programs [82, 83].

More importantly, a profound crosstalk exists between metabolic perturbations and epigenetic reprogramming within the tumor microenvironment, further exacerbating the stabilization of the T_EX_ cell phenotype. Metabolites, serving as essential substrates or regulators of epigenetic modifications, directly influence chromatin states [84]. For instance, recent studies have demonstrated that histone lactoylation, including H3K18la and H3K9la, is highly enriched in effector T cells and functions as a critical link between metabolism and epigenetics, coinciding with active expression of cytotoxic and effector function‐related genes [85, 86, 87]. In contrast, in T_EX‐term_ cells, levels of lactoylation are markedly reduced due to metabolic dysregulation, while histone acetylation becomes the predominant regulatory modification—indicating that the metabolic state of T cells directly shapes their epigenetic landscape [88, 89]. Furthermore, it has been established that targeted metabolic or epigenetic interventions can partially remodel the epigenetic architecture of T_EX_ and restore functional capacity. For example, L‐2‐hydroxyglutaric acid (L‐2‐HG), an immunomodulatory metabolite, enhances T_EX_ cells functionality and antitumor immunity by modulating epigenetic marks such as H3K27me3 [90]. These findings indicate that the irreversibility of T cell exhaustion arises not only from persistent epigenetic locking but also from dysregulation of the metabolism‐epigenetics axis.

Therefore, the irreversibility of T cell exhaustion stems from persistent antigen stimulation, which initiates a self‐reinforcing, multilevel epigenetic network encompassing DNA methylation, histone modifications, and chromatin remodeling. This network stabilizes aberrant transcriptional programs into durable “epigenetic scars” with metabolic dysregulation serving as a key enabler in establishing this profound “molecular lock”.

#### 2.2.3 Metabolic Disorders and Immunosuppressive Microenvironment: External Constraints on Functional Execution

Metabolic dysregulation represents a central extrinsic factor contributing to T cell exhaustion within the TME, with profound interconnections at the molecular level. Persistent antigen exposure, coupled with chronic hypoxia and nutrient deprivation in the TME, induces mitochondrial dysfunction in T cells, characterized by impaired mitochondrial biogenesis, loss of membrane potential, and reduced ATP production [14, 91]. Notably, this mitochondrial impairment is not merely a consequence of T cell exhaustion but also a key driver of its progression. Accumulation of ROS suppresses PGC‐1α‐mediated mitochondrial biogenesis, leading to diminished mitochondrial quantity and function [92, 93]. This mitochondrial insufficiency not only compromises cellular energy metabolism but also promotes aberrant activation of transcription factors such as NFAT via ROS signaling, thereby upregulating expression of exhaustion‐associated genes, including Blimp‐1 and hypoxia‐inducible factor 1 alpha (HIF‐1α), and facilitating the differentiation of T cells toward a terminally exhausted phenotype [94]. Furthermore, defective mitophagy results in the accumulation of dysfunctional mitochondria, exacerbating both metabolic disturbances and functional decline in T cells [95, 96].

In recent years, sphingolipid metabolism—recognized as a critical component of metabolic reprogramming within the TME—has been established as a key regulator of T cell function and response to immunotherapy. Emerging evidence indicates that CERS4 mediates the suppression of CD8^+^ Tim‐3^+^ T_EX_ cells via RhoB signaling, thereby promoting immune modulation and serving as a predictive biomarker for favorable responses to anti‐PD‐1 therapy [97]. Beyond sphingolipid metabolism, other metabolic pathways also significantly contribute to T cell exhaustion. Tumor cells reprogram methionine metabolism to generate elevated levels of S‐adenosylmethionine (SAM) and 5’‐methylthioadenosine (MTA), which induce chromatin condensation and epigenetic remodeling in T cells, thereby altering the expression profiles of exhaustion‐related genes [20, 98]. Notably, supplementation with L‐2‐hydroxyglutarate (L‐2‐HG) has been shown to partially reverse the exhausted phenotype [90]. Moreover, methionine deficiency reduces H3K79me2 levels, suppresses AMPK expression, upregulates PD‐1, and promotes exhaustion in CD4^+^ T cells [99]. Concurrently, tumor cells and immunosuppressive populations deplete essential amino acids such as tryptophan and methionine, impairing T cell proliferation and effector functions. Kynurenine (Kyn), a tryptophan metabolite, activates the aryl hydrocarbon receptor (AhR) and directly drives T cell exhaustion [99, 100]. Additionally, T_EX_ cells exhibit diminished glycolytic capacity and restricted glucose uptake and utilization. Despite sustained activation of mTOR signaling, downstream metabolic pathways are inhibited by signals from immune checkpoint receptors such as PD‐1, leading to impaired OXPHOS, insufficient energy production, and compromised cytotoxic and proliferative capacities [14, 101, 102]. Notably, monocarboxylate transporter 11 (MCT11), a lactate transporter, is highly expressed in T_EX‐term_ cells, facilitating increased lactate uptake and thereby exacerbating metabolic stress and dysfunction [103, 104]. Under conditions of hyperlactatemia, both glycolysis and mitochondrial metabolism in CD8^+^ T cells are impaired, further promoting the stabilization of the exhausted phenotype [105]. However, recent studies have revealed that lactate exerts a dual role in the regulation of T cell exhaustion. In addition to its traditionally recognized immunosuppressive effects, lactate contributes to the preservation of CD8^+^ T cell stemness through metabolic‐epigenetic crosstalk. Specifically, on one hand, lactate‐driven histone lactylation—such as H3K18la and H3K9la—can regulate the transcription of key genes involved in CD8^+^ T cell differentiation and function [86]. On the other hand, lactate inhibits HDAC activity, increases H3K27 acetylation at the Tcf7 super‐enhancer region, and enhances TCF‐1 expression, thereby supporting the stem‐like properties and self‐renewal capacity of CD8^+^ T cells [106]. Thus, metabolic dysregulation cooperates through multiple interconnected mechanisms—including mitochondrial dysfunction, epigenetic remodeling, impaired glycolysis, lactate accumulation, and amino acid depletion—to drive the initiation, maintenance, and progression of T cell exhaustion.

The immunosuppressive cell populations within the TME play a pivotal role in the induction and maintenance of T cell exhaustion [107]. Regulatory T cell (Treg) directly suppresses effector T cell function through the secretion of immunosuppressive cytokines such as IL‐10 and TGF‐β, as well as through competitive consumption of IL‐2 [108, 109]. Tumor‐associated macrophages (TAMs) and myeloid‐derived suppressor cells (MDSCs) collaboratively establish a robust immunosuppressive network by upregulating inhibitory ligands, including PD‐L1, and producing immunosuppressive mediators such as arginase‐1 (ARG1) and IDO [110]. Furthermore, dendritic cells (DCs) in the TME frequently exhibit a tolerogenic phenotype characterized by reduced expression of costimulatory molecules and elevated expression of PD‐L1 and IDO, thereby failing to effectively prime full T cell activation and instead promoting T cell exhaustion [111, 112, 113].

#### 2.2.4 Emerging Drivers: The Regulatory Role of the Microbiome

The gut microbiome and its metabolites are emerging as systemic regulators of T cell exhaustion [114, 115]. Preclinical evidence demonstrates that chronic restraint stress can disrupt oral microbial homeostasis, thereby promoting premature CD8^+^ T cell exhaustion and accelerating the progression of head and neck squamous cell carcinoma [116]. Moreover, gut‐derived metabolites, including short‐chain fatty acids (SCFAs), secondary bile acids, and tryptophan derivatives (e.g., indole‐3‐lactic acid), can modulate immune responses. These metabolites signal through various receptors, such as G protein‐coupled receptors (GPCRs), nuclear hormone receptors (NHRs), and AhR, to directly influence T cell differentiation, metabolism, and function [114, 117, 118]. Collectively, these findings highlight mechanisms underlying the “gut–tumor axis” and the broader microbiota–immune axis in the systemic regulation of anti‐tumor immunity and T cell fate, offering novel insights into the systemic determinants of T cell exhaustion.

The process of T cell exhaustion is highly complex, involving multiple regulatory layers, including persistent antigen stimulation, transcriptional control, epigenetic reprogramming, and metabolic dysregulation. Current studies indicate that under sustained antigen exposure, TCR signaling initiates the activation of key transcription factors such as NFAT and TOX in T cells. These factors not only directly regulate the expression of inhibitory receptors but also drive extensive epigenetic modifications—including DNA methylation, histone modifications, and chromatin remodeling—leading to the establishment of stable “epigenetic scars” that render the exhausted phenotype largely irreversible [11, 119]. Concurrently, this epigenetic locking interacts with metabolic impairments such as defective glycolysis, mitochondrial dysfunction, and the immunosuppressive tumor microenvironment, collectively amplifying T cell exhaustion and ultimately resulting in a permanent loss of effector function [12, 14]. These mechanisms are not isolated; rather, they engage in extensive crosstalk and jointly govern the initiation, maintenance, and therapeutic responsiveness of T cell exhaustion. Therefore, therapeutic strategies targeting T cell exhaustion should extend beyond isolated interventions—such as PD‐1 blockade—and instead focus on modulating early critical nodes within the regulatory network (e.g., preventing the formation of epigenetic scars) or leveraging synergistic approaches, such as combining epigenetic modulators with ICB, to achieve more durable and effective immune restoration.

## 3 Heterogeneity in T Cell Exhaustion

**FIGURE 3:**
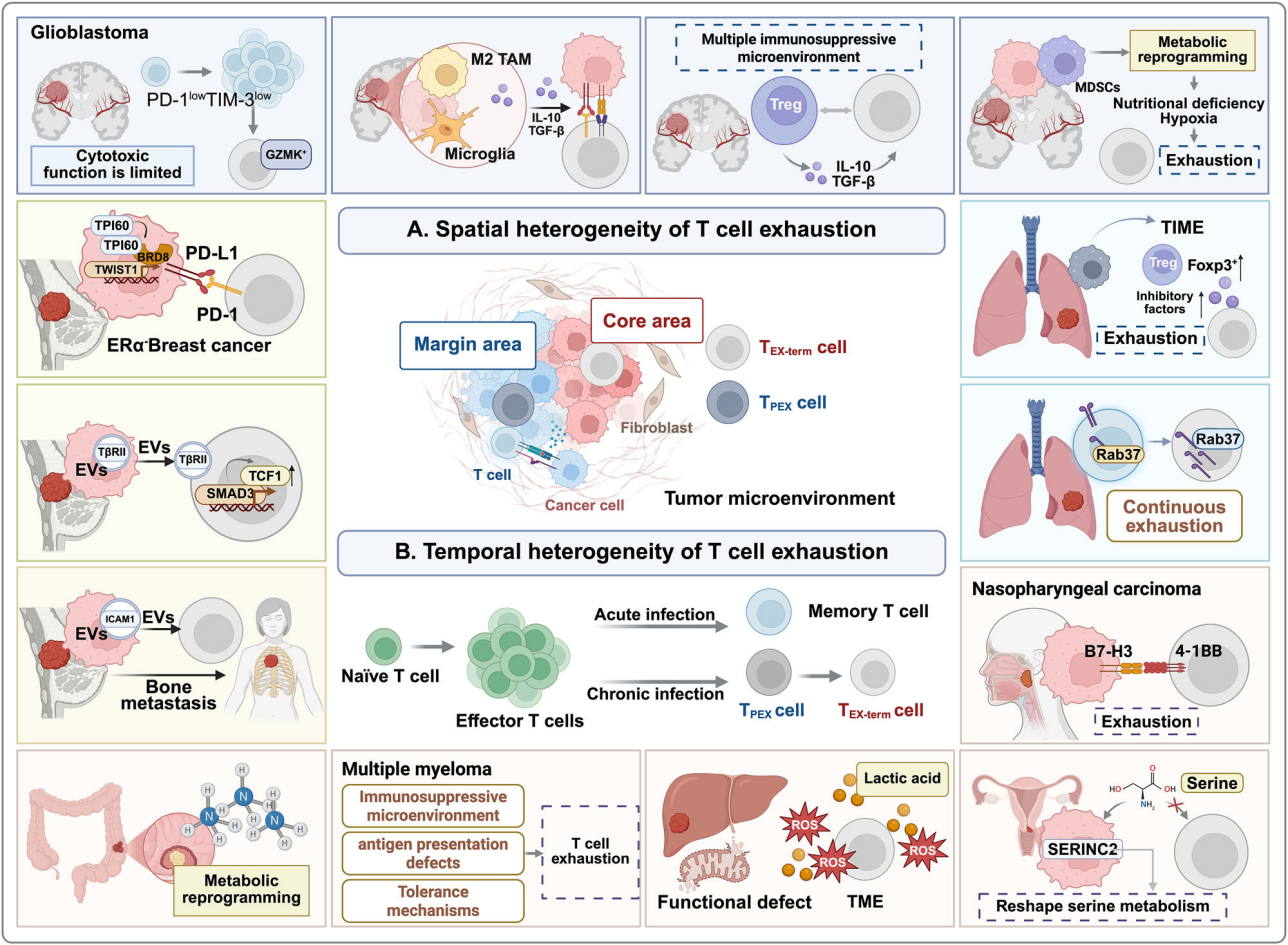
Heterogeneity of T cell exhaustion. T cell exhaustion is a highly heterogeneous and dynamic process characterized by complexity across multiple dimensions. Temporally, it represents a progressive differentiation trajectory driven by key transcription factors and accompanied by epigenetic remodeling, leading irreversibly from T_PEX_ to T_EX‐term_ cells with diminished effector function. Spatially, distinct cancer types foster disease‐specific regulatory networks of T cell exhaustion due to differences in tumor microenvironment, antigen sources, and metabolic profiles. Notably, the core and peripheral regions of tumors are enriched with functionally distinct subsets—terminal and T_PEX_, respectively—forming a defined immuno‐spatial architecture. Furthermore, heterogeneity extends across T cell lineages: CD8^+^ T cells, CD4^+^ T cells, and certain unconventional T cell populations exhibit lineage‐specific exhaustion pathways and functional phenotypes. A comprehensive understanding of this multidimensional heterogeneity is critical for the development of precision immunotherapies targeting specific stages of exhaustion, anatomical locations, cancer types, or T cell subsets. Abbreviations: ERα, estrogen receptor Alpha; EV, extracellular vesicle; MDSC, myeloid‐derived suppressor cell; ROS, reactive oxygen species; TAM, tumor‐associated macrophage; T_EX‐term_ cell, terminally differentiated exhausted T cell; TIME, tumor immune microenvironment; TME, tumor microenvironment; T_PEX_ cell, precursor exhausted T cell; Treg, regulatory T cell.

T cell exhaustion is not a uniform condition but exhibits substantial spatiotemporal heterogeneity, regulated by multiple factors within the TME, including the intensity and duration of antigen exposure as well as epigenetic programming, all of which significantly influence tumor progression and response to immunotherapy (Figure 3, Table 1).

**TABLE 1:** Heterogeneity of T cell exhaustion: shared and context‐specific features.

Shared features	References
Core mechanisms: core drivers (chronic antigenic stimulation), key transcriptional regulation (e.g., TOX, TCF‐1, NR4A), epigenetic programming (stable epigenetic locking), and expression of signature inhibitory receptors (e.g., PD‐1, TIM‐3).	[11, 48]
Functional outcome: These shared mechanisms collectively result in the impairment of T cell effector function, reduced proliferative capacity, and defective cytokine secretion, which constitute a critical pathway underlying tumor immune escape and resistance to immunotherapy.	[3, 120]

Context‐ Specific Features						
Context/Category	Tumor Type/Subset	Niche		Dominant T_EX_ Subset(s)	Key Markers/Pathways/Mechanism	References
Temporal and dynamic heterogeneity	Pan‐cancer species	Tumor microenvironment		T_PEX_ cell	TCF‐1^+^; Self‐renewal, functional plasticity	[121]
				T_EX‐int_ cell	T‐bet^+^, CX3CR1^+^; Partial effect function	[122]
				T_EX‐term_ cell	TOX^+^, EOMES^+^; Epigenetic locking and severely impaired function	[123, 124]
Heterogeneity of spatial distribution	Pan‐cancer species	Draining lymph nodes/invasive front		T_PEX_ cell	TCF‐1^+^	[125]
		Tertiary lymphatic structure	mTLS and its surrounding areas	T_PEX_ cell, CD39^+^CD8^+^ T_PEX_ cell, CD4^+^ T_PEX_ cell	TCF‐1^+^ (TCF7^+^); Function is maintained and anti‐tumor immunity is enhanced	[126]
			imTLS or tumor margins	T_PEX_ cell	TCF‐1^+^; EMT‐related genes were expressed and immunosuppressed	[127]
		The core area of the tumor		T_EX‐term_ cell	TIM‐3^+^, LAG‐3^+^, PD‐1^+^, CD101^+^, EOMES+	[128]
Heterogeneity among cancer species	Lung cancer	Tumor microenvironment		T_PEX_ cell	PD‐1^+^ CD8^+^T cell, TIM‐3, TIGIT, CTLA‐4, CK2B	[129, 130]
	Triple‐negative Breast Cancer	Tumor microenvironment		CD8^+^ T_PEX_ cell	PD‐1^+^ CXCL13^+^ T_EX_ (Down‐regulation of IFN‐γ and TNF expression, up‐regulation of granzyme B)	[131, 132]
	Glioblastoma multiforme	Tumor microenvironment		T_PEX_ cell (Atypical)	PD‐1 low, TIM‐3 low, GZMK^+^, Kdm6a (Sex specific)	[133]
	Colorectal cancer	Tumor microenvironment		CD8^+^ T_PEX_ cell	CD160^+^ T cell (anti‐terminal exhaustion, strong clonal expansion), TSH/TSHR signaling pathway	[134, 135]
	Hepatocellular carcinoma	Liver microenvironment		CD8^+^ T_PEX_ cell	TIGIT^+^, PD‐1^+^; Unique immune tolerance microenvironment	[136]
	B‐cell acute lymphoblastic leukemia	Bone marrow		CD4^+^ T_PEX_ cell	TIM‐3^+^, PD‐1^+^; Thctx/Thctx‐ex(cytotoxic and helper functions)	[137, 138]
	Cervical cancer	Tumor microenvironment		T_PEX_ cell	TIM‐3^+^, LAG‐3^+^, PD^+^;TIGIT/CD155; serine metabolism	[139, 140]
Heterogeneity between subtypes	/	Tertiary lymphatic structures, lymph nodes		CD8^+^ T_PEX_ cell	TCF‐1^+^, Ly108^+^, CD69^+^; Self renewal capacity	[122, 141]
	/	The core area of the tumor		CD8^+^ T_EX‐term_ cell	TOX^+^, EOMES^+^, TIM‐3^+^, LAG‐3^+^, CD101^+^	[142, 143]
	/	Tumor microenvironment		CD4^+^ T_PEX_ cell	TCF‐1^+^ T_PEX_, BLIMP‐1^+^ LAG‐3^+^ T_EX‐term_; Indirect effects on CD8^+^ T cell activity	[144, 145, 146]
	/	Tumor microenvironment		Tregs exhibiting exhaustion‐associated phenotypes	PD‐1^+^, TIM‐3^+^, TIGIT^+^	[147]
	/	Tumor microenvironment		Exhausted γδ T cell	PD‐1^+^, NR4A2/3^+^	[148, 149]
	/	Tumor microenvironment		Exhausted MAIT cell	PD‐1^+^, TIM‐3^+^, CD39^+^, Lipid peroxidation	[150, 151]

Abbreviations: MAIT, mucosal‐associated invariant T cell; T_PEX_ cell, progenitor exhausted T cell; T_EX_ cell, exhausted T cell; T_EX‐int_ cell, intermediate exhausted T cell; T_EX‐term_ cell, terminally exhausted T cell; Treg, regulatory T cell; mTLS, mature tertiary lymphoid structures; imTLS, immature tertiary lymphoid structures.

### 3.1 Temporal Dynamics of Heterogeneity

From the perspective of temporal dynamics, T cell exhaustion follows a progressive differentiation trajectory initiated by chronic antigen stimulation [152]. During acute immune responses, Naïve T cells are activated through TCR engagement and costimulatory signals like CD28. They differentiate into effector T cells with potent cytotoxic activity; some subsequently develop into memory T cells [153, 154, 155]. In contrast, under persistent antigen exposure, Naïve T cells gradually diverge from the memory differentiation pathway and enter an exhaustion trajectory, characterized by the emergence of precursor T_PEX_ that maintain self‐renewal capacity but exhibit gradually diminished effector functions [156, 157]. This transition is driven by sustained TCR signaling, which induces key transcription factors such as TOX and TCF‐1 [121]. TOX promotes chromatin remodeling and exhaustion‐associated gene programs, while TCF‐1 preserves the “precursor” state and functional plasticity [8, 158]. With the continuous stimulation of antigens, T_PEX_ cells gradually downregulate the expression of TCF‐1 and differentiate into transitional T_EX‐int_ cell subsets [159]. These subsets exhibit cytokine secretion and enhanced cytotoxic activity. However, they have irreversibly diverged from the memory/stem lineage [160]. Further differentiation leads to the T_EX‐term_ cell stage, characterized by dominant TOX activity and accompanied by significant epigenetic alterations such as DNA methylation and histone modifications [123]. This process stabilizes the state of depletion even after antigen clearance. T_EX‐term_ cells display severe functional impairment, including loss of proliferative capacity, reduced cytokine production (e.g., IFN‐γ and TNF‐α), increased secretion of inhibitory cytokines (e.g., IL‐10), and high expression of multiple inhibitory receptors (e.g., PD‐1, CTLA‐4, TIM‐3, LAG‐3), rendering them refractory to ICB therapy [161, 162]. Notably, the proportion and functional status of the T_PEX_ subpopulation directly influence ICB efficacy and tumor immune control, suggesting that targeting the regulation of differentiation trajectories, maintaining the T_PEX_ state, or blocking the differentiation to T_EX‐term_ cell is a cutting‐edge direction for enhancing the effectiveness of immunotherapy [163, 164, 165]. Recent single‐cell multi‐omics investigations increasingly confirm that T cell exhaustion is not a simple linear process but involves a spectrum of rare and transitional subsets with distinct molecular and epigenetic features. For example, multi‐omics profiling in colorectal cancer identified TNFRSF18 and CXCL13 as dynamic markers of exhausted CD8^+^ T cells, with TNFRSF18‐high cells representing an early, stem‐like exhausted state and CXCL13‐high cells marking more terminally exhausted populations. The transition from TNFRSF18 to CXCL13 expression parallels disease progression and is accompanied by changes in ribosomal stemness, highlighting a previously unappreciated link between tumor cell stemness and T cell exhaustion [166]. It should be noted that current research on temporal dynamics primarily focuses on CD8^+^ T cells, while temporal heterogeneity in other T cell subsets (e.g., CD4^+^ T cells) remains underexplored. For a detailed classification of CD8^+^ T_EX_ cell subsets, refer to Section 3.4.1.

### 3.2 Heterogeneity in Spatial Distribution

In the tumor microenvironment, the spatial distribution of T cell exhaustion exhibits marked heterogeneity. Numerous single‐cell and spatial transcriptomic studies have demonstrated that T_EX‐term_ cells are predominantly localized within the tumor core—a region characterized by profound immunosuppression, nutrient deprivation, and hypoxia—conditions that collectively impair T cell functionality [128, 167, 168]. Similarly, omics analyses in extramedullary myeloma (EMD) have further revealed that TIM3^+^ PD‐1^+^ T cells closely colocalize with myeloma cells in the tumor core, where enhanced activity of the oxidative phosphorylation pathway contributes to the exacerbation of T cell dysfunction [169]. In contrast, T_PEX_ cells are preferentially enriched at the tumor periphery or invasive margin. These cells possess stem‐like properties, including self‐renewal capacity and high expression of TCF‐1, and are associated with robust immune responsiveness and greater sensitivity to ICB therapy [125, 135]. Moreover, specific subsets such as CD160^+^ CD8^+^ T cells demonstrate anti‐exhaustion and anti‐tumor activities within the tumor‐normal interface, highlighting the critical influence of the local microenvironment on T cell functional states [170, 171]. Notably, integrated spatial transcriptomic and single‐cell sequencing analyses have identified a rare transitional subset of NK‐like exhausted T cells at the tumor edge or invasion front. These cells frequently express natural killer (NK)‐associated receptors (e.g., members of the KLR family), co‐express partial effector functions and exhaustion markers, and may serve as a key cellular substrate underlying responses to immunotherapy [122, 160].

Recent studies have demonstrated that tertiary lymphoid structures (TLS), as ectopic immune activation sites within tumors, play a critical role in shaping spatial heterogeneity and sustaining T cell functionality [172, 173]. Emerging evidence from spatial omics and multiplex immunohistochemical analyses indicates that mature TLS (mTLS), functioning as newly formed lymphoid‐like organs in the tumor microenvironment, serve as niches for TCF1^+^ stem‐like CD8^+^ T cells and pre‐exhausted CD4^+^ T cells. These structures facilitate a multicellular cooperative network centered on B cells and DCs, thereby promoting sustained T cell activation and differentiation, supporting their proliferative capacity, and enhancing antitumor immunity [126, 174]. The presence of mTLS is strongly associated with improved survival prognosis and enhanced response to ICB. Spatial transcriptomic and imaging mass cytometry analyses have revealed that CD39^+^ PD‐1^+^ CD8^+^ stem‐like T cells are highly enriched within TLS and their surrounding regions. The density of T_PEX_ in TLS‐positive tumors is significantly higher than in TLS‐negative counterparts, indicating that TLS serves as a reservoir for T_PEX_ and facilitates their migration into the tumor parenchyma, thereby augmenting anti‐tumor immunity and ICB efficacy [126]. Mechanistically, mTLS promotes the sustained recruitment and functional maintenance of tumor‐specific T cells by enriching and activating T_PEX_, CD4^+^ T follicular helper (T_fh_) cells, and B cells. This process suppresses the accumulation of T_EX‐term_ cells, delays T cell dysfunction, and enhances responsiveness to ICB. In contrast, in the absence or dysfunction of TLS, the proportion of T_PEX_ cells declines while the proportion of T_EX‐term_ cells increases, contributing to immunotherapy resistance and an elevated risk of tumor immune escape [126, 174, 175]. Notably, the maturity and spatial distribution of TLS critically influence their immunomodulatory function. mTLS located within the tumor core are enriched with memory B cells, plasma cells, and CD4^+^ T cells, and promote B cell activation, antibody production, and sustained T cell activation. These features collectively enhance intratumoral anti‐tumor immunity and correlate with improved patient survival [174]. In contrast, immature TLS (imTLS) or those at the tumor periphery are enriched in endothelial cells, fibroblasts, and regulatory T cells, upregulate expression of epithelial‐mesenchymal transition (EMT)‐related genes, and may promote immunosuppression and tumor progression [127, 176]. Thus, TLS not only provides a spatial niche for the differentiation and activation of T cell subsets but also, through their maturity and anatomical localization, determines whether the immune microenvironment exhibits an anti‐tumor or pro‐tumorigenic phenotype.

### 3.3 Specificity Across Cancer Types

Although T cell exhaustion is commonly observed across various malignant tumors, the specific phenotype, regulatory mechanisms, and therapeutic responses exhibit considerable heterogeneity among different cancer types. This variability arises from the distinct tissue microenvironments, persistent antigen exposure, and metabolic characteristics inherent to each tumor type, collectively shaping a disease‐specific T cell exhaustion landscape.

#### 3.3.1 Lung Cancer

The regulatory network underlying T cell exhaustion in lung cancer exhibits distinct molecular features and spatial distribution patterns. Mechanistic investigations have demonstrated that Rab37, a small GTPase, mediates the intracellular trafficking and membrane localization of PD‐1 in a lung cancer‐specific manner. Impaired Rab37 function results in intracellular accumulation of PD‐1 and disrupts its proper translocation to the cell membrane, thereby sustaining exhaustion signals and establishing the Rab37/PD‐1/TIM‐3 signaling axis as a potential prognostic biomarker in lung cancer [177]. In addition, recent single‐cell multi‐omics analyses have revealed that casein kinase II (CK2) upregulates TBX21 expression through HDAC8‐mediated epigenetic reprogramming, thereby reversing CD8^+^ T cell exhaustion and enhancing responsiveness to anti‐PD‐1 therapy in non‐small cell lung cancer (NSCLC). These findings indicate that CK2B is a potential therapeutic target for restoring the function of exhausted effector CD8^+^ T cells [129]. Notably, cross‐tissue single‐cell sequencing has systematically uncovered the spatial heterogeneity of the exhausted T cell population in NSCLC. The study demonstrated that T_PEX_ cell subsets are predominantly enriched in tumor‐draining lymph nodes, whereas terminally exhausted subsets are localized within the tumor core, delineating distinct differentiation trajectories and migratory patterns [178]. At the level of the immune microenvironment, M2 macrophages in small cell lung cancer (SCLC) contribute to CD8^+^ T cell inactivation through the recruitment of FOXP3^+^ Tregs and the upregulation of PD‐L1 and other immunosuppressive factors. This fosters an “immune‐excluded” microenvironment, where increased immune cell infiltration is accompanied by functional impairment [179]. Collectively, these findings elucidate the multi‐scale regulation of T cell exhaustion in lung cancer and provide a rational foundation for developing combination immunotherapies targeting distinct phases of T cell dysfunction and the associated tissue microenvironment.

#### 3.3.2 Breast Cancer

The heterogeneous mechanisms underlying CD8^+^ T_EX_ cell in breast cancer are primarily driven by molecular subtype‐specific factors, particularly in ERα‐negative subtypes such as triple‐negative breast cancer (TNBC) [180, 181]. In ERα‐negative tumors, twist family BHLH transcription factor 1 (TWIST1) binds to the PD‐L1 promoter and recruits the TIP60 acetyltransferase complex via BRD8, leading to increased PD‐L1 expression and enhanced CD8^+^ T_EX_ cells [182]. Furthermore, extracellular vesicles secreted by breast cancer cells deliver the TGF‐β type II receptor (TβRII) to CD8^+^ T cells, thereby activating the SMAD3‐TCF1 signaling axis and promoting T cell exhaustion [16]. This immunosuppressive pathway is especially pronounced in TNBC, where the deubiquitinating enzyme USP8 stabilizes TβRII, thereby enhancing sustained TGF‐β signaling and immune evasion. Concurrently, exosome‐mediated transfer of immune cell adhesion molecule 1 (ICAM1) contributes to bone metastasis in TNBC through a related mechanism [183]. In addition, single‐cell transcriptomics and spatial proteomics have revealed that PD‐1^high^ CXCL13^+^ T_EX_ cells in breast cancer are spatially associated with an inflammatory immune microenvironment and imTLS, exhibiting significant functional and spatial heterogeneity across subsets [131, 132]. These findings elucidate subtype‐specific immune escape mechanisms in breast cancer and identify potential therapeutic targets for combination immunotherapies targeting distinct molecular pathways.

#### 3.3.3 Glioblastoma

Glioblastoma (GBM) is characterized by a distinct immunosuppressive tumor microenvironment, in which T cell exhaustion is particularly pronounced among solid tumors [184]. A cross‐cancer comparative study revealed that GBM‐infiltrating T cells display an atypical exhaustion phenotype compared to those in melanoma, breast cancer, and lung cancer [185]. Specifically, clonally expanded infiltrating T cell populations often exhibit low PD‐1 and TIM‐3 expression and tend to differentiate into a GZMK^+^ effector‐like phenotype with limited cytotoxic capacity [186]. Single‐cell lineage tracing and multi‐omics analysis further revealed that myeloid cells—including TAMs and microglia—play a pivotal role in promoting T cell exhaustion through the secretion of immunosuppressive cytokines such as IL‐10 and TGF‐β, as well as the expression of inhibitory ligands including PD‐L1 and B7‐H3 [187]. Furthermore, anatomical barriers such as the blood‐brain barrier and bone marrow sequestration restrict T cell infiltration, while metabolic reprogramming‐induced nutrient competition and hypoxia collectively contribute to a complex, multilayered immunosuppressive milieu in GBM [188, 189]. Notably, a significant sex‐based difference in T cell exhaustion has been observed in GBM. Male CD8^+^ T cells demonstrate a greater propensity for exhaustion, evidenced by a higher proportion of progenitor‐exhausted subsets, whereas female T cells preserve stronger effector functionality. This sexual dimorphism is driven by the X‐chromosome escape gene Kdm6a, highlighting unique features of the GBM immune landscape [190]. Furthermore, another study demonstrated that the knockdown of tumor necrosis factor receptor type II (TNFR2) led to the emergence of a T cell population expressing TIM3, yet characterized by reduced TOX expression and altered functional properties, encompassing both precursor and terminally exhausted subsets. In a glioma mouse model, inhibition of TNFR2 signaling significantly prolonged the overall survival of tumor‐bearing mice [191]. These findings may provide new insights into the understanding of T cell exhaustion within intracranial tumor microenvironments. Clinical evidence further indicates that GBM patients experiencing disease progression following anti‐angiogenic therapy exhibit a markedly exacerbated state of T cell exhaustion, characterized by increased Treg infiltration, upregulated PD‐1 expression, and functional impairment—highlighting the intricate dynamic interplay between therapeutic response and immune modulation [192].

#### 3.3.4 Colorectal Cancer

The heterogeneity of T cell exhaustion in colorectal cancer (CRC) exhibits distinct organ‐specific characteristics, primarily shaped by the unique immune microenvironment, molecular subtypes, and metabolic reprogramming within the gut. A recent landmark study has identified thyroid‐stimulating hormone (TSH)/thyroid‐stimulating hormone receptor (TSHR) signaling as a key intrinsic pathway that promotes CD8^+^ T cell exhaustion in CRC. Specifically, cancer cells deliver the TSHR to CD8^+^ T cells via the exosomal pathway, thereby inhibiting their effector differentiation—a mechanism that plays a pivotal role in establishing the CRC‐specific exhausted microenvironment [135]. In contrast, by comparing ileal and colonic samples from CRC patients with those from healthy donors, a group of researchers recently identified an ileum‐enriched population of CD160^+^ CD8^+^ T cells that exhibit resistance to terminal exhaustion and possess robust clonal expansion capacity. In both microsatellite instability‐high (MSI‐H) and anti‐PD‐1‐resistant CRC models, CD160^+^ CD8^+^ T cells enhance tumor‐infiltrating progenitor‐exhausted T cells, improve anti‐PD‐1 efficacy, and overcome therapeutic resistance, thereby significantly augmenting antitumor immunity [134]. In microsatellite stable (MSS) tumors, CD8^+^ T_EX_ cells co‐express PD‐1 and TIGIT, display reduced cytokine secretion, and exhibit limited cytotoxic function, consistent with features of terminal exhaustion [193]. Furthermore, gut‐specific dysregulation of ammonia metabolism exacerbates T cell exhaustion through metabolic reprogramming, which may partially account for the widespread resistance to ICB observed in CRC and provides a rationale for therapeutic strategies targeting ammonia clearance [170].

#### 3.3.5 Hematologic Malignancies

Hematologic malignancies arise within the hematopoietic system, and their systemic dissemination confers unique dynamic features to T cell exhaustion. In B‐cell acute lymphoblastic leukemia (B‐ALL), CD4^+^ T cells—rather than CD8^+^ T cells—are identified as the primary population undergoing exhaustion. In a clinical study of children with B‐ALL, researchers observed that CD4^+^ T cells in the bone marrow exhibited elevated expression of exhaustion markers, including TIM‐3 and PD‐1, along with impaired effector function and a diminished anti‐leukemic response—features strongly associated with a significantly increased risk of disease recurrence. Patients with a high proportion of TIM‐3^+^ CD4^+^ T cells in the bone marrow at diagnosis had a 7.1‐fold increased risk of relapse, indicating that TIM‐3‐mediated dysfunction of CD4^+^ T cells plays a critical role in immune escape and disease recurrence in B‐ALL [137]. This exhaustion may be driven by leukemia‐induced TIM‐3 signaling, particularly through CD200/TIM‐3 interactions, rather than the classical PD‐1/PD‐L1 pathway [137]. On the other hand, Sean I. Tracy et al. revealed, through single‐cell sequencing of bone marrow T cells from patients with B‐ALL, that tumor‐T_EX_ encompasses multiple differentiation states and functional lineages. The study identified two distinct CD4^+^ T cell subsets, designated as Thctx (T helper/cytotoxic) and exhausted Thctx (Thctx‐ex). These cells co‐expressed cytotoxic genes such as GZMB and NKG7, as well as helper cytokine genes including IFNG and TNF, indicating dual functional capabilities encompassing both cytotoxic and helper activities. Furthermore, the study demonstrated that the combination of the BCR‐ABL tyrosine kinase inhibitor nilotinib and the anti‐PD‐L1 monoclonal antibody significantly reversed this exhausted phenotype, thereby promoting clonal expansion of leukemia‐specific CD4^+^ T cells and markedly improving long‐term survival in a murine model of leukemia [138]. Compared with B‐ALL, other hematological malignancies, such as multiple myeloma (MM), exhibit a more complex immune microenvironment. Studies have shown that T cells in the bone marrow and peripheral blood of MM patients display elevated expression of exhaustion markers, including PD‐1, TIM‐3, and TOX. However, the classical terminal exhaustion phenotype is not prominently observed, and exhausted T cell clones are typically small in size and non‐persistent. Functional impairment in these cells is primarily due to the immunosuppressive microenvironment and defects in antigen presentation, rather than classical antigen‐driven exhaustion [194, 195]. This provides a mechanistic explanation for the limited efficacy of PD‐1/PD‐L1 inhibitors in MM. Similarly, in diffuse large B‐cell lymphoma (DLBCL), recent evidence indicates that although certain CD8^+^ T cells exhibit features of terminal exhaustion (e.g., IFN‐γ^+^ T_PEX_), they retain cytotoxic activity and cytokine secretion capacity—distinct from the functionally impaired T_EX_ cells observed in chronic infections [196]. In contrast, CD8^+^ T cells may express exhaustion markers such as PD‐1 without complete loss of function in some contexts, which may account for the modest response of DLBCL to single‐agent ICIs.

#### 3.3.6 Hepatocellular Carcinoma

The exhaustion phenotype in hepatocellular carcinoma (HCC) is driven not only by persistent tumor antigen stimulation but also by the liver's innate immune regulatory mechanisms and the presence of viral antigens, resulting in a more complex and sustained state of T cell dysfunction [171]. Transcriptomic analyses have demonstrated that TIGIT, an inhibitory receptor, is significantly more upregulated in HCC‐specific exhausted CD8^+^ T cells compared to PD‐1, suggesting that TIGIT may serve as a more reliable biomarker for identifying this subset of dysfunctional T cells [197]. Furthermore, the unique immune‐tolerant microenvironment characteristic of HCC influences the distinct differentiation trajectory of T_EX_ cells. This microenvironment is enriched with Tregs, MDSCs, and immunosuppressive cytokines such as TGF‐β and IL‐10, which collectively foster a highly tolerant milieu and promote the accelerated progression of CD8^+^ T cells toward terminal exhaustion—a state typically more profound and durable [198]. Notably, approximately 80% of HCC cases are associated with chronic HBV or HCV infection [199]. In such contexts, virus‐specific and tumor‐specific T cells coexist within the same microenvironment and are subjected to prolonged antigenic stimulation. This dual pressure from viral and tumor antigens leads to earlier and more robust establishment of epigenetic programs linked to T cell exhaustion, thereby limiting the responsiveness of these cells to ICB therapies, including PD‐1 monotherapy [200, 201, 202]. Additionally, the liver‐specific metabolic environment—particularly the elevated levels of ROS and lactic acid resulting from mitochondrial dysfunction—exacerbates T cell exhaustion through metabolic stress, further impairing anti‐tumor immunity [203].

#### 3.3.7 Cervical Cancer

Cervical cancer (CESC) exhibits a distinct pattern of T cell exhaustion, primarily attributable to its well‐established viral etiology. The persistent expression of human papillomavirus (HPV)‐derived E6 and E7 oncoproteins serves as a shared tumor‐associated antigen, driving sustained and intense activation of virus‐specific T cell clones. This chronic stimulation promotes early and stable induction of T cell exhaustion program via NFAT‐TOX‐mediated epigenetic remodeling [139, 204, 205, 206]. Beyond this canonical viral‐driven mechanism, CESC also demonstrates unique metabolic dysregulation in the tumor microenvironment. Specifically, tumor cells overexpress SERINC2, leading to competitive depletion of serine, which results in metabolic deprivation of T cells. This nutrient limitation directly impairs T cell proliferation and effector functions, thereby accelerating their functional exhaustion [140]. In addition, spatial omics and immunohistochemistry confirmed that tumor‐specific B cells and SPP1^+^ macrophages interact closely with T cell subsets to collectively shape the immunosuppressive tumor microenvironment [207, 208]. At the immune checkpoint level, in addition to upregulation of classical inhibitory receptors such as PD‐1 and TIM‐3, CESC exploits the non‐classical checkpoint B7‐H3, which aberrantly engages the costimulatory receptor 4‐1BB to deliver inhibitory signals—a mechanism that constitutes a distinctive pathway of immune evasion [209, 210]. Recent studies have reported that MiR‐379‐5p, as an epigenetic tumor suppressor, may serve as a novel biomarker for cancer immunotherapy by enhancing the function of CD8^+^ T effector cells and inhibiting T cell exhaustion [211]. Collectively, these molecular and immunological features underscore the potential responsiveness of CESC to emerging therapeutic strategies, including HPV antigen‐targeted vaccines, metabolic modulation, and B7‐H3‐directed interventions.

### 3.4 Heterogeneity of T Cell Exhaustion Subsets

T cell exhaustion manifests across diverse T cell lineages, including CD8^+^, CD4^+^, γδ T cells, and mucosa‐associated invariant T (MAIT) cells. However, the underlying regulatory mechanisms exhibit both conserved and subset‐specific features. Common drivers include persistent antigen stimulation, sustained expression of inhibitory receptors (e.g., PD‐1, TIM‐3), epigenetic reprogramming mediated by transcription factors such as TOX and NR4A, and metabolic dysregulation. In contrast, the functional specialization and differentiation pathways of each subset also confer unique exhaustion phenotypes and therapeutic vulnerabilities.

#### 3.4.1 CD8^+^ T Cell Exhaustion Subsets

The heterogeneity of CD8^+^ T cell exhaustion subsets has been elucidated through single‐cell omics and functional studies, revealing major populations including T_PEX_, T_EX‐int_, and T_EX‐term_ cells. These subsets exhibit multi‐level complexity in terms of spatial distribution, molecular signatures, functional properties, and clinical relevance [122, 142]. The T_PEX_ subset is characterized by high expression of TCF‐1 (Tcf7), Ly108 (SLAMF6), and CD69, and is predominantly localized in lymphoid tissues, tumor‐associated TLS, and the tumor stroma. T_PEX_ cells possess self‐renewal capacity and robust proliferative potential, with low expression of inhibitory receptors. They represent a key target for ICB therapy and play a pivotal role in mediating anti‐tumor immune responses and determining ICB efficacy [122, 141]. The T_EX‐int_ subset is defined by high T‐bet expression, CX3CR1 positivity, and partial retention of effector functions, such as the secretion of IFN‐γ and TNF‐α, as well as cytotoxic activity. Functionally and phenotypically intermediate between T_PEX_ and T_EX‐term_ cells, T_EX‐int_ cells can respond to ICB and contribute to anti‐tumor immunity, although they have irreversibly lost stem‐like properties [74]. Recent single‐cell omics analyses have further identified rare transitional subpopulations within the T_EX_ cell compartment, including NK‐like, ISG+, and other novel intermediate states, which express distinct molecular markers such as those from the KLR family, CXCL13, and TNFRSF18. These findings have significantly expanded the heterogeneity landscape of T cell exhaustion [122, 166, 212, 213]. However, the functional roles and clinical implications of these emerging subsets remain unclear. Their inclusion in the canonical classification framework requires further validation. The T_EX‐term_ cell subset is characterized by high expression of transcription factors such as TOX, EOMES, and NR4A, along with upregulation of surface molecules including CD101, TIM‐3, and LAG‐3. It is predominantly localized within the tumor parenchyma and exhibits severe functional impairment, epigenetic fixation, and poor responsiveness to ICB. As such, it represents a major contributor to immunotherapy resistance and tumor immune escape [142, 143]. Recent pan‐cancer single‐cell transcriptomic studies have established a unified clustering framework for CD8^+^ T_EX_ cells across species and tumor types, identifying six distinct subsets (C1–C6) with divergent functional and molecular profiles. Notably, the C1 subset is significantly expanded following ICB treatment, suggesting its potential as a predictive biomarker for therapeutic response [143]. In summary, the spatial distribution and functional states of CD8^+^ T_EX_ cell subsets exhibit considerable plasticity and dynamic variation across different tumor types and tissue microenvironments [143, 213, 214].

#### 3.4.2 CD4^+^ T Cell Exhaustion Subsets

CD4^+^ T cells are core coordinators of immune responses, and their exhaustion affects the integrity of the tumor immune network [146]. Recent studies have demonstrated that CD4^+^ T cells not only share a high degree of phenotypic and differentiation similarity with CD8^+^ T cells, but also possess distinct subset characteristics and regulatory mechanisms [146, 215]. T_fh_ cells were divided into non‐cytotoxic and cytotoxic groups. The former had high expression of TCF1, low BLIMP1 and LAG‐3, self‐renewal ability, and sensitivity to PD‐1 monoclonal antibody, similar to CD8^+^ T_EX_ cell subsets. The latter group has low TCF1, high BLIMP1 and LAG‐3, loss of self‐renewal, and is resistant to PD‐1 monoclonal antibody, but may be sensitive to PD‐1/LAG‐3 dual blockade, corresponding to the terminal exhaustion state [145, 146, 216]. This parallel differentiation pattern suggests a partially conserved regulatory mechanism underlying T cell exhaustion in both CD4^+^ and CD8^+^ T cell lineages.

Additionally, Tregs exhibiting exhaustion‐associated phenotypes (e.g., upregulation of inhibitory receptors such as PD‐1, TIM‐3, and altered suppressive function) are increasingly recognized as a distinct functional state [146, 147, 217]. Traditionally, Tregs have been regarded as key mediators of immunosuppression [218]. However, in the contexts of chronic inflammation and the tumor microenvironment, a subset of Tregs upregulates multiple inhibitory receptors, such as PD‐1, TIGIT, and TIM‐3, acquiring enhanced immunosuppressive capacity, while potentially compromising their lineage stability [219, 220, 221]. In this “super‐suppressive” state, Tregs not only exert more potent inhibition on effector T cell function but also actively contribute to shaping an immunosuppressive microenvironment through the secretion of cytokines, including IL‐10 and TGF‐β [15, 222, 223]. However, it is important to note that there exists a critical distinction between the “super inhibitory” activity of Tregs exhibiting exhaustion‐associated phenotypes and their therapeutic capacity in maintaining systemic immune homeostasis. Lamarche et al. demonstrated that although exhausted CAR‐Tregs exhibited enhanced suppressive function in vitro, they completely lost their protective efficacy against graft‐versus‐host disease (GVHD) in vivo [224]. This discrepancy highlights the functional complexity of Tregs and underscores the necessity of relying on in vivo models for accurate evaluation of cellular therapies. This loss of in vivo functionality may be attributed to metabolic rigidity and epigenetic stabilization associated with T cell exhaustion, which could impair adaptability in complex physiological environments.

It is worth emphasizing that although both CD8^+^ and CD4^+^ T cell exhaustion are characterized by functional impairment and elevated expression of inhibitory receptors driven by persistent antigen stimulation and an immunosuppressive microenvironment, there exist significant differences as well as overlapping features between these subsets in terms of molecular mechanisms, phenotypic characteristics, and functional outcomes. The exhaustion trajectory of CD8^+^ T cells is relatively well defined, involving a stepwise lineage differentiation from T_PEX_ to T_EX‐int_ and ultimately to T_EX‐term_ cells [225]. In contrast, CD4^+^ T cell exhaustion follows a more heterogeneous differentiation pathway, encompassing both stem‐like to terminal differentiation within T**_fh_**‐like subsets and the acquisition of a “hypersuppressive” state in Tregs [144, 216]. Functionally, CD8^+^ T cell exhaustion primarily results in diminished cytotoxic capacity against tumors, whereas CD4^+^ T cell exhaustion indirectly impairs CD8^+^ T cell activity, dendritic cell maturation, and immune microenvironment remodeling through dysfunctions in helper, regulatory, and cytotoxic functions [144, 215]. Moreover, exhausted CD4^+^ T cells promote the formation of tumor‐associated tertiary lymphoid structures by secreting chemokines such as CXCL13, thereby facilitating the recruitment and activation of CXCR5^+^ CD8^+^ T_PEX_ cells [226, 227]. Both subsets play synergistic roles in tumor progression and immune escape, underscoring the importance of developing coordinated targeting strategies in future cancer immunotherapies.

#### 3.4.3 Unconventional T Cell Exhaustion

In addition to the well‐documented depletion of CD8^+^ and CD4^+^ T cells, the functional exhaustion of γδ T cells has increasingly drawn attention in cancer research in recent years [228]. γδ T cells represent a distinct subset of T lymphocytes that are independent of MHC restriction and antigen‐presenting cells, and they play a critical role in immune surveillance and host defense through the secretion of diverse cytokines [229]. However, within the tumor microenvironment, γδ T cells frequently exhibit functional exhaustion. For instance, a recent single‐cell RNA sequencing (scRNA‐seq) study in a murine model of colon cancer identified tumor‐infiltrating CD8α^−^ PD‐1^+^ γδ T cells with downregulated expression of cytotoxicity‐associated genes and upregulated expression of IL‐17‐related and tumor‐promoting genes [230]. More importantly, a mechanistic investigation in cervical cancer demonstrated that BTN3A1 expressed on tumor cell surfaces activates the TCR signaling pathway, leading to increased expression of the exhaustion‐associated transcription factors NR4A2 and NR4A3, thereby driving Vδ2 T cells into a state of functional exhaustion [148]. This finding provides a defined molecular mechanism underlying Vδ2 T cell exhaustion. Furthermore, terminally differentiated Vδ2^+^ γδ T cells display an exhausted phenotype that correlates with lymph node metastasis, and elevated PD‐1 expression on these cells in the peripheral blood of breast cancer patients underscores their potential as a biomarker for disease progression [231]. PD‐1 blockade has been shown to significantly restore the cytotoxic function of tumor‐infiltrating γδ T cells and enhance the antitumor efficacy of γδ T cell‐based adoptive immunotherapy in a murine model of prostate cancer [149]. Clinical studies have further demonstrated that the functional capacity of Vδ2^+^ γδ T cells is restored in a subset of patients with solid tumors following PD‐1/PD‐L1 immune checkpoint blockade, indicating that γδ T cell dysfunction is reversible and may represent a viable target for immunotherapeutic intervention. Similarly, tumor‐infiltrating Vγ9^−^ γδ T cells are frequently observed in high numbers in both solid tumors and lymphomas. Their abundance correlates with the extent of tissue residency and exhaustion and exhibits significant similarity to CD8^+^ T cells in terms of differentiation trajectory, gene expression profiles, and responsiveness to immune checkpoint therapy [232]. Collectively, these findings suggest that activated γδ T cells in the tumor microenvironment display exhaustion features analogous to those of CD8^+^ T cells, characterized by concurrent and sustained upregulation of immune checkpoint molecules—although this phenotype extends beyond checkpoint expression alone.

The exhaustion of MAIT cells has garnered significant attention in recent years [233]. MAIT cells play a critical role in mucosal immunity through the recognition of microbial metabolites presented by MR1 molecules. Evidence indicates that tumor‐infiltrating MAIT cells exhibit high expression of exhaustion markers, including PD‐1, Tim‐3, and CD39, consistent with a terminally exhausted phenotype. Although these cells retain some proliferative capacity, their functional versatility is markedly impaired, particularly in terms of the production of key anti‐tumor cytokines such as IFN‐γ and TNF [150]. At the same time, recent studies have demonstrated that in cancer‐associated chronic conditions such as HCC, abnormal accumulation of polyunsaturated fatty acids (PUFAs) in MAIT cells induces lipid peroxidation, leading to impaired mitochondrial respiration and glycolysis and establishing a state of “metabolic exhaustion.” Sustained lipid peroxidation ultimately triggers ferroptosis, resulting in functional impairment and cell death of MAIT cells [151]. Encouragingly, in vitro studies have demonstrated that PD‐1 blockade can partially restore MAIT cell function, suggesting that this subset may represent a promising target for ICB therapies [150].

In summary, T cell exhaustion across different subsets shares a common molecular framework centered on the TOX/NR4A‐driven transcriptional program, epigenetic fixation, and upregulation of PD‐1, while also exhibiting significant heterogeneity due to differences in antigen recognition patterns, functional specialization, and microenvironmental cues. For CD8^+^ T cells, the primary therapeutic target is PD‐1 blockade to restore cytotoxic function. For CD4^+^ T cells, intervention should focus on correcting the imbalance between helper and suppressor activities. For γδ T cells, strategies must address their MHC‐unrestricted activation pathways. Future therapeutic approaches should integrate broad‐spectrum checkpoint inhibitors with subset‐specific targeting modalities to achieve precise immune reconstitution.

## 4 Biomarkers of T Cell Exhaustion

The establishment and maintenance of T cell exhaustion represent a dynamic process that is tightly regulated by multilayered molecular networks. These molecules not only serve as “identity markers” for identifying exhausted states but also function as key effectors directly involved in driving T cell dysfunction. This section provides a systematic overview of the critical biomarkers defining T cell exhaustion, organized across three dimensions: surface inhibitory receptors, core transcription factors, and metabolism‐associated molecules, along with an in‐depth analysis of their intrinsic regulatory mechanisms.

### 4.1 Surface Inhibitory Receptors

**FIGURE 4:**
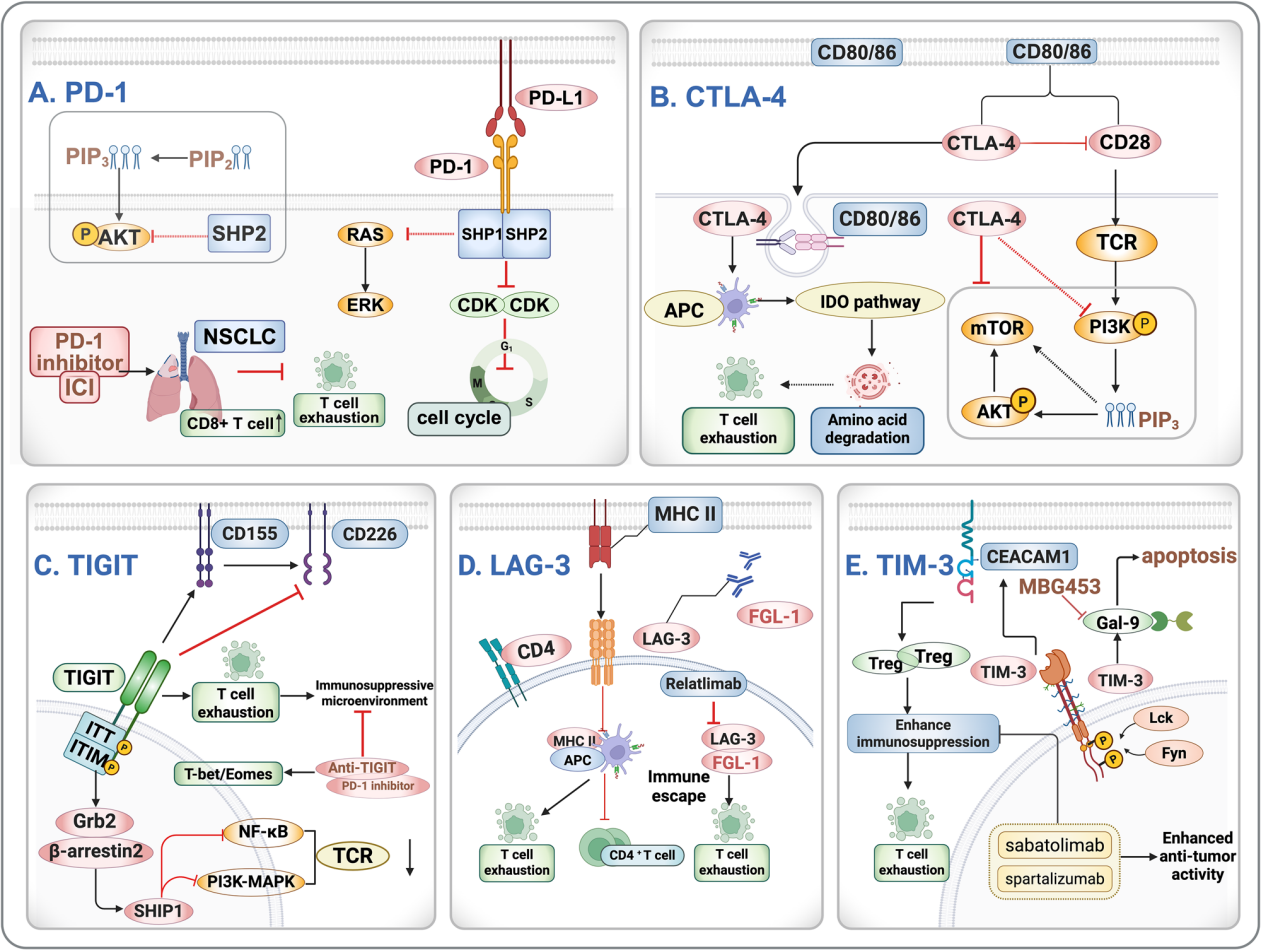
Diagram of surface inhibitory receptors and mechanisms of T cell exhaustion. (A) PD‐1, one of the most prominent markers of T cell exhaustion, suppresses downstream AKT and ERK activation by engaging PD‐L1 or PD‐L2 and recruiting phosphatases that propagate inhibitory signaling pathways. Additionally, PD‐1 can impede cell cycle progression through inhibition of CDKs. ICIs targeting the PD‐1 pathway have emerged as a cornerstone in the treatment of various malignancies. (B) CTLA‐4 competitively binds to CD80 and CD86, thereby attenuating the CD28‐mediated costimulatory signal and indirectly promoting T cell exhaustion via activation of the IDO pathway. (C) Following PD‐1 and CTLA‐4, TIGIT has emerged as a promising novel target in cancer immunotherapy. Upon activation, TIGIT interferes with the PI3K‐MAPK and NF‐κB signaling cascades, dampens TCR signaling, and is closely associated with T cell exhaustion across multiple cancer types. (D) LAG‐3 is highly expressed on exhausted T cells, and its interaction with MHC‐II and FGL‐1 compromises antigen presentation by APCs or directly suppresses T cell function. Therapeutic strategies targeting LAG‐3, particularly in combination with PD‐1 blockade, have demonstrated enhanced efficacy in restoring anti‐tumor T cell responses. (E) Similarly, TIM‐3 functions as a negative regulatory receptor that promotes T cell exhaustion in both tumor microenvironments and chronic viral infections; its engagement with ligands such as Gal‐9 can trigger apoptosis or augment immunosuppressive mechanisms. Currently, numerous immunotherapies directed against these inhibitory receptors, particularly combinatorial approaches, are advancing rapidly, offering new prospects for overcoming T cell exhaustion and reinvigorating anti‐tumor immunity, with notable achievements already observed in clinical settings. A comprehensive understanding of the intricate regulatory mechanisms governing these receptors, as well as their context‐dependent roles across diverse cell populations and tissue microenvironments, is critical for refining current therapeutic regimens and developing more precise, effective combination strategies. Abbreviations: PD‐1, programmed death protein 1; PD‐L1, programmed death‐ligand 1; PD‐L2, programmed death‐ligand 2; CDKs, cyclin‐dependent kinases; ICI, immune checkpoint inhibitor; CTLA‐4, cytotoxic T lymphocyte‐associated antigen 4; IDO, indoleamine 2,3‐Dioxygenase; TIGIT, T cell immunoreceptor with Ig and ITIM domains; TCR, T cell receptor; MHC‐II, MHC class II molecule; LAG‐3, Lymphocyte‐activation gene 3; FGL‐1, fibrinogen‐like protein 1; APC, antigen‐presenting cell; TIM‐3, T cell immunoglobulin and mucin‐domain containing‐3; Gal‐9, galectin‐9.

Under conditions of chronic antigen stimulation, T cells co‐express multiple inhibitory receptors on their cell surface, thereby establishing a synergistic immune checkpoint network that collectively suppresses T cell functionality. A comprehensive understanding of both the distinct and overlapping regulatory mechanisms of these receptors is fundamental to the development of effective combination immunotherapies (Figure 4).

#### 4.1.1 PD‐1

PD‐1, also known as CD279, is widely recognized as the most characteristic surface marker of T cell exhaustion [234]. It functions as a critical immunosuppressive molecule and is predominantly expressed on activated T cells, B cells, and macrophages, playing a significant role in immune regulation [235, 236]. As a key receptor on the surface of T cells, PD‐1 effectively suppresses T cell activation and dampens the immune response upon interaction with its ligands, thereby facilitating the transition of T cells into an exhausted state [237, 238]. To date, two ligands for PD‐1 have been identified: PD‐L1 (B7‐H1, CD274) and PD‐L2 (B7‐DC, CD273) [239]. The interaction between PD‐1 and its ligands is recognized as a pivotal immune checkpoint mechanism that negatively regulates T cell proliferation. Upon ligand binding, PD‐1 recruits intracellular SHP‐1 and SHP‐2, leading to the inhibition of key signaling molecules within the TCR signaling pathway [240].

Further mechanistic investigations have demonstrated that PD‐1 signaling mediates immunosuppression through multiple distinct pathways. First, it inhibits cell cycle progression by suppressing cyclin‐dependent kinase (CDK) activity [241, 242]. Second, it attenuates TCR signaling microcluster formation via downregulating CK2. Additionally, in the TME, sustained PD‐1 activation promotes epigenetic remodeling—such as DNA methylation and histone modifications—which stabilizes the T_EX_ cell phenotype [243, 245]. Moreover, PD‐1 signaling is not only prevalent in T cells during chronic viral infections but is also markedly upregulated in tumor‐infiltrating T cells, contributing to T cell exhaustion and impaired anti‐tumor immunity [246]. For instance, in approximately half of NSCLC patients treated with PD‐1 inhibitors, an increase in proliferating Ki‐67^+^CD8^+^ T cells was observed in peripheral blood, with these cells predominantly residing in the PD‐1‐expressing subset. Additional evidence indicates that the CD28/B7 costimulatory pathway plays a critical role in modulating this response [243]. Notably, Ma et al. reported that co‐expression of PD‐1 and TIM‐3 at high levels is strongly associated with functional impairment of CD8^+^ T cells and reduced survival in HCC patients, highlighting the importance of further evaluating these receptors for potential therapeutic targeting [247].

The sustained high expression of PD‐1 on T_EX_ cells has driven the development of ICB therapies targeting the PD‐1/PD‐L1 axis [248]. In 2014, the U.S. Food and Drug Administration (FDA) approved two PD‐1‐blocking monoclonal antibodies, pembrolizumab and nivolumab, for the treatment of advanced melanoma, squamous NSCLC, and renal cell carcinoma [249, 250]. In liver cancer research, GLIS family zinc finger 1 (GLIS1) has been identified as a transcriptional regulator of the SGK1‐STAT3‐PD‐1 signaling pathway, which maintains elevated PD‐1 expression on CD8^+^ T cells and promotes T cell exhaustion. Downregulation of GLIS1 expression has been shown to synergistically enhance CD8^+^ T cell infiltration and reduce their exhausted phenotype in combination with anti‐PD‐1 therapy, offering a novel and promising strategy for liver cancer immunotherapy [251]. Furthermore, PD‐1/PD‐L1 blockade has demonstrated the ability to augment the antitumor efficacy of peptide vaccines by promoting the induction of tumor‐specific CTLs in patients with HCC [252]. Despite these encouraging clinical advances, PD‐1 blockade still faces significant challenges. Multiple adaptive resistance mechanisms within the tumor microenvironment—including upregulation of alternative immune checkpoints, loss of costimulatory signals, and metabolic reprogramming—collectively contribute to heterogeneous treatment responses. These findings underscore the need for future research to comprehensively elucidate the regulatory mechanisms of the PD‐1 signaling network using multi‐omics approaches and to develop biomarker‐guided precision therapeutic strategies to overcome current limitations in immunotherapy [253].

#### 4.1.2 CTLA‐4

CTLA‐4 functions as a dominant suppressor at the immune synapse by competing with CD28 for binding to CD80 and CD86 [254]. CTLA‐4 exhibits significantly higher affinity for CD80/CD86 than CD28 and delivers a potent inhibitory signal through its cytoplasmic domain. Additionally, through the process of trans‐endocytosis, CTLA‐4 can directly remove CD80/CD86 from the surface of antigen‐presenting cells, thereby attenuating the costimulatory signals required for T cell activation [58, 255, 256, 257]. Notably, CTLA‐4 has been shown to induce the expression of IDO in antigen‐presenting cells, leading to the catabolism of tryptophan into kynurenine—a metabolite with immunosuppressive properties—thus indirectly contributing to T cell functional exhaustion [258]. Elevated CTLA‐4 expression has been consistently associated with T cell dysfunction across various tumor types. In patients with HCC and chronic hepatitis B, both peripheral and tumor‐infiltrating CD8^+^ T cells exhibit high levels of CTLA‐4 expression, which strongly correlates with impaired effector function [259]. Furthermore, in the ovarian cancer microenvironment, CTLA‐4 promotes the development of a T cell senescence phenotype by antagonizing CD28‐mediated signaling pathways [260].

Given the central role of CTLA‐4 in immune regulation, its targeted therapy has emerged as a promising strategy in tumor immunotherapy [261]. In clinical trials involving patients with metastatic melanoma, the CTLA‐4 inhibitor ipilimumab demonstrated significantly improved progression‐free survival compared to PD‐1 checkpoint inhibitor monotherapy, along with a lower incidence of adverse events [262]. In models of chronic HCV infection, CTLA‐4 and PD‐1 have been shown to induce T cell exhaustion in a functionally redundant manner, and dual blockade of these pathways can synergistically restore T cell function in a CD4‐independent but CD28‐dependent manner, highlighting their therapeutic potential [44]. However, in highly immunosuppressive tumor microenvironments such as those observed in pancreatic cancer, CTLA‐4 blockade enhances infiltration of CD4^+^ T cells but exerts limited effects on CD8^+^ T cell recruitment, indicating that the efficacy of this intervention is strongly influenced by tumor type‐specific factors [263]. These findings underscore the importance of further investigating cell‐type‐specific mechanisms underlying CTLA‐4‐mediated immune regulation. Future research should focus on developing precise patient stratification strategies based on the characteristics of the tumor immune microenvironment (TIME) as well as optimizing combination regimens involving CTLA‐4‐targeted therapies and other immunomodulatory approaches to achieve more effective antitumor immune responses.

#### 4.1.3 TIGIT

As an immune checkpoint molecule following CTLA‐4 and PD‐1, TIGIT has garnered significant attention, with its structure and functional mechanisms becoming increasingly well characterized [264]. The molecular architecture of TIGIT consists of an extracellular immunoglobulin variable (IgV) domain, a transmembrane domain, and an intracellular domain. The latter contains two highly conserved signaling motifs: an ITIM and an immunoreceptor tyrosine‐based tail (ITT)‐like motif [265]. Upon ligand binding, TIGIT undergoes phosphorylation and subsequently recruits adaptor proteins such as Grb2 and β‐arrestin 2, ultimately facilitating the recruitment of the SHIP1 phosphatase. This cascade leads to the negative regulation of the PI3K‐MAPK and NF‐κB signaling pathways, thereby directly suppressing TCR signaling and diminishing the activation capacity of CD8^+^ T cells. Additionally, TIGIT synergistically enhances Treg immunosuppressive function [266, 267]. TIGIT expression is closely associated with T cell exhaustion across various malignancies [268, 269]. It can be transiently upregulated following TCR activation and is stably expressed in NK cells, Tregs, T_fh_ cells, and CD8^+^ T_EX_ cells [120]. Notably, TIGIT is specifically and highly expressed in a well‐defined subset of exhausted CD8^+^ T cells—namely, TOX^hi^ TCF‐1^hi^ cells—in both human and murine models, and is widely regarded as a key marker of T cell exhaustion [197, 246]. Elevated TIGIT expression has also been significantly correlated with T cell exhaustion and poor clinical outcomes in patients with CRC, further underscoring its role as a central mediator of negative immune regulation [270].

The interaction of TIGIT with its multiple ligands plays a pivotal role in mediating immunosuppression. Known TIGIT ligands include CD155 (PVR), CD112, CD113, and Nectin‐4, among which CD155 is recognized as the primary high‐affinity ligand for TIGIT in both humans and mice [271, 272, 273]. Targeted inhibition of the TIGIT–ligand interaction, particularly with CD155, has emerged as a promising strategy to restore antitumor immune responses. For instance, in cervical cancer, Liu et al. demonstrated that elevated expression of CD155 and TIGIT within the TME is strongly associated with impaired immune cell function, and that blockade of the TIGIT/CD155 signaling axis can reverse CD8^+^ T cell exhaustion and enhance anti‐tumor immunity [139]. Similarly, Wu et al. reported in head and neck squamous cell carcinoma (HNSCC) that anti‐TIGIT therapy significantly ameliorates the immunosuppressive microenvironment by reducing ARG1 expression in MDSCs and suppressing TGF‐β1 secretion from Tregs [274]. Currently, several TIGIT‐blocking monoclonal antibodies are under clinical development. Notably, Ociperlimab (BGB‐A1217), a humanized IgG1 monoclonal antibody with intact Fc functionality, effectively inhibits the binding of TIGIT to CD155 and CD112. Preclinical studies have indicated that combining Ociperlimab with PD‐1/PD‐L1 inhibitors yields synergistic anti‐tumor effects [275]. Furthermore, in an autologous stem cell transplantation model of multiple myeloma, dual blockade of TIGIT and PD‐1 markedly prolonged tumor control, potentially through modulation of the T‐bet/Eomes expression ratio to enhance T cell cytotoxicity and suppress IL‐10‐mediated immune microenvironment remodeling [276]. With ongoing advances in understanding the TIGIT signaling pathway and its immunoregulatory functions, TIGIT‐targeted therapies—particularly in combination with other immunotherapeutic approaches—are anticipated to offer novel therapeutic opportunities for cancer patients.

#### 4.1.4 LAG‐3

LAG‐3 (CD223) is an inhibitory receptor that has garnered significant attention in the field of cancer immunotherapy in recent years. Marked upregulation of LAG‐3 on T_EX_ cells in chronic viral infections and the TME suggests that LAG‐3 blockade may restore antitumor T cell function [40, 277]. Structurally, LAG‐3 is highly homologous to CD4 but binds MHC class II molecule (MHC‐II) with much higher affinity [278]. Ei Wakamatsu et al. demonstrated that LAG‐3 can reduce surface expression of MHC‐II on antigen‐presenting cells (APCs) through trans‐endocytosis, thereby impairing antigen presentation efficiency and suppressing CD4^+^ T cell activation. Anti‐LAG‐3 therapy can directly inhibit this process, alleviate the intrinsic inhibitory signals mediated by LAG‐3, prevent downregulation of MHC‐II, and promote the functional recovery of T_EX_ cells, offering a promising strategy for cancer immunotherapy [279]. In addition to MHC‐II, fibrinogen‐like protein 1 (FGL‐1) has been identified as another key functional ligand of LAG‐3. FGL‐1, primarily secreted by hepatocytes, promotes HCC cell proliferation [280, 281]. The binding of elevated FGL1 to LAG‐3 within the TME drives the exhaustion of CD8^+^ T resident memory (T_RM_) cells within the tumor, thus facilitating tumor immune escape [282]. Given its critical role in tumor immune regulation, targeting the LAG‐3/FGL‐1 signaling axis represents a promising avenue for the development of novel immunotherapeutic approaches [283].

To date, a diverse array of anti‐LAG‐3 monoclonal antibodies has entered clinical development [284]. Relatlimab (BMS‐986016), the first fully human IgG4‐κ monoclonal antibody targeting LAG‐3, exhibits high‐affinity binding to LAG‐3 and effectively blocks its interaction with MHC‐II. It has demonstrated favorable tolerability and promising clinical activity in phase I‐III trials involving patients with solid tumors and hematologic malignancies [285]. Additional investigational agents, including Ieramilimab (LAG525), TSR‐033, and Favezelimab (MK‐4280), are currently under evaluation in clinical studies for advanced solid tumors [286, 287, 288]. Notably, LAG‐3 is often co‐expressed with PD‐1, together driving T cell exhaustion [289]. Ngiow et al. reported that, in a melanoma model, dual blockade of LAG‐3 and PD‐1 can recalibrate the expression balance of CD94/NKG2 receptors on exhausted CD8^+^ T cells, thereby restoring their antitumor immune function [290]. Compared to monotherapy, combined inhibition of the LAG‐3 and PD‐1 pathways demonstrates superior efficacy in enhancing antitumor immunity [291]. Nevertheless, key questions remain regarding the distinct signaling pathways of PD‐1 and LAG‐3. Although the broad repertoire of LAG‐3 ligands suggests multifaceted regulatory roles, the precise mechanisms and functional outcomes of its interactions with individual ligands remain incompletely elucidated. Further research is warranted to refine targeted therapeutic approaches [292].

#### 4.1.5 TIM‐3

TIM‐3 was initially identified as a marker and regulator of T cell exhaustion in the context of HIV‐1 infection. Subsequent studies have consistently demonstrated that TIM‐3 plays a critical role in mediating negative immune regulation across various chronic viral infections, including HBV, HCV, and Friend virus, as well as within the tumor microenvironment [293, 294]. Elevated expression of TIM‐3 on TILs is strongly associated with T cell exhaustion, thereby facilitating tumor immune evasion [295]. Mechanistically, the intracellular domain of TIM‐3 contains conserved tyrosine residues that can be phosphorylated by Src family tyrosine kinases, such as Lck and Fyn. This phosphorylation enables the recruitment of multiple SH2 domain‐containing signaling proteins, including PI3K, PLC‐γ1, and Ras‐GAP, leading to the activation of NFAT/AP‐1 and NF‐κB signaling pathways [296]. While this signaling cascade may transiently enhance T cell responses, sustained activation promotes terminal T cell differentiation into an exhausted phenotype [297]. Clinical evidence underscores the pivotal role of TIM‐3 in T cell dysfunction. For instance, in patients with HBV‐related HCC and prostate cancer, increased TIM‐3 expression on tumor antigen‐specific CD8^+^ T cells correlates positively with disease progression, suggesting its potential utility as a prognostic biomarker [298, 299]. Furthermore, emerging research has highlighted the involvement of TIM‐3 in Treg function. Activated human TIM‐3^+^ FoxP3^+^ Tregs isolated from tumor tissues or expanded in vitro exhibit enhanced immunosuppressive activity compared to their TIM‐3^−^ FoxP3^+^ counterparts and contribute to the exacerbation of effector T cell exhaustion, underscoring the broad inhibitory influence of TIM‐3 across diverse immune cell subsets [300].

The mechanism of TIM‐3 is highly complex, as it exerts diverse inhibitory effects on various immune cells through interactions with multiple ligands. For instance, the binding of TIM‐3 to galectin‐9 (Gal‐9) induces apoptosis in Th1 cells and CTLs [301]. Furthermore, its interaction with Gal‐9 or carcinoembryonic antigen‐related cell adhesion colecule 1 (CEACAM‐1) enhances the suppressive function of Tregs and impairs the effector functions of CD8^+^ T and Th1 cells, thereby contributing to T cell exhaustion within the TME and correlating closely with poor patient prognosis [302, 303]. Consequently, blockade of the TIM‐3 signaling pathway may alleviate its inhibitory effects on multiple immune cell populations, restore anti‐tumor immune responses, and facilitate the development of immunological memory [304].

Currently, a range of immunotherapeutic agents targeting TIM‐3 have advanced into phase I/II clinical trials. Notably, sabatolimab (MBG453), a high‐affinity humanized IgG4 monoclonal antibody, effectively inhibits the interaction of TIM‐3 with Gal‐9 and phosphatidylserine (PtdSer) [305, 306]. This agent demonstrates a distinctive dual “immune‐bone marrow” mechanism of action in acute myeloid leukemia (AML) and myelodysplastic syndromes (MDS), enabling both restoration of immune‐mediated clearance of leukemia stem cells and direct suppression of tumor cell proliferation in TIM‐3‐expressing malignancies [307]. Similarly, the anti‐TIM‐3 antibody M6903 has been shown to concurrently disrupt the binding of Gal‐9, PtdSer, and CEACAM‐1 to TIM‐3, thereby enhancing T cell functionality [308]. Beyond monoclonal antibodies, small molecule inhibitors such as ML‐T7 bind to the FG‐CC loop of TIM‐3, interfering with its interactions with PtdSer and CEACAM‐1; preclinical studies indicate that ML‐T7 significantly improves survival and enhances the antitumor activity of CD8^+^ CTLs [309]. Importantly, TIM‐3 is frequently co‐expressed with PD‐1 on T_EX‐term_ cells, suggesting that combined blockade may yield synergistic antitumor effects. A phase I/II clinical trial (NCT02608268) evaluating sabatolimab as monotherapy or in combination with the anti‐PD‐1 antibody spartalizumab in patients with advanced solid tumors demonstrated limited efficacy with monotherapy, but the combination regimen exhibited preliminary antitumor activity and was well tolerated. In conclusion, TIM‐3 represents a promising immune checkpoint target with significant potential to reverse T cell exhaustion and augment antitumor immunity. Targeted therapies against TIM‐3, particularly when combined with PD‐1 inhibition, may offer a novel therapeutic strategy for patients with diverse cancer types.

### 4.2 Transcription Factors

**FIGURE 5:**
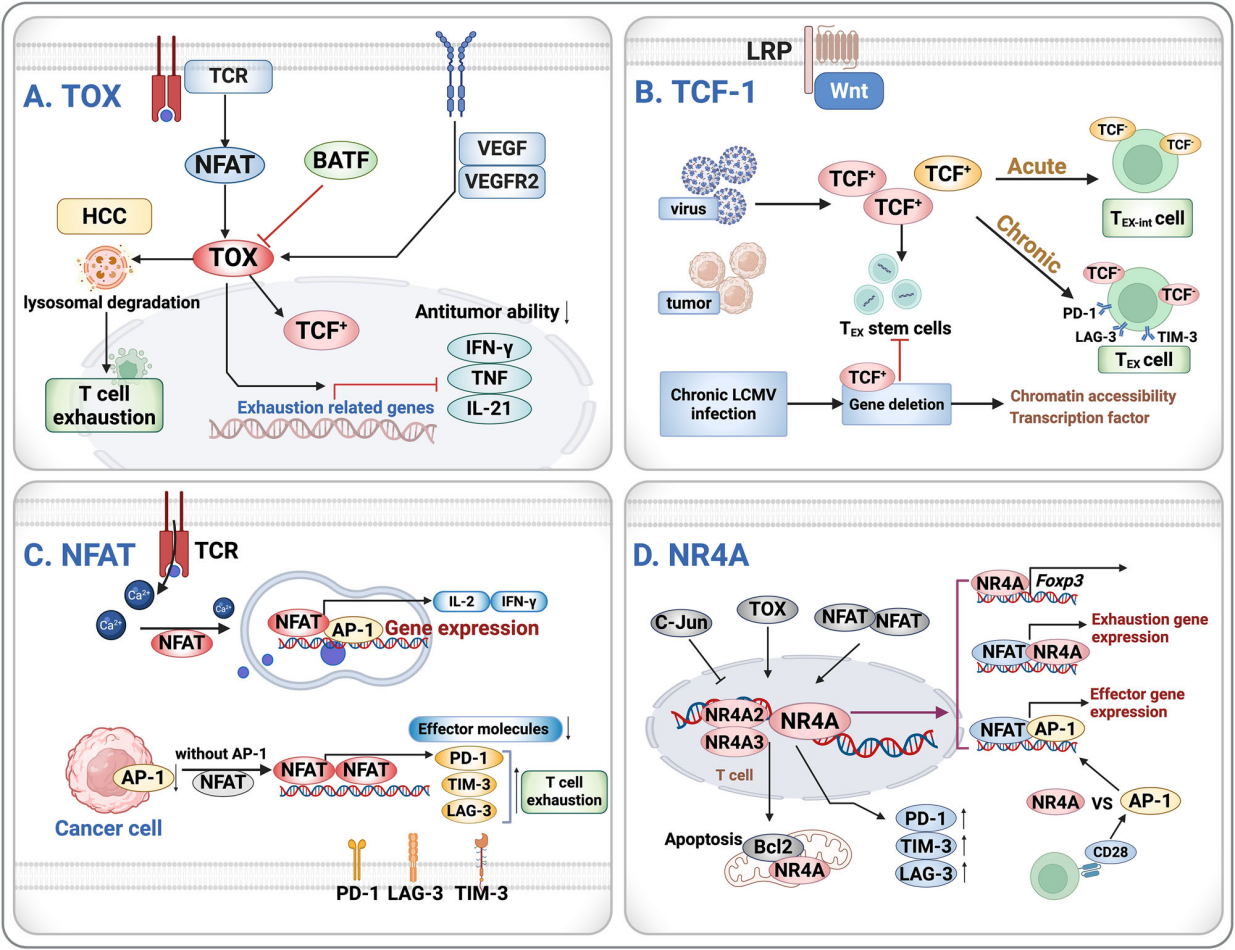
Key transcription factors of T cell exhaustion and their regulatory networks. (A) TOX, a central transcription factor in the initiation and maintenance of T cell exhaustion, is upregulated through the calcineurin‐NFAT signaling pathway. It promotes the expression of inhibitory receptors such as PD‐1 and LAG‐3, and directly suppresses the production of effector‐related molecules, including IFN‐γ, TNF, and IL‐21, thereby further impairing the anti‐tumor functionality of CD8^+^ T cells. (B) TCF‐1 plays a critical role in the development and survival of T_PEX_ cells, preserving their long‐term persistence and self‐renewal capacity. Analogous to its function in memory T cells, TCF‐1 is essential for the sustained presence of T cells during chronic infections and tumor progression. (C) NFAT drives the expression of effector genes upon acute T cell activation, under conditions of chronic stimulation, unopposed NFAT—lacking cooperation with AP‐1—preferentially binds to regulatory regions of inhibitory receptor genes such as PD‐1, TIM‐3, and LAG‐3, thereby promoting T cell exhaustion. NFAT also collaborates with transcription factors, including IRF4 and BATF, to reinforce exhaustion‐associated transcriptional programs. (D) The NR4A family represents another key group of transcription factors implicated in driving T cell exhaustion. Upregulated by NFAT signaling, elevated NR4A expression enhances inhibitory receptor expression, suppresses effector cytokine production, and engages in positive feedback regulation with TOX/TOX2 to stabilize the exhausted phenotype. Consequently, targeting these transcription factors and their upstream signaling pathways—through strategies such as direct inhibition (e.g., TOX), modulation of expression levels (e.g., TCF‐1), or disruption of cooperative interactions (e.g., restoring the NFAT/AP‐1 balance)—holds significant therapeutic potential for reversing T cell dysfunction and reconstituting anti‐tumor immune responses. Abbreviations: TOX, thymocyte selection–associated high mobility group box protein; PD‐1, programmed death protein 1; LAG‐3, lymphocyte‐activation gene 3; IFN‐γ, interferon‐γ; TNF, tumor necrosis factor; IL‐21, Interleukin‐21; HCC, hepatocellular carcinoma; AP‐1, activator protein‐1; TIM‐3, T cell immunoglobulin domain and mucin domain‐3; IRF4, interferon regulatory factor 4; BATF, basic leucine zipper ATF‐like transcription factor; NFAT, nuclear factor of activated T cell; NR4A, nuclear receptor 4A; TCF‐1, T cell factor 1; LCMV, lymphocytic choriomeningitis virus; TCR, T cell receptor; T_PEX_ cell, precursor exhausted T cell; T_EX‐int_ cell, intermediate exhausted T cell.

The fate of T_EX_ cells is governed by a core hierarchical network of transcription factors that establishes and maintains a dysfunctional state through the remodeling of the epigenetic landscape (Figure 5).

#### 4.2.1 TOX

As an intranuclear DNA‐binding protein, TOX plays a pivotal role in the epigenetic regulation of T cell exhaustion [52]. Under conditions of persistent antigen stimulation, the calcineurin‐NFAT signaling pathway becomes aberrantly activated, leading to sustained expression of both TOX and its homolog TOX2 [310]. Notably, in contrast to conventional T cell activation—which relies on the NFAT‐AP‐1 transcriptional complex—NFAT can independently initiate the TOX transcriptional program in the absence of AP‐1 within the tumor microenvironment [10]. Furthermore, TOX stabilizes the epigenetic and transcriptional landscape of T_EX_ cells by preserving the expression of key lineage‐defining factors such as the progenitor marker TCF‐1 and the survival regulator Eomes, while also establishing a calcineurin‐independent autoregulatory feedback loop [8]. Importantly, a robust positive feedback mechanism exists between TOX and co‐inhibitory receptors, which exacerbates T cell dysfunction. On one hand, TOX directly upregulates the transcription of inhibitory receptors, including PD‐1 and LAG‐3 [10]. On the other hand, in models of HCC, TOX may enhance PD‐1 protein stability by interfering with its lysosomal degradation pathway [311]. Intriguingly, different immune checkpoint molecules exert distinct regulatory effects on TOX expression. Depletion of LAG‐3 results in a significantly greater reduction in TOX levels compared to PD‐1 depletion, indicating a dominant role for LAG‐3 in sustaining TOX expression [312]. This differential regulatory pattern has been corroborated in models of chronic viral infection, where LAG‐3 primarily supports TOX expression, whereas PD‐1 predominantly modulates the proliferation of T_EX_ cells [24]. Moreover, TOX directly suppresses the expression of effector cytokines such as IFN‐γ, TNF, and IL‐21, thereby further impairing the anti‐tumor functionality of CD8^+^ T cells [312].

Targeting TOX has emerged as a promising strategy to reverse T cell dysfunction and enhance antitumor immune responses. Experimental evidence demonstrates that knockout of TOX in established T_EX_ cells significantly reduces apoptosis resistance, downregulates the expression of inhibitory receptors such as PD‐1 and TIM‐3, and promotes a shift toward an effector‐like phenotype [70]. These findings provide a robust theoretical foundation for the development of TOX‐targeted inhibitors. Furthermore, several studies indicate that modulating upstream signaling pathways of TOX may hold therapeutic potential; for instance, overexpression of basic leucine zipper ATF‐like transcription factor (BATF) or blockade of vascular endothelial growth factor (VEGF)/VEGFR2 signaling can suppress TOX expression and restore T cell functionality [313, 314]. Notably, therapeutic approaches targeting TOX face significant challenges. While TOX ablation can drive reprogramming of T_EX_ cells into effector‐like states, its intrinsically disordered protein structure poses substantial obstacles to the design of direct small‐molecule inhibitors [315]. More critically, TOX plays a protective role in preventing T cell overactivation and activation‐induced cell death, highlighting its dual biological functions, which must be carefully considered in therapeutic contexts [316]. Future research should prioritize targeting TOX upstream regulatory networks or exploring synergistic combinations with existing immunotherapeutic modalities.

#### 4.2.2 TCF‐1

TCF‐1 (encoded by Tcf7), a key effector molecule of the Wnt/β‐catenin signaling pathway, plays an essential role in maintaining T_PEX_ [9, 317]. Unlike conventional effector or memory T cells, TCF‐1^+^ T_PEX_ cells retain self‐renewal capacity and proliferative potential in chronically antigen‐stimulated environments, while avoiding terminal differentiation through epigenetic reprogramming [318, 319, 320]. Specifically, TCF‐1 regulates T_PEX_ cell fate decisions via multi‐level mechanisms. At the epigenetic level, TCF‐1 significantly influences global chromatin accessibility and the transcriptome in Tex by upregulating a set of transcriptional regulators associated with critical effector functions—such as FOXO1, ZEB2, ID3, and Eomes—and by suppressing the expression of Blimp‐1 and Bim [321]. In contrast, TCF‐1 inactivation is partially mediated by DNA methylation, as demonstrated in a rapid in vitro CD8^+^ T cell exhaustion system [76]. Genetic deletion of Tcf7 leads to a marked reduction in the frequency of T_PEX_ cells and an increased viral load in a chronic LCMV infection model, underscoring its pivotal role in sustaining antiviral immune responses [322]. Collectively, these findings highlight TCF‐1 as a promising therapeutic target for enhancing both antiviral and antitumor immunity.

Clinical transformation studies have demonstrated that the expression level of TCF‐1 in tumor‐infiltrating CD8^+^ T cells is closely associated with the therapeutic efficacy of PD‐1 blockade. In melanoma patients, pretreatment intratumoral enrichment of TCF‐1^+^ CD8^+^ T cells has been significantly correlated with improved treatment response and prolonged survival, whereas the accumulation of CD39^+^ Tim‐3^+^ T_EX‐term_ cells is indicative of treatment resistance [323]. These findings establish TCF‐1 as a promising biomarker for predicting immunotherapy outcomes. Moreover, TCF‐1^+^ T_PEX_ cells have emerged as a novel therapeutic target for enhancing the durability of antitumor immunity. In vitro studies indicate that the expansion and differentiation of stem cell‐like TCF‐1^+^ memory T cells can be promoted through the combined application of antioxidants, TCR stimulation, and pro‐inflammatory cytokines, offering a potential strategy to augment antitumor immune responses [317]. Notably, memory T cells in human lymph nodes exhibit strong phenotypic and functional similarities to TCF‐1^+^ CD8^+^ T cells observed in mouse models following checkpoint blockade, including high TCF‐1 expression, robust proliferative capacity, and self‐renewal potential [323]. This suggests their critical role as a therapeutic target in chronic infections and cancer. TCF‐1 plays a pivotal role in modulating T cell exhaustion during chronic infections and malignancies, as well as in sustaining immune responses under PD‐1 blockade. Consequently, targeting TCF‐1—either directly or through its upstream regulators or downstream effectors—alone or in combination with other therapeutic approaches, may enhance and prolong antitumor and anti‐infective immunity. Nevertheless, the precise regulatory mechanisms governing TCF‐1 expression in CD8^+^ T cells, along with its dynamic interactions with other transcription factors, require further investigation to identify more effective therapeutic interventions.

#### 4.2.3 NFAT

The NFAT family, serving as central effectors in the calcium signaling pathway, exhibits a distinctive duality in regulating T cell function [324, 325]. Under physiological conditions, TCR‐mediated calcium influx activates calcineurin, leading to NFAT dephosphorylation and subsequent nuclear translocation. Once in the nucleus, NFAT forms a transcriptional complex with AP‐1 to cooperatively drive the expression of effector cytokines such as IL‐2 and IFN‐γ [326]. However, under persistent antigen stimulation within the tumor microenvironment, the absence of costimulatory signals results in inadequate AP‐1 expression [69]. This condition promotes NFAT to bind to regulatory regions of exhaustion‐associated genes in an atypical monomeric form. This “cofactor‐deficient” mode of NFAT activation selectively enhances the transcriptional upregulation of immune checkpoint molecules, including PD‐1, TIM‐3, and LAG‐3, while simultaneously suppressing the expression of memory‐related genes [69]. Mechanistic investigations have revealed that NFAT family members exhibit both functional redundancy and distinct roles in the T cell exhaustion program. Combined deletion of NFAT1 and NFAT2 markedly reduces the expression levels of inhibitory receptors, whereas additional knockdown of NFAT4 nearly abolishes residual expression, indicating a cumulative contribution of individual NFAT members to the exhaustion network [69]. Furthermore, NFAT can cooperate with transcription factors such as IRF4 and BATF to reinforce the exhaustion‐associated epigenetic program by establishing specific patterns of chromatin accessibility [327]. Notably, the role of NFAT5, a member of the NFAT family, has garnered increasing attention in recent years with respect to T cell exhaustion. Unlike other NFAT family members, NFAT5 operates independently of calcineurin and lacks an AP‐1 binding site. Instead, NFAT5 is primarily activated by TCR signaling and metabolic stresses such as hyperosmolality within the tumor microenvironment. Importantly, NFAT5 exerts minimal influence on T cell exhaustion associated with chronic infections, and its pro‐exhaustion effects are largely restricted to the tumor context. This indicates that NFAT5 plays a context‐specific regulatory role across different pathological conditions [328].

Based on these findings, the therapeutic strategy targeting NFAT is evolving from conventional calcineurin inhibition toward more precise and selective interventions. Emerging research directions include the development of small‐molecule compounds designed to specifically disrupt the binding of unchaperoned NFAT to target genes, as well as the restoration of normal NFAT/AP‐1 transcription complex function through epigenetic modulation. These approaches aim to selectively reverse T cell exhaustion while preserving essential immune functions, thereby offering novel insights for enhancing immunotherapy efficacy [329].

#### 4.2.4 NR4A

The NR4A orphan nuclear receptor family, comprising NR4A1, NR4A2, and NR4A3, is markedly upregulated in T_EX‐term_ cells. Its expression is directly induced by persistent calcium‐NFAT signaling and further amplified in the absence of AP‐1 activity [330]. Similar to TOX, NR4A contributes to the establishment and maintenance of the T cell exhaustion phenotype through epigenetic remodeling [11, 331]. Specifically, NR4A mediates the formation of repressive chromatin states by recruiting histone‐modifying complexes to the promoter regions of effector genes, while simultaneously increasing chromatin accessibility at exhaustion‐associated gene loci. Notably, NR4A and the HMG‐box transcription factors TOX/TOX2 engage in a positive regulatory feedback loop, mutually enhancing each other's expression to establish a stable transcriptional network. This self‐reinforcing mechanism is especially pronounced in T_EX‐term_ cells and may contribute to the limited efficacy of conventional ICIs in reversing this state. In preclinical CAR‐T cell models, genetic ablation of NR4A not only substantially reduces the expression of inhibitory receptors such as PD‐1 and TIM‐3 but also enhances cytokine production and in vivo persistence, underscoring its pivotal role in driving T cell dysfunction [10].

Given the pivotal role of NR4A in T cell exhaustion, therapeutic strategies targeting the NR4A family hold considerable promise. Current research efforts are focused on developing small‐molecule inhibitors to suppress NR4A transcriptional activity and employing epigenetic approaches to disrupt its interactions with downstream effector molecules. However, given NR4A's essential role in T cell homeostasis, these physiological functions must be carefully considered [332]. Future studies should aim to elucidate strategies that achieve a precise balance between reversing T cell exhaustion and preserving immune homeostasis.

#### 4.2.5 Other Key Transcription Factors

In addition to TOX, TCF‐1, NFAT, and NR4A, recent studies have revealed that a variety of key transcription factors also play critical roles in the initiation and maintenance of T cell exhaustion. Given the high complexity of their mechanisms and functions, and due to space limitations, this review provides only a brief overview.

Interferon regulatory factor 4 (IRF4) and BATF are key transcription factors involved in TCR signaling. Their expression is significantly elevated in chronic infectious and neoplastic conditions. IRF4 promotes the expression of the inhibitory receptor PD‐1 and suppresses TCF‐1, thereby contributing to metabolic dysregulation and functional exhaustion of T_EX_ cells. Reduction of IRF4 expression has been shown to partially restore T cell function and facilitate the development of memory‐like T cells, highlighting its central role in the transcriptional program of T cell exhaustion [100, 327]. BATF acts in concert with IRF4 to modulate the phenotype and functional state of T_EX_ cells. Upregulation of BATF is closely associated with the downregulation of AP‐1 family transcription factors. Notably, BATF overexpression can reverse the T_EX_ cell phenotype and enhance antitumor immunity, whereas BATF depletion favors the generation of memory T cells, indicating a bidirectional regulatory mechanism between T cell exhaustion and effector function [314].

As a transcriptional repressor, Blimp‐1 (PRDM1) promotes the upregulation of inhibitory receptors and suppresses memory T cell differentiation during chronic infection, with its expression level playing a critical role in regulating the balance between effector function and exhaustion in T_EX_ cells [333]. Deficiency of Blimp‐1 reduces the expression of inhibitory receptors such as PD‐1 and can partially restore T cell effector function; however, it may also compromise the persistence of antiviral and antitumor immune responses [334].

As classical regulators of T cell differentiation, EOMES and T‐bet also play critical roles in the differentiation of T_EX_ cell subsets. The T_EX_ cell subset characterized by high T‐bet expression exhibits enhanced proliferative capacity and responsiveness, whereas elevated EOMES expression is associated with a terminally exhausted phenotype. The dynamic balance between these two transcription factors determines the differentiation trajectory and functional state of T_EX_ cells [122, 335].

In addition, members of the IKZF family—specifically Ikaros (IKZF1), Helios (IKZF2), and Aiolos (IKZF3)—have recently been identified as key regulators of T_EX_ cell differentiation and functional maintenance. Dual knockdown of IKZF1 and IKZF3 significantly enhances antitumor and antiviral effector functions in T_EX_ cells, highlighting their critical role in modulating the expansion and functional capacity of the T_EX_ cell subpopulation [336]. Concurrently, the NRF2 signaling pathway influences T_EX_ cells metabolism and differentiation by regulating the oxidative stress response and downstream PTGIR receptor expression, revealing a novel mechanism underlying the metabolic‐transcriptional regulatory axis in T_EX_ cells development [337, 338].

The transcriptional regulatory network governing T_EX_ cells is highly complex, with the coordinated interplay—including both synergistic and antagonistic interactions—of multiple transcription factors collectively determining cell fate and functional states. A comprehensive understanding of this regulatory machinery provides a crucial theoretical foundation for optimizing immunotherapeutic strategies.

### 4.3 Metabolic Markers

The functional state of T cells is closely linked to their metabolic health, with specific metabolic enzymes and their products now serving as key indicators for defining the exhaustion state.

#### 4.3.1 CD39/CD73

The purinergic signaling pathway, comprising CD39 (ENTPD1) and its downstream effector CD73, plays a pivotal role in the metabolic regulation of T cell exhaustion. This pathway is highly expressed on terminally differentiated exhausted CD8^+^ T cells and mediates the sequential hydrolysis of extracellular ATP and ADP to AMP, which is subsequently converted by CD73 into adenosine—a potent immunosuppressive molecule. This process establishes an adenosine‐rich immunosuppressive network within the tumor microenvironment [339, 340]. Functionally, CD39^hi^ CD8^+^ T cells exhibit a characteristic exhausted phenotype, marked by significantly reduced secretion of effector cytokines such as IFN‐γ and TNF‐α, along with co‐upregulation of inhibitory receptors PD‐1 and TIM‐3 [340]. This metabolic‐immune crosstalk not only directly compromises T cell antitumor activity but also leads to sustained activation of the cAMP‐PKA signaling cascade via the adenosine A2a receptor (A2aR) axis, thereby suppressing TCR signaling and impairing cell cycle progression [341]. Notably, recent studies indicate that adenosine‐mediated immunosuppression can also involve additional pathways, such as inhibition of the mTORC1 pathway and modulation of MAP kinase signaling, further dampening T cell metabolic fitness and effector function [342, 343]. Moreover, CD39/CD73^+^ T cells often co‐express immune checkpoints like PD‐1, and adenosine signaling may synergize with these pathways to enhance immunosuppression [344, 345]. Hypoxia within the TME enhances CD39 expression through an HIF‐1α‐dependent mechanism while concurrently inducing mitochondrial metabolic dysfunction, thus creating a self‐reinforcing cycle of immunometabolic suppression [346]. Preclinical evidence indicates that targeting the CD39/CD73‐adenosine axis can effectively reverse T cell exhaustion and synergize with ICB therapies [347]. In particular, when combined with chemotherapeutic agents such as oxaliplatin, this strategy demonstrates robust potential to remodel TIME, offering a promising metabolic intervention to overcome resistance to cancer immunotherapy [348].

#### 4.3.2 IDO1

Indoleamine 2,3‐dioxygenase 1 (IDO1), a key rate‐limiting enzyme in the tryptophan‐kynurenine metabolic pathway, plays a critical role in establishing an immunosuppressive microenvironment [349]. It promotes T cell exhaustion through two synergistic mechanisms: first, by depleting tryptophan—an essential amino acid—within the microenvironment, IDO1 induces amino acid starvation stress, activates the general control nonderepressible 2 (GCN2) kinase signaling pathway, and consequently leads to T cell proliferation arrest and functional impairment [350]. Second, its catalytic product, kynurenine, directly modulates exhaustion‐associated transcriptional programs by activating the AhR signaling axis, thereby promoting Treg differentiation and recruitment of MDSCs [351, 352]. Mechanistically, IDO1‐mediated metabolic reprogramming is closely intertwined with epigenetic regulation. AhR not only upregulates the expression of inhibitory receptors such as PD‐1 and TIM‐3 but also alters chromatin accessibility in T cells via histone modifications, thus stabilizing the exhausted phenotype [353, 354]. Notably, studies in glioblastoma models have shown that while IDO1 knockdown transiently restores T cell function, it simultaneously triggers compensatory upregulation of IDO1 in other immune cells, underscoring the complexity of this regulatory network [355].

Based on current mechanistic insights, the combination of IDO1 inhibitors with radiotherapy and ICIs holds significant therapeutic potential [22]. Clinical studies in renal cell carcinoma have demonstrated synergistic antitumor effects with the combination of pembrolizumab and epacadostat [356]. However, the variability observed across clinical trials indicates that the efficacy of IDO1 inhibitors may be influenced by multiple factors, including tumor type, microbiome composition, and host metabolic status. Therefore, future efforts should focus on developing more precise biomarker‐guided strategies for patient stratification [357, 358, 359, 360].

#### 4.3.3 Other Metabolic Markers

In the study of T cell exhaustion, beyond the classical CD39/CD73 and IDO1 pathways, accumulating experimental evidence in recent years has identified additional key metabolic markers and elucidated their underlying mechanisms. Dysregulation of lactate metabolism represents a critical characteristic of T cell exhaustion within the tumor microenvironment. T_EX‐term_ cells specifically exhibit high expression of MCT11, which facilitates lactate uptake, resulting in intracellular acidification and metabolic suppression, thereby exacerbating T cell exhaustion. Conditional deletion of MCT11 or its blockade via antibodies has been shown to significantly reduce lactate influx, restore T cell effector function, and suppress tumor growth in preclinical tumor models, highlighting MCT11 as a specific metabolic marker and a promising therapeutic target for T_EX_ cells [103]. Furthermore, dysregulated cholesterol metabolism has been firmly linked to T cell exhaustion. Cholesterol accumulation in tumor tissues enhances the expression of immune checkpoint molecules—including PD‐1, 2B4, TIM‐3, and LAG‐3—on CD8^+^ T cells, promoting an exhausted phenotype. Mechanistically, cholesterol induces transcription of immune checkpoint genes by augmenting endoplasmic reticulum stress and activating the XBP1 signaling pathway. Reducing cholesterol levels in T cells or inhibiting XBP1 signaling effectively restores their antitumor functionality [361].

Reprogramming of methionine metabolism represents another critical mechanism underlying tumor‐induced T cell exhaustion. Tumor cells directly promote T cell exhaustion by enhancing the methionine cycle and accumulating high levels of SAM and MTA. CRISPR‐Cas9‐mediated knockout of MAT2A, the enzyme responsible for SAM synthesis, significantly attenuates T cell exhaustion and delays tumor progression, indicating that molecules involved in methionine metabolism serve as novel metabolic markers of T cell exhaustion [20]. Moreover, levels of L‐2‐hydroxyglutarate (L‐2‐HG), an immunomodulatory metabolite, are markedly reduced in T_EX_ cells. Exogenous supplementation of L‐2‐HG has been shown to improve mitochondrial function, reverse epigenetic repression, promote memory T cell differentiation, and enhance antitumor immunity, highlighting its regulatory role in T cell exhaustion and its potential as a metabolic intervention target [90].

Mitochondrial dysfunction and oxidative stress represent additional key metabolic features of T cell exhaustion. T_EX_ cells exhibit loss of mitochondrial membrane potential, impaired respiratory chain activity, and accumulation of ROS, leading to insufficient energy production and cellular dysfunction [95, 362]. Downregulation of regulators of mitochondrial biogenesis, such as PGC‐1α, is closely associated with the exhausted phenotype, and supplementation with NAD^+^ precursors or enhancement of mitochondrial function has been shown to partially restore T cell functionality [101]. Furthermore, dysregulated arginine metabolism leads to upregulation of ARG1, resulting in arginine depletion within T cells, inhibition of the mTOR‐T‐bet signaling axis, and promotion of the exhausted phenotype. Modulation of arginine metabolism can improve the metabolic fitness of T cells and enhance their antitumor efficacy [363].

Notably, the functional roles of certain metabolic markers remain controversial. For instance, fatty acid metabolism exhibits a dual role in T cell exhaustion: it can support T cell survival by providing energy through β‐oxidation, yet excessive accumulation may lead to lipid peroxidation and cellular dysfunction. Specifically, molecules such as fatty acid binding protein 5 (FABP5) promote fatty acid uptake and oxidation, enabling T cells to adapt to the glucose‐deprived tumor microenvironment. However, sustained or excessive fatty acid uptake and oxidation can result in lipid peroxidation, mitochondrial damage, and impaired T cell function [364]. Moreover, the interplay between metabolic reprogramming and epigenetic regulation or signaling pathways has not been fully elucidated. Future studies should aim to comprehensively characterize the dynamic changes and underlying molecular mechanisms of metabolic markers during the induction, maintenance, and reversal of T cell exhaustion, thereby informing more precise immunometabolic intervention strategies.

## 5 Immunotherapy Strategies Targeting T Cell Exhaustion

The advent of cancer immunotherapy, particularly ICB and ACT, has profoundly transformed the treatment landscape for numerous advanced malignancies by restoring the antitumor activity of CTLs [365]. Nevertheless, clinical efficacy varies significantly across patient populations, and long‐term response rates remain suboptimal. T cell exhaustion is widely recognized as a central biological barrier contributing to this therapeutic challenge [366]. Within the TME, persistent antigen exposure, the synergistic effects of multiple immunosuppressive signals, and metabolic stress collectively drive T cells toward progressive functional impairment and epigenetic reprogramming, ultimately leading to the differentiation of T_EX‐term_ cells, which have severely diminished effector capacity [161]. This dysfunctional state not only compromises the cytolytic potential of T cells but also confers resistance to both ICB and ACT, thereby representing a key mechanism underlying immunotherapy failure and disease relapse [3, 367].

**FIGURE 6:**
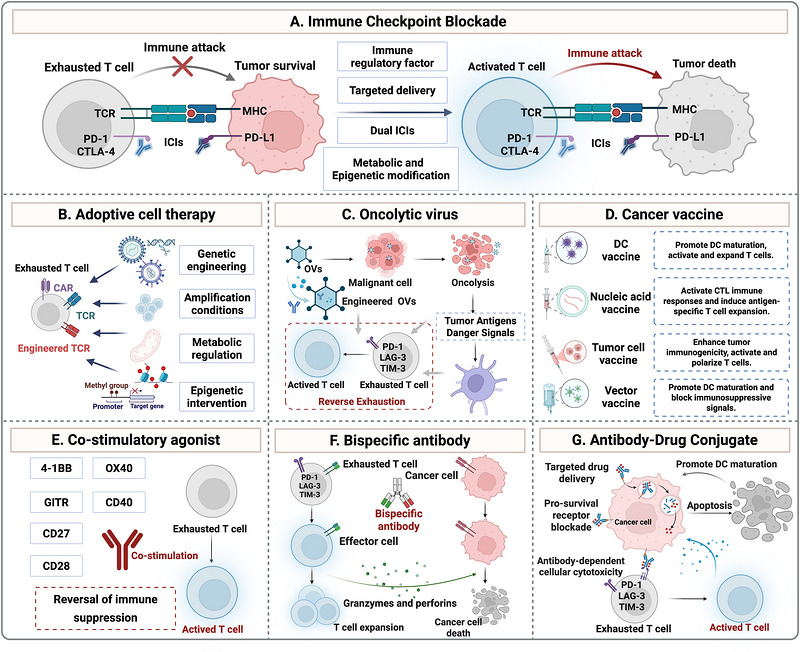
Combination immunotherapy strategies targeting T cell exhaustion. A.Immune checkpoint blockade therapy: Utilizing immunomodulatory agents, targeted delivery systems, dual ICIs, and interventions involving metabolic and epigenetic modifications, this approach alleviates tumor‐mediated suppression of T cells and reactivates anti‐tumor immune responses. B. ACT: This strategy enhances the anti‐tumor capacity of T_EX_ cells through genetic engineering, optimized ex vivo expansion protocols, and metabolic or epigenetic modulation. C. OVs: Oncolytic viruses selectively infect and lyse tumor cells, leading to the release of tumor‐associated antigens and danger signals, which promote DCs activation, reverse T cell exhaustion, and stimulate broad anti‐tumor immunity.D.Cancer vaccines: These include DC vaccines, nucleic acid‐based vaccines, whole tumor cell vaccines, and viral vector vaccines, all designed to enhance tumor immunogenicity, promote DC maturation, and elicit robust CTL responses. E. Costimulatory agonists: Agonistic molecules targeting receptors such as 4‐1BB, OX40, GITR, CD40, CD27, and CD28 can counteract immunosuppressive microenvironments and restore effector functions in T_EX_ cells. F. BsAbs: These engineered antibodies simultaneously bind to T_EX_ cells and tumor cells, facilitating immune synapse formation, promoting granzyme and perforin release from effector cells, inducing tumor cell apoptosis, and supporting T cell proliferation. G. ADCs: By enabling targeted delivery of cytotoxic payloads, blocking pro‐survival signaling in cancer cells, promoting DC maturation, and mediating ADCC, ADCs contribute to tumor cell elimination and may partially reverse T cell exhaustion. Abbreviations: ACT, adoptive cell therapy; CAR, chimeric antigen receptor; CTL, cytotoxic T lymphocyte; DC, dendritic cell; ICI, immune checkpoint inhibitor; OV, oncolytic virus; TCR, T cell receptor; BsAbs, bispecific antibodies; ADC, antibody–drug conjugate; ADCC, antibody‐dependent cellular cytotoxicity.

Given the pivotal role of T cell exhaustion in promoting immune evasion, the development of therapeutic approaches capable of precisely modulating or reversing this state has become a critical priority in overcoming current limitations in cancer immunotherapy. The research paradigm is shifting from monotherapy‐based ICB toward mechanism‐driven, multidimensional combinatorial strategies [3, 368, 369]. As previously outlined, these emerging strategies aim to enhance the efficacy of existing ICB and ACT regimens by targeting co‐inhibitory receptor networks on T cell exhaustion (e.g., PD‐1, LAG‐3, TIGIT), reprogramming intrinsic transcriptional and epigenetic regulatory circuits (e.g., TOX and TCF‐1), or remodeling the metabolic environment of the TME. This section provides a systematic and critical evaluation of the most recent therapeutic approaches directed at T cell exhaustion, with emphasis on their mechanisms of action, potential for synergistic integration, and translational prospects, aiming to establish a conceptual framework and strategic guidance for the design of next‐generation personalized combination immunotherapies (Figure 6 and Table 2).

**TABLE 2:** Clinical trials for improving immunotherapy through targeting T cell exhaustion.

Tumor type	Specific exhaustion characteristics	Therapeutic strategy	Drug or measure	Mechanism	Phase/Status and enrolment	NCT number	Key ending
Lung cancer	High expression of inhibitory receptors, reduced effector function, and limited proliferative persistence.	By enhancing the intrinsic anti‐depletion ability of TILs through gene editing, combined with immune checkpoint blockade and cytokine support, the activity, proliferation ability and persistence of T cells in NSCLC can be reconstructed	TIL+ICB (Nivolumab)	PD‐1 blockade with TIL + IL‐2 boosts expansion	Phase 1 Completed	NCT03215810	ORR 23% with tumor reduction in 11 patients; two durable CR.
			OBX‐115+TIL	mbIL‐15–engineered TILs enhance persistence and resist T‐cell exhaustion	Phase 1/2 Recruiting	NCT06060613	Among 11 evaluable patients, the ORR was 36% (1 CR, 3 PR), with five SD ≥12 weeks, yielding a DCR of 82%; 73% showed tumor reduction. In the RP2D cohort (n = 6), the ORR was 67% (1 CR, 3 PR), and the remaining two achieved SD ≥12 weeks, resulting in a DCR of 100%, with tumor shrinkage in 83% of patients.
			CISH KO+TIL	CISH KO boosts TCR activity and cytokine function, improving TIL resistance to exhaustion	Phase 1/2 Not yet recruiting	NCT05566223	/
			IOV‐4001 (PD‐1 KO) +TIL	PD‐1 KO removes inhibitory signaling, reducing exhaustion	Phase 1/2 Recruiting	NCT05361174	/
			L‐TIL+DTX+ ICB (Tislelizumab)	L‐TIL combined with PD‐1 blockade to re‐activate exhausted T cells	Phase 2 Recruiting	NCT05878028	/
			IL‐2+ICB (Pembrolizumab)	IL‐2–mediated stimulation of effector‐like T‐cell responses	Phase 1 Recruiting	NCT05493566	/
Breast cancer	Reduced tumor‐specific T‐cell activity with increased inhibitory signaling	vaccine+ICB	tumor membrane vesicle vaccine+ICB (Pembrolizumab/Ipilimumab)	TMV vaccine boosts tumor‐specific T‐cell priming	Phase 1 Not yet recruiting	NCT06324240	/
Glioblastoma	Severely impaired T‐cell function with deep exhaustion and low infiltration	DC+ICB	ppDC+ICB (Nivolumab/Ipilimumab)	DC‐vaccine–primed T cells plus dual ICB reverse exhaustion	Phase 1 Withdrawn	NCT05457959	/
Colorectal cancer	MSI‐H tumors are ICB‐responsive; MSS tumors are resistant.	Cytokine+ICB	IBI363+ICB	PD‐1/PD‐L1 blockade + IL‐2–driven expansion of effector/T_PEX_ subsets	Observational Recruiting	NCT06946745	/
Hematologic malignancies	Limited durability, weakened effector activity, and a tendency toward exhaustion under chronic antigen exposure and insufficient immune reconstitution.	Integrating CAR‐T enhancement, TCR‐engineered immunity, cytokine support, and donor‐derived immune reconstitution to overcome exhaustion and improve persistence of antitumor T cells in hematologic malignancies	Donor Lymphocyte Infusion+ICB (Daratumumab)	DLI plus daratumumab to restore anti‐leukemia T‐cell activity after exhaustion	Phase 1/2 Terminated	NCT03537599	/
			NT‐I7+CAR‐T	NT‐I7 enhances CAR‐T expansion and persistence, preventing exhaustion	Phase 1 Not yet recruiting	NCT07052305	/
			CARAMBA‐1 (SLAMF7‐CAR)+CAR‐T	Engineered activation boosts anti‐myeloma killing while preventing exhaustion	Phase 1/2 Active, not recruiting	NCT04499339	/
			WT1‐TCRc4–engineered CD8^+^ T_CM_/T_N_ cells + TCR‐T	WT1‐TCR–engineered CD8^+^ T_CM_/T_N_ cells limit exhaustion	Phase 1/2 Terminated	NCT02770820	/
			TCRαβ^+^ and CD45RA^+^ cell‐depleted haploidentical HSCT+CAR‐T	Donor‐derived NK/γδ T cell GvL activity plus durable memory immunity to compensate for CAR T exhaustion	Not Applicable Not yet recruiting	NCT07087847	/
Hepatocellular carcinoma	Immunosuppressive liver environment	CAR‐T engineering transformation	RCAR01 (GPC3‐CAR)+CAR‐T	GPC3‐targeted suppression of TGF‐β prevents T cell exhaustion	Phase 1 Not yet recruiting	NCT06968195	/
Head and neck squamous cell carcinoma	Pronounced PD‐1–mediated inhibition, reduced tumor‐specific effector function, and limited responsiveness to antigen restimulation.	By combining PD‐1 blockade with TIL or TCR‐engineered T cells, the anti‐tumor effector function is restored, and T cell exhaustion is reversed	Pembrolizumab+TIL (ITIL‐168)	ITIL‐168 enriches tumor‐reactive TILs	Phase 1 Withdrawn	NCT05393635	/
			PD1inhibitor+TCR‐T	HPV‐E6–specific TCR‐T cells secrete a local PD‐1 antagonist	Phase 1 Unknown status	NCT03578406	/
			PD1inhibitor+TCR‐T	Targeting EBV antigens with an autocrine PD‐1 antagonist to reverse T cell exhaustion	Phase 1/2 Recruiting	NCT04139057	Among the six patients, two achieved PR, including one with a response lasting 9 months; the remaining three patients achieved SD, resulting in a DCR of 100%.
Melanoma	Characterized by high inhibitory signaling, weakened cytokine responsiveness, and insufficient intrinsic persistence.	Enhancing T‐cell antitumor function through next‐generation checkpoint blockade, cytokine modulators, and engineered CAR/TCR‐based cellular therapies to overcome exhaustion and improve persistence	NKG2D‐CAR+CAR‐T	NKG2D‐CAR memory T cells enhance tumor recognition and prevent exhaustion	Phase 1 Recruiting	NCT06087341	/
			Avadomide (CC‐122)+ICB (Nivolumab)	Prevents exhaustion via cereblon‐mediated IKZF1/IKZF3 degradation.	Phase 2 Completed	NCT03834623	/
			Ipilimumab/Nivolumab/ Cabozantinib+ICB	PD‐1 blockade to reinvigorate exhausted T cells	Phase 2 Terminated	NCT05200143	/
			Relatlimab (LAG‐3inhibitor)+ICB (Nivolumab)	Enhances T cell effector function through dual ICI.	Phase 2 Recruiting	NCT05418972	/
Pancreatic Ductal Adenocarcinoma	Extremely “cold”, dense stroma, poor T cell infiltration	TLR agonist+ICB	Rintatolimod (TLR‐3 agonist)+ICB (Durvalumab)	Enhance DC maturation and CD8^+^ T‐cell cross‐priming while reducing Treg recruitment	Phase 1/2 Recruiting	NCT05927142	/
Pan‐cancer	/	/	GSK4381562+ICB	Remove the inhibitory signal mediated by PVRIG	Phase 1 Recruiting	NCT05277051	/
			TP53‐EphA‐2‐CAR‐DC Vaccine+ICB	Activate the TIDCs function to reverse the immunosuppressive TME	Phase 1 Recruiting	NCT05631886	/
			KRAS‐EphA‐2‐CAR‐ DC vaccine+ICB	Activate the TIDCs function to reverse the immunosuppressive TME	Phase 1 Recruiting	NCT05631899	/
			TIL [LN‐144/LN‐145 (lifileucel)]+ICB	Autologous TIL + IL‐2 reinvigorate T cells	Phase 2 Recruiting	NCT03645928	Among 22 treated patients, the EGFR‐wt subgroup showed an ORR of 64.3%, with 6 responses among 11 EGFR‐wt/PD‐L1–negative cases and 5 durable responses overall. In the post–EGFR TKI subgroup (n = 8), the ORR was 12.5%
			MAQ‐001+ICB (Ipilimumab)	Restores T‐cell function by modulating inhibitory signaling to reverse exhaustion	Phase 1 Recruiting	NCT06514651	/
			IL2/mutant p53 peptide pulsed DC vaccine/RAS peptide cancer vaccine+TIL	Boost T‐cell activation and expansion, limiting exhaustion	Phase 2 Completed	NCT00019084	/
			Pembrolizumab+TIL (TBio‐4101)	TBio‐4101 enriches neoantigen‐specific tumor‐reactive TILs and reverses exhaustion.	Phase 1 Terminated	NCT05576077	Terminated due to high manufacturing costs and insufficient funding.

Abbreviations: CAR, chimeric antigen receptor; CR, complete response; DC, dendritic cell; DCR, disease control rate; DLI, donor lymphocyte infusion; EBV, epstein–barr virus; HPV, human papillomavirus; ICB, immune checkpoint blockade; ICI, immune checkpoint inhibitor; MSS, microsatellite stable; MSI‐H, microsatellite instability–high; NK, natural killer; NSCLC, non–small cell lung cancer; ORR, objective response rate; PD‐1, programmed death protein 1; PD‐L1, programmed death‐ligand 1; PR, partial response; SD, stable disease; T_CM_, central memory T cell; TCR, T cell receptor; TIDC, tumor‐infiltrating dendritic cell; TIL, tumor‐infiltrating lymphocytes; TKI, tyrosine kinase inhibitor; T_N_, naïve T cell; T_PEX_, precursor exhausted T cell; TME, tumor microenvironment; TMV, tumor membrane vesicle.

### 5.1 ICB Therapy

ICB therapy inhibits immune checkpoint molecules and their ligands, restoring T cell function and suppressing tumors [370]. However, as a monotherapy, ICB exhibits limited efficacy, with objective response rates below 30% across multiple tumor types [371]. To better reverse T cell exhaustion, researchers are actively exploring combinatorial strategies that integrate ICB with other therapeutic modalities.

#### 5.1.1 Combination with Immunomodulatory Factors

The initiation and persistence of T cell exhaustion are closely associated with a broad spectrum of immunoregulatory factors, including immunosuppressive cytokines (e.g., TGF‐β and IL‐10), immune checkpoint receptors (e.g., PD‐1, CTLA‐4, LAG‐3, and TIM‐3), and metabolic regulatory enzymes (e.g., IDO and ARG1). These factors contribute to T cell dysfunction by inhibiting T cell activation, amplifying suppressive signaling pathways, and disrupting metabolic homeostasis within the tumor microenvironment.

Cytokines play a central role in regulating T cell differentiation, functional maintenance, and immune microenvironment remodeling. The combination of cytokine‐targeted approaches with ICB has emerged as a key strategy for overcoming T cell exhaustion [372]. For example, IFN‐I drives CD8^+^ TILs toward an exhausted phenotype, a process mediated by chronic IFN‐I signaling that disrupts lipid metabolism and redox balance, exacerbates lipid peroxidation, and promotes T cell dysfunction. Clinical evidence indicates that elevated IFN‐I levels are strongly associated with reduced ICB efficacy [373]. Furthermore, Poly(ADP‐ribose) polymerase 14 (PARP14), a critical mediator of IFN‐γ‐driven immune escape, not only facilitates terminal exhaustion of CTLs but also regulates the polarization of TAMs. In preclinical models, inhibition of PARP14 in combination with PD‐1 blockade significantly enhances antitumor immune responses and ameliorates the immunosuppressive tumor microenvironment [374].

Interleukin‐mediated regulation of T cell exhaustion is closely linked to immunotherapy outcomes. IL‐2 promotes the differentiation of TCF‐1^+^ stem‐like CD8^+^ T cells into effector phenotypes, thereby enhancing the antitumor efficacy of PD‐1 blockade [375]. Joonbeom Bae et al. further demonstrated that engineered mesenchymal stem cells (MSCs) delivering an IL‐2 mutant protein can effectively reverse CD8^+^ T cell exhaustion, highlighting the therapeutic potential of IL‐2‐based interventions [376]. IL‐6 contributes to CD8^+^ T cell dysfunction by upregulating exhaustion markers such as PD‐1. Dual blockade of IL‐6 and PD‐1 has been shown to significantly enhance the cytotoxic function of TILs and suppress tumor growth in vivo [377]. Thus, distinct cytokines exert diverse and context‐dependent effects on T cell exhaustion, and targeted modulation of specific cytokine signaling pathways enables multi‐level intervention in this process, thereby improving the efficacy of ICB. Lipopolysaccharide (LPS) also exhibits a dual role in modulating T cell function. On one hand, LPS promotes T cell infiltration in pancreatic ductal adenocarcinoma (PDAC); on the other hand, it upregulates PD‐L1 expression via the TLR4/MyD88/AKT/NF‐κB signaling pathway, leading to impaired T cell function or induction of exhaustion. Based on these mechanistic insights, combining LPS with PD‐L1 blockade represents a promising integrative therapeutic strategy for PDAC [378].

Moreover, direct targeting of inhibitory molecules associated with T cell exhaustion offers novel opportunities to improve durable responses to immunotherapy. P‐selectin glycoprotein ligand‐1 (PSGL‐1) is a key inhibitory receptor that sustains the exhausted state of T cells and limits the functional recovery of T_PEX_ cells. Rienk Offringa et al. demonstrated that targeting PSGL‐1 not only restores effector function in T_EX_ cells but also acts synergistically with ICB, significantly enhancing T cell reactivation and providing a promising avenue for cancer immunotherapy [379]. Similarly, B7‐H3 impairs ICB efficacy by promoting CD8^+^ T cell exhaustion and reshaping the immunosuppressive TME across multiple cancer types, suggesting that co‐targeting B7‐H3 may represent a viable strategy to overcome limitations imposed by T cell exhaustion in current immunotherapies [380].

#### 5.1.2 Targeting Tumor‐Related Signaling Pathway Regulation

Targeting specific signaling pathways to modulate T cell activation, proliferation, and differentiation has emerged as a pivotal strategy for enhancing the efficacy of ICB. In mismatch repair‐deficient (dMMR) tumor models, the CDK4/6 inhibitor abemaciclib significantly prolongs survival, increases the CD4^+^ to CD8^+^ T cell ratio among TILs, reduces expression levels of T cell exhaustion markers, and diminishes Treg populations. These findings reveal novel therapeutic opportunities for patients resistant to ICB or ineligible for monotherapy [381]. In tumors with low neoantigen burden and poor responsiveness to ICB, transient inhibition of SHP‐1 lowers the TCR activation threshold, markedly enhances the effector function and tumor infiltration of low‐affinity, endogenous autoantigen‐specific CD8^+^ T cells, and effectively reverses the exhausted phenotype [382].

Concurrently, inhibition of the DNA damage response pathway has been shown to augment T cell‐mediated immunity. In hepatocellular carcinoma models, the ATR inhibitor AZD6738 remodels the immunosuppressive TME via activation of the cGAS/STING pathway, reduces the frequency of Tregs and T_EX_ cells, and synergizes with PD‐L1 blockade to enhance the durability of antitumor immune memory [383]. In metastatic renal cell carcinoma, 5α‐reductase inhibitors improve responses to ICB by reducing CD8^+^ T cell exhaustion and PD‐1 expression through modulation of androgen receptor signaling, indicating that androgen signaling represents a promising target for combination immunotherapy [384]. Furthermore, semaphorin 4A (Sema4A) enhances ICB efficacy by promoting the proliferation and cytotoxic activity of tumor‐specific CD8^+^ T cells while suppressing terminal exhaustion, highlighting its potential as both a predictive biomarker and a therapeutic target in combinatorial immunotherapy strategies [385]. Notably, IGF‐Trap, an inhibitor of type I insulin‐like growth factor receptor (IGF‐IR) signaling, effectively alleviates the local immunosuppressive microenvironment in the liver, reduces recruitment of MDSCs, and reverses T cell exhaustion, with its therapeutic efficacy further enhanced when combined with PD‐1 inhibitors [386].

Although targeting tumor‐associated signaling pathways has demonstrated promise in improving T cell function, mitigating exhaustion, and enhancing the efficacy of ICB across multiple preclinical models, significant challenges remain. First, signaling dependencies vary considerably across tumor types, limiting the broad applicability of single‐target interventions. Second, these approaches may induce immune‐related adverse events or disrupt physiological functions in non‐tumor tissues; for example, inhibition of SHP‐1 or IGF‐IR can perturb normal immune homeostasis or endocrine system activity. Therefore, future research should prioritize multi‐target combination therapies, dose optimization, and biomarker‐guided patient stratification to balance therapeutic efficacy with safety and to ensure clinical feasibility across diverse tumor types.

#### 5.1.3 Metabolic and Epigenetic Interventions

Metabolic reprogramming is a key mechanism underlying tumor immune escape. In recent years, targeting tumor metabolic pathways has emerged as a promising strategy to reverse T cell exhaustion and enhance the efficacy of ICB. In tumors exhibiting a preference for aerobic glycolysis, cancer cells efficiently compete for glucose and secrete lactate, thereby exacerbating immunosuppression and promoting the exhausted phenotype of CTLs. Citrate serves as a critical metabolic regulator. Preclinical studies have demonstrated that high‐dose sodium citrate (SCT) can restore glucose availability and inhibit lactate accumulation in the TME by suppressing both tumor glycolysis and oxidative metabolism, consequently enhancing T cell functionality and driving the polarization of TAMs toward a pro‐inflammatory phenotype. The combination of ICIs with SCT therapy represents a promising approach to alleviate the immunosuppressive microenvironment at the metabolic level and improve immunotherapy response rates in solid tumors [387].

Moreover, mitochondrial function plays a crucial role in maintaining CD8^+^ T cell effector capacity and reversing exhaustion. The application of optogenetic technology, termed “OptoMito‐On”, enables remote enhancement of mitochondrial ATP production and improvement of mitochondrial membrane potential in CD8^+^ T cells, leading to increased migratory capacity and cytotoxic activity, effective reversal of the exhausted state, and augmented antitumor immune responses [388]. Thus, metabolic intervention constitutes a novel synergistic strategy to ameliorate the immunosuppressive TME and enhance ICB efficacy through modulation of glycolysis, oxidative metabolism, and mitochondrial function, demonstrating significant potential for combination therapy in solid tumors.

Recent studies have shown that epigenetic regulation is an effective strategy for improving the efficacy of ICB. The histone demethylase lysine‐specific demethylase 1 (LSD1) establishes a distinct epigenetic program in progenitor‐exhausted CD8^+^ T cells that antagonizes TCF‐1‐mediated stemness maintenance and drives their terminal differentiation. Genetic ablation or pharmacological inhibition of LSD1 significantly enhances the persistence of progenitor‐exhausted CD8^+^ T cells, thereby sustaining a pool of terminally differentiated effector cells with enhanced tumoricidal capacity and enabling more durable and robust responses to anti‐PD‐1 therapy [389]. However, although LSD1 inhibition promotes CD8^+^ T cell tumor infiltration, its cytotoxic potential is limited by the induction of TGF‐β expression, which compromises antitumor immunity. Recent evidence indicates that combining LSD1 inhibition with TGF‐β blockade and PD‐1 antibody treatment significantly enhances CD8^+^ T cell infiltration and cytotoxic function, reverses the exhausted phenotype, and achieves tumor eradication and long‐term immune protection in poorly immunogenic tumor models. These findings suggest that this triple‐combination regimen holds substantial promise for overcoming T cell exhaustion and improving the therapeutic efficacy of ICB [390].

#### 5.1.4 Targeted Delivery

Targeted delivery strategies enable the precise localization of ICIs and related therapeutic agents to the TME, thereby enhancing T cell activity, mitigating T cell exhaustion, and improving the efficacy of combination immunotherapy. A variety of delivery platforms are currently under development, including viral vectors, engineered extracellular vesicles (EVs), and targeted systems for small molecules and siRNA, offering innovative approaches to overcome tumor‐induced immunosuppression.

The integration of viral vectors with ICB has demonstrated significant synergistic effects. For example, combining anti‐LAG‐3 therapy with cowpea mosaic virus‐based immunotherapy (CPMV IT) reduces T cell exhaustion and enhances local immune activation, providing new insights into improving cancer immunotherapy outcomes [391]. Similarly, a tumor‐selective oncolytic herpesvirus vector (YST‐OVH) enables site‐specific delivery of PD‐1 inhibitors and synergizes with other checkpoint blockades [392]. Beyond viruses, engineered EVs offer another promising platform. Zhang et al. designed EVs to overexpress PD‐1 protein and co‐administered them with CTLs, achieving dual functional benefits: not only blocking PD‐1/PD‐L1‐mediated immune evasion but also inducing tumor cell apoptosis via factor‐related apoptosis ligand (FasL) and granzyme B (GzmB). This strategy leverages the intrinsic trafficking capacity of EVs to deliver immunomodulatory agents directly to the TME, simultaneously activating effector T cells while ensuring high target specificity and enhanced immune stimulation, thus representing a compelling approach for reversing T cell exhaustion. Targeted siRNA delivery has demonstrated unique advantages in treating tumors at anatomically challenging sites. In glioblastoma, for instance, EV‐loaded siRNA can specifically target PD‐L1 on tumor cell surfaces, cross the blood‐brain barrier, and achieve complete blockade of the PD‐1/PD‐L1 axis, thereby enhancing T cell cytotoxicity and reversing the exhausted phenotype [393]. This approach addresses key limitations of conventional drug delivery systems and expands the applicability of immunotherapy to tumors residing in immunologically hostile or physically inaccessible microenvironments.

#### 5.1.5 Dual ICIs Strategy

T cells exhibit distinct functional states and differentiation trajectories across different stages of exhaustion. Therefore, the rational design of ICI combinations tailored to specific phases of exhaustion enables precise therapeutic intervention and optimization of clinical outcomes. In the early stage of T cell hyporesponsiveness, dual blockade of PD‐1 and CTLA‐4 effectively prevents the upregulation of multiple inhibitory receptors, limits the progression of T cell dysfunction, and preserves effector potential [394]. During the critical transition from effector to exhausted state, short‐term ICI intervention can downregulate inhibitory receptor expression and promote functional recovery of effector Th1 and CD8^+^ T cells. For example, in a peritoneal dissemination model of gastric cancer, while anti‐PD‐1 and anti‐CTLA‐4 therapy enhances CD8^+^ T cell tumor infiltration, resistance is frequently associated with aberrant activation of the JAK‐STAT pathway and accumulation of immunosuppressive cell populations. The addition of Janus kinase inhibitors restores CD8^+^ T cell function, remodels the TME, and significantly improves therapeutic efficacy [395]. Moreover, studies in melanoma patients have demonstrated that anti‐CTLA‐4 promotes the expansion of progenitor T cells, and when combined with anti‐PD‐1, drives clonal expansion of CD8^+^ T cells, thereby enhancing clinical response rates [396]. Interventions during this phase aim to selectively reactivate partially dysfunctional but not their T_EX‐term_ cells, thereby enhancing downstream antitumor immunity.

In the terminal exhaustion phase, T cell function is severely compromised and exhibits limited responsiveness to monotherapy with ICIs. A neoadjuvant study in patients with head and neck squamous cell carcinoma showed that the combination of anti‐PD‐1 and anti‐LAG‐3 antibodies was more effective than CTLA‐4 blockade in reprogramming the transcriptional profile of CD8^+^ TILs, promoting their conversion from an exhausted phenotype toward effector or tissue‐resident memory T cell states. This dual regimen also enhanced TCR diversity and facilitated clonal expansion [397].

#### 5.1.6 Combining ICIs with Other Therapies

A variety of therapeutic modalities, including chemotherapy, radiotherapy, nano‐immunomodulators, and cancer vaccines, offer promising combinatorial approaches for treating drug‐resistant and poorly immunogenic tumors by improving the TME, enhancing antigen presentation, and activating T cell effector functions.

The combination of radiotherapy and ICB demonstrates significant immunostimulatory effects. Key mechanisms include the induction of damage‐associated molecular patterns (DAMPs) and chemokine release, promotion of DC and CTL infiltration, reversal of T cell exhaustion, conversion of “cold” to “hot” tumor phenotypes, and enhancement of ICB responsiveness [398]. A randomized phase I trial showed that in patients with immunologically “cold” tumors and high tumor aneuploidy, concurrent administration of radiotherapy and ICB resulted in significantly higher local and distant response rates and prolonged overall survival compared to sequential therapy, an effect strongly associated with enhanced antigen presentation [399, 400]. Similarly, Philip J. Saylor et al. demonstrated that radium‐223 radionuclide therapy effectively alleviates T cell exhaustion in metastatic prostate cancer by promoting intratumoral CD8^+^ T cell proliferation and infiltration, with a marked survival benefit when combined with anti‐PD‐1 therapy [401]. Collectively, radiotherapy and targeted radionuclide therapies represent robust strategies to overcome T cell dysfunction through multi‐level activation of antitumor immunity.

Accumulating evidence indicates that conventional chemotherapy exerts not only direct cytotoxic effects but also enhances the efficacy of ICB by remodeling TIME. Recent single‐cell transcriptomic and trajectory analyses using CITE‐seq revealed that neoadjuvant low‐dose metronomic chemotherapy (MCT) induces dynamic reprogramming of myeloid cell subsets in TNBC, thereby establishing an immunologically favorable microenvironment conducive to improved immunotherapy outcomes [402]. In colorectal cancer models, FOLFOX chemotherapy promotes the transition of CD8^+^ TILs to a high‐responsiveness phenotype characterized by reduced expression of PD‐1 and TIM‐3, thereby increasing their sensitivity to ICB. The combination of FOLFOX with anti‐PD‐1 therapy significantly suppresses tumor growth [403]. Furthermore, the paclitaxel‐loaded nano‐immunomodulator Nano‐PI, when administered in conjunction with anti‐PD‐1, enhances T cell activity, effectively inhibits breast cancer metastasis, and achieves durable responses [404]. Together, these findings highlight chemotherapy as a viable strategy for synergizing with ICB through immune microenvironment modulation and functional reprogramming of T cells.

Vaccines and costimulatory agonists can further enhance T cell function and antitumor responses. For example, the λ phage vaccine targeting the tumor‐associated antigen ASPH, when combined with PD‐1 blockade, synergistically improves TIME in TNBC and hepatocellular carcinoma by activating CD4^+^ Th1 cells and CD8^+^ CTLs, promoting the formation of tertiary lymphoid structures, and reducing Treg infiltration. This combination significantly inhibits tumor progression and prolongs survival [405]. Intratumoral dual CD40‐TLR4 stimulation reverses irreversible T cell exhaustion resistant to PD‐1 blockade by eliminating exhausted PD‐1hi T cells and inducing systemic antitumor CD8^+^ T cell responses, thereby restoring sensitivity to immunotherapy [406]. The novel bifunctional agonist STAR0602 targets specific Vβ TCR subpopulations and cis‐activates IL‐2 signaling, significantly expanding CD4^+^ and CD8^+^ T cell populations exhibiting atypical effector memory phenotypes. This overcomes the constraints of T cell exhaustion and demonstrates durable antitumor effects across multiple PD‐1–refractory solid tumor models [407]. Vaccines and costimulatory agonists offer novel pathways for immune reactivation in drug‐resistant tumors through direct activation or expansion of T cell populations.

### 5.2 Adoptive Cell Therapy

ACT enhances antitumor immune responses by expanding TILs or peripheral blood‐derived immune cells ex vivo and reinfusing them into patients [408]. Similar to ICB therapy, the efficacy of ACT relies on CD8^+^ T cell‐mediated antitumor immunity within the TME. Consequently, T cell exhaustion not only impairs anticancer immune responses but also constitutes a direct mechanism of resistance to ACT.

**FIGURE 7:**
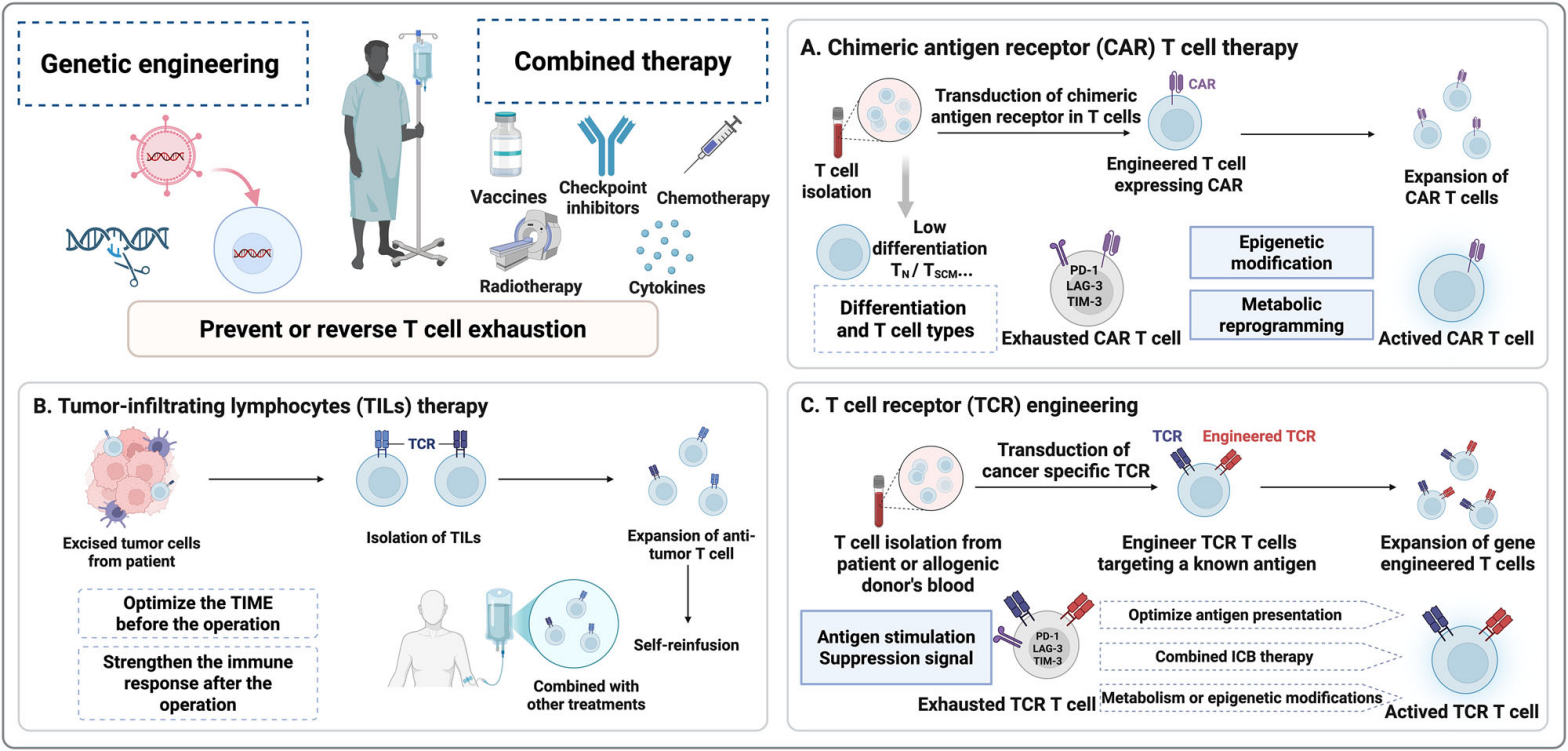
Multidimensional strategies to target T cell exhaustion to enhance ACT. A.CAR‐T therapy: T cells are isolated from patients and transduced with a CAR) to generate engineered T cells that express CAR molecules. Exhausted CAR‐T cells can be reactivated through epigenetic modifications and metabolic reprogramming, while genetically modified T cells can be further expanded ex vivo to enhance their anti‐tumor efficacy. B.TIL therapy: Antitumor T cells are isolated from resected tumor tissues, expanded in vitro, and subsequently reinfused autologously. By optimizing the TIME prior to surgery and augmenting the immune response postoperatively, functional exhaustion of TILs can be effectively reversed. C.TCR engineering therapy: Peripheral T cells are isolated and transduced with TCRs specifically targeting tumor‐associated antigens. For TCR‐T cells that have undergone functional exhaustion due to persistent antigen exposure and inhibitory signaling, restoration of function can be achieved through improved antigen presentation, combination immunotherapies, and modulation of metabolic or epigenetic pathways. Abbreviations: ACT, adoptive cell therapy; CAR‐T, chimeric antigen receptor T cell; CAR, chimeric antigen receptor; TIL, tumor‐infiltrating lymphocyte; ICB, immune checkpoint blockade; TCR, T cell receptor; TIME, tumor immune microenvironment; T_N_, Naïve T cell; T_SCM_, T stem cell memory.

To overcome this limitation, various strategies have been developed, among which genetic engineering has emerged as a pivotal approach. Genetic engineering can partially restore T cell proliferation and effector function by modulating exhaustion‐associated transcription factors, immune checkpoints, or epigenetic programs, thereby offering a promising strategy to enhance the potency of ACT [409] (Table 3). It should be noted that such interventions may induce adverse effects, including aberrant T cell proliferation. For instance, biallelic deletion of ten‐eleven translocation 2 (TET2) in combination with forced expression of BATF3 can lead to antigen‐independent clonal expansion of CAR‐T cells, posing potential safety risks [410]. In light of these challenges, this section presents a systematic review of therapeutic strategies aimed at reversing T cell exhaustion, with a focus on recent advances in three representative ACT modalities—CAR‐T, TCR‐T, and TILs therapy—providing a theoretical foundation and practical guidance for optimizing cellular immunotherapy (Figure 7).

**TABLE 3:** Studies on gene engineering modification to reverse T cell exhaustion and enhance the efficacy of ACT.

ACT	Genetic engineering techniques	Type of cancer	Target	Result	References
CAR‐T	Lentivirus‐mediated co‐expression of c‐Jun	Solid tumors	HA‐28z	Reduce and/or replace the AP‐1/IRF transcription complex in the chromatin, and resist depletion	[411]
	Lentivirus‐mediated co‐expression of BATF	melanoma	CD19	Increase the production of effector cytokines and reduce the expression of inhibitory receptors and exhaustion‐related transcription factor TOX	[314]
	Retrovirus‐mediated co‐expression of IL‐10	Solid tumors	HER2/TRP‐1 EGFRvIII / CD19	Increase IL‐10 secretion, enhance mitochondrial oxidative phosphorylation, induce stem cell‐like memory response, and resist T cell exhaustion	[412]
	Revertendo virus‐mediated co‐expression of IL‐18	SCLC	DLL3	Increase gene phenotype and T cell activation, and reduce exhaustion	[413]
	Lentivirus‐mediated co‐expression of FOXO1	Solid tumors	CD19	mote the emergence of the CD8^+^ CAR T cell subset, characterized by high expression of CD62L, IL7R, and KLF2	[414]
	Lentivirus‐mediated co‐expression of RUNX3	hepatoma	GPC3	The reduction of AICD and TNF signals, along with enhanced proliferation and enhanced effector functions, delays or reduces T cell exhaustion	[415, 416]
	Lentivirus‐mediated co‐expression of IL‐7	prostatic cancer	NKG2D	Promote glucose uptake and anti‐apoptotic ability, upregulate Bcl‐2, and inhibit PD‐1 and Tim‐3	[417]
	Lentivirus‐mediated co‐expression of BATF3	/	HER‐2	Significantly increase the expression of IL‐7R and reduce the expression of exhaustion markers (LAG3, TIGIT, TIM3, CISH)	[418]
	CRISPR‐Cas9 knockout of BATF	Solid tumors	/	Promote the transformation of cell populations into central memory subsets, resist the exhaustion of CAR‐T cells, and enhance their cytotoxic activity	[419]
	Retrovirus‐mediated co‐expression of IL‐15‐IL‐15 Rα	B‐cell malignancy	CD 19	Preserves the T_scm_ phenotype and results in enhanced self‐renewal capacity	[420]
	CRISPR‐Cas9 knockout of SOX4/ID3	pancreatic cancer	MSLN	Block or reverse the transcriptional program that leads to T cell exhaustion, prevent T cells from transforming into NK‐like state, and enhance the effector function of CAR‐T cells	[421]
	CRISPR‐Cas‐mediated double knockout of PRDM1 and NR4A3	prostatic cancer	PSMA	The CAR T cell phenotype deviates from TIM‐3CD8 and leans towards TCF1CD8, in order to counteract the exhaustion of tumor‐infiltrating CAR T cells	[422]
Retrovirus‐mediated triple knockout of Nr4a1/2/3	Colon cancer/melanoma/ T‐cell lymphoma	CD19	Block the NFAT‐driven exhaustion transcriptional pathway, transforming CAR T cells from the exhausted state to the effector state	[330]
	Retrovirus‐mediated dual knockdown of TOX/TOX2	/	CD19	T cells retain their effector functions and reduce the upregulation of inhibitory receptors	[10]
	Lentivirus‐mediated co‐expression of SMAD7	Solid tumors	HER‐2 CD19	Resist TGF‐β‐triggered T cell exhaustion and prevent cytokine release, enhancing the efficacy of CAR‐T cells	[423]
	CRISPR‐Cas9 knockout of MAP4K1	/	CD19 HER‐2	Blocking the HPK1‐Blimp1 axis, improving chromatin accessibility and enhancing TCR signaling, alleviating T cell exhaustion	[424]
	Lentivirus‐mediated gene silencing of PD‐1	MM	BCMA	Increase the proportions of Naïve and T_cm_ subsets, and reduce the exhaustion of CAR‐T cells	[425]
	Retrovirus‐mediated co‐expression of PD‐1 DNR	malignant pleural mesothelioma	MSLN	PD‐1 DNR competitively binds to PD‐L1/PD‐L2 to block the endogenous PD‐1 signal and resist exhaustion	[426]
	Lentivirus‐mediated gene silencing of PD‐1/Tim‐3/LAG‐2	/	HER‐2	Eliminating inhibitory signals reverses T cell exhaustion and upregulates CD56 to enhance antitumor effects	[427]
	CRISPR‐Cas9 knockout of PRDM1	/	CD19	Inhibiting terminal differentiation, maintaining the early memory phenotype (T_cm_/T_scm_), and enhancing the secretion of multifunctional cytokines	[428]
TCR‐T	Retrovirus‐mediated co‐expression of IL15+IL21	Solid tumors	KKLC1/HPV16 E7/MART‐1	Reduce the proportion of NK‐like cells	[429]
	Lentivirus‐mediated co‐expression of c‐Jun	hepatoma	AFP_158_	The expression of LAG3 and TIM3 was significantly reduced, the exhaustion of TCR‐T cells was decreased, and the expansion was increased.	[430]
	Retrovirus‐mediated co‐expression of Epas1	Solid tumors	OVA	Maintaining the T_PEX_ cell phenotype and reducing exhaustion markers can effectively delay or reverse the exhaustion of TCR‐T cells	[431]
	Retrovirus‐mediated co‐transduction of TCR and PD‐1–41BB fusion receptor	melanoma	PRAME	Transform the inhibitory signals of tumor cells on PD‐L1 expression into co‐stimulation, thereby enhancing the efficacy of TCR‐T therapy	[432]
	CRISPR‐Cas9 knockout of TIM‐3 / 2B4	MM	NY‐ESO‐1	Maintain the degranulation function of T cells	[433]
	CRISPR‐Cas9 knockout of LAG3	MM	NY‐ESO‐1	Compensatory upregulation of other inhibitory receptors	[433]
TIL	CRISPR‐Cas9 knockout of CISH	Solid tumors	/	Remove negative regulation and enhance the activation and antigen sensitivity of TIL	[434]
	CRISPR‐Cas9 knockout of AKT1/2	Cervical cancer / Ovarian cancer	/	Regulating the PI3K‐AKT signaling pathway enhances the memory phenotype and cytotoxicity of TILs	[434]
	Lentivirus‐mediated TIL secretion of bispecific T‐cell engager (STAb)	NSCLC	/	Redirect non‐tumor dominant clonal‐mediated anti‐tumor responses and alleviate exhaustion	[435]
	Lentivirus‐mediated co‐expression of TCR and CoStAR	melanoma	/	Synergistically enhance TCR signals, provide continuous co‐stimulation, and delay TIL exhaustion	[436]

Abbreviations: ACT, adoptive cell therapy; CAR‐T, chimeric antigen receptor T cell; MM, multiple myeloma; NSCLC, non–small cell lung cancer; SCLC, small cell lung cancer; T_cm_, central memory T cell; TCR, T cell receptor; TCR‐T, T cell receptor‐engineered T cells; TIL, tumor‐infiltrating lymphocyte; T_N_, naïve T cell; T_PEX_ cell, precursor exhausted T cell; Tscm, stem cell memory T cell.

#### 5.2.1 CAR‐T Therapy

Among various ACT, CAR‐T therapy has demonstrated considerable potential due to its ability to specifically recognize tumor antigens and its MHC‐independent mechanism of action. By genetically engineering T cells to express chimeric antigen receptors (CARs), CAR‐T cells can specifically target and eliminate malignant cells, thereby mediating potent antitumor activity [437, 438]. However, approximately 75% of patients experience disease recurrence following treatment, a phenomenon closely associated with tumor antigen escape mechanisms as well as the limited persistence and functional durability of CAR‐T cells in vivo [240, 439].

Clinical evidence has clearly established that T cell exhaustion is a key factor limiting the efficacy of CAR‐T therapy. In a study of 24 patients with large B‐cell lymphoma (LBCL) treated with axicabtagene ciloleucel (Yescarta), the proportion of memory CD8^+^ T cells within the infused CAR‐T product was significantly higher in patients achieving sustained complete response (CR) compared to those with partial response (PR) or progressive disease (PD). Conversely, the frequency of exhausted CD8^+^ T cells showed an inverse correlation [440]. Another clinical study involving 32 patients with LBCL demonstrated that memory‐like CD8^+^ T cell populations were significantly associated with response to tisagenlecleucel (Kymriah), but not to axicabtagene ciloleucel [441]. These findings indicate that the differentiation status of T cells in CAR‐T products—particularly the balance between exhausted and memory T cell subsets—is a critical determinant of clinical outcomes.

Accumulating evidence suggests that the antitumor activity of CAR‐T cells is regulated by multiple molecular mechanisms, including transcriptional regulatory networks, chronic antigen stimulation, and antigen‐independent signaling pathways. Moreover, the degree of T cell exhaustion is inversely correlated with the proportion of memory T cells [3]. A comprehensive understanding of these regulatory mechanisms will provide a robust theoretical foundation for the rational design of next‐generation CAR‐T products and for developing effective strategies to mitigate T cell exhaustion.

##### 5.2.1.1 CAR‐T Cell Type and Differentiation Status

The type and differentiation status of T cells are critical determinants of the efficacy of CAR‐T therapy. Accumulating evidence indicates that although Naïve T cells and T stem cell memory (T_SCM_)‐enriched CAR‐T products exhibit relatively limited cytotoxic activity in vitro, they demonstrate enhanced antitumor effects, greater proliferative capacity, and improved persistence in vivo, along with reduced T cell exhaustion and a more pronounced memory phenotype. Compared to unselected bulk T cell products, T_N/SCM_‐derived CAR‐T cells are associated with a lower risk of cytokine release syndrome (CRS), exhibit attenuated T cell activation, and display a higher therapeutic safety index [442]. Furthermore, co‐expression of IL‐15 with GD2‐targeting CAR effectively preserves the central memory and stem cell‐like phenotypes and reduces PD‐1 expression, thereby delaying functional exhaustion [443, 444].

The superior performance of less differentiated T cell subsets in CAR‐T therapy has been consistently demonstrated. CD19‐directed CAR‐T cells enriched for T_SCM_—the most primitive T cell subset with self‐renewal capacity—exhibit enhanced proliferative activity in vitro, as well as prolonged persistence and superior tumor control in vivo [445, 446, 447]. Cong He et al. reported that NKG2D‐IL7 CAR‐T cells promote CD8^+^ T cell proliferation and upregulate B‐cell lymphoma 2 (Bcl‐2) expression, thereby reducing apoptosis and mitigating functional exhaustion—a mechanism closely linked to the enrichment of poorly differentiated T cell phenotypes [417].

Since CAR‐T cell exhaustion predominantly occurs in terminally differentiated effector T cells, enriching early‐differentiated T cell populations—particularly naïve and T_N/SCM_ or T_SCM_ subsets—during the initial stages of CAR‐T product manufacturing can help prevent premature functional exhaustion and enhance long‐term antitumor efficacy. In summary, optimizing the composition and differentiation state of CAR‐T cells not only reduces T cell exhaustion but also establishes a robust theoretical and technical foundation for the development of durable, effective, and safe adoptive cell therapies.

##### 5.2.1.2 Regulation of CAR‐T Cell Metabolism

In the heterogeneous TME, T cell function is impaired by metabolic constraints, which significantly limit the differentiation, proliferation, and antitumor persistence of CAR‐T cells, ultimately compromising therapeutic efficacy [448]. In recent years, multidimensional intervention strategies targeting metabolic reprogramming have shown considerable promise in mitigating T cell exhaustion and enhancing CAR‐T cell functionality. Restoring mitochondrial function represents a central mechanism. Exogenous supplementation of mitochondria has been demonstrated to substantially improve energy metabolism and enhance functional capacity in T cells. This approach enhances antitumor activity and represents a novel synergistic strategy for T cell‐based immunotherapy [449]. Similarly, upregulation of Complement component 1 Q subcomponent‐binding protein (C1QBP) expression in T cells helps preserve mitochondrial integrity, suppress ROS accumulation, and activate anti‐apoptotic signaling pathways, thereby improving the antitumor efficacy of CAR‐T therapy [450]. Moreover, hypoxia‐responsive CAR‐T cells engineered with hypoxia response elements can selectively induce CAR expression within tumor‐specific hypoxic niches, effectively alleviating T cell exhaustion and enhancing both the safety and efficacy of solid tumor therapy [451].

Regulation of glucose metabolism has also been established as a critical determinant in reversing T cell exhaustion. Forced expression of the glucose transporter1 (GLUT1) confers metabolic advantages to CAR‐T cells in glucose‐deprived TME, promotes the development of a stem cell‐like memory phenotype, and reduces cellular exhaustion [452]. Stable overexpression of GLUT1 not only enhances glycolytic flux and antioxidant capacity but also increases secretion of proinflammatory cytokines and drives Th17 differentiation, thereby augmenting cytotoxicity and long‐term antitumor persistence [453]. During in vitro expansion, the addition of IL‐15 reduces glycolytic activity by inhibiting the mTOR‐GLUT4 signaling pathway, improves T cell metabolic fitness, mitigates exhaustion, and supports the survival and effector function of CAR‐T cells [454].

Cholesterol deficiency in the TME also impairs CAR‐T cell proliferation and induces autophagy‐mediated apoptosis. Targeted modulation of liver X receptor β (LXRβ) can restore cholesterol homeostasis, enhance membrane integrity, and improve antitumor efficacy [455]. Concurrently, sustained overactivation of calcium signaling leads to excessive store‐operated calcium entry (SOCE), a key driver of CAR‐T cell exhaustion. The SOCE inhibitor BTP‐2 significantly reduces T cell exhaustion and terminal differentiation while enhancing in vivo persistence and antitumor activity, an effect mediated by suppression of the SOCE–calcineurin–NFAT and glycolytic pathways [456]. Furthermore, arginine deprivation in the TME limits CAR‐T cell proliferation and undermines clinical outcomes. Genetic engineering to express functional argininosuccinate synthase (ASS) or ornithine transcarbamylase (OTC) enables metabolic adaptation, restores proliferative capacity, and attenuates the exhausted phenotype [457].

##### 5.2.1.3 Epigenetic Modification of CAR‐T Cells

Epigenetic regulation plays a critical role in the development of CAR‐T cell dysfunction and exhaustion, with DNA methylation representing a central mechanism. Studies have demonstrated that DNMT3A‐mediated DNA methylation restricts the functional plasticity of CAR‐T cells and promotes exhaustion, whereas genetic ablation of DNMT3A not only enhances CAR‐T cell functionality but also increases IL‐10 expression, revealing the molecular basis of DNA methylation‐driven exhaustion. These findings provide a novel rationale for improving the long‐term antitumor efficacy of CAR‐T cells through epigenetic modulation [75]. Further evidence shows that the DNA methyltransferase inhibitor decitabine sustains high expression levels of memory‐associated genes and reduces expression of exhaustion‐related genes, thereby delaying functional decline in CAR‐T cells [458]. Additionally, the use of piggyBac transposons with low CpG content has been shown to reduce TLR9‐mediated inflammatory responses, suppress DNA methylation‐induced T cell exhaustion, significantly enhance antitumor activity, promote expansion of TILs, and decrease pro‐inflammatory cytokine release, ultimately supporting sustained CAR‐T cell function [459]. Collectively, these results underscore that targeting DNA methylation is a promising strategy to mitigate CAR‐T cell exhaustion.

Histone modification represents another effective approach to enhancing CAR‐T cell functionality. High‐throughput studies have demonstrated that class I HDACi, such as M344 and chidamide, significantly improve memory maintenance and resistance to exhaustion by reducing HDAC1 expression, enhancing H3K27ac activity, and activating key genes in the Wnt/β‐catenin signaling pathway, thereby augmenting antitumor efficacy [460]. Maik Luu et al. further confirmed that in vitro treatment with valerate and butyrate inhibits class I histone deacetylase activity and promotes the secretion of effector molecules—including CD25, IFN‐γ, and TNF‐α—resulting in markedly enhanced antitumor activity of antigen‐specific CTLs and ROR1‐targeted CAR‐T cells in syngeneic mouse models of melanoma and pancreatic cancer [461]. Furthermore, Zhang et al. found that odd‐skipped related 2 (Osr2) recruits HDAC3 to reshape the epigenetic landscape by integrating TCR signaling with the Piezo1/calcium/CREB axis‐mediated mechanical stress response, selectively inducing terminal exhaustion and suppressing cytotoxic gene expression. Notably, Osr2 knockout markedly alleviates exhaustion in tumor‐specific T cells and CAR‐T cells and enhances their antitumor function [462].

Beyond DNA methylation and histone modification, chromatin remodeling also contributes to the regulation of CAR‐T cell exhaustion. Elena Battistello et al. conducted a systematic analysis of the genome‐wide occupancy of the mSWI/SNF chromatin remodeling complex during acute and chronic T cell stimulation, revealing that this complex modulates T cell activation and phenotypic differentiation by regulating chromatin accessibility. Pharmacological or genetic inhibition of mSWI/SNF function enhances T cell persistence, alleviates CAR‐T cell exhaustion, and improves both expansion and antitumor efficacy in vivo, thereby providing a novel theoretical foundation for combining small‐molecule therapeutics with immunotherapy [82].

##### 5.2.1.4 Combining CAR‐T With Other Therapies

In the clinical application of CAR‐T therapy, monotherapy is frequently limited by TIME and T cell exhaustion, leading to suboptimal therapeutic outcomes in certain patient populations. Consequently, combining CAR‐T therapy with complementary treatment modalities to enhance antitumor activity and long‐term persistence has become a major focus of current research.

Among various combination strategies, small molecule inhibitors and OVs have demonstrated significant synergistic effects. Studies have shown that MEK1/2 inhibitors can reduce CAR‐T cell exhaustion and terminal differentiation by suppressing the MAPK‐c‐Fos‐JunB signaling axis, thereby substantially improving CAR‐T cell efficacy in both hematologic malignancies and solid tumors [25]. Dasatinib, a tyrosine kinase inhibitor targeting abelson tyrosine kinase (ABL), can transiently inhibit CAR‐T cell signaling, maintain cells in an early differentiation state, and prevent activation‐induced cell death [463]. Furthermore, OVs enhance antitumor immunity by releasing immunostimulatory signals, promoting DC maturation, and facilitating the recruitment of CAR‐T cells and other immune effector cells to tumor sites [464, 465].

Traditional therapies such as chemotherapy and radiotherapy also play crucial roles in combination regimens. Preclinical studies indicate that administering chemotherapy 3–6 days prior to CAR‐T cell infusion reduces tumor burden, improves the TME, and promotes CAR‐T cell infiltration, expansion, and long‐term persistence [466]. Radiotherapy, meanwhile, enhances CAR‐T cell antitumor responses by inducing tumor antigen release, promoting recruitment of cytotoxic T cells, and alleviating T cell exhaustion. The combination of radiotherapy with CAR‐T therapy not only enhances therapeutic efficacy but also reduces the required radiation dose and improves CAR‐T cell migration to tumor sites [467].

Clinical evidence further supports the benefits of combination approaches. A study showed that PD‐1 blockade using pembrolizumab reversed post‐infusion T cell exhaustion following CD19‐directed CAR‐T therapy and significantly enhanced CAR‐T cell activation and proliferation. In a cohort of 12 patients with relapsed or refractory B‐cell lymphoma, the combination of pembrolizumab and CAR‐T therapy achieved an overall response rate of 25%, including one complete response, two partial responses, and one stable disease, resulting in a clinical benefit rate of 33% [468]. These results indicate that ICIs can effectively mitigate CAR‐T cell exhaustion and improve clinical outcomes.

Notably, the “drug‐induced transient rest” strategy proposed by Evan W. Weber et al. represents a novel approach to functional recovery of CAR‐T cells. By transiently downregulating CAR protein expression or using the multikinase inhibitor dasatinib, this strategy promotes acquisition of a memory‐like phenotype, induces global transcriptional and epigenetic reprogramming, and effectively reverses T cell exhaustion while restoring antitumor activity [469]. These findings challenge the traditional notion that T cell exhaustion is a fixed and irreversible state, and provide a robust theoretical foundation and promising clinical avenue for optimizing CAR‐T therapy through rational combination strategies.

#### 5.2.2 TIL Therapy

TILs are a heterogeneous population of lymphocytes that naturally infiltrate the TME and actively participate in antitumor immune responses. A substantial proportion of these cells are T cells capable of recognizing tumor‐specific antigens and neoantigens. ACT therapy, which involves the ex vivo expansion and reinfusion of autologous TILs into cancer patients, has demonstrated considerable clinical potential in recent years [470]. Although TIL‐based ACT leverages patient‐derived T cells to target tumor‐specific antigens with high specificity, its therapeutic efficacy is frequently limited by T cell exhaustion. Upon prolonged exposure to an immunosuppressive TME in vivo, TILs progressively lose their proliferative capacity and effector function. They also exhibit upregulated expression of inhibitory receptors such as PD‐1 and TIM‐3. Therefore, optimizing TIL expansion protocols, combining ICIs, and modulating metabolic or epigenetic states have emerged as key strategies to reverse TIL exhaustion and enhance the potency of ACT therapy. These approaches align closely with exhaustion‐targeted interventions in CAR‐T and TCR‐T therapies, highlighting that multidimensional regulation of T cell function constitutes a central paradigm for achieving durable and effective cancer immunotherapy.

##### 5.2.2.1 Preoperative and Postoperative Strategies for Modulating the Tumor Microenvironment

In TIL therapy, T cell exhaustion represents a key limitation to antitumor efficacy. To enhance the survival, functionality, and in vivo persistence of TILs, researchers routinely combine TIL infusion with preoperative and postoperative modulation of the TME to optimize antitumor immune responses. Preoperative lymphodepletion—typically achieved through chemotherapy (e.g., fludarabine or cyclophosphamide) or total body irradiation (TBI)—has significantly improved objective response rates in patients with metastatic melanoma [471]. The therapeutic benefit of lymphodepletion is partially attributed to increased availability of homeostatic cytokines such as IL‐7 and IL‐15, which are produced by non‐lymphoid tissues and play critical roles in T cell proliferation and memory formation. Transient exhaustion of endogenous lymphocytes and NK cells results in elevated serum levels of IL‐7 and IL‐15, a phenomenon known as the “cytokine sink” effect that has been strongly associated with long‐term survival and favorable clinical outcomes in TIL‐treated patients [470]. Thus, preoperative optimization of the TME establishes a supportive milieu for adoptively transferred TILs by enhancing their activation, functional capacity, and antitumor activity through improved cytokine availability.

Postoperative interventions primarily involve vaccination within prime‐boost regimens. Vaccines can activate tumor‐specific T cells and act synergistically with TIL infusion to amplify antitumor immunity [472, 473]. Additionally, vaccines may be administered as a booster after ACT therapy to promote reactivation of infused TILs and facilitate their infiltration into tumor sites [474]. By providing sustained antigenic stimulation, vaccine‐based strategies combined with TIL therapy may delay the onset of T cell exhaustion and promote differentiation toward memory phenotypes, thereby enhancing the therapeutic efficacy of TIL therapy in solid tumors.

##### 5.2.2.2 Optimization of In Vitro Expansion Protocols

In TIL therapy, ex vivo expansion is a critical step for generating sufficient numbers of functional T cells for adoptive transfer. The clinical response to TIL‐based adoptive cell transfer depends on the presence and persistence of tumor‐specific CD8^+^ T cell clones [475]. However, prolonged in vitro culture often induces T cell exhaustion, compromising TIL survival and antitumor efficacy upon reinfusion. To address this limitation, researchers have developed optimized in vitro expansion strategies aimed at preserving T cell functionality, stemness, and antitumor potential.

Culturing TILs in a “synthetic immune niche” (SIN) composed of immobilized CCL21 and ICAM‐1 significantly enhances proliferative capacity and cytotoxicity, reduces expression of the exhaustion marker TIM‐3, and increases secretion of IFN‐γ and granzyme B, thereby effectively suppressing tumor growth [476]. Additionally, co‐stimulation with IL‐2, IL‐15, and IL‐21 during TIL expansion from glioma patients generates a population enriched in central memory phenotype cells, characterized by reduced expression of exhaustion markers and enhanced cytokine production and cytotoxic activity against autologous tumors and shared antigens [477]. Combining the SIN platform with multi‐cytokine stimulation provides an efficient and reproducible method for in vitro TIL expansion, enabling simultaneous optimization of cellular phenotype and functional potency.

The phenotypic composition of expanded TILs directly influences therapeutic efficacy, making the selective enrichment and maintenance of stem cell‐like and effector T cell subsets during in vitro expansion critically important. Studies have demonstrated that CD27^−^ TILs exhibiting an effector phenotype exhibit superior therapeutic efficacy compared to CD27^+^ TILs with a memory phenotype [478]. Further analysis of TIL infusion products has revealed that a small subset of stem cell‐like, CD39^−^CD69^−^ tumor‐reactive T cells is strongly associated with durable clinical responses [479]. Activation of the ProS1‐MerTK signaling pathway promotes central memory differentiation, enhances metabolic adaptability and proliferative capacity in CD4^+^ T cells, drives type 1 immune responses, and concurrently reinforces stemness while mitigating exhaustion during TIL expansion, thereby improving the efficacy of ACT [480]. Moreover, reprogramming exhausted TILs into induced pluripotent stem cells (iPSCs), followed by redifferentiation into heterogeneous T cell populations enriched in naïve and memory phenotypes, represents a promising strategy to overcome the limitations of conventional expansion protocols—particularly the scarcity of functional T cells—and significantly enhances antitumor activity [481].

##### 5.2.2.3 Combining TILs With Other Therapies

The combination therapy strategy has demonstrated significant potential in optimizing TIL therapy. The core of this approach lies in delaying or reversing T cell exhaustion and enhancing the durability and antitumor functionality of TILs through the synergistic action of various immunomodulatory interventions. For instance, cytokine modulation via membrane‐bound IL‐15 reduces the proportion of terminally differentiated T cells, downregulates expression of inhibitory receptors such as LAG‐3, TIGIT, and TIM‐3, promotes the generation of stem‐like CD8^+^ T cells, and significantly enhances TIL persistence and antitumor efficacy in the treatment of NSCLC [482].

Combined epigenetic interventions provide a novel and optimized approach for enhancing TIL therapy. Studies have demonstrated that EZH2 inhibitors can block H3K27me3‐mediated gene silencing, improve the differentiation status and functional capacity of TILs, and enhance synergy with ICIs [483, 484, 485, 486]. Bromodomain and extra‐terminal domain (BET) inhibitors increase the proportion of stem cell‐like and central memory T cells, reduce expression of Th17 differentiation‐associated factors, and improve the durability and antitumor efficacy of TILs [487]. Furthermore, inhibition of DNA methyltransferases or histone deacetylases can also enhance T cell function to a certain extent [488, 489], offering a viable strategy to maintain the long‐term activity of TILs during ex vivo expansion and post‐infusion persistence.

The combination of ICIs with TIL therapy has also demonstrated significant advantages. In a TNBC mouse model, TIL therapy combined with PD‐1 inhibitor treatment significantly increases the secretion of IFN‐γ and TNF‐α, enhances tumor infiltration by activated T cells, and downregulates inhibitory markers such as PD‐1, thereby effectively alleviating T cell exhaustion and achieving tumor clearance [490]. Radiotherapy induces immunogenic cell death, promotes the release of tumor antigens and danger signals, and creates an immunological window for TIL activation, thereby enhancing the local antitumor effect [470]. Furthermore, spermidine‐induced restoration of autophagy reduces the expression of inhibitory receptors, increases TCR diversity, and significantly enhances the proliferative capacity and cytotoxic activity of TILs, offering a novel metabolic regulatory strategy to reverse T cell exhaustion [491].

#### 5.2.3 TCR‐T Therapy

TCR‐T therapy involves introducing the TCR gene encoding a specific tumor antigen into the patient's T cells, enabling them to accurately recognize and eliminate antigen‐expressing tumor cells. Unlike CAR‐T cells, which target surface antigens in an MHC‐independent manner, TCR‐T cells recognize peptide antigens derived from intracellular protein processing through MHC molecule presentation, thereby expanding their applicability to a broad range of endogenous tumor antigens. Existing studies have demonstrated that TCR‐T cells hold significant potential for enhancing antitumor immunity, reversing T cell exhaustion, and serving as a component of combination immunotherapy. However, their clinical efficacy remains limited by MHC restriction, tumor immune escape mechanisms, and immunosuppression within the TME. Therefore, strategies such as optimizing TCR affinity, alleviating T cell exhaustion, and combining TCR‐T therapy with ICIs or cytokine‐based interventions have become key approaches to improving its antitumor efficacy.

Studies have shown that a multi‐engineered T cell vaccine (Multi‐Tvac) can activate TCR‐T cells and alleviate their exhaustion by promoting DC maturation and enhancing tumor antigen presentation in secondary lymphoid organs. In a solid tumor model, the combined use of Multi‐Tvac and TCR‐T cell therapy prevented immune escape due to antigen loss and achieved complete tumor regression, highlighting the critical role of optimized antigen presentation in overcoming TCR‐T cell exhaustion [492].

In recent years, combination immunotherapy has been recognized as a key strategy to alleviate T cell exhaustion and enhance the efficacy of cellular immunotherapy [365]. For example, EBV‐positive tumors recruit mononuclear‐macrophages via CCL5 to secrete CSF1 and IL‐10, thereby inducing CD163^+^ M2 macrophage polarization. These M2 macrophages secrete high levels of MMP9, leading to TIL dysfunction and resistance to TCR‐T therapy. Inhibition of MMP9 not only restores T cell function but also significantly suppresses the growth of EBV‐positive solid tumors when combined with TCR‐T treatment [493]. Additionally, Akt1 and Akt2 isoforms promote terminal differentiation of CD8^+^ T cells, thereby restricting the formation of central memory T cells (T_CM_). Selective inhibition of these two isoforms delays T cell exhaustion and preserves naïve and T_CM_ populations, thus enhancing the proliferation, survival, and effector function of CD8^+^ T cells [494].

Moreover, studies have shown that linoleic acid (LA) promotes the formation of endoplasmic reticulum‐mitochondrial contact sites (MERCs), enhances calcium signaling and mitochondrial energy metabolism, improves the metabolic fitness of CD8^+^ T cells, prevents their exhaustion, promotes a memory‐like effector phenotype, and significantly enhances antitumor function [495]. Andrew Nguyen et al. found that MS‐275, a histone deacetylase inhibitor, reverses tumor burden‐induced immunosuppression, reduces exhaustion‐associated features, enhances IL‐2–STAT5 signaling pathway activity, and promotes the differentiation of CD8^+^ T cells into terminally differentiated effector cells, thereby improving durable regression in adoptive T cell therapy [496]. In a combinatorial TCR and CAR strategy, GPC3‐HLA‐A2 transgenic mice were used to screen efficient TCRs, which were then engineered into TCR‐T cells. Combined administration of ACT and CAR‐T cells enhances the proliferation, effector function, and resistance to exhaustion of TCR‐T cells, thereby significantly improving the antitumor effect in HCC [497].

TCR‐T cell therapy has demonstrated unique targeting advantages and broad clinical potential in cancer treatment. Optimizing antigen presentation, metabolic regulation, signaling pathway modulation, and combining TCR‐T therapy with ICIs, cytokine‐based interventions, or other cell engineering approaches hold promise for significantly alleviating T cell exhaustion, enhancing long‐term antitumor immunity, and overcoming immune escape. In the future, multi‐dimensional combination strategies will enable more precise and effective TCR‐T–based immunotherapies for solid tumors.

### 5.3 OVs

OVs are self‐replicating viruses that selectively target and infect tumor cells, replicate extensively, and induce tumor cell lysis. Recent studies have demonstrated that, in addition to their direct tumoricidal activity, OVs can modulate the host immune response via their distinct structural features and mechanisms of action. This immunomodulatory capacity helps alleviate T cell exhaustion and enhances antitumor immunity.

#### 5.3.1 Engineered OVs as a Platform for Delivering Immunomodulatory Factors

In recent years, a range of OVs have been developed for the localized or systemic delivery of immunomodulatory agents aimed at improving the TIME and mitigating T cell exhaustion. The tumor‐selective herpes simplex virus (HSV) vector, engineered by Fei Ju et al. to encode a human PD‐1 single‐chain variable fragment (hPD‐1scFv), enables targeted release of a PD‐1 inhibitor within the tumor microenvironment, thereby significantly enhancing CD8^+^ T cell infiltration and activation, reducing T cell exhaustion markers, and promoting antitumor immune responses [392]. Additionally, conditionally replicating adenoviruses (CRAds) genetically modified to express immune‐stimulatory transgenes mCD40L or m4‐1BBL have demonstrated efficacy in a murine melanoma model, where they delayed tumor progression, extended survival, and enhanced immune cell infiltration: m4‐1BBL primarily promotes CD8^+^ T cell recruitment, whereas mCD40L facilitates CD4^+^ T cell infiltration, and their combined action leads to a synergistic enhancement of anti‐tumor immunity [498].

The engineered adenovirus TILT‐322, which expresses an aMUC1×aCD3 T cell engager and IL‐2, has been shown to activate γδ T cells and various immune cell populations, elevate levels of granzyme B, perforin, and IFN‐γ, and reduce the proportion of exhausted CD8^+^ T cells in ovarian cancer ascites. These effects contribute to enhanced T cell functionality and improved tumor control [499]. Similarly, TILT‐517 expressing IL‐7 enhances T cell and NK cell activity and promotes remodeling of the TME in the renal cell carcinoma (RCC) model [500]. VSV‐IFNβ exerts direct oncolytic effects, activates innate immunity, and enhances CD8^+^ T cell responses through sustained IFNβ expression, while simultaneously reducing the frequency of Tregs. When combined with ICIs, VSV‐IFNβ helps preserve the functionality of tumor‐specific T cells and prevents their exhaustion [501]. Pexa‐Vec can convert the TME from an immunologically “cold” to a “hot” state by inducing interferon signaling and pro‐inflammatory cytokine production, thereby promoting CTL infiltration and activation [502].

In addition, mesenchymal stem cell‐mediated delivery of oncolytic adenovirus (OAd‐MSCs) has been shown to increase the proportions of TAMs, natural killer cells, and TILs, while reducing the frequency of PD‐1^+^ TILs, thereby partially reversing T cell exhaustion [503]. The oncolytic adenovirus Ad5/3Δ24‐GM‐CSF was demonstrated to significantly decrease TIM‐3^+^ CD8^+^ TILs and improve the intratumoral immune microenvironment [504]. Therefore, engineered OVs not only mediate antitumor effects through direct oncolysis but also contribute to the reversal of T cell exhaustion and enhancement of antitumor immune responses via immunomodulation.

#### 5.3.2 Combination Immunotherapy Involving OVs

Notably, OVs possess the dual capabilities of directly lysing tumor cells and modulating the immune microenvironment. When combined with immunotherapy, OVs can mitigate T cell exhaustion and augment anti‐tumor immune responses. In recent years, accumulating evidence has demonstrated the potential of OVs in reversing T cell dysfunction. For instance, an oncolytic virus expressing OX40L (OV‐mOX40L) administered in PDAC models has been shown to enhance CD4^+^ T cell activation, reduce Treg infiltration, and alleviate CTL exhaustion. Therapeutic efficacy is further enhanced when this approach is combined with anti‐IL‐6 and anti‐PD‐1 therapies, offering a promising multimodal strategy for PDAC [505]. Additionally, NKG2A blockade in combination with the KISIMA‐VSV‐GP‐TAg vaccine significantly reduced CD8^+^ T cell exhaustion within the tumor microenvironment, improved local T cell functionality, prolonged survival in murine models, and promoted the development of effector memory CD8^+^ T cells [506].

Furthermore, the combination of oncolytic adenovirus expressing interleukin‐7 (oAD‐IL7) with B7‐H3‐targeted CAR‐T cells has been shown to significantly enhance T cell proliferation and survival, as well as improve the persistence of tumor‐infiltrating CAR‐T cells [507]. Additionally, OAd‐MSCs combined with granulocyte colony‐stimulating factor (G‐CSF) have been shown to increase TIL counts, reduce T cell exhaustion, and suppress tumor growth in murine osteosarcoma models [508].

The development of multifunctional and multi‐target OVs therapy strategies offers novel insights into mitigating T cell exhaustion. Recombinant vaccinia virus encoding an EpCAM‐targeting bispecific T cell engager (VV‐EpCAM BiTE) is capable of infecting and lysing tumor cells, thereby releasing the BiTE molecule, which facilitates the formation of a bridge between tumor cells and T cells, activates naïve T cells, and enhances cytokine production. Intratumoral administration of this construct has been shown to significantly increase immune cell infiltration, reduce CD8^+^ T cell exhaustion, and augment anti‐tumor immune responses across various solid tumors [509]. Additionally, the virus‐like vesicle (VLV) vector CARG‐2020 enables co‐delivery of single‐chain IL‐12, PD‐L1‐targeted shRNA, and a dominant‐negative mutant of IL‐17RA, leading to sustained immune memory and systemic anti‐tumor effects through the activation of Th1 immunity and suppression of genes associated with T cell exhaustion and pro‐tumorigenic inflammation [510].

### 5.4 Cancer Vaccines

Cancer vaccines aim to reverse T cell exhaustion by reactivating antitumor T‐cell responses through the delivery of tumor‐specific antigens. However, within the profoundly immunosuppressive tumor microenvironment, antigen stimulation alone often yields limited efficacy and may even aggravate T cell exhaustion. Consequently, recent strategies have focused on combining cancer vaccines with immunomodulatory approaches to enhance their effectiveness. These include blocking inhibitory immune checkpoints, remodeling the tumor microenvironment, and promoting the generation of memory T cells. For instance, integrating vaccines with small‐molecule inhibitors—such as those targeting the JAK‐STAT pathway or bromodomain proteins—has been shown to augment T cell activation, mitigate exhaustion, and facilitate tumor infiltration [511, 512]. Additionally, when combined with adoptive cell therapies, such as CAR‐T or TILtransfer, vaccines enhance effector T cell expansion, persistence, and antigen‐specific immune responses [513, 514].

Collectively, these integrated strategies demonstrate the potential of cancer vaccines to reverse T cell exhaustion and enhance the effectiveness of immunotherapy. To this end, we summarize the relevant studies and underlying mechanisms (Table 4 **)**.

**TABLE 4:** Cancer vaccine reverses T cell exhaustion: mechanisms and combined strategies.

Vaccine type	Combination therapy	Strategy	Type of cancer	Result	References
DC vaccine	Radiotherapy / ICB	SnC/DC‐STING pathway	/	Promote DC maturation, activate CD8^+^ CTL	[515]
	/	OCDC (autologous DC + tumor lysate)	Ovarian cancer	Induce multi‐epitope T cell expansion, relieve T cell exhaustion	[516]
	/	TYMP+ DC activation	Colorectal cancer	Reduce Treg/PD‐L1, reverse exhaustion, enhance CTL infiltration	[517]
	/	Spatiotemporal nanoregulator (DNR, TLR4/TLR7/8 synergy)	/	Optimize TDLN DC function, inhibit immune checkpoints, and alleviate exhaustion	[518]
	ICB	Target PD‐L1^+^ TAM	/	Block TAM‐mediated immunosuppression, reverse exhaustion	[519]
	CD40 agonist	Syngeneic BM‐derived DC loaded with KPC pancreatic cancer or AE17 mesothelioma tumor lysates	Pancreatic cancer	Induce tumor‐specific T cell responses, promote CD8^+^ T cell proliferation and activation, and enable cross‐recognition of pancreatic cancer antigens	[520]
Nucleic acid vaccine	/	circH19‐vac	GBM	Activate CTL responses and inhibit MDSC/TAM recruitment	[521]
	ICB	C2@mLMP2(LMP2‐mRNA LNP) – Targeting EBV	Nasopharyngeal carcinoma (NPC)	Induce CD8^+^ memory T cell expansion and reverse exhaustion	[522]
	ICB	HPV mRNA‐LNP (E7 antigen)	Cervical cancer	Activate CD8^+^ T cell function in spleen and the TME, modulate exhaustion trajectory	[523]
Tumor cell vaccine	/	AGI‐101H (genetically modified allogeneic melanoma vaccine)	Melanoma	Activate TNF‐α/TGF‐β, inhibit IL2‐STAT5, upregulate BCL6, suppress exhaustion markers	[524]
	ICB	Whole tumor cell vaccine (intracellular hydrogel + CD47 blockade + DAMP exposure)	/	Enhance immunogenicity, activate CD4^+^ T cells toward Th1 polarization, and induce tumor antigen‐specific T cell responses	[525]
	ICB	SA‐GM‐CSF vaccine + anti‐PD‐1 + anti‐Tim‐3 triple therapy	/	Increase tumor regression, reduce CD8^+^ TIL apoptosis, and reverse exhaustion	[526]
Vector vaccine	/	Lentivirus + CD40L + soluble PD‐1 microparticle	/	Promote DC maturation, block immune checkpoints, and induce antigen‐specific CD8^+^ T cell expansion	[527]
	Combined with TriVax vaccine	Retroviral + STAT5 + TriVax vaccine	Melanoma	Enhance T cell proliferation and multifunctionality, reduce PD‐1, and partially block the PD‐1/PD‐L1 pathway	[528]

Abbreviations: CTL, cytotoxic T lymphocyte; DAMP, damage‐associated molecular pattern; DC, dendritic cell; EBV, epstein–barr virus; GBM, glioblastoma; HPV, human papillomavirus; ICB, immune checkpoint blockade; MDSC, myeloid‐derived suppressor cell; NPC, nasopharyngeal carcinoma; TAM, tumor‐associated macrophage; TIL, tumor‐infiltrating lymphocyte.

It is noteworthy that the vaccine delivery strategy constitutes a critical factor in enhancing therapeutic efficacy and counteracting T cell exhaustion. Evidence from preclinical studies indicates that intravenous administration demonstrates superior efficacy compared to subcutaneous injection in generating ICIs‐responsive T_PEX_ cells in MC38 tumor‐bearing mice, leading to improved tumor control [529]. Furthermore, targeted delivery of vaccines to lymph nodes via amphiphilic vectors has been shown to optimize DC function within lymphoid tissues and potentiate antitumor immune responses [530, 531]. Additionally, the selection of adjuvants can modulate the expression of costimulatory molecules and cytokines on DCs, thereby influencing the differentiation and functional fate of CD8^+^ T cells [532].

### 5.5 Costimulatory Agonists

Depleted T cells often lose critical costimulatory signals (e.g., OX40, 4‐1BB, GITR, CD40), compromising their functions and reducing the effectiveness of ICIs. Agonists targeting these receptors act as “functional restoration switches” delivering survival and effector signals to T_EX_ cells and restoring or enhancing their antitumor capabilities. Costimulatory agonists, when used alone or in combination with ICIs and other immunotherapies, have demonstrated potential in delaying or reversing T cell exhaustion (Table 5).

**TABLE 5:** Co‐stimulatory receptor agonists in reversing T cell exhaustion: Mechanisms and combination therapies.

Co‐stimulatory receptor	Combination therapy	Mechanism	Type of cancer	References
4‐1BB	ICB	Reduce TIL exhaustion, improve TIL function, and significantly prolong survival	GBM	[533]
	ICB	High 4‐1BB expression on PD‐1^hi^ CD39^+^ CD8 TILs promotes T cell activation and proliferation, reduces exhaustion, and 4‐1BB agonists enhance anti‐PD‐1–mediated functional restoration	Ovarian cancer	[534]
	/	Enhance CD4/CD8 T cell cytotoxicity and promote tumor clearance	/	[535]
	ICB+IL15C	Promote CD8^+^ T cell clonal expansion and induce exhaustion features; addition of IL‐15C reverses T cell exhaustion and enhances effector and memory differentiation	PDAC	[536]
OX40	TLR9 agonist	Enhance CD8^+^ T cell proliferation and cytokine secretion, inhibit Treg, promote memory T cell generation, and induce systemic antitumor immunity	BC	[537]
	ICB	Sequential administration significantly enhances efficacy and promotes CD4^+^ and CD8^+^ T cell functionality	BC	[538]
GITR	/	Enhance effector T cell function and activate antitumor immune responses.	/	[539]
	/	Agonize GITR to deplete exhausted Tregs, restore CD8^+^ T cell function, and downregulate exhaustion markers such as PD‐1 and LAG‐3	/	[540]
	Anti–Gal‐9	Reverse Treg‐mediated suppression, enhance CD8^+^ TIL antitumor activity	Solid tumors	[301]
CD40	TNFR2 antagonist	Selectively deplete activated tumor‐infiltrating Tregs and alleviate CD8^+^ T cell exhaustion	PDAC	[541]
	Local radiotherapy	Enhances CD40 agonist efficacy, induces tumor regression, and generates durable Tmem responses	Pancreatic cancer	[542]
CD27	ICB	Provide help signals to promote functional CTL generation	/	[543]
CD28	ICB/CD40L	Restore APC activity to enhance the antitumor function of exhausted CD8^+^ TILs	Ovarian cancer	[544]
CD27/CD28	ICB	Enhances cytokine (TNF‐α, IFN‐γ) production and improves T_RM_ cell function	NSCLC	[545]

Abbreviations: APC, antigen‐presenting cell; BC, breast cancer; CTL, cytotoxic T lymphocyte; GBM, glioblastoma; ICB, immune checkpoint blockade; NSCLC, non‐small cell lung cancer; PDAC, pancreatic ductal adenocarcinoma; TIL, tumor‐infiltrating lymphocyte; T_RM_, tissue‐resident memory T cell.

Although costimulatory agonists may pose clinical risks such as cytokine release syndrome, autoimmune responses, and thrombosis [546, 547], they show significant potential as a “functional recovery switch” in combination immunotherapy. Nevertheless, their safety profile, optimal dosage, and administration regimen require further optimization and validation.

### 5.6 Bispecific Antibody

BsAbs are next‐generation immunotherapies that can simultaneously target two distinct antigens, making them highly promising anticancer agents. By bridging T cells to tumor‐associated antigens or modulating two immune signaling pathways, BsAbs can activate effector T cells, alleviate immunosuppression, reprogram the function of T_EX_ cells, and restore their proliferation and cytotoxicity. Notably, BsAbs have shown substantial clinical potential in hematological malignancies and some solid tumors, offering a novel strategy to reverse T cell exhaustion and enhance cancer immunotherapy [548].

One of the key contributors to T cell exhaustion is the persistent presence of inhibitory immune signals, particularly through sustained activation of immune checkpoint receptors such as CTLA‐4 and PD‐1. BsAbs demonstrate distinct therapeutic potential in this context by simultaneously targeting tumor‐associated antigens and inhibitory receptors, or by engaging two immune regulatory molecules concurrently, thereby directly suppressing exhaustion‐related signaling pathways and restoring T‐cell functionality. For instance, MGD019, a bispecific antibody that co‐blocks PD‐1 and CTLA‐4, enables localized modulation of Tregs within the TME while preserving systemic anti‐CTLA‐4 activity, thus enhancing both safety and antitumor efficacy [549]. Similarly, in patients with B‐cell non‐Hodgkin lymphoma (B‐NHL), the combination of BsAbs targeting CD39 or CD73 has been shown to augment T‐cell responses, particularly among individuals exhibiting a high frequency of PD‐1^+^ TIM‐3^+^ T cells [345].

T_EX_ cells exhibit not only diminished effector function but also a loss of costimulatory signaling, making durable immune reconstitution difficult to achieve through the mere blockade of inhibitory pathways. By incorporating costimulatory signals, BsAbs can promote the reactivation of T_EX_ cells and sustain their functional capacity, thereby enhancing tumor control. In multiple myeloma, BCMA‐targeted immunotherapies have significantly improved patient outcomes; however, the recurrence rate remains high. Next‐generation T cell–redirecting BsAbs, such as those targeting GPRC5D or FcRH5, have demonstrated clinical promise in patients who have relapsed after prior treatments. Trispecific antibodies (TsAbs), which integrate costimulatory signals while simultaneously engaging two tumor antigens, offer a strategic advancement by mitigating both T cell exhaustion and antigen escape [550], thus providing a novel approach to overcoming the limitations of conventional ICB.

In addition, the immunosuppressive characteristics of the TME, such as upregulation of PD‐1/PD‐L1 during treatment, can limit the efficacy of BsAbs in solid tumors [551]. Combining ICB with BsAbs, including bispecific T‐cell engagers (BiTEs), has been shown to significantly restore and enhance T‐cell function. For instance, the CD33/CD3 bispecific antibody AMG 330 induces cytotoxic activity against AML cells, accompanied by concurrent upregulation of PD‐L1 expression. The blockade of PD‐1/PD‐L1 further enhances AMG 330‐mediated cell lysis, T‐cell proliferation, and interferon‐γ secretion [552]. In studies involving CEA‐targeting Bsabs, upregulation of PD‐1 and impaired effector T‐cell function have been observed; combining such agents with anti‐PD‐1/PD‐L1 therapy can augment tumor cell killing [553]. Although HER2‐TDB enhances the antitumor activity of anti‐HER2 therapies, it may also induce PD‐L1‐mediated resistance. The combination of HER2‐TDB with anti‐PD‐L1 treatment has been demonstrated to significantly improve antitumor efficacy and prolong response duration [554].

BsAbs have demonstrated considerable potential in reversing T cell exhaustion and enhancing antitumor immune responses by concurrently blocking inhibitory signals and activating costimulatory pathways, particularly in hematological malignancies and certain solid tumors. Nevertheless, challenges remain in the context of solid tumors, including immunosuppressive tumor microenvironments, suboptimal target selection, and immune‐related toxicities. Future research should focus on refining BsAb design, improving tumor‐specific delivery strategies, combining BsAbs with ICB or other immunotherapies to mitigate adverse effects, and utilizing biomarkers for patient stratification to enable personalized, precision‐based therapeutic approaches. A deeper understanding of the mechanisms underlying the response of T_EX_ cell subsets to various BsAb formats will further contribute to enhanced therapeutic efficacy and sustained anti‐tumor immunity.

### 5.7 Antibody–Drug Conjugates

ADCs are composed of a monoclonal antibody, a chemical linker, and a highly potent cytotoxic agent, representing a targeted therapeutic strategy in oncology. By selectively delivering cytotoxic payloads to tumor cells, ADCs achieve potent antitumor efficacy while minimizing systemic toxicity. In comparison with conventional chemotherapy, ADCs offer not only direct tumor cell killing but also potential immunomodulatory effects. Specifically, ADC‐induced tumor cell lysis through ICD leads to the release of tumor‐associated antigens and DAMPs, such as ATP and HMGB1. These molecules facilitate DC maturation and promote antigen‐specific T cell activation. Collectively, these immune‐mediated responses help overcome tumor immune tolerance, remodel the immunosuppressive microenvironment, and contribute to reversing T cell exhaustion.

Recent studies have demonstrated that a novel humanized AXL‐targeting antibody‐drug conjugate (AXL02‐MMAE) effectively suppresses tumor migration and EMT by selectively targeting tumor cells exhibiting high AXL expression as well as tumor‐associated M2 macrophages. This agent also attenuates the immunosuppressive TME and restores CTL function, leading to significant reversal of T cell exhaustion [555]. Similarly, ADCs incorporating pyrrolobenzodiazepine or tubulysin payloads have been shown to activate the immune system and enhance CD8^+^ T cell function through induction of ICD. These ADCs exhibit synergistic antitumor activity when combined with PD‐1/PD‐L1 checkpoint inhibitors or costimulatory agonists targeting OX40 and GITR pathways [556].

The cGAS‐STING pathway is activated upon recognition of foreign DNA released by necrotic tumor cells, leading to the production of type I interferons and subsequent activation of the innate immune system, thereby promoting T cell infiltration into the tumor microenvironment [557, 558, 559, 560]. Building on this mechanism, STING‐ADC enhances antitumor immunity while maintaining systemic tolerance through targeted delivery of STING agonists, and demonstrates synergistic efficacy when combined with PD‐L1 inhibitors, highlighting its potential for clinical translation [561]. For instance, XMT‐2056 utilizes HER2‐targeted delivery to administer a STING agonist, thereby inducing tumor regression via activation of innate immunity, reversal of immunosuppressive conditions, reduction of T cell exhaustion, and enhancement of T cell effector function [562]. Additionally, trastuzumab deruxtecan (T‐DXd) can induce immunogenic cell death by releasing cytotoxic payloads, activate myeloid cells, and augment the antitumor activity of CD8^+^ T cells. However, it may concurrently upregulate tumor expression of CD47 and contribute to T cell exhaustion. Combination with CD47 blockade can counteract this immunosuppressive effect, significantly improve the therapeutic efficacy of antibody‐drug conjugates, enhance CD8^+^ T cell memory responses, and reduce the risk of tumor recurrence [563].

ADCs not only exert direct tumoricidal effects but also reverse T cell exhaustion through the induction of immunogenic cell death, enhancement of antigen presentation, and remodeling of the tumor microenvironment. However, their immunoregulatory efficacy is limited by tumor heterogeneity, immunosuppressive features of the TME, and potential systemic toxicity. Future strategies to enhance immune reconstitution and anti‐tumor activity may include optimization of ADC design, combination with costimulatory signals or ICIs, augmentation of cGAS/STING pathway activation, and integration of biomarker‐driven personalized therapeutic approaches.

## 6 Challenges and Future Directions

T cell exhaustion resulting from persistent tumor antigen stimulation has emerged as one of the most significant biological barriers in cancer immunotherapy. Although ICB has demonstrated remarkable efficacy in reinvigorating T_EX_ cells, critical challenges persist, shaping key priorities for future research.

T cell exhaustion is a dynamic differentiation process with multiple distinct subsets, rather than a uniform cellular state. At the two extremes of this lineage are T_PEX_ cells, which possess self‐renewal and proliferative capacity, and T_EX‐term_ cell, which exhibit limited functionality. However, the origins and differentiation pathways of these subsets remain a subject of debate [122, 564]. Some studies propose that T_EX_ cells arise from conventional effector T cells, which progressively lose effector function under persistent antigen stimulation through upregulation of inhibitory receptors and gradual impairment of cytokine production, following a model of “continuous phenotypic evolution” [565]. In contrast, accumulating evidence indicates that T cells may commit to a distinct differentiation trajectory early during activation in the setting of chronic antigen exposure. Key transcription factors such as TOX are rapidly induced and drive extensive epigenetic reprogramming, establishing an exhaustion‐specific chromatin landscape that actively directs T cell fate toward exhaustion rather than representing mere functional decline [316]. Heterogeneity in the TME—such as differences in tumor type, antigen burden, and metabolic conditions—futher complicates differentiation pathways and increases therapeutic uncertainty [566, 567]. However, traditional analytical approaches are limited in their ability to fully capture these complex divergence trajectories and spatial distributions. The integration of single‐cell multi‐omics technologies—such as scRNA‐seq, ATAC‐seq, and single‐cell T cell receptor sequencing (scTCR‐seq)—with spatial profiling methods, including spatial transcriptomics, has overcome this technical limitation. This integrated approach enables the simultaneous analysis of transcriptional, epigenetic, and spatial localization data at single‐cell resolution, thereby allowing for a precise characterization of the differentiation trajectories, spatial organization, and interactions of T_EX_ cells within the tumor microenvironment [123, 568, 569, 570, 571].

A major challenge is the epigenetic basis of T cell exhaustion. Upon entering the exhausted state, T cells undergo extensive epigenetic remodeling, characterized by alterations in chromatin accessibility, DNA methylation, and histone modifications—collectively forming an “epigenetic scar” [24]. This epigenetic stabilization renders the exhausted phenotype persistent even after antigen removal, which explains why ICB therapies, such as PD‐1 inhibition, often fail to fully reverse T_EX‐term_ cells. Instead, therapeutic efficacy primarily depends on the expansion of residual TCF1^+^ progenitor‐like cells [572, 573]. Thus, safely and effectively reprogramming the epigenetic landscape of T_EX_ cells remains a critical hurdle in immunotherapy. Although direct ablation of exhaustion‐associated genes such as TOX and Tim‐3 can transiently restore T cell function, it frequently results in rapid T cell exhaustion or severe immunopathological consequences, underscoring the essential biological role of the exhaustion program in preserving T cell longevity and systemic homeostasis [297]. Novel approaches—including low‐dose epigenetic modulators (e.g., DNMT3A or HDAC inhibitors) combined with ICB, as well as CRISPR‐Cas9‐mediated targeted epigenome editing—are currently under investigation in clinical settings [24, 574, 575]. Moreover, T cell exhaustion is not limited to transcriptional and epigenetic dysregulation but also involves significant disruptions at the translational level [576]. Recent evidence indicates that tumor‐infiltrating T cells adopt an aberrant “hypertranslation” state during exhaustion. In particular, the RNA‐binding protein LARP4 enhances the translational efficiency of nuclear‐encoded OXPHOS mRNAs, leading to mitochondrial translational imbalance and accumulation of ROS, thereby accelerating T cell dysfunction [577]. Targeting this hyperactive translation state may offer a promising strategy for reversing T cell exhaustion; however, research in this area remains in its early stages.

In addressing the complex challenge of T cell exhaustion, a fundamental paradigm shift in therapeutic strategy is required—from attempting to reverse terminally exhausted states toward early intervention and fate regulation. Rather than pursuing the impractical goal of fully reversing terminal T cell exhaustion, greater emphasis should be placed on preserving the population of T_PEX_ cells with stem‐like properties, delaying their differentiation into terminal states, and integrating strategies for remodeling the TME to enhance T cell functionality and persistence. For instance, inhibition of suppressive signals such as TGF‐β and IL‐10, or provision of supportive cytokines including CD28 costimulatory signals and IL‐21, has been shown to effectively delay the exhaustion process [578, 579, 580, 581]. Notably, T cell exhaustion should not be viewed as an isolated dysfunction of CD8^+^ T cells, but rather as a component of a complex, multicellular immune network. The impairment of CD4^+^ T cells results in the loss of critical helper signals, thereby accelerating the functional decline of CD8^+^ T cells [41, 582]. Furthermore, dysregulation of Tregs during chronic inflammation may transiently reduce immunosuppressive activity, yet over time leads to disruption of immune homeostasis and increased susceptibility to autoimmune pathology [583]. Consequently, any effective therapeutic approach must account for these intricate intercellular interactions within the immune system.

Innovative technologies are advancing the field at an unprecedented pace. Emerging technologies such as CRISPR‐Cas9 gene editing enable precise modulation of exhaustion‐related pathways (e.g., NR4A, TOX), offering new avenues for durable T cell rejuvenation. Recent studies have demonstrated that deletion of the three NR4A family genes (NR4A1, NR4A2, and NR4A3) in CAR‐T cells significantly enhances their durable cytotoxic capacity against solid tumors and improves mitochondrial metabolic adaptability, with consistent efficacy observed across multiple donor‐derived T cell populations [584]. Similarly, knockdown of TOX downregulates the expression of multiple immune checkpoint molecules, delays the progression of T cell exhaustion, and has been associated with improved prediction of immunotherapy response [335]. Synthetic biology enables T cells to sense and self‐regulate exhaustion signals through the construction of artificial genetic circuits. Recent evidence shows that T cells can be reprogrammed into an atypical exhausted state via orthogonal cytokine engineering—such as expression of IL‐2 variants, IL‐33, or PD‐1 decoy receptors—which effectively suppresses TOX expression and enhances T cell persistence and antitumor efficacy in solid tumor models [160, 585]. Furthermore, logic‐gated systems based on AND, OR, and NOT Boolean circuits have been employed to precisely control T cell responses to tumor microenvironment signals, thereby reducing off‐target toxicity while preserving effector function [586, 587]. In addition, advances in nanotechnology and delivery systems have enabled the development of efficient and precise therapeutic platforms; for instance, lipid nanoparticle (LNP)‐based mRNA delivery systems allow localized and controlled expression of the cytokine IL‐12, minimizing systemic toxicity while enhancing T cell function [368]. However, it must be emphasized that the efficient and specific delivery of therapeutic agents to target cells within the tumor microenvironment, while minimizing off‐target effects, remains a major barrier to clinical translation. Future strategies are likely to rely on the development of intelligent, stimuli‐responsive delivery systems—such as nanomaterial‐based drug carriers sensitive to the unique pH, enzymatic activity, or redox conditions of the TME—or the engineering of viral and non‐viral vectors designed to recognize specific T cell surface antigens, enabling controlled in vivo drug release [588, 589]. Interestingly, biomimetic physical barriers can transiently block immunosuppressive interactions between T cells and tumor cells, creating a “protective microenvironment” that promotes T cell accumulation and functional preservation, with restored cytotoxic activity upon timely release [590]. A deeper understanding of the mechanisms underlying T cell exhaustion offers promising avenues for therapies that precisely modulate this state, enabling the immune system to mount robust and sustained antitumor responses—ultimately leading to durable, safe, and effective cancer immunotherapy.

## 7 Conclusion

T cell exhaustion represents a central mechanism underlying tumor immune escape. Because of its multi‐layered and dynamic nature, single therapeutic interventions are unlikely to yield significant breakthroughs. Future research should build upon a comprehensive understanding of the heterogeneity of T_EX_ cells, epigenetic regulation, and metabolism‐translation coupling networks to develop multi‐targeted, temporally controlled combination strategies. Integrating interdisciplinary approaches from immunology, epigenetics, synthetic biology, and nanomaterials science may enable precise reprogramming of T cell states. This holds the potential to stabilize stem‐like T cell subpopulations, inhibit terminal differentiation, remodel the TIME, and ultimately enable durable and safe anti‐tumor immunity. Achieving this goal requires both innovative scientific insights and rigorous translational and clinical validation.

## Conflicts of Interest

The authors declare no conflicts of interest.

## References

[1] X. He and C. Xu , “Immune Checkpoint Signaling and Cancer Immunotherapy,” Cell Research 30, no. 8 (2020): 660–669, 10.1038/s41422-020-0343-4. 32467592 PMC7395714

[2] M. M. Gubin , X. Zhang , H. Schuster , et al., “Checkpoint Blockade Cancer Immunotherapy Targets Tumour‐specific Mutant Antigens,” Nature 515, no. 7528 (2014): 577, 10.1038/nature13988. 25428507 PMC4279952

[3] A. Chow , K. Perica , C. A. Klebanoff , and J. D. Wolchok , “Clinical Implications of T Cell Exhaustion for Cancer Immunotherapy,” Nature Reviews Clinical Oncology 19, no. 12 (2022): 775–790, 10.1038/s41571-022-00689-z. PMC10984554 36216928

[4] D. Kasakovski , L. Xu , and Y. Li , “T Cell Senescence and CAR‐T Cell Exhaustion in Hematological Malignancies,” Journal of Hematology & Oncology 11, no. 1 (2018): 91, 10.1186/s13045-018-0629-x. 29973238 PMC6032767

[5] A. Baessler and D. A. A. Vignali , “T Cell Exhaustion,” Annual Review of Immunology 42, no. 1 (2024): 179–206, 10.1146/annurev-immunol-090222-110914. 38166256

[6] C. U. Blank , W. N. Haining , W. Held , et al., “Defining ‘T Cell Exhaustion’,” Nature Reviews Immunology 19, no. 11 (2019): 665–674, 10.1038/s41577-019-0221-9. PMC7286441 31570879

[7] C. Tsui , L. Kretschmer , S. Rapelius , et al., “MYB Orchestrates T Cell Exhaustion and Response to Checkpoint Inhibition,” Nature 609, no. 7926 (2022): 354–360, 10.1038/s41586-022-05105-1. 35978192 PMC9452299

[8] O. Khan , J. R. Giles , S. McDonald , et al., “TOX Transcriptionally and Epigenetically Programs CD8+ T Cell Exhaustion,” Nature 571, no. 7764 (2019): 211–218, 10.1038/s41586-019-1325-x. 31207603 PMC6713202

[9] Z. Chen , Z. Ji , S. F. Ngiow , et al., “TCF‐1‐Centered Transcriptional Network Drives an Effector versus Exhausted CD8 T Cell‐Fate Decision,” Immunity 51, no. 5 (2019): 840–855, 10.1016/j.immuni.2019.09.013. 31606264 PMC6943829

[10] H. Seo , J. Chen , E. Gonzalez‐Avalos , et al., “TOX and TOX2 Transcription Factors Cooperate with NR4A Transcription Factors to Impose CD8 + T Cell Exhaustion,” Proceedings of the National Academy of Sciences 116, no. 25 (2019): 12410, 10.1073/pnas.1905675116. PMC6589758 31152140

[11] J. A. Belk , B. Daniel , and A. T. Satpathy , “Epigenetic Regulation of T Cell Exhaustion,” Nature Immunology 23, no. 6 (2022): 848–860, 10.1038/s41590-022-01224-z. 35624210 PMC10439681

[12] F. Franco , A. Jaccard , P. Romero , Y. R. Yu , and P. C. Ho , “Metabolic and Epigenetic Regulation of T‐Cell Exhaustion,” Nature Metabolism 2, no. 10 (2020): 1001–1012, 10.1038/s42255-020-00280-9. 32958939

[13] G. Soto‐Heredero , G. Desdin‐Mico , and M. Mittelbrunn , “Mitochondrial Dysfunction Defines T Cell Exhaustion,” Cell Metabolism 33, no. 3 (2021): 470–472, 10.1016/j.cmet.2021.02.010. 33657392

[14] N. E. Scharping , D. B. Rivadeneira , A. V. Menk , et al., “Mitochondrial Stress Induced by Continuous Stimulation Under Hypoxia Rapidly Drives T Cell Exhaustion,” Nature Immunology 22, no. 2 (2021): 205–215, 10.1038/s41590-020-00834-9. 33398183 PMC7971090

[15] D. V. Sawant , H. Yano , M. Chikina , et al., “Adaptive Plasticity of IL‐10+ and IL‐35+ Treg Cells Cooperatively Promotes Tumor T Cell Exhaustion,” Nature Immunology 20, no. 6 (2019): 724–735, 10.1038/s41590-019-0346-9. 30936494 PMC6531353

[16] F. Xie , X. Zhou , P. Su , et al., “Breast Cancer Cell‐Derived Extracellular Vesicles Promote CD8+ T Cell Exhaustion via TGF‐β Type II Receptor Signaling,” Nature Communications 13, no. 1 (2022): 4461, 10.1038/s41467-022-31250-2. PMC9343611 35915084

[17] Y. Wang , A. Ma , N. J. Song , et al., “Proteotoxic Stress Response Drives T Cell Exhaustion and Immune Evasion,” Nature 647 (2025): 1025–1035. 41034580 10.1038/s41586-025-09539-1 PMC12657239

[18] H. Kissick and R. Ahmed , “New Epigenetic Regulators of T Cell Exhaustion,” Cancer Cell 40, no. 7 (2022): 708–710, 10.1016/j.ccell.2022.06.008. 35820395

[19] H. E. Ghoneim , Y. Fan , A. Moustaki , et al., “De Novo Epigenetic Programs Inhibit PD‐1 Blockade‐Mediated T Cell Rejuvenation,” Cell 170, no. 1 (2017): 142–157, 10.1016/j.cell.2017.06.007. 28648661 PMC5568784

[20] M. H. Hung , J. S. Lee , C. Ma , et al., “Tumor Methionine Metabolism Drives T‐Cell Exhaustion in Hepatocellular Carcinoma,” Nature Communications 12, no. 1 (2021): 1455, 10.1038/s41467-021-21804-1. PMC7935900 33674593

[21] J. Li , H. H. Huang , B. Tu , et al., “Reversal of the CD8+ T‐Cell Exhaustion Induced by Chronic HIV‐1 Infection Through Combined Blockade of the Adenosine and PD‐1 Pathways,” Frontiers in Immunology 12 (2021): 687296, 10.3389/fimmu.2021.687296. 34177939 PMC8222537

[22] M. Liu , Z. Li , W. Yao , et al., “IDO Inhibitor Synergized with Radiotherapy to Delay Tumor Growth by Reversing T Cell Exhaustion,” Mol Med Rep 21, no. 1 (2020): 445–453. 31746428 10.3892/mmr.2019.10816

[23] D. Xiong , H. Yu , and Z. J. Sun , “Unlocking T Cell Exhaustion: Insights and Implications for CAR‐T Cell Therapy,” Acta Pharmaceutica Sinica B 14, no. 8 (2024): 3416, 10.1016/j.apsb.2024.04.022. 39220881 PMC11365448

[24] T. Ahn , E. A. Bae , and H. Seo , “Decoding and Overcoming T Cell Exhaustion: Epigenetic and Transcriptional Dynamics in CAR‐T Cells Against Solid Tumors,” Molecular Therapy 32, no. 6 (2024): 1617–1627, 10.1016/j.ymthe.2024.04.004. 38582965 PMC11184340

[25] X. Wang , X. Tao , P. Chen , et al., “MEK Inhibition Prevents CAR‐T Cell Exhaustion and Differentiation via Downregulation of c‐Fos and JunB,” Signal Transduction and Targeted Therapy 9, no. 1 (2024): 293, 10.1038/s41392-024-01986-y. 39438476 PMC11496645

[26] K. Minton , “Overcoming CAR T Cell Exhaustion,” Nature Reviews Immunology 20, no. 2 (2020): 72–73, 10.1038/s41577-019-0265-x. 31844329

[27] A. Gallimore , A. Glithero , A. Godkin , et al., “Induction and Exhaustion of Lymphocytic Choriomeningitis Virus–Specific Cytotoxic T Lymphocytes Visualized Using Soluble Tetrameric Major Histocompatibility Complex Class I–Peptide Complexes,” The Journal of Experimental Medicine 187, no. 9 (1998): 1383–1393, 10.1084/jem.187.9.1383. 9565631 PMC2212278

[28] A. J. Zajac , J. N. Blattman , K. Murali‐Krishna , et al., “Viral Immune Evasion due to Persistence of Activated T Cells without Effector Function,” The Journal of Experimental Medicine 188, no. 12 (1998): 2205–2213, 10.1084/jem.188.12.2205. 9858507 PMC2212420

[29] C. Boni , P. Fisicaro , C. Valdatta , et al., “Characterization of Hepatitis B Virus (HBV)‐Specific T‐Cell Dysfunction in Chronic HBV Infection,” Journal of Virology 81, no. 8 (2007): 4215, 10.1128/JVI.02844-06. 17287266 PMC1866111

[30] C. L. Day , D. E. Kaufmann , P. Kiepiela , et al., “PD‐1 Expression on HIV‐specific T Cells Is Associated with T‐cell Exhaustion and Disease Progression,” Nature 443, no. 7109 (2006): 350–354, 10.1038/nature05115. 16921384

[31] H. Radziewicz , C. C. Ibegbu , M. L. Fernandez , et al., “Liver‐Infiltrating Lymphocytes in Chronic Human Hepatitis C Virus Infection Display an Exhausted Phenotype with High Levels of PD‐1 and Low Levels of CD127 Expression,” Journal of Virology 81, no. 6 (2007): 2545, 10.1128/JVI.02021-06. 17182670 PMC1865979

[32] S. Urbani , B. Amadei , D. Tola , et al., “PD‐1 Expression in Acute Hepatitis C Virus (HCV) Infection Is Associated with HCV‐Specific CD8 Exhaustion,” Journal of Virology 80, no. 22 (2006): 11398, 10.1128/JVI.01177-06. 16956940 PMC1642188

[33] C. Fenwick , V. Joo , P. Jacquier , et al., “T‐Cell Exhaustion in HIV Infection,” Immunological Reviews 292, no. 1 (2019): 149, 10.1111/imr.12823. 31883174 PMC7003858

[34] J. Shi , S. Hou , Q. Fang , X. Liu , X. Liu , and H. Qi , “PD‐1 Controls Follicular T Helper Cell Positioning and Function,” Immunity 49, no. 2 (2018): 264–274, 10.1016/j.immuni.2018.06.012. 30076099 PMC6104813

[35] G. J. Freeman , E. J. Wherry , R. Ahmed , and A. H. Sharpe , “Reinvigorating Exhausted HIV‐Specific T Cells via PD‐1–PD‐1 Ligand Blockade,” The Journal of Experimental Medicine 203, no. 10 (2006): 2223–2227, 10.1084/jem.20061800. 17000870 PMC2118103

[36] D. L. Barber , E. J. Wherry , D. Masopust , et al., “Restoring Function in Exhausted CD8 T Cells During Chronic Viral Infection,” Nature 439, no. 7077 (2006): 682–687, 10.1038/nature04444. 16382236

[37] E. J. Wherry , “T Cell Exhaustion,” Nature Immunology 12, no. 6 (2011): 492–499, 10.1038/ni.2035. 21739672

[38] T. A. Doering , A. Crawford , J. M. Angelosanto , M. A. Paley , C. G. Ziegler , and E. J. Wherry , “Network Analysis Reveals Centrally Connected Genes and Pathways Involved in CD8+ T Cell Exhaustion Versus Memory,” Immunity 37, no. 6 (2012): 1130–1144, 10.1016/j.immuni.2012.08.021. 23159438 PMC3749234

[39] E. J. Wherry , S. J. Ha , S. M. Kaech , et al., “Molecular Signature of CD8+ T Cell Exhaustion during Chronic Viral Infection,” Immunity 27, no. 4 (2007): 670–684, 10.1016/j.immuni.2007.09.006. 17950003

[40] S. D. Blackburn , H. Shin , W. N. Haining , et al., “Coregulation of CD8+ T Cell Exhaustion by Multiple Inhibitory Receptors During Chronic Viral Infection,” Nature Immunology 10, no. 1 (2009): 29–37, 10.1038/ni.1679. 19043418 PMC2605166

[41] E. J. Wherry , J. N. Blattman , K. Murali‐Krishna , R. van der Most , and R. Ahmed , “Viral Persistence Alters CD8 T‐Cell Immunodominance and Tissue Distribution and Results in Distinct Stages of Functional Impairment,” Journal of Virology 77, no. 8 (2003): 4911, 10.1128/JVI.77.8.4911-4927.2003. 12663797 PMC152117

[42] E. O'Brien and K. O'Malley , “Twenty‐Four‐Hour Ambulatory Blood Pressure Monitoring: A Review of Validation Data,” Journal of Hypertension Supplement 8, no. 6 (1990): S11. 2081992

[43] A. Gros , P. F. Robbins , X. Yao , et al., “PD‐1 Identifies the Patient‐Specific CD8+ Tumor‐reactive Repertoire Infiltrating Human Tumors,” Journal of Clinical Investigation 124, no. 5 (2014): 2246, 10.1172/JCI73639. 24667641 PMC4001555

[44] N. Nakamoto , H. Cho , A. Shaked , et al., “Synergistic Reversal of Intrahepatic HCV‐Specific CD8 T Cell Exhaustion by Combined PD‐1/CTLA‐4 Blockade,” PLoS Pathogens 5, no. 2 (2009): 1000313, 10.1371/journal.ppat.1000313. PMC2642724 19247441

[45] J. P. Allison , C. Benoist , and A. V. Chervonsky , “Nobels: Toll Pioneers Deserve Recognition,” Nature 479, no. 7372 (2011): 178, 10.1038/479178a. 22071753

[46] K. Chamoto , T. Yaguchi , M. Tajima , and T. Honjo , “Insights From a 30‐Year Journey: Function, Regulation and Therapeutic Modulation of PD1,” Nature Reviews Immunology 23, no. 10 (2023): 682–695, 10.1038/s41577-023-00867-9. 37185300

[47] K. E. Pauken , M. A. Sammons , P. M. Odorizzi , et al., “Epigenetic Stability of Exhausted T Cells Limits Durability of Reinvigoration by PD‐1 Blockade,” Science 354, no. 6316 (2016): 1160–1165, 10.1126/science.aaf2807. 27789795 PMC5484795

[48] P. Tonnerre , D. Wolski , S. Subudhi , et al., “Differentiation of Exhausted CD8+ T Cells After Termination of Chronic Antigen Stimulation Stops Short of Achieving Functional T Cell Memory,” Nature Immunology 22, no. 8 (2021): 1030–1041, 10.1038/s41590-021-00982-6. 34312544 PMC8323980

[49] N. Hensel , Z. Gu , D. W. Sagar , et al., “Memory‐Like HCV‐Specific CD8+ T Cells Retain a Molecular Scar After Cure of Chronic HCV Infection,” Nature Immunology 22, no. 2 (2021): 229–239, 10.1038/s41590-020-00817-w. 33398179

[50] K. S. P. Devi , E. Wang , A. Jaiswal , et al., “PD‐1 Is Requisite for Skin TRM Cell Formation and Specification by TGFβ,” Nature Immunology 26, no. 8 (2025): 1339–1351, 10.1038/s41590-025-02228-1. 40730902 PMC12307224

[51] M. Osaki and S. Sakaguchi , “Soluble CTLA‐4 Regulates Immune Homeostasis and Promotes Resolution of Inflammation by Suppressing Type 1 But Allowing Type 2 Immunity,” Immunity 58, no. 4 (2025): 889–908, 10.1016/j.immuni.2025.03.004. 40168991

[52] L. Monney , C. A. Sabatos , J. L. Gaglia , et al., “Th1‐Specific Cell Surface Protein Tim‐3 Regulates Macrophage Activation and Severity of an Autoimmune Disease,” Nature 415, no. 6871 (2002): 536–541, 10.1038/415536a. 11823861

[53] F. Triebel , S. Jitsukawa , E. Baixeras , et al., “LAG‐3, A Novel Lymphocyte Activation Gene Closely Related to CD4,” The Journal of Experimental Medicine 171, no. 5 (1990): 1393–1405, 10.1084/jem.171.5.1393. 1692078 PMC2187904

[54] J. Du , H. Chen , J. You , et al., “Proximity Between LAG‐3 and the T Cell Receptor Guides Suppression of T Cell Activation and Autoimmunity,” Cell 188, no. 15 (2025): 4025–4042, 10.1016/j.cell.2025.06.004.40592325

[55] N. Joller , A. C. Anderson , and V. K. Kuchroo , “LAG‐3, TIM‐3, and TIGIT: Distinct Functions in Immune Regulation,” Immunity 57, no. 2 (2024): 206–222, 10.1016/j.immuni.2024.01.010. 38354701 PMC10919259

[56] K. Wing , Y. Onishi , P. Prieto‐Martin , et al., “CTLA‐4 Control over Foxp3 + Regulatory T Cell Function,” Science 322, no. 5899 (2008): 271–275, 10.1126/science.1160062. 18845758

[57] S. Minguet , M. V. Maus , and W. W. Schamel , “From TCR Fundamental Research to Innovative Chimeric Antigen Receptor Design,” Nature Reviews Immunology 25, no. 3 (2025): 212–224, 10.1038/s41577-024-01093-7. 39433885

[58] O. S. Qureshi , Y. Zheng , K. Nakamura , et al., “Trans‐Endocytosis of CD80 and CD86: a Molecular Basis for the Cell‐Extrinsic Function of CTLA‐4,” Science 332, no. 6029 (2011): 600–603, 10.1126/science.1202947. 21474713 PMC3198051

[59] J. M. Chemnitz , R. V. Parry , K. E. Nichols , C. H. June , and J. L. Riley , “SHP‐1 and SHP‐2 Associate with Immunoreceptor Tyrosine‐Based Switch Motif of Programmed Death 1 Upon Primary Human T Cell Stimulation, But Only Receptor Ligation Prevents T Cell Activation,” The Journal of Immunology 173, no. 2 (2004): 945, 10.4049/jimmunol.173.2.945. 15240681

[60] K. Bardhan , H. I. Aksoylar , T. L. Bourgeois , et al., “Phosphorylation of PD‐1‐Y248 Is a Marker of PD‐1‐mediated Inhibitory Function in Human T Cells,” Scientific Reports 9, no. 1 (2019): 17252, 10.1038/s41598-019-53463-0. 31754127 PMC6872651

[61] T. Yokosuka , M. Takamatsu , W. Kobayashi‐Imanishi , A. Hashimoto‐Tane , M. Azuma , and T. Saito , “Programmed Cell Death 1 Forms Negative Costimulatory Microclusters That Directly Inhibit T Cell Receptor Signaling by Recruiting Phosphatase SHP2,” Journal of Experimental Medicine 209, no. 6 (2012): 1201, 10.1084/jem.20112741. 22641383 PMC3371732

[62] E. Ahn , K. Araki , M. Hashimoto , et al., “Role of PD‐1 During Effector CD8 T Cell Differentiation,” Proceedings of the National Academy of Sciences 115, no. 18 (2018): 4749–4754, 10.1073/pnas.1718217115. PMC5939075 29654146

[63] M. Quigley , F. Pereyra , B. Nilsson , et al., “Transcriptional Analysis of HIV‐Specific CD8+ T Cells Shows That PD‐1 Inhibits T Cell Function by Upregulating BATF,” Nature Medicine 16, no. 10 (2010): 1147–1151, 10.1038/nm.2232. PMC3326577 20890291

[64] M. Philip and A. Schietinger , “CD8+ T Cell Differentiation and Dysfunction in Cancer,” Nature Reviews Immunology 22, no. 4 (2022): 209, 10.1038/s41577-021-00574-3. PMC9792152 34253904

[65] J. Fourcade , Z. Sun , M. Benallaoua , et al., “Upregulation of Tim‐3 and PD‐1 Expression Is Associated with Tumor Antigen–Specific CD8+ T Cell Dysfunction in Melanoma Patients,” Journal of Experimental Medicine 207, no. 10 (2010): 2175–2186, 10.1084/jem.20100637. 20819923 PMC2947081

[66] T. G. Kang , J. T. Johnson , C. C. Zebley , and B. Youngblood , “Epigenetic Regulation of T Cell Exhaustion in Cancer,” Nature Reviews Cancer 26 (2025): 46–61. 41145849 10.1038/s41568-025-00883-y PMC13203999

[67] G. P. Mognol , R. Spreafico , V. Wong , et al., “Exhaustion‐Associated Regulatory Regions in CD8 + Tumor‐Infiltrating T Cells,” Proceedings of the National Academy of Sciences of the United States of America 114, no. 13 (2017): E2776, 10.1073/pnas.1620498114. 28283662 PMC5380094

[68] T. Srirat , T. Hayakawa , S. Mise‐Omata , et al., “NR4a1/2 Deletion Promotes Accumulation of TCF1+ Stem‐Like Precursors of Exhausted CD8+ T Cells in the Tumor Microenvironment,” Cell Reports 43, no. 3 (2024): 113898, 10.1016/j.celrep.2024.113898. 38451819

[69] G. J. Martinez , R. M. Pereira , T. Aijo , et al., “The Transcription Factor NFAT Promotes Exhaustion of Activated CD8 + T Cells,” Immunity 42, no. 2 (2015): 265, 10.1016/j.immuni.2015.01.006. 25680272 PMC4346317

[70] Y. J. Huang , S. F. Ngiow , A. E. Baxter , et al., “Continuous Expression of TOX Safeguards Exhausted CD8 T Cell Epigenetic Fate,” Science Immunology 10, no. 105 (2025): ado3032, 10.1126/sciimmunol.ado3032. 40053604

[71] C. Liang , S. Huang , Y. Zhao , S. Chen , and Y. Li , “TOX as a Potential Target for Immunotherapy in Lymphocytic Malignancies,” Biomarker Research 9, no. 1 (2021): 20, 10.1186/s40364-021-00275-y. 33743809 PMC7981945

[72] K. B. Yates , P. Tonnerre , G. E. Martin , et al., “Epigenetic Scars of CD8+ T Cell Exhaustion Persist after Cure of Chronic Infection in Humans,” Nature Immunology 22, no. 8 (2021): 1020, 10.1038/s41590-021-00979-1. 34312547 PMC8600539

[73] J. P. Scott‐Browne , I. F. Lopez‐Moyado , S. Trifari , et al., “Dynamic Changes in Chromatin Accessibility Occur in CD8 + T Cells Responding to Viral Infection,” Immunity 45, no. 6 (2016): 1327–1340, 10.1016/j.immuni.2016.10.028. 27939672 PMC5214519

[74] B. C. Miller , D. R. Sen , R. Al Abosy , et al., “Subsets of Exhausted CD8+ T Cells Differentially Mediate Tumor Control and Respond to Checkpoint Blockade,” Nature Immunology 20, no. 3 (2019): 326–336, 10.1038/s41590-019-0312-6. 30778252 PMC6673650

[75] B. Prinzing , C. C. Zebley , C. T. Petersen , et al., “Deleting DNMT3A in CAR T Cells Prevents Exhaustion and Enhances Antitumor Activity,” Science Translational Medicine 13, no. 620 (2021): abh0272, 10.1126/scitranslmed.abh0272. PMC8733956 34788079

[76] M. Zhao , C. H. Kiernan , C. J. Stairiker , et al., “Rapid In Vitro Generation of Bona Fide Exhausted CD8+ T Cells Is Accompanied by Tcf7 Promotor Methylation,” PLOS Pathogens 16, no. 6 (2020): 1008555, 10.1371/journal.ppat.1008555. PMC7340326 32579593

[77] C. Lu , “Abstract PR004: Interplay Between Histone Modifications and DNA Methylation in Cancer,” Cancer Research 85, no. 3 (2025): PR004–PR004, 10.1158/1538-7445.DNAMETHYLATION-PR004.

[78] G. Sendzikaite , C. W. Hanna , K. R. Stewart‐Morgan , E. Ivanova , and G. Kelsey , “A DNMT3A PWWP Mutation Leads to Methylation of Bivalent Chromatin and Growth Retardation in Mice,” Nature Communications 10, no. 1 (2019): 1884, 10.1038/s41467-019-09713-w. PMC6478690 31015495

[79] C. Schmidl , M. Delacher , J. Huehn , and M. Feuerer , “Epigenetic Mechanisms Regulating T‐Cell Responses,” Journal of Allergy and Clinical Immunology 142, no. 3 (2018): 728–743, 10.1016/j.jaci.2018.07.014. 30195378

[80] B. R. Ford , P. D. A. Vignali , N. L. Rittenhouse , et al., “Tumor Microenvironmental Signals Reshape Chromatin Landscapes to Limit the Functional Potential of Exhausted T Cells,” Science Immunology 7, no. 74 (2022): abj9123, 10.1126/sciimmunol.abj9123. PMC9851604 35930654

[81] X. Zeng , L. Wei , L. Lv , et al., “Revealing Key Regulatory Factors in Lung Adenocarcinoma: The Role of Epigenetic Regulation of Autophagy‐Related Genes from Transcriptomics, scRNA‐seq, and Machine Learning,” Frontiers in Pharmacology 16 (2025): 1542338, 10.3389/fphar.2025.1542338. 40832611 PMC12359839

[82] E. Battistello , K. A. Hixon , D. E. Comstock , et al., “Stepwise Activities of mSWI/SNF family Chromatin Remodeling Complexes Direct T Cell Activation and Exhaustion,” Molecular Cell 83, no. 8 (2023): 1216–1236, 10.1016/j.molcel.2023.02.026. 36944333 PMC10121856

[83] K. Zhuang , L. Wang , C. Lu , et al., “Assessment of SWI/SNF Chromatin Remodeling Complex Related Genes as Potential Biomarkers and Therapeutic Targets in Pan‐Cancer,” Molecular Cancer 23, no. 1 (2024): 176, 10.1186/s12943-024-02015-w. 39192265 PMC11348550

[84] X. Liao , Y. Guo , Y. He , Y. Xiao , J. Li , and R. Liu , “Metabolic Enzymes Function as Epigenetic Modulators: A Trojan Horse for Chromatin Regulation and Gene Expression,” Pharmacological Research 173 (2021): 105834, 10.1016/j.phrs.2021.105834. 34450321

[85] C. Bao , Q. Ma , X. Ying , et al., “Histone Lactylation in Macrophage Biology and Disease: from Plasticity Regulation to Therapeutic Implications,” EBioMedicine 111 (2025): 105502, 10.1016/j.ebiom.2024.105502. 39662177 PMC11697715

[86] D. Raychaudhuri , P. Singh , B. Chakraborty , et al., “Histone Lactylation Drives CD8+ T Cell Metabolism and Function,” Nature Immunology 25, no. 11 (2024): 2140–2151, 10.1038/s41590-024-01985-9. 39375549 PMC13211864

[87] W. Wu , J. Zhang , H. Sun , et al., “Glycolysis Induces Abnormal Transcription through Histone Lactylation in T‐cell Acute Lymphoblastic Leukemia,” Genomics, Proteomics & Bioinformatics 23, no. 2 (2025), 10.1093/gpbjnl/qzaf029. PMC12402983 40193528

[88] D. Raychaudhuri , P. Singh , B. Chakraborty , A. Tannir , S. Meher , and S. Goswami , “Abstract 4832: Differential Roles of Histone Lactylation in Regulating CD8 T Cell Effector Functions and Exhaustion,” Cancer Research 85, no. 8 (2025): 4832, 10.1158/1538-7445.AM2025-4832.

[89] S. H. Moller , P. C. Hsueh , Y. R. Yu , L. Zhang , and P. C. Ho , “Metabolic Programs Tailor T Cell Immunity in Viral Infection, Cancer, and Aging,” Cell Metabolism 34, no. 3 (2022): 378–395, 10.1016/j.cmet.2022.02.003. 35235773

[90] Y. Yang , X. Li , F. Liu , et al., “Immunometabolite L‐2‐HG Promotes Epigenetic Modification of Exhausted T Cells and Improves Antitumor Immunity,” JCI Insight 10, no. 7 (2025): 174600. 10.1172/jci.insight.174600 PMC11981629 40043713

[91] M. Q. Yang , S. L. Zhang , L. Sun , et al., “Targeting Mitochondria: Restoring the Antitumor Efficacy of Exhausted T Cells,” Molecular Cancer 23, no. 1 (2024): 260, 10.1186/s12943-024-02175-9. 39563438 PMC11575104

[92] N. E. Scharping , A. V. Menk , R. S. Moreci , et al., “The Tumor Microenvironment Represses T Cell Mitochondrial Biogenesis to Drive Intratumoral T Cell Metabolic Insufficiency and Dysfunction,” Immunity 45, no. 2 (2016): 374–388, 10.1016/j.immuni.2016.07.009. 27496732 PMC5207350

[93] H. Wu , X. Zhao , S. M. Hochrein , et al., “Mitochondrial Dysfunction Promotes the Transition of Precursor to Terminally Exhausted T Cells Through HIF‐1α‐Mediated Glycolytic Reprogramming,” Nature Communications 14, no. 1 (2023): 6858, 10.1038/s41467-023-42634-3. PMC10611730 37891230

[94] Y. Huang , X. Si , M. Shao , X. Teng , G. Xiao , and H. Huang , “Rewiring Mitochondrial Metabolism to Counteract Exhaustion of CAR‐T Cells,” Journal of Hematology & Oncology 15, no. 1 (2022): 38, 10.1186/s13045-022-01255-x. 35346311 PMC8960222

[95] Y. R. Yu , H. Imrichova , H. Wang , et al., “Disturbed Mitochondrial Dynamics in CD8+ TILs Reinforce T Cell Exhaustion,” Nature Immunology 21, no. 12 (2020): 1540–1551, 10.1038/s41590-020-0793-3. 33020660

[96] L. Zhang , W. Zhang , Z. Li , et al., “Mitochondria Dysfunction in CD8+ T Cells as an Important Contributing Factor for Cancer Development and a Potential Target for Cancer Treatment: A Review,” Journal of Experimental & Clinical Cancer Research 41, no. 1 (2022): 227, 10.1186/s13046-022-02439-6. 35864520 PMC9306053

[97] J. Wang , R. Z. Li , W. J. Wang , et al., “CERS4 Predicts Positive Anti‐PD‐1 Response and Promotes Immunomodulation Through Rhob‐Mediated Suppression of CD8+Tim3+ Exhausted T Cells in Non‐Small Cell Lung Cancer,” Pharmacological Research 194 (2023): 106850, 10.1016/j.phrs.2023.106850. 37453674

[98] D. N. Edwards , V. M. Ngwa , A. L. Raybuck , et al., “Selective Glutamine Metabolism Inhibition in Tumor Cells Improves Antitumor T Lymphocyte Activity in Triple‐Negative Breast Cancer,” Journal of Clinical Investigation 131, no. 4 (2021), 10.1172/JCI140100. PMC7880417 33320840

[99] M. Pandit , Y. S. Kil , J. H. Ahn , et al., “Methionine Consumption by Cancer Cells Drives a Progressive Upregulation of PD‐1 Expression in CD4 T Cells,” Nature Communications 14, no. 1 (2023): 2593, 10.1038/s41467-023-38316-9. PMC10162977 37147330

[100] D. Saka , M. Gokalp , B. Piyade , et al., “Mechanisms of T‐Cell Exhaustion in Pancreatic Cancer,” Cancers (Basel) 12, no. 8 (2020): 2274. 32823814 10.3390/cancers12082274 PMC7464444

[101] B. Bengsch , A. L. Johnson , M. Kurachi , et al., “Bioenergetic Insufficiencies Due to Metabolic Alterations Regulated by the Inhibitory Receptor PD‐1 Are an Early Driver of CD8 + T Cell Exhaustion,” Immunity 45, no. 2 (2016): 358–373, 10.1016/j.immuni.2016.07.008. 27496729 PMC4988919

[102] P. J. Siska , G. J. van der Windt , R. J. Kishton , et al., “Suppression of Glut1 and Glucose Metabolism by Decreased Akt/mTORC1 Signaling Drives T Cell Impairment in B Cell Leukemia,” The Journal of Immunology 197, no. 6 (2016): 2532–2540, 10.4049/jimmunol.1502464. 27511728 PMC5010978

[103] R. M. Peralta , B. Xie , K. Lontos , et al., “Dysfunction of Exhausted T Cells Is Enforced by MCT11‐Mediated Lactate Metabolism,” Nature Immunology 25, no. 12 (2024): 2297–2307, 10.1038/s41590-024-01999-3. 39516648 PMC11588660

[104] I. Elia , J. H. Rowe , S. Johnson , et al., “Tumor Cells Dictate Anti‐Tumor Immune Responses by Altering Pyruvate Utilization and Succinate Signaling in CD8+ T Cells,” Cell Metabolism 34, no. 8 (2022): 1137–1150, 10.1016/j.cmet.2022.06.008. 35820416 PMC9357162

[105] M. Wenes , A. Jaccard , T. Wyss , et al., “The Mitochondrial Pyruvate Carrier Regulates Memory T Cell Differentiation and Antitumor Function,” Cell Metabolism 34, no. 5 (2022): 731–746, 10.1016/j.cmet.2022.03.013. 35452600 PMC9116152

[106] Q. Feng , Z. Liu , X. Yu , et al., “Lactate Increases Stemness of CD8 + T Cells to Augment Anti‐Tumor Immunity,” Nature Communications 13, no. 1 (2022): 4981, 10.1038/s41467-022-32521-8. PMC9448806 36068198

[107] Y. Jiang , Y. Li , and B. Zhu , “T‐cell Exhaustion in the Tumor Microenvironment,” Cell Death & Disease 6, no. 6 (2015): 1792, 10.1038/cddis.2015.162. PMC4669840 26086965

[108] F. Tuisku and C. Hildebrand , “Nodes of Ranvier and Myelin Sheath Dimensions Along Exceptionally Thin Myelinated Vertebrate PNS Axons,” Journal of Neurocytology 21, no. 11 (1992): 796–806, 10.1007/BF01237905. 1279131

[109] W. Chen , W. Jin , N. Hardegen , et al., “Conversion of Peripheral CD4+CD25− Naive T Cells to CD4+CD25+ Regulatory T Cells by TGF‐β Induction of Transcription Factor Foxp3,” The Journal of Experimental Medicine 198, no. 12 (2003): 1875–1886,, 10.1084/jem.20030152. 14676299 PMC2194145

[110] E. Loeuillard , J. Yang , E. Buckarma , et al., “Targeting Tumor‐Associated Macrophages and Granulocytic Myeloid‐derived Suppressor Cells Augments PD‐1 Blockade in Cholangiocarcinoma,” Journal of Clinical Investigation 130, no. 10 (2020): 5380, 10.1172/JCI137110. 32663198 PMC7524481

[111] A. A. Hurwitz and S. K. Watkins , “Immune Suppression in the Tumor Microenvironment: a Role for Dendritic Cell‐mediated Tolerization of T Cells,” Cancer Immunology, Immunotherapy 61, no. 2 (2012): 289–293, 10.1007/s00262-011-1181-5. 22237887 PMC6948839

[112] Y. J. Kim , S. J. Park , and H. E. Broxmeyer , “Phagocytosis, a Potential Mechanism for Myeloid‐Derived Suppressor Cell Regulation of CD8+ T Cell Function Mediated through Programmed Cell Death‐1 and Programmed Cell Death‐1 Ligand Interaction,” The Journal of Immunology 187, no. 5 (2011): 2291–2301, 10.4049/jimmunol.1002650. 21795591 PMC3159723

[113] B. F. Zamarron and W. Chen , “Dual Roles of Immune Cells and Their Factors in Cancer Development and Progression,” International Journal of Biological Sciences 7, no. 5 (2011): 651–658, 10.7150/ijbs.7.651. 21647333 PMC3107473

[114] M. Tran , J. R. Huh , and A. S. Devlin , “The Role of Gut Microbial Metabolites in the T Cell Lifecycle,” Nature Immunology 26, no. 8 (2025): 1246, 10.1038/s41590-025-02227-2. 40691327 PMC13124069

[115] W. Mou , Z. Deng , L. Zhu , et al., “Intratumoral Mycobiome Heterogeneity Influences the Tumor Microenvironment and Immunotherapy Outcomes in Renal Cell Carcinoma,” Science Advances 11, no. 15 (2025): adu1727, 10.1126/sciadv.adu1727. PMC11980860 40203108

[116] F. Lou , L. Yan , S. Luo , et al., “Dysbiotic Oral Microbiota‐derived Kynurenine, Induced by Chronic Restraint Stress, Promotes Head and Neck Squamous Cell Carcinoma by Enhancing CD8 + T Cell Exhaustion,” Gut 74, no. 6 (2025): 935, 10.1136/gutjnl-2024-333479. 39904603 PMC12229062

[117] Q. Zhang , Q. Zhao , T. Li , et al., “Lactobacillus Plantarum‐Derived Indole‐3‐Lactic Acid Ameliorates Colorectal Tumorigenesis via Epigenetic Regulation of CD8+ T Cell Immunity,” Cell Metabolism 35, no. 6 (2023): 943–960, 10.1016/j.cmet.2023.04.015. 37192617

[118] X. Yu , J. Ou , L. Wang , et al., “Gut Microbiota Modulate CD8 + T Cell Immunity in Gastric Cancer through Butyrate/GPR109A/HOPX,” Gut Microbes 16, no. 1 (2024): 2307542, 10.1080/19490976.2024.2307542. 38319728 PMC10854374

[119] D. R. Sen , J. Kaminski , R. A. Barnitz , et al., “The Epigenetic Landscape of T Cell Exhaustion,” Science 354, no. 6316 (2016): 1165–1169. 27789799 10.1126/science.aae0491 PMC5497589

[120] S. Xiang , S. Li , and J. Xu , “Unravelling T Cell Exhaustion Through Co‐Inhibitory Receptors and Its Transformative Role in Cancer Immunotherapy,” Clinical and Translational Medicine 15, no. 5 (2025): 70345, 10.1002/ctm2.70345. PMC12104568 40415479

[121] A. Moustaki , J. C. Crawford , S. Alli , et al., “Antigen Cross‐Presentation in Young Tumor‐Bearing Hosts Promotes CD8 + T Cell Terminal Differentiation,” Science Immunology 7, no. 68 (2022): abf6136, 10.1126/sciimmunol.abf6136. PMC8990347 35119937

[122] J. C. Beltra , S. Manne , M. S. Abdel‐Hakeem , et al., “Developmental Relationships of Four Exhausted CD8+ T Cell Subsets Reveals Underlying Transcriptional and Epigenetic Landscape Control Mechanisms,” Immunity 52, no. 5 (2020): 825, 10.1016/j.immuni.2020.04.014. 32396847 PMC8360766

[123] B. Daniel , K. E. Yost , S. Hsiung , et al., “Divergent Clonal Differentiation Trajectories of T Cell Exhaustion,” Nature Immunology 23, no. 11 (2022): 1614–1627, 10.1038/s41590-022-01337-5. 36289450 PMC11225711

[124] J. W. Polania , A. Hoyt‐Miggelbrink , W. H. Tomaszewski , et al., “Antigen Presentation by Tumor‐Associated Macrophages Drives T Cells from a Progenitor Exhaustion State to Terminal Exhaustion,” Immunity 58, no. 1 (2025): 232–246, 10.1016/j.immuni.2024.11.026. 39724910

[125] X. Lan , C. C. Zebley , and B. Youngblood , “Cellular and Molecular Waypoints Along the Path of T Cell Exhaustion,” Science Immunology 8, no. 87 (2023): adg3868, 10.1126/sciimmunol.adg3868. PMC10618911 37656775

[126] K. Tanoue , H. Ohmura , K. Uehara , et al., “Spatial Dynamics of CD39+CD8+ Exhausted T Cell Reveal Tertiary Lymphoid Structures‐Mediated Response to PD‐1 Blockade in Esophageal Cancer,” Nature Communications 15, no. 1 (2024): 9033, 10.1038/s41467-024-53262-w. PMC11490492 39426955

[127] S. Xu , C. Han , J. Zhou , et al., “Distinct Maturity and Spatial Distribution of Tertiary Lymphoid Structures in Head and Neck Squamous Cell Carcinoma: Implications for Tumor Immunity and Clinical Outcomes,” Cancer Immunology, Immunotherapy 74, no. 3 (2025): 107, 10.1007/s00262-025-03952-1. 39932546 PMC11813844

[128] A. R. Kim , S. J. Choi , J. Park , et al., “Spatial Immune Heterogeneity of Hypoxia‐Induced Exhausted Features in High‐Grade Glioma,” Oncoimmunology 11, no. 1 (2022): 2026019. 35036078 10.1080/2162402X.2022.2026019 PMC8757477

[129] S. Liu , S. Ma , G. Liu , et al., “CK2B Induces CD8 + T‐Cell Exhaustion Through HDAC8‐Mediated Epigenetic Reprogramming to Limit the Efficacy of Anti‐PD‐1 Therapy in Non‐Small‐Cell Lung Cancer,” Advanced Science 12, no. 16 (2025): 2411053, 10.1002/advs.202411053. 40013761 PMC12021095

[130] C. G. Kim , G. Kim , K. H. Kim , et al., “Distinct Exhaustion Features of T Lymphocytes Shape the Tumor‐Immune Microenvironment with Therapeutic Implication in Patients With Non‐Small‐Cell Lung Cancer,” Journal for ImmunoTherapy of Cancer 9, no. 12 (2021): 002780. 10.1136/jitc-2021-002780 PMC8671984 34907028

[131] S. Tietscher , J. Wagner , T. Anzeneder , et al., “A Comprehensive Single‐Cell Map of T Cell Exhaustion‐Associated Immune Environments in Human Breast Cancer,” Nature Communications 14, no. 1 (2023): 98, 10.1038/s41467-022-35238-w. PMC9822999 36609566

[132] C. Abrate , F. P. Canale , S. N. Bossio , et al., “CD8+ T Cells in Breast Cancer Tumors and Draining Lymph Nodes: PD‐1 Levels, Effector Functions and Prognostic Relevance,” Oncoimmunology 14, no. 1 (2025): 2502354. 40351118 10.1080/2162402X.2025.2502354 PMC12077459

[133] R. Mirzaei , S. Sarkar , and V. W. Yong , “T Cell Exhaustion in Glioblastoma: Intricacies of Immune Checkpoints,” Trends in Immunology 38, no. 2 (2017): 104–115, 10.1016/j.it.2016.11.005. 27964820

[134] T. Zheng , C. Ding , S. Lai , et al., “CD160 Dictates Anti‐PD‐1 Immunotherapy Resistance by Regulating CD8+ T Cell Exhaustion in Colorectal Cancer,” Nature Cell Biology 27, no. 9 (2025): 1555–1571, 10.1038/s41556-025-01753-3. 40925954

[135] S. Zeng , H. Hu , Z. Li , et al., “Local TSH/TSHR Signaling Promotes CD8 + T Cell Exhaustion and Immune Evasion in Colorectal Carcinoma,” Cancer Communications 44, no. 11 (2024): 1287, 10.1002/cac2.12605. 39285586 PMC11570765

[136] Y. Zhu , H. Tan , J. Wang , H. Zhuang , H. Zhao , and X. Lu , “Molecular Insight into T Cell Exhaustion in Hepatocellular Carcinoma,” Pharmacological Research 203 (2024): 107161, 10.1016/j.phrs.2024.107161. 38554789

[137] F. Blaeschke , S. Willier , D. Stenger , et al., “Leukemia‐Induced Dysfunctional TIM‐3+CD4+ Bone Marrow T Cells Increase Risk of Relapse in Pediatric B‐Precursor ALL Patients,” Leukemia 34, no. 10 (2020): 2607, 10.1038/s41375-020-0793-1. 32203137

[138] S. I. Tracy , H. Venkatesh , C. Hekim , et al., “Combining Nilotinib and PD‐L1 Blockade Reverses CD4+ T‐Cell Dysfunction and Prevents Relapse in Acute B‐cell Leukemia,” Blood 140, no. 4 (2022): 335. 35275990 10.1182/blood.2021015341 PMC9335501

[139] L. Liu , A. Wang , X. Liu , et al., “Blocking TIGIT/CD155 Signalling Reverses CD8+ T Cell Exhaustion and Enhances the Antitumor Activity in Cervical Cancer,” Journal of Translational Medicine 20, no. 1 (2022): 280, 10.1186/s12967-022-03480-x. 35729552 PMC9210727

[140] Y. Sun , Y. Zhou , Q. Peng , et al., “SERINC2‐Mediated Serine Metabolism Promotes Cervical Cancer Progression and Drives T Cell Exhaustion,” International Journal of Biological Sciences 21, no. 3 (2025): 1361–1377, 10.7150/ijbs.105572. 39897034 PMC11781177

[141] Z. Liu , Y. Zhang , N. Ma , et al., “Progenitor‐Like Exhausted SPRY1+CD8+ T Cells Potentiate Responsiveness to Neoadjuvant PD‐1 Blockade in Esophageal Squamous Cell Carcinoma,” Cancer Cell 41, no. 11 (2023): 1852–1870, 10.1016/j.ccell.2023.09.011. 37832554

[142] L. M. McLane , M. S. Abdel‐Hakeem , and E. J. Wherry , “CD8 T Cell Exhaustion During Chronic Viral Infection and Cancer,” Annual Review of Immunology 37 (2019): 457, 10.1146/annurev-immunol-041015-055318. 30676822

[143] R. Mu , R. Barakat , and D. H. Gutmann , “Pan‐cancer Single Cell Transcriptomic Clustering Reveals Heterogeneous CD8+ Exhausted T Cell Populations with Different Immune Checkpoint Inhibitor Responses,” Oncoimmunology 14, no. 1 (2025): 2540504. 40753639 10.1080/2162402X.2025.2540504 PMC12320814

[144] A. Crawford , J. M. Angelosanto , C. Kao , et al., “Molecular and Transcriptional Basis of CD4+ T Cell Dysfunction During Chronic Infection,” Immunity 40, no. 2 (2014): 289–302, 10.1016/j.immuni.2014.01.005. 24530057 PMC3990591

[145] W. Zhou , S. Kawashima , T. Ishino , et al., “Stem‐Like Progenitor and Terminally Differentiated TFH‐Like CD4+ T Cell Exhaustion in the Tumor Microenvironment,” Cell Reports 43, no. 2 (2024): 113797, 10.1016/j.celrep.2024.113797. 38363680

[146] A. M. Miggelbrink , J. D. Jackson , S. J. Lorrey , et al., “CD4 T‐Cell Exhaustion: Does It Exist and What Are Its Roles in Cancer?,” Clinical Cancer Research 27, no. 21 (2021): 5742–5752, 10.1158/1078-0432.CCR-21-0206. 34127507 PMC8563372

[147] Z. Liu , E. L. McMichael , G. Shayan , et al., “Novel Effector Phenotype of Tim‐3+ Regulatory T Cells Leads to Enhanced Suppressive Function in Head and Neck Cancer Patients,” Clinical Cancer Research 24, no. 18 (2018): 4529–4538, 10.1158/1078-0432.CCR-17-1350. 29712685 PMC6139056

[148] J. Liu , M. Wu , Y. Yang , et al., “BTN3A1 Expressed in Cervical Cancer Cells Promotes Vγ9Vδ2 T Cells Exhaustion Through Upregulating Transcription Factors NR4A2/3 Downstream of TCR Signaling,” Cell Communication and Signaling 22, no. 1 (2024): 459, 10.1186/s12964-024-01834-0. 39342337 PMC11439235

[149] M. H. Nada , H. Wang , A. J. Hussein , Y. Tanaka , and C. T. Morita , “PD‐1 Checkpoint Blockade Enhances Adoptive Immunotherapy by Human Vγ2Vδ2 T Cells Against Human Prostate Cancer,” Oncoimmunology 10, no. 1 (2021): 1989789. 34712512 10.1080/2162402X.2021.1989789 PMC8547840

[150] E. Leeansyah , A. Ganesh , M. F. Quigley , et al., “Activation, Exhaustion, and Persistent Decline of the Antimicrobial MR1‐Restricted MAIT‐Cell Population in Chronic HIV‐1 Infection,” Blood 121, no. 7 (2013): 1124, 10.1182/blood-2012-07-445429. 23243281 PMC3575756

[151] S. Deschler , J. Pohl‐Topcu , L. Ramsauer , et al., “Polyunsaturated Fatty Acid‐Induced Metabolic Exhaustion and Ferroptosis Impair the Anti‐Tumour Function of MAIT Cells in MASLD,” Journal of Hepatology 83 (2025): 1364–1378. 40543602 10.1016/j.jhep.2025.06.006

[152] C. M. Bucks , J. A. Norton , A. C. Boesteanu , Y. M. Mueller , and P. D. Katsikis , “Chronic Antigen Stimulation Alone Is Sufficient to Drive CD8+ T Cell Exhaustion,” The Journal of Immunology 182, no. 11 (2009): 6697–6708, 10.4049/jimmunol.0800997. 19454664 PMC2923544

[153] S. M. Kaech and W. Cui , “Transcriptional Control of Effector and Memory CD8+ T Cell Differentiation,” Nature Reviews Immunology 12, no. 11 (2012): 749–761, 10.1038/nri3307. PMC4137483 23080391

[154] Y. Fu , J. Wang , C. Liu , et al., “Glycogen Synthase Kinase 3 Controls T‐Cell Exhaustion by Regulating NFAT Activation,” Cellular & Molecular Immunology 20, no. 10 (2023): 1127, 10.1038/s41423-023-01075-0. 37553428 PMC10541428

[155] A. C. Wells , K. A. Hioki , C. C. Angelou , et al., “Let‐7 Enhances Murine Anti‐Tumor CD8 T Cell Responses by Promoting Memory and Antagonizing Terminal Differentiation,” Nature Communications 14, no. 1 (2023): 5585, 10.1038/s41467-023-40959-7. PMC10495470 37696797

[156] M. W. Rudloff , P. Zumbo , N. R. Favret , et al., “Hallmarks of CD8+ T Cell Dysfunction Are Established within Hours of Tumor Antigen Encounter Before Cell Division,” Nature Immunology 24, no. 9 (2023): 1527–1539, 10.1038/s41590-023-01578-y. 37537361 PMC10878719

[157] W. Zheng , J. Wei , C. C. Zebley , et al., “Regnase‐1 Suppresses TCF‐1+ Precursor Exhausted T‐cell Formation to Limit CAR–T‐Cell Responses Against ALL,” Blood 138, no. 2 (2021): 122–135, 10.1182/blood.2020009309. 33690816 PMC8288655

[158] I. Siddiqui , K. Schaeuble , V. Chennupati , et al., “Intratumoral Tcf1+PD‐1+CD8+ T Cells with Stem‐Like Properties Promote Tumor Control in Response to Vaccination and Checkpoint Blockade Immunotherapy,” Immunity 50, no. 1 (2019): 195–211, 10.1016/j.immuni.2018.12.021. 30635237

[159] M. K. Rahim , T. L. H. Okholm , K. B. Jones , et al., “Dynamic CD8+ T Cell Responses to Cancer Immunotherapy in Human Regional Lymph Nodes Are Disrupted in Metastatic Lymph Nodes,” Cell 186, no. 6 (2023): 1127–1143, 10.1016/j.cell.2023.02.021. 36931243 PMC10348701

[160] J. C. Beltra , M. S. Abdel‐Hakeem , S. Manne , et al., “Stat5 Opposes the Transcription Factor Tox and Rewires Exhausted CD8+ T Cells toward Durable Effector‐Like States During Chronic Antigen Exposure,” Immunity 56, no. 12 (2023): 2699–2718, 10.1016/j.immuni.2023.11.005. 38091951 PMC10752292

[161] Q. Sun and C. Dong , “Regulators of CD8+ T Cell Exhaustion,” Nature Reviews Immunology (2025), 10.1038/s41577-025-01221-x. 41034337

[162] M. Hofmann , R. Thimme , and W. W. Schamel , “PD‐1 and LAG‐3: Synergistic Fostering of T Cell Exhaustion,” Signal Transduction and Targeted Therapy 9, no. 1 (2024): 291, 10.1038/s41392-024-02000-1. 39424778 PMC11489778

[163] H. Ji , C. Hu , X. Yang , et al., “Lymph Node Metastasis in Cancer Progression: Molecular Mechanisms, Clinical Significance and Therapeutic Interventions,” Signal Transduction and Targeted Therapy 8, no. 1 (2023): 367, 10.1038/s41392-023-01576-4. 37752146 PMC10522642

[164] L. Ni , “Potential Mechanisms of Cancer Stem‐like Progenitor T‐Cell Bio‐Behaviours,” Clinical and Translational Medicine 14, no. 8 (2024): 1817, 10.1002/ctm2.1817. PMC11338842 39169517

[165] A. L. Gill , P. H. Wang , J. Lee , et al., “PD‐1 Blockade Increases the Self‐Renewal of Stem‐Like CD8 T Cells to Compensate for Their Accelerated Differentiation into Effectors,” Science Immunology 8, no. 86 (2023): adg0539, 10.1126/sciimmunol.adg0539. PMC10798572 37624909

[166] T. Jia , Y. Guo , X. M. Cheng , et al., “Multi‐Omics Profiling Identifies TNFRSF18 as a Novel Marker of Exhausted CD8^+^ T Cells and Reveals Tumour‐Immune Dynamics in Colorectal Cancer,” Clinical and Translational Medicine 15, no. 8 (2025): 70425, 10.1002/ctm2.70425. PMC12328248 40770837

[167] L. Zuyin , L. Zhao , C. Qian , et al., “Single‐Cell and Spatial Transcriptomics Delineate the Microstructure and Immune Landscape of Intrahepatic Cholangiocarcinoma in the Leading‐Edge Area,” Advanced Science 12, no. 7 (2025): 2412740, 10.1002/advs.202412740. 39716897 PMC11831447

[168] C. Mangana and B. B. Maier , “A Progressive T Cell Exhaustion Program Mapped across Tissues in Patients with Lung Cancer on Immune Checkpoint Blockade,” Cancer Cell 41, no. 4 (2023): 653, 10.1016/j.ccell.2023.02.020. 37001525

[169] M. John , M. Helal , J. Duell , et al., “Spatial Transcriptomics Reveals Profound Subclonal Heterogeneity and T‐cell Dysfunction in Extramedullary Myeloma,” Blood 144, no. 20 (2024): 2121–2135, 10.1182/blood.2024024590. 39172759

[170] H. N. Bell , A. K. Huber , R. Singhal , et al., “Microenvironmental Ammonia Enhances T Cell Exhaustion in Colorectal Cancer,” Cell Metabolism 35, no. 1 (2023): 134–149, 10.1016/j.cmet.2022.11.013. 36528023 PMC9841369

[171] L. Hao , S. Li , and X. Hu , “New Insights into T‐Cell Exhaustion in Liver Cancer: From Mechanism to Therapy,” Journal of Cancer Research and Clinical Oncology 149, no. 13 (2023): 12543–12560, 10.1007/s00432-023-05083-5. 37423958 PMC11797504

[172] T. N. Schumacher and D. S. Thommen , “Tertiary Lymphoid Structures in Cancer,” Science 375, no. 6576 (2022): abf9419, 10.1126/science.abf9419. 34990248

[173] F. Peyraud , J. P. Guegan , L. Vanhersecke , et al., “Tertiary Lymphoid Structures and Cancer Immunotherapy: From Bench to Bedside,” Med 6, no. 1 (2025): 100546, 10.1016/j.medj.2024.10.023. 39798544

[174] H. Li , M. J. Zhang , B. Zhang , et al., “Mature Tertiary Lymphoid Structures Evoke Intra‐Tumoral T and B Cell Responses via Progenitor Exhausted CD4+ T Cells in Head and Neck Cancer,” Nature Communications 16, no. 1 (2025): 4228, 10.1038/s41467-025-59341-w. PMC12059173 40335494

[175] T. S. Groen‐van Schooten , R. F. Fernandez , N. C. T. van Grieken , et al., “Mapping the Complexity and Diversity of Tertiary Lymphoid Structures in Primary and Peritoneal Metastatic Gastric Cancer,” Journal for ImmunoTherapy of Cancer 12, no. 7 (2024): 009243. 10.1136/jitc-2024-009243 PMC11218001 38955417

[176] Y. Zhang , M. Xu , Y. Ren , et al., “Tertiary Lymphoid Structural Heterogeneity Determines Tumour Immunity and Prospects for Clinical Application,” Molecular Cancer 23, no. 1 (2024): 75, 10.1186/s12943-024-01980-6. 38582847 PMC10998345

[177] W. T. Kuo , I. Y. Kuo , H. C. Hsieh , S. T. Wu , W. C. Su , and Y. C. Wang , “Rab37 mediates Trafficking and Membrane Presentation of PD‐1 to Sustain T Cell Exhaustion in Lung Cancer,” Journal of Biomedical Science 31, no. 1 (2024): 20, 10.1186/s12929-024-01009-6. 38321486 PMC10848371

[178] Y. Yan , D. Sun , J. Hu , et al., “Multi‐Omic Profiling Highlights Factors Associated with Resistance to Immuno‐Chemotherapy in Non‐Small‐Cell Lung Cancer,” Nature Genetics 57, no. 1 (2025): 126–139, 10.1038/s41588-024-01998-y. 39658657

[179] S. Klein , A. Schulte , C. Arolt , et al., “Intratumoral Abundance of M2‐Macrophages Is Associated with Unfavorable Prognosis and Markers of T‐Cell Exhaustion in Small Cell Lung Cancer Patients,” Modern Pathology 36, no. 10 (2023): 100272, 10.1016/j.modpat.2023.100272. 37423586

[180] D. Feng , D. Pu , J. Ren , et al., “CD8+ T‐Cell Exhaustion: Impediment to Triple‐Negative Breast Cancer (TNBC) Immunotherapy,” Biochimica Et Biophysica Acta (BBA)—Reviews on Cancer 1879, no. 6 (2024): 189193, 10.1016/j.bbcan.2024.189193. 39413858

[181] H. Xie , X. Xi , T. Lei , H. Liu , and Z. Xia , “CD8+ T Cell Exhaustion in the Tumor Microenvironment of Breast Cancer,” Front Immunol 15 (2024): 1507283. 39717767 10.3389/fimmu.2024.1507283 PMC11663851

[182] X. Yu and J. Xu , “TWIST1 Drives Cytotoxic CD8+ T‐Cell Exhaustion through Transcriptional Activation of CD274 (PD‐L1) Expression in Breast Cancer Cells,” Cancers 16, no. 11 (2024): 1973. 38893094 10.3390/cancers16111973 PMC11171171

[183] M. Chen , Z. Fu , and C. Wu , “Tumor‐Derived Exosomal ICAM1 Promotes Bone Metastasis of Triple‐negative Breast Cancer by Inducing CD8+ T Cell Exhaustion,” The International Journal of Biochemistry & Cell Biology 175 (2024): 106637, 10.1016/j.biocel.2024.106637. 39147124

[184] B. J. Puviindran , S. Wallace , K. Ayasoufi , E. Lerner , and P. E. Fecci , “Within and Beyond the Tumor: Mechanisms of Glioblastoma‐induced Immunosuppression,” Neurooncological Advances 7, no. 4 (2025): iv4–iv18. 10.1093/noajnl/vdaf006 PMC12418596 40933031

[185] K. Woroniecka , P. Chongsathidkiet , K. Rhodin , et al., “T‐Cell Exhaustion Signatures Vary with Tumor Type and Are Severe in Glioblastoma,” Clinical Cancer Research 24, no. 17 (2018): 4175–4186, 10.1158/1078-0432.CCR-17-1846. 29437767 PMC6081269

[186] A. Z. Wang , B. L. Mashimo , M. O. Schaettler , et al., “Glioblastoma‐Infiltrating CD8 + T Cells Are Predominantly a Clonally Expanded GZMK + Effector Population,” Cancer Discovery 14, no. 6 (2024): 1106, 10.1158/2159-8290.CD-23-0913. 38416133 PMC13480461

[187] D. Henrik Heiland , V. M. Ravi , S. P. Behringer , et al., “Tumor‐Associated Reactive Astrocytes Aid the Evolution of Immunosuppressive Environment in Glioblastoma,” Nature Communications 10, no. 1 (2019): 2541, 10.1038/s41467-019-10493-6. PMC6559986 31186414

[188] D. M. Long , “Capillary Ultrastructure and the Blood‐Brain Barrier in Human Malignant Brain Tumors,” Journal of Neurosurgery 32, no. 2 (1970): 127–144, 10.3171/jns.1970.32.2.0127. 5411991

[189] S. Liu , X. Liu , C. Zhang , W. Shan , and X. Qiu , “T‐Cell Exhaustion Status under High and Low Levels of Hypoxia‐Inducible Factor 1α Expression in Glioma,” Frontiers in Pharmacology 12 (2021): 711772, 10.3389/fphar.2021.711772. 34305618 PMC8299942

[190] J. Lee , M. Nicosia , E. S. Hong , et al., “Sex‐Biased T‐Cell Exhaustion Drives Differential Immune Responses in Glioblastoma,” Cancer Discovery 13, no. 9 (2023): 2090, 10.1158/2159-8290.CD-22-0869. 37378557 PMC10481130

[191] A. M. Hoyt‐Miggelbrink , J. W. Polania , L. Wachsmuth , et al., “Upregulation of TNFR2 Precedes TOX Expression by Exhausted T Cells and Restricts Antitumor and Antiviral Immunity,” Clinical Cancer Research (2025). 10.1158/1078-0432.CCR-24-3455 PMC13056295 41269210

[192] S. Du Four , S. K. Maenhout , D. Benteyn , et al., “Disease Progression in Recurrent Glioblastoma Patients Treated with the VEGFR Inhibitor Axitinib Is Associated with Increased Regulatory T Cell Numbers and T Cell Exhaustion,” Cancer Immunology, Immunotherapy 65, no. 6 (2016): 727, 10.1007/s00262-016-1836-3. 27098427 PMC11029796

[193] J. D. Fumet , C. Latour , L. Nuttin , et al., “Tumor‐Associated Macrophages Produce PGE2 to Promote CD8+ T Cell Exhaustion and Drive Resistance to PD‐L1 Blockade in Microsatellite Stable Colorectal Cancer,” Cancer Research (2025). 10.1158/0008-5472.CAN-25-0079 41196020

[194] C. Shasha , D. R. Glass , E. Moelhman , et al., “Hallmarks of T‐Cell Exhaustion and Antigen Experience Are Absent in Multiple Myeloma From Diagnosis to Maintenance Therapy,” Blood 145, no. 26 (2025): 3113–3123, 10.1182/blood.2024025655. 40163891 PMC12782984

[195] Y. Zhao , P. Liao , S. Huang , et al., “Increased TOX Expression Associates with Exhausted T Cells in Patients with Multiple Myeloma,” Experimental Hematology & Oncology 11, no. 1 (2022): 12, 10.1186/s40164-022-00267-0. 35246241 PMC8895562

[196] S. A. Minnie , O. G. Waltner , P. Zhang , et al., “TIM‐3 + CD8 T Cells with a Terminally Exhausted Phenotype Retain Functional Capacity in Hematological Malignancies,” Science Immunology 9, no. 94 (2024): adg1094, 10.1126/sciimmunol.adg1094. PMC11093588 38640253

[197] D. Ostroumov , S. Duong , J. Wingerath , et al., “Transcriptome Profiling Identifies TIGIT as a Marker of T‐Cell Exhaustion in Liver Cancer,” Hepatology 73, no. 4 (2021): 1399, 10.1002/hep.31466. 32716559

[198] H. Zhang , Y. Song , H. Yang , et al., “Tumor Cell‐Intrinsic Tim‐3 Promotes Liver Cancer via NF‐κB/IL‐6/STAT3 Axis,” Oncogene 37, no. 18 (2018): 2456–2468, 10.1038/s41388-018-0140-4. 29449693

[199] E. Y. Tan , P. Danpanichkul , J. N. Yong , et al., “Liver Cancer in 2021: Global Burden of Disease Study,” Journal of Hepatology 82, no. 5 (2025): 851, 10.1016/j.jhep.2024.10.031. 39481652

[200] P. Fisicaro , V. Barili , B. Montanini , et al., “Targeting Mitochondrial Dysfunction Can Restore Antiviral Activity of Exhausted HBV‐Specific CD8 T Cells in Chronic Hepatitis B,” Nature Medicine 23, no. 3 (2017): 327–336, 10.1038/nm.4275. 28165481

[201] Y. Cheng , B. Gunasegaran , H. D. Singh , et al., “Non‐Terminally Exhausted Tumor‐Resident Memory HBV‐Specific T Cell Responses Correlate with Relapse‐Free Survival in Hepatocellular Carcinoma,” Immunity 54, no. 8 (2021): 1825–1840, 10.1016/j.immuni.2021.06.013. 34270940

[202] V. Barili , P. Fisicaro , B. Montanini , et al., “Targeting p53 and Histone Methyltransferases Restores Exhausted CD8+ T Cells in HCV Infection,” Nature Communications 11, no. 1 (2020): 604, 10.1038/s41467-019-14137-7. PMC6992697 32001678

[203] B. S. Song , J. S. Moon , J. Tian , et al., “Mitoribosomal Defects Aggravate Liver Cancer via Aberrant Glycolytic Flux and T Cell Exhaustion,” Journal for ImmunoTherapy of Cancer 10, no. 5 (2022): 004337. 10.1136/jitc-2021-004337 PMC9114962 35580931

[204] A. de Jong , M. I. van Poelgeest , J. M. van der Hulst , et al., “Human Papillomavirus Type 16‐Positive Cervical Cancer Is Associated With Impaired CD4+ T‐Cell Immunity against Early Antigens E2 and E6,” Cancer Research 64, no. 15 (2004): 5449–5455, 10.1158/0008-5472.CAN-04-0831. 15289354

[205] M. J. Welters , G. G. Kenter , S. J. Piersma , et al., “Induction of Tumor‐Specific CD4^+^ and CD8^+^ T‐Cell Immunity in Cervical Cancer Patients by a Human Papillomavirus Type 16 E6 and E7 Long Peptides Vaccine,” Clinical Cancer Research 14, no. 1 (2008): 178–187, 10.1158/1078-0432.CCR-07-1880. 18172269

[206] P. J. de Vos van Steenwijk , M. Heusinkveld , T. H. Ramwadhdoebe , et al., “An Unexpectedly Large Polyclonal Repertoire of HPV‐Specific T Cells Is Poised for Action in Patients With Cervical Cancer,” Cancer Research 70, no. 7 (2010): 2707–2717, 10.1158/0008-5472.CAN-09-4299. 20233872

[207] G. Cao , J. Yue , Y. Ruan , et al., “Single‐Cell Dissection of Cervical Cancer Reveals Key Subsets of the Tumor Immune Microenvironment,” EMBO Journal 42, no. 16 (2023): 110757, 10.15252/embj.2022110757. PMC10425846 37427448

[208] P. Xia , J. Zhou , R. Shen , and D. Wang , “Deciphering the Cellular and Molecular Landscape of Cervical Cancer Progression through Single‐Cell and Spatial Transcriptomics,” Npj Precision Oncology 9, no. 1 (2025): 158, 10.1038/s41698-025-00948-z. 40437003 PMC12120119

[209] X. Zhu , Y. Feng , P. Fan , et al., “Increased co‐expression of 4‐1BB with PD‐1 on CD8^+^ Tumor‐infiltrating Lymphocytes Is Associated With Improved Prognosis and Immunotherapy Response in Cervical Cancer,” Frontiers in Oncology 14 (2024): 1381381, 10.3389/fonc.2024.1381381. 38756662 PMC11096482

[210] J. M. Rojas‐Diaz , F. Solorzano‐Ibarra , N. T. Garcia‐Barrientos , et al., “Uncovering the Expression Pattern of the Costimulatory Receptors ICOS, 4‐1BB, and OX‐40 in Exhausted Peripheral and Tumor‐Infiltrating Natural Killer Cells from Patients with Cervical Cancer,” International Journal of Molecular Sciences 25, no. 16 (2024): 8775. 39201462 10.3390/ijms25168775 PMC11354483

[211] Y. Z. Lin , C. H. Liu , W. R. Wu , et al., “Memory‐promoting Function of miR‐379‐5p Attenuates CD8 + T Cell Exhaustion by Targeting Immune Checkpoints,” Journal for ImmunoTherapy of Cancer 13, no. 4 (2025): 010363, 10.1136/jitc-2024-010363. PMC11997822 40221151

[212] J. R. Giles , S. F. Ngiow , S. Manne , et al., “Shared and Distinct Biological Circuits in Effector, Memory and Exhausted CD8+ T Cells Revealed by Temporal Single‐cell Transcriptomics and Epigenetics,” Nature Immunology 23, no. 11 (2022): 1600–1613, 10.1038/s41590-022-01338-4. 36271148 PMC10408358

[213] Z. Zhang , L. Chen , H. Chen , et al., “Pan‐cancer Landscape of T‐cell Exhaustion Heterogeneity within the Tumor Microenvironment Revealed a Progressive Roadmap of Hierarchical Dysfunction Associated with Prognosis and Therapeutic Efficacy,” EBioMedicine 83 (2022): 104207, 10.1016/j.ebiom.2022.104207. 35961204 PMC9382263

[214] S. Naulaerts , A. Datsi , D. M. Borras , et al., “Multiomics and Spatial Mapping Characterizes human CD8 + T Cell States in Cancer,” Science Translational Medicine 15, no. 691 (2023): add1016, 10.1126/scitranslmed.add1016. 37043555

[215] M. Stachowiak , W. J. Becker , P. B. Olkhanud , et al., “Cancer Cells Accelerate Exhaustion of Persistently Activated Mouse CD^4+^ T Cells,” Oncoimmunology 14, no. 1 (2025): 2521392. 40536473 10.1080/2162402X.2025.2521392 PMC12184148

[216] C. C. Balanca , A. Salvioni , C. M. Scarlata , et al., “PD‐1 Blockade Restores Helper Activity of Tumor‐infiltrating, Exhausted PD‐1hiCD39+ CD4 T Cells,” JCI Insight 6, no. 2 (2021), 10.1172/jci.insight.142513. PMC7934837 33332284

[217] H. R. Kim , H. J. Park , J. Son , et al., “Tumor Microenvironment Dictates Regulatory T Cell Phenotype: Upregulated Immune Checkpoints Reinforce Suppressive Function,” Journal for ImmunoTherapy of Cancer 7, no. 1 (2019): 339, 10.1186/s40425-019-0785-8. 31801611 PMC6894345

[218] C. Li , P. Jiang , S. Wei , X. Xu , and J. Wang , “Regulatory T Cells in Tumor Microenvironment: New Mechanisms, Potential Therapeutic Strategies and Future Prospects,” Molecular Cancer 19, no. 1 (2020): 116, 10.1186/s12943-020-01234-1. 32680511 PMC7367382

[219] R. Kattelus , I. Starskaia , M. Linden , et al., “Phenotypic Profiling of human Induced Regulatory T Cells at Early Differentiation: Insights into Distinct Immunosuppressive Potential,” Cellular and Molecular Life Sciences 81, no. 1 (2024): 399, 10.1007/s00018-024-05429-3. 39264416 PMC11393232

[220] L. E. Lucca and M. Dominguez‐Villar , “Modulation of Regulatory T Cell Function and Stability by Co‐Inhibitory Receptors,” Nature Reviews Immunology 20, no. 11 (2020): 680–693, 10.1038/s41577-020-0296-3. 32269380

[221] S. Kurtulus , K. Sakuishi , S. F. Ngiow , et al., “TIGIT Predominantly Regulates the Immune Response via Regulatory T Cells,” Journal of Clinical Investigation 125, no. 11 (2015): 4053, 10.1172/JCI81187. 26413872 PMC4639980

[222] A. G. Jarnicki , J. Lysaght , S. Todryk , and K. H. Mills , “Suppression of Antitumor Immunity by IL‐10 and TGF‐β‐Producing T Cells Infiltrating the Growing Tumor: Influence of Tumor Environment on the Induction of CD4^+^ and CD8^+^ Regulatory T Cells,” Journal of Immunology 177, no. 2 (2006): 896, 10.4049/jimmunol.177.2.896. 16818744

[223] R. Saleh and E. Elkord , “FoxP3+ T Regulatory Cells in Cancer: Prognostic Biomarkers and Therapeutic Targets,” Cancer Letters 490 (2020): 174–185, 10.1016/j.canlet.2020.07.022. 32721551

[224] C. Lamarche , K. Ward‐Hartstonge , T. Mi , et al., “Tonic‐signaling Chimeric Antigen Receptors Drive human Regulatory T Cell Exhaustion,” Proceedings of the National Academy of Sciences of the United States of America 120, no. 14 (2023): 2219086120, 10.1073/pnas.2219086120. PMC10083618 36972454

[225] D. J. Verdon , M. Mulazzani , and M. R. Jenkins , “Cellular and Molecular Mechanisms of CD8+ T Cell Differentiation, Dysfunction and Exhaustion,” International Journal of Molecular Sciences 21, no. 19 (2020): 7357. 33027962 10.3390/ijms21197357 PMC7582856

[226] M. Ukita , J. Hamanishi , H. Yoshitomi , et al., “CXCL13‐Producing CD^4+^ T Cells Accumulate in the Early Phase of Tertiary Lymphoid Structures in Ovarian Cancer,” JCI Insight 7, no. 12 (2022): 157215. 10.1172/jci.insight.157215 PMC9309049 35552285

[227] J. Nie , S. Zhang , Y. Guo , et al., “Mapping of the T‐Cell Landscape of Biliary Tract Cancer Unravels Anatomic Subtype‐Specific Heterogeneity,” Cancer Research 85, no. 4 (2025): 704–722, 10.1158/0008-5472.CAN-24-1173. 39570809

[228] D. Chen , Y. Guo , J. Jiang , et al., “γδ T Cell Exhaustion: Opportunities for Intervention,” Journal of Leukocyte Biology 112, no. 6 (2022): 1669–1676, 10.1002/JLB.5MR0722-777R. 36000310 PMC9804355

[229] T. Wang , H. Wang , R. Lv , C. Wen , M. Wang , and L. Huang , “The Role of γδ T Cells and CAR‐γδ T Cell Therapy in Autoimmune Diseases,” Autoimmunity Reviews 24, no. 10 (2025): 103883, 10.1016/j.autrev.2025.103883. 40681068

[230] B. S. Reis , P. W. Darcy , I. Z. Khan , et al., “TCR‐Vγδ Usage Distinguishes Protumor From Antitumor Intestinal γδ T Cell Subsets,” Science 377, no. 6603 (2022): 276, 10.1126/science.abj8695. 35857588 PMC9326786

[231] S. Fattori , L. Gorvel , S. Granjeaud , et al., “Quantification of Immune Variables from Liquid Biopsy in Breast Cancer Patients Links Vδ2+ γδ T Cell Alterations with Lymph Node Invasion,” Cancers 13, no. 3 (2021): 441. 33503843 10.3390/cancers13030441 PMC7865589

[232] J. P. Cerapio , M. Perrier , C. C. Balanca , et al., “Phased Differentiation of γδ T and T CD8 Tumor‐Infiltrating Lymphocytes Revealed by Single‐Cell Transcriptomics of Human Cancers,” Oncoimmunology 10, no. 1 (2021): 1939518. 34721945 10.1080/2162402X.2021.1939518 PMC8555559

[233] M. Kronenberg and T. Riffelmacher , “Defenders or Defectors: Mucosal‐Associated Invariant T Cells in Autoimmune Diseases,” Current Opinion in Immunology 93 (2025): 102542, 10.1016/j.coi.2025.102542. 40020256 PMC11908677

[234] X. He and C. Xu , “PD‐1: A Driver or Passenger of T Cell Exhaustion?,” Molecular Cell 77, no. 5 (2020): 930–931, 10.1016/j.molcel.2020.02.013. 32142689

[235] X. Zhang , J. C. Schwartz , X. Guo , et al., “Structural and Functional Analysis of the Costimulatory Receptor Programmed Death‐1,” Immunity 20, no. 3 (2004): 337–347, 10.1016/S1074-7613(04)00051-2. 15030777

[236] T. Okazaki , Y. Iwai , and T. Honjo , “New Regulatory Co‐Receptors: Inducible co‐Stimulator and PD‐1,” Current Opinion in Immunology 14, no. 6 (2002): 779–782, 10.1016/S0952-7915(02)00398-9. 12413529

[237] A. H. Sharpe and K. E. Pauken , “The Diverse Functions of the PD1 Inhibitory Pathway,” Nature Reviews Immunology 18, no. 3 (2018): 153–167, 10.1038/nri.2017.108. 28990585

[238] L. Simula , Y. Antonucci , G. Scarpelli , et al., “PD‐1‐Induced T Cell Exhaustion Is Controlled by a Drp1‐Dependent Mechanism,” Molecular Oncology 16, no. 1 (2022): 188, 10.1002/1878-0261.13103. 34535949 PMC8732338

[239] G. J. Freeman , A. J. Long , Y. Iwai , et al., “Engagement of the Pd‐1 Immunoinhibitory Receptor by a Novel B7 Family Member Leads to Negative Regulation of Lymphocyte Activation,” The Journal of Experimental Medicine 192, no. 7 (2000): 1027–1034, 10.1084/jem.192.7.1027. 11015443 PMC2193311

[240] D. Gumber and L. D. Wang , “Improving CAR‐T Immunotherapy: Overcoming the Challenges of T Cell Exhaustion,” EBioMedicine 77 (2022): 103941, 10.1016/j.ebiom.2022.103941. 35301179 PMC8927848

[241] N. Patsoukis , J. Brown , V. Petkova , F. Liu , L. Li , and V. A. Boussiotis , “Selective Effects of PD‐1 on Akt and Ras Pathways Regulate Molecular Components of the Cell Cycle and Inhibit T Cell Proliferation,” Science Signaling 5, no. 230 (2012): ra46, 10.1126/scisignal.2002796. 22740686 PMC5498435

[242] K. Bardhan , T. Anagnostou , and V. A. Boussiotis , “The PD1:PD‐L1/2 Pathway From Discovery to Clinical Implementation,” Frontiers in Immunology 7 (2016): 550, 10.3389/fimmu.2016.00550. 28018338 PMC5149523

[243] A. O. Kamphorst , A. Wieland , T. Nasti , et al., “Rescue of Exhausted CD8 T Cells by PD‐1–Targeted Therapies Is CD28‐Dependent,” Science 355, no. 6332 (2017): 1423–1427, 10.1126/science.aaf0683. 28280249 PMC5595217

[244] L. Carter , L. A. Fouser , J. Jussif , et al., “PD‐1:PD‐L Inhibitory Pathway Affects both CD4^+^ and CD8^+^ T Cells and Is Overcome by IL‐2,” European Journal of Immunology 32, no. 3 (2002): 634, 10.1002/1521-4141(200203)32:3<634::AID-IMMU634>3.0.CO;2-9. 11857337

[245] N. Selenko‐Gebauer , O. Majdic , A. Szekeres , et al., “B7‐H1 (Programmed Death‐1 Ligand) on Dendritic Cells Is Involved in the Induction and Maintenance of T Cell Anergy,” The Journal of Immunology 170, no. 7 (2003): 3637, 10.4049/jimmunol.170.7.3637. 12646628

[246] E. J. Wherry and M. Kurachi , “Molecular and Cellular Insights into T Cell Exhaustion,” Nature Reviews Immunology 15, no. 8 (2015): 486–499, 10.1038/nri3862. PMC4889009 26205583

[247] J. Ma , B. Zheng , S. Goswami , et al., “PD1Hi CD8+ T Cells Correlate With Exhausted Signature and Poor Clinical Outcome in Hepatocellular Carcinoma,” Journal for ImmunoTherapy of Cancer 7, no. 1 (2019): 331, 10.1186/s40425-019-0814-7. 31783783 PMC6884778

[248] M. K. Callahan , M. A. Postow , and J. D. Wolchok , “Targeting T Cell Co‐Receptors for Cancer Therapy,” Immunity 44, no. 5 (2016): 1069–1078, 10.1016/j.immuni.2016.04.023. 27192570

[249] I. L. Mercier , J. L. Lines , and R. J. Noelle , “Beyond CTLA‐4 and PD‐1, the Generation Z of Negative Checkpoint Regulators,” Frontiers in Immunology 6 (2015): 418, 10.3389/fimmu.2015.00418. 26347741 PMC4544156

[250] S. H. Baumeister , G. J. Freeman , G. Dranoff , and A. H. Sharpe , “Coinhibitory Pathways in Immunotherapy for Cancer,” Annual Review of Immunology 34 (2016): 539–573, 10.1146/annurev-immunol-032414-112049. 26927206

[251] D. Rong , Y. Wang , L. Liu , et al., “GLIS1 Intervention Enhances Anti‐PD1 Therapy for Hepatocellular Carcinoma by Targeting SGK1‐STAT3‐PD1 Pathway,” Journal for ImmunoTherapy of Cancer 11, no. 2 (2023): 005126, 10.1136/jitc-2022-005126. PMC9930610 36787938

[252] Y. Sawada , T. Yoshikawa , M. Shimomura , T. Iwama , I. Endo , and T. Nakatsura , “Programmed Death‐1 Blockade Enhances the Antitumor Effects of Peptide Vaccine‐Induced Peptide‐Specific Cytotoxic T Lymphocytes,” International Journal of Oncology 46, no. 1 (2015): 28–36, 10.3892/ijo.2014.2737. 25354479 PMC4238729

[253] K. Pang , Z. D. Shi , L. Y. Wei , et al., “Research Progress of Therapeutic Effects and Drug Resistance of Immunotherapy Based on PD‐1/PD‐L1 Blockade,” Drug Resistance Updates 66 (2023): 100907, 10.1016/j.drup.2022.100907. 36527888

[254] K. J. Scalapino and D. I. Daikh , “CTLA‐4: a Key Regulatory Point in the Control of Autoimmune Disease,” Immunological Reviews 223 (2008): 143, 10.1111/j.1600-065X.2008.00639.x. 18613834

[255] P. A. van der Merwe , D. L. Bodian , S. Daenke , P. Linsley , and S. J. Davis , “CD80 (B7‐1) Binds both CD28 and CTLA‐4 With a Low Affinity and Very Fast Kinetics,” The Journal of Experimental Medicine 185, no. 3 (1997): 393, 10.1084/jem.185.3.393. 9053440 PMC2196039

[256] J. F. Brunet , F. Denizot , M. F. Luciani , et al., “A New Member of the Immunoglobulin Superfamily—CTLA‐4,” Nature 328, no. 6127 (1987): 267, 10.1038/328267a0. 3496540

[257] S. M. Krummey and M. L. Ford , “Braking Bad: Novel Mechanisms of CTLA‐4 Inhibition of T Cell Responses,” American Journal of Transplantation 14, no. 12 (2014): 2685, 10.1111/ajt.12938. 25387592 PMC4364523

[258] A. D. Apol , A. A. Winckelmann , R. B. Duus , J. Bukh , and N. Weis , “The Role of CTLA‐4 in T Cell Exhaustion in Chronic Hepatitis B Virus Infection,” Viruses 15, no. 5 (2023): 1141. 37243227 10.3390/v15051141 PMC10223466

[259] X. Wang , Q. He , H. Shen , X. J. Lu , and B. Sun , “Genetic and Phenotypic Difference in CD8 + T Cell Exhaustion between Chronic Hepatitis B Infection and Hepatocellular Carcinoma,” Journal of Medical Genetics 56, no. 1 (2019): 18, 10.1136/jmedgenet-2018-105267. 29666149 PMC6327916

[260] C. Y. Hsu , T. M. Muhammed , S. Uthirapathy , et al., “CTLA‐4 Blockade in Ovarian Cancer Immunotherapy: Mechanisms and Clinical Strategies,” Seminars in Oncology 52, no. 4 (2025): 152370, 10.1016/j.seminoncol.2025.152370. 40482550

[261] H. F. Tsai and P. N. Hsu , “Cancer Immunotherapy by Targeting Immune Checkpoints: Mechanism of T Cell Dysfunction in Cancer Immunity and New Therapeutic Targets,” Journal of Biomedical Science 24, no. 1 (2017): 35, 10.1186/s12929-017-0341-0. 28545567 PMC5445514

[262] J. Larkin , V. Chiarion‐Sileni , R. Gonzalez , et al., “Combined Nivolumab and Ipilimumab or Monotherapy in Untreated Melanoma,” New England Journal of Medicine 373, no. 1 (2015): 23, 10.1056/NEJMoa1504030. 26027431 PMC5698905

[263] F. Bengsch , D. M. Knoblock , A. Liu , F. McAllister , and G. L. Beatty , “CTLA‐4/CD80 Pathway Regulates T Cell Infiltration into Pancreatic Cancer,” Cancer Immunology, Immunotherapy 66, no. 12 (2017): 1609, 10.1007/s00262-017-2053-4. 28856392 PMC5677559

[264] X. Chu , W. Tian , Z. Wang , J. Zhang , and R. Zhou , “Co‐Inhibition of TIGIT and PD‐1/PD‐L1 in Cancer Immunotherapy: Mechanisms and Clinical Trials,” Molecular Cancer 22, no. 1 (2023): 93, 10.1186/s12943-023-01800-3. 37291608 PMC10249258

[265] R. J. Johnston , L. Comps‐Agrar , J. Hackney , et al., “The Immunoreceptor TIGIT Regulates Antitumor and Antiviral CD8 + T Cell Effector Function,” Cancer Cell 26, no. 6 (2014): 923, 10.1016/j.ccell.2014.10.018. 25465800

[266] S. Liu , H. Zhang , M. Li , et al., “Recruitment of Grb2 and SHIP1 by the ITT‐Like Motif of TIGIT Suppresses Granule Polarization and Cytotoxicity of NK Cells,” Cell Death & Differentiation 20, no. 3 (2013): 456, 10.1038/cdd.2012.141. 23154388 PMC3569986

[267] X. Guan , R. Hu , Y. Choi , et al., “Anti‐TIGIT Antibody Improves PD‐L1 Blockade through Myeloid and Treg Cells,” Nature 627, no. 8004 (2024): 646, 10.1038/s41586-024-07121-9. 38418879 PMC11139643

[268] C. R. Hartigan , K. P. Tong , D. Liu , S. J. Laurie , and M. L. Ford , “TIGIT Agonism Alleviates Costimulation Blockade‐resistant Rejection in a Regulatory T Cell–Dependent Manner,” American Journal of Transplantation 23, no. 2 (2023): 180, 10.1016/j.ajt.2022.12.011. 36695691 PMC10062175

[269] N. Stanietsky , H. Simic , J. Arapovic , et al., “The Interaction of TIGIT with PVR and PVRL2 Inhibits human NK Cell Cytotoxicity,” Proceedings of the National Academy of Sciences 106, no. 42 (2009): 17858, 10.1073/pnas.0903474106. PMC2764881 19815499

[270] R. Liang , X. Zhu , T. Lan , et al., “TIGIT Promotes CD8+T Cells Exhaustion and Predicts Poor Prognosis of Colorectal Cancer,” Cancer Immunology, Immunotherapy 70, no. 10 (2021): 2781, 10.1007/s00262-021-02886-8. 33634371 PMC10992182

[271] A. Reches , Y. Ophir , N. Stein , et al., “Nectin4 is a Novel TIGIT Ligand Which Combines Checkpoint Inhibition and Tumor Specificity,” Journal for ImmunoTherapy of Cancer 8, no. 1 (2020): 000266, 10.1136/jitc-2019-000266. PMC7279670 32503945

[272] C. Gur , Y. Ibrahim , B. Isaacson , et al., “Binding of the Fap2 Protein of Fusobacterium nucleatum to Human Inhibitory Receptor TIGIT Protects Tumors from Immune Cell Attack,” Immunity 42, no. 2 (2015): 344, 10.1016/j.immuni.2015.01.010. 25680274 PMC4361732

[273] Z. Ge , M. P. Peppelenbosch , D. Sprengers , and J. Kwekkeboom , “TIGIT, the Next Step Towards Successful Combination Immune Checkpoint Therapy in Cancer,” Frontiers in Immunology 12 (2021): 699895, 10.3389/fimmu.2021.699895. 34367161 PMC8339559

[274] L. Wu , L. Mao , J. F. Liu , et al., “Blockade of TIGIT/CD155 Signaling Reverses T‐Cell Exhaustion and Enhances Antitumor Capability in Head and Neck Squamous Cell Carcinoma,” Cancer Immunology Research 7, no. 10 (2019): 1700, 10.1158/2326-6066.CIR-18-0725. 31387897

[275] X. Chen , L. Xue , X. Ding , et al., “An Fc‐Competent Anti‐Human TIGIT Blocking Antibody Ociperlimab (BGB‐A1217) Elicits Strong Immune Responses and Potent Anti‐Tumor Efficacy in Pre‐Clinical Models,” Frontiers in Immunology 13 (2022): 828319, 10.3389/fimmu.2022.828319. 35273608 PMC8902820

[276] S. A. Minnie , R. D. Kuns , K. H. Gartlan , et al., “Myeloma Escape after Stem Cell Transplantation Is a Consequence of T‐Cell Exhaustion and Is Prevented by TIGIT Blockade,” Blood 132, no. 16 (2018): 1675, 10.1182/blood-2018-01-825240. 30154111

[277] Y. Jo , H. S. Jin , and Y. Park , “No More LAGging behind PD‐1: Uncovering the Unique Role of LAG‐3 in T‐Cell Exhaustion,” Cellular & Molecular Immunology 21, no. 12 (2024): 1351, 10.1038/s41423-024-01227-w. 39433966 PMC11607349

[278] B. Huard , P. Prigent , M. Tournier , D. Bruniquel , and F. Triebel , “CD4/major Histocompatibility Complex Class II Interaction Analyzed with CD4‐ and Lymphocyte Activation Gene‐3 (LAG‐3)‐Ig Fusion Proteins,” European Journal of Immunology 25, no. 9 (1995): 2718, 10.1002/eji.1830250949. 7589152

[279] T. Maruhashi , D. Sugiura , I. M. Okazaki , et al., “Binding of LAG‐3 to Stable Peptide‐MHC Class II Limits T Cell Function and Suppresses Autoimmunity and Anti‐Cancer Immunity,” Immunity 55, no. 5 (2022): 912, 10.1016/j.immuni.2022.03.013. 35413245

[280] J. L. Silberstein , J. Du , K. W. Chan , et al., “Structural Insights Reveal Interplay between LAG‐3 Homodimerization, Ligand Binding, and Function,” Proceedings of the National Academy of Sciences U S A 121, no. 12 (2024): 2310866121, 10.1073/pnas.2310866121. PMC10962948 38483996

[281] L. Pedersen , L. L. Eriksen , F. H. Brix , et al., “The FGL‐1/LAG‐3 Axis Is Associated With Disease Course in Alcohol‐Associated Hepatitis: A Preliminary Report,” Journal of Clinical and Experimental Hepatology 15, no. 1 (2025): 102424, 10.1016/j.jceh.2024.102424. 39553834 PMC11567029

[282] Y. Lu , A. Yang , C. Quan , et al., “A Single‐Cell Atlas of the Multicellular Ecosystem of Primary and Metastatic Hepatocellular Carcinoma,” Nature Communications 13, no. 1 (2022): 4594, 10.1038/s41467-022-32283-3. PMC9357016 35933472

[283] J. Wang , M. F. Sanmamed , I. Datar , et al., “Fibrinogen‐Like Protein 1 Is a Major Immune Inhibitory Ligand of LAG‐3,” Cell 176, no. 1‐2 (2019): 334, 10.1016/j.cell.2018.11.010. 30580966 PMC6365968

[284] L. Cai , Y. Li , J. Tan , L. Xu , and Y. Li , “Correction: Targeting LAG‐3, TIM‐3, and TIGIT for Cancer Immunotherapy,” Journal of Hematology & Oncology 16, no. 1 (2023): 105, 10.1186/s13045-023-01503-8. 37773132 PMC10543833

[285] L. Chocarro , E. Blanco , H. Arasanz , et al., “Clinical Landscape of LAG‐3‐Targeted Therapy,” Immuno‐Oncology and Technology 14 (2022): 100079, 10.1016/j.iotech.2022.100079. PMC9216443 35755891

[286] P. Schoffski , D. S. W. Tan , M. Martin , et al., “Phase I/II Study of the LAG‐3 Inhibitor Ieramilimab (LAG525) ± Anti‐PD‐1 Spartalizumab (PDR001) in Patients With Advanced Malignancies,” Journal for ImmunoTherapy of Cancer 10, no. 2 (2022): 003776, 10.1136/jitc-2021-003776. PMC8883259 35217575

[287] M. R. Sullivan , G. S. Ugolini , S. Sarkar , et al., “Quantifying the Efficacy of Checkpoint Inhibitors on CD8^+^ Cytotoxic T Cells for Immunotherapeutic Applications via Single‐Cell Interaction,” Cell Death & Disease 11, no. 11 (2020): 979, 10.1038/s41419-020-03173-7. 33188167 PMC7666200

[288] E. Garralda , A. Sukari , N. J. Lakhani , et al., “A First‐In‐Human Study of the Anti‐LAG‐3 Antibody Favezelimab Plus Pembrolizumab in Previously Treated, Advanced Microsatellite Stable Colorectal Cancer,” ESMO Open 7, no. 6 (2022): 100639. 36493599 10.1016/j.esmoop.2022.100639 PMC9832734

[289] L. P. Andrews , A. E. Marciscano , C. G. Drake , and D. A. Vignali , “LAG 3 (CD 223) as a Cancer Immunotherapy Target,” Immunological Reviews 276, no. 1 (2017): 80, 10.1111/imr.12519. 28258692 PMC5338468

[290] S. F. Ngiow , S. Manne , Y. J. Huang , et al., “LAG‐3 Sustains TOX Expression and Regulates the CD94/NKG2‐Qa‐1b Axis to Govern Exhausted CD8 T Cell NK Receptor Expression and Cytotoxicity,” Cell 187, no. 16 (2024): 4336, 10.1016/j.cell.2024.07.018. 39121847 PMC11337978

[291] S. R. Woo , M. E. Turnis , M. V. Goldberg , et al., “Immune Inhibitory Molecules LAG‐3 and PD‐1 Synergistically Regulate T‐cell Function to Promote Tumoral Immune Escape,” Cancer Research 72, no. 4 (2012): 917, 10.1158/0008-5472.CAN-11-1620. 22186141 PMC3288154

[292] X. Mao , M. T. Ou , S. S. Karuppagounder , et al., “Pathological α‐Synuclein Transmission Initiated by Binding Lymphocyte‐Activation Gene 3,” Science 353, no. 6307 (2016). 10.1126/science.aah3374 PMC5510615 27708076

[293] R. B. Jones , L. C. Ndhlovu , J. D. Barbour , et al., “Tim‐3 Expression Defines a Novel Population of Dysfunctional T Cells with Highly Elevated Frequencies in Progressive HIV‐1 Infection,” The Journal of Experimental Medicine 205, no. 12 (2008): 2763, 10.1084/jem.20081398. 19001139 PMC2585847

[294] S. Takamura , S. Tsuji‐Kawahara , H. Yagita , et al., “Premature Terminal Exhaustion of Friend Virus‐Specific Effector CD8+ T Cells by Rapid Induction of Multiple Inhibitory Receptors,” Journal of Immunology 184, no. 9 (2010): 4696, 10.4049/jimmunol.0903478. 20351188

[295] K. Sakuishi , L. Apetoh , J. M. Sullivan , B. R. Blazar , V. K. Kuchroo , and A. C. Anderson , “Targeting Tim‐3 and PD‐1 Pathways to Reverse T Cell Exhaustion and Restore Anti‐Tumor Immunity,” Journal of Experimental Medicine 207, no. 10 (2010): 2187, 10.1084/jem.20100643. 20819927 PMC2947065

[296] Y. Wolf , A. C. Anderson , and V. K. Kuchroo , “TIM3 Comes of Age as an Inhibitory Receptor,” Nature Reviews Immunology 20, no. 3 (2020): 173, 10.1038/s41577-019-0224-6. PMC7327798 31676858

[297] K. O. Dixon , G. F. Lahore , and V. K. Kuchroo , “Beyond T Cell Exhaustion: TIM‐3 Regulation of Myeloid Cells,” Science Immunology 9, no. 93 (2024): adf2223, 10.1126/sciimmunol.adf2223. 38457514

[298] J. Jiang , M. S. Jin , F. Kong , et al., “Decreased Galectin‐9 and Increased Tim‐3 Expression Are Related to Poor Prognosis in Gastric Cancer,” PLoS ONE 8, no. 12 (2013): 81799, 10.1371/journal.pone.0081799. PMC3858245 24339967

[299] A. S. Japp , M. A. Kursunel , S. Meier , et al., “Dysfunction of PSA‐Specific CD8+ T Cells in Prostate Cancer Patients Correlates with CD38 and Tim‐3 Expression,” Cancer Immunology, Immunotherapy 64, no. 11 (2015): 1487, 10.1007/s00262-015-1752-y. 26289091 PMC11028650

[300] K. Sakuishi , S. F. Ngiow , J. M. Sullivan , et al., “TIM3 + FOXP3 + Regulatory T Cells Are Tissue‐Specific Promoters of T‐Cell Dysfunction in Cancer,” Oncoimmunology 2, no. 4 (2013): 23849, 10.4161/onci.23849. PMC3654601 23734331

[301] R. Yang , L. Sun , C. F. Li , et al., “Galectin‐9 Interacts With PD‐1 and TIM‐3 to Regulate T Cell Death and Is a Target for Cancer Immunotherapy,” Nature Communications 12, no. 1 (2021): 832, 10.1038/s41467-021-21099-2. PMC7864927 33547304

[302] Y. Zhang , P. Cai , L. Li , et al., “Co‐Expression of TIM‐3 and CEACAM1 Promotes T Cell Exhaustion in Colorectal Cancer Patients,” International Immunopharmacology 43 (2017): 210, 10.1016/j.intimp.2016.12.024. 28038383

[303] Y. H. Huang , C. Zhu , Y. Kondo , et al., “CEACAM1 Regulates TIM‐3‐Mediated Tolerance and Exhaustion,” Nature 517, no. 7534 (2015): 386, 10.1038/nature13848. 25363763 PMC4297519

[304] I. Ausejo‐Mauleon , S. Labiano , D. de la Nava , et al., “TIM‐3 Blockade in Diffuse Intrinsic Pontine Glioma Models Promotes Tumor Regression and Antitumor Immune Memory,” Cancer Cell 41, no. 11 (2023): 1911, 10.1016/j.ccell.2023.09.001. 37802053 PMC10644900

[305] J. Gong , A. Chehrazi‐Raffle , S. Reddi , and R. Salgia , “Development of PD‐1 and PD‐L1 Inhibitors as a Form of Cancer Immunotherapy: A Comprehensive Review of Registration Trials and Future Considerations,” Journal for ImmunoTherapy of Cancer 6, no. 1 (2018): 8, 10.1186/s40425-018-0316-z. 29357948 PMC5778665

[306] R. V. Parry , J. M. Chemnitz , K. A. Frauwirth , et al., “CTLA‐4 and PD‐1 Receptors Inhibit T‐Cell Activation by Distinct Mechanisms,” Molecular and Cellular Biology 25, no. 21 (2005): 9543, 10.1128/MCB.25.21.9543-9553.2005. 16227604 PMC1265804

[307] S. Xu , N. Zhang , M. L. Rinne , H. Sun , and A. M. Stein , “Sabatolimab (MBG453) Model‐Informed Drug Development for Dose Selection in Patients With Myelodysplastic Syndrome/Acute Myeloid Leukemia and Solid Tumors,” CPT: Pharmacometrics & Systems Pharmacology 12, no. 11 (2023): 1653, 10.1002/psp4.12962. 37186155 PMC10681456

[308] D. Zhang , F. Jiang , R. Zaynagetdinov , et al., “Identification and Characterization of M6903, an Antagonistic Anti‐TIM‐3 Monoclonal Antibody,” Oncoimmunology 9, no. 1 (2020): 1744921. 32313722 10.1080/2162402X.2020.1744921 PMC7153820

[309] S. Ma , Y. Tian , J. Peng , et al., “Identification of a Small‐Molecule Tim‐3 Inhibitor to Potentiate T Cell–mediated Antitumor Immunotherapy in Preclinical Mouse Models,” Science Translational Medicine 15, no. 722 (2023): adg6752, 10.1126/scitranslmed.adg6752. 37967204

[310] B. Artegiani , A. M. de Jesus Domingues , S. Bragado Alonso , et al., “Tox: A Multifunctional Transcription Factor and Novel Regulator of Mammalian Corticogenesis,” The EMBO Journal 34, no. 7 (2015): 896–910, 10.15252/embj.201490061. 25527292 PMC4388598

[311] X. Wang , Q. He , H. Shen , et al., “TOX Promotes the Exhaustion of Antitumor CD8+ T Cells by Preventing PD1 Degradation in Hepatocellular Carcinoma,” J Hepatol 71, no. 4 (2019): 731–741. 31173813 10.1016/j.jhep.2019.05.015

[312] L. P. Andrews , S. C. Butler , J. Cui , et al., “LAG‐3 and PD‐1 Synergize on CD8+ T Cells to Drive T Cell Exhaustion and Hinder Autocrine IFN‐γ‐Dependent Anti‐Tumor Immunity,” Cell 187, no. 16 (2024): 4355–4372, 10.1016/j.cell.2024.07.016. 39121848 PMC11323044

[313] W. S. Lee , H. Yang , H. J. Chon , and C. Kim , “Combination of Anti‐Angiogenic Therapy and Immune Checkpoint Blockade Normalizes Vascular‐Immune Crosstalk to Potentiate Cancer Immunity,” Experimental & Molecular Medicine 52, no. 9 (2020): 1475–1485, 10.1038/s12276-020-00500-y. 32913278 PMC8080646

[314] H. Seo , E. Gonzalez‐Avalos , W. Zhang , et al., “BATF and IRF4 Cooperate to Counter Exhaustion in Tumor‐Infiltrating CAR T Cells,” Nature Immunology 22, no. 8 (2021): 983–995, 10.1038/s41590-021-00964-8. 34282330 PMC8319109

[315] F. Alfei , K. Kanev , M. Hofmann , et al., “TOX Reinforces the Phenotype and Longevity of Exhausted T Cells in Chronic Viral Infection,” Nature 571, no. 7764 (2019): 265–269, 10.1038/s41586-019-1326-9. 31207605

[316] A. C. Scott , F. Dundar , P. Zumbo , et al., “TOX Is a Critical Regulator of Tumour‐Specific T Cell Differentiation,” Nature 571, no. 7764 (2019): 270–274, 10.1038/s41586-019-1324-y. 31207604 PMC7698992

[317] J. Zhang , T. Lyu , Y. Cao , and H. Feng , “Role of TCF‐1 in Differentiation, Exhaustion, and Memory of CD8 + T Cells: A Review,” FASEB Journal 35, no. 5 (2021): 21549, 10.1096/fj.202002566R. 33913198

[318] E. N. Neubert , J. M. DeRogatis , S. A. Lewis , et al., “HMGB2 Regulates the Differentiation and Stemness of Exhausted CD8+ T Cells During Chronic Viral Infection and Cancer,” Nature Communications 14, no. 1 (2023): 5631, 10.1038/s41467-023-41352-0. PMC10499904 37704621

[319] H. Huang , J. Ge , Z. Fang , et al., “Precursor Exhausted CD8+T Cells in Colorectal Cancer Tissues Associated With Patient's Survival and Immunotherapy Responsiveness,” Frontiers in Immunology 15 (2024): 1362140, 10.3389/fimmu.2024.1362140. 38510246 PMC10950923

[320] E. Humblin , I. Korpas , J. Lu , et al., “Sustained CD28 Costimulation Is Required for Self‐Renewal and Differentiation of TCF‐1 + PD‐1 + CD8 T Cells,” Science Immunology 8, no. 86 (2023): adg0878, 10.1126/sciimmunol.adg0878. PMC10805182 37624910

[321] Q. Shan , S. Hu , X. Chen , et al., “Ectopic Tcf1 Expression Instills a Stem‐Like Program in Exhausted CD8+ T Cells to Enhance Viral and Tumor Immunity,” Cellular & Molecular Immunology 18, no. 5 (2021): 1262–1277, 10.1038/s41423-020-0436-5. 32341523 PMC8093427

[322] T. Wu , Y. Ji , E. A. Moseman , et al., “The TCF1‐Bcl6 Axis Counteracts Type I Interferon to Repress Exhaustion and Maintain T Cell Stemness,” Science Immunology 1, no. 6 (2016): aai8593. 10.1126/sciimmunol.aai8593 PMC5179228 28018990

[323] M. Sade‐Feldman , K. Yizhak , S. L. Bjorgaard , et al., “Defining T Cell States Associated with Response to Checkpoint Immunotherapy in Melanoma,” Cell 176, no. 1‐2 (2019): 404, 10.1016/j.cell.2018.12.034. 30633907 PMC6647017

[324] J. Fric , T. Zelante , A. Y. Wong , A. Mertes , H. B. Yu , and P. Ricciardi‐Castagnoli , “NFAT Control of Innate Immunity,” Blood 120, no. 7 (2012): 1380, 10.1182/blood-2012-02-404475. 22611159

[325] A. Schietinger and P. D. Greenberg , “Tolerance and Exhaustion: Defining Mechanisms of T Cell Dysfunction,” Trends in Immunology 35, no. 2 (2014): 51–60, 10.1016/j.it.2013.10.001. 24210163 PMC3946600

[326] F. Macian , C. Garcia‐Rodriguez , and A. Rao , “Gene Expression Elicited by NFAT in the Presence or Absence of Cooperative Recruitment of Fos and Jun,” The EMBO Journal 19, no. 17 (2000): 4783–4795, 10.1093/emboj/19.17.4783. 10970869 PMC302068

[327] K. Man , S. S. Gabriel , Y. Liao , et al., “Transcription Factor IRF4 Promotes CD8+ T Cell Exhaustion and Limits the Development of Memory‐Like T Cells During Chronic Infection,” Immunity 47, no. 6 (2017): 1129–1141, 10.1016/j.immuni.2017.11.021. 29246443

[328] L. Tille , D. Cropp , M. Charmoy , et al., “Activation of the Transcription Factor NFAT5 in the Tumor Microenvironment Enforces CD8+ T Cell Exhaustion,” Nature Immunology 24, no. 10 (2023): 1645–1653, 10.1038/s41590-023-01614-x. 37709986

[329] L. E. Worton , R. Y. Kwon , E. M. Gardiner , T. S. Gross , and S. Srinivasan , “Enhancement of Flow‐Induced AP‐1 Gene Expression by Cyclosporin A Requires NFAT‐Independent Signaling in Bone Cells,” Cellular and Molecular Bioengineering 7, no. 2 (2014): 254–265, 10.1007/s12195-014-0321-3. 25484988 PMC4255985

[330] J. Chen , I. F. Lopez‐Moyado , H. Seo , et al., “NR4A Transcription Factors Limit CAR T Cell Function in Solid Tumours,” Nature 567, no. 7749 (2019): 530–534, 10.1038/s41586-019-0985-x. 30814732 PMC6546093

[331] J. Hao , R. Li , X. Zhao , et al., “NR4A1 Transcriptionally Regulates the Differentiation of Stem‐Like CD8+ T Cells in the Tumor Microenvironment,” Cell Reports 43, no. 6 (2024): 114301, 10.1016/j.celrep.2024.114301. 38823016

[332] D. Bending and J. Zikherman , “Nr4a Nuclear Receptors: Markers and Modulators of Antigen Receptor Signaling,” Current Opinion in Immunology 81 (2023): 102285, 10.1016/j.coi.2023.102285. 36764055

[333] H. Shin , S. D. Blackburn , A. M. Intlekofer , et al., “A Role for the Transcriptional Repressor Blimp‐1 in CD8+ T Cell Exhaustion During Chronic Viral Infection,” Immunity 31, no. 2 (2009): 309–320, 10.1016/j.immuni.2009.06.019. 19664943 PMC2747257

[334] S. H. Fu , L. T. Yeh , C. C. Chu , B. L. Yen , and H. K. Sytwu , “New Insights Into Blimp‐1 in T Lymphocytes: A Divergent Regulator of Cell Destiny and Effector Function,” Journal of Biomedical Science 24, no. 1 (2017): 49, 10.1186/s12929-017-0354-8. 28732506 PMC5520377

[335] K. Kim , S. Park , S. Y. Park , et al., “Single‐Cell Transcriptome Analysis Reveals TOX as a Promoting Factor for T Cell Exhaustion and a Predictor for Anti‐PD‐1 Responses in Human Cancer,” Genome Medicine 12, no. 1 (2020): 22, 10.1186/s13073-020-00722-9. 32111241 PMC7048139

[336] S. M. Reading , I. Munoz , M. N. de Menezes , et al., “Abstract LT02: Regulation of Exhausted CD8+ T Cell Differentiation by IKZF Transcription Factors,” Cancer Research 84, no. 8 (2024): LT02–LT02, 10.1158/1538-7445.FCS2023-LT02.

[337] M. S. Dahabieh , L. M. DeCamp , B. M. Oswald , et al., “The Prostacyclin Receptor PTGIR Is a NRF2‐Dependent Regulator of CD8+ T Cell Exhaustion,” Nature Immunology 26, no. 7 (2025): 1139–1151, 10.1038/s41590-025-02185-9. 40579556 PMC12208871

[338] Y. Jo , J. A. Shim , J. W. Jeong , et al., “Targeting ROS‐Sensing Nrf2 Potentiates Anti‐Tumor Immunity of Intratumoral CD8+ T and CAR‐T Cells,” Molecular Therapy 32, no. 11 (2024): 3879–3894, 10.1016/j.ymthe.2024.08.019. 39169624 PMC11573615

[339] S. Tiwari‐Heckler , G. R. Lee , J. Harbison , et al., “Extracellular Mitochondria Drive CD8 T Cell Dysfunction in Trauma by Upregulating CD39,” Thorax 78, no. 2 (2023): 151, 10.1136/thoraxjnl-2021-218047. 35613855 PMC9691787

[340] P. K. Gupta , J. Godec , D. Wolski , et al., “CD39 Expression Identifies Terminally Exhausted CD8+ T Cells,” PLOS Pathogens 11, no. 10 (2015): 1005177, 10.1371/journal.ppat.1005177. PMC4618999 26485519

[341] W. Ding , J. Mo , Y. Su , et al., “Metabolic Reprogramming of Tumor‐Associated Macrophages via Adenosine‐A2AR Signaling Drives Cross‐Resistance in Non‐Small Cell Lung Cancer,” Drug Resistance Updates 82 (2025): 101272, 10.1016/j.drup.2025.101272. 40618433

[342] C. Xia , S. Yin , K. K. W. To , and L. Fu , “CD39/CD73/A2AR Pathway and Cancer Immunotherapy,” Molecular Cancer 22, no. 1 (2023): 44, 10.1186/s12943-023-01733-x. 36859386 PMC9979453

[343] B. Mastelic‐Gavillet , B. Navarro Rodrigo , L. Decombaz , et al., “Adenosine Mediates Functional and Metabolic Suppression of Peripheral and Tumor‐Infiltrating CD8+ T Cells,” Journal for ImmunoTherapy of Cancer 7, no. 1 (2019): 257, 10.1186/s40425-019-0719-5. 31601268 PMC6788118

[344] I. Shevchenko , A. Mathes , C. Groth , et al., “Enhanced Expression of CD39 and CD73 on T Cells in the Regulation of Anti‐Tumor Immune Responses,” Oncoimmunology 9, no. 1 (2020): 1744946. 33457090 10.1080/2162402X.2020.1744946 PMC7790505

[345] C. Kolbe , J. Kauer , B. Brinkmann , et al., “Blocking the CD39/CD73 Pathway Synergizes with Anti‐CD20 Bispecific Antibody in Nodal B‐Cell Lymphoma,” Journal for ImmunoTherapy of Cancer 13, no. 1 (2025): 009245, 10.1136/jitc-2024-009245. PMC11784132 39884778

[346] P. D. A. Vignali , K. DePeaux , M. J. Watson , et al., “Hypoxia Drives CD39‐Dependent Suppressor Function in Exhausted T Cells to Limit Antitumor Immunity,” Nature Immunology 24, no. 2 (2023): 267–279, 10.1038/s41590-022-01379-9. 36543958 PMC10402660

[347] M. Witt , L. Oliveira‐Ferrer , F. Koch‐Nolte , et al., “Expression of CD39 Is Associated with T Cell Exhaustion in Ovarian Cancer and Its Blockade Reverts T Cell Dysfunction,” Oncoimmunology 13 (2024): 2346359. 38737794 10.1080/2162402X.2024.2346359 PMC11087076

[348] I. Perrot , H. A. Michaud , M. Giraudon‐Paoli , et al., “Blocking Antibodies Targeting the CD39/CD73 Immunosuppressive Pathway Unleash Immune Responses in Combination Cancer Therapies,” Cell Reports 27, no. 8 (2019): 2411–2425, 10.1016/j.celrep.2019.04.091. 31116985

[349] I. Campia , I. Buondonno , B. Castella , et al., “An Autocrine Cytokine/JAK/STAT‐Signaling Induces Kynurenine Synthesis in Multidrug Resistant Human Cancer Cells,” PLoS ONE 10, no. 5 (2015): 0126159, 10.1371/journal.pone.0126159. PMC4425697 25955018

[350] M. S. Paul , S. D. Saibil , M. Kates , et al., “Ex Vivo Activation of the GCN2 Pathway Metabolically Reprograms T Cells, Leading to Enhanced Adoptive Cell Therapy,” Cell Reports Medicine 5, no. 3 (2024): 101465, 10.1016/j.xcrm.2024.101465. 38460518 PMC10983112

[351] X. Liu , M. Yang , P. Xu , et al., “Kynurenine‐AhR Reduces T‐Cell Infiltration and Induces a Delayed T‐Cell Immune Response by Suppressing the STAT1‐CXCL9/CXCL10 Axis in Tuberculosis,” Cellular & Molecular Immunology 21, no. 12 (2024): 1426–1440, 10.1038/s41423-024-01230-1. 39438693 PMC11607402

[352] L. F. Campesato , S. Budhu , J. Tchaicha , et al., “Blockade of the AHR Restricts a Treg‐Macrophage Suppressive Axis Induced by L‐Kynurenine,” Nature Communications 11, no. 1 (2020): 4011, 10.1038/s41467-020-17750-z. PMC7419300 32782249

[353] Y. Liu , N. Zhou , L. Zhou , et al., “IL‐2 Regulates Tumor‐Reactive CD8+ T Cell Exhaustion by Activating the Aryl Hydrocarbon Receptor,” Nature Immunology 22, no. 3 (2021): 358–369, 10.1038/s41590-020-00850-9. 33432230

[354] S. Liu , Y. Cao , K. Cui , et al., “Regulation of T Helper Cell Differentiation by the Interplay Between Histone Modification and Chromatin Interaction,” Immunity 57, no. 5 (2024): 987–1004, 10.1016/j.immuni.2024.03.018. 38614090 PMC11096031

[355] Y. Zhou , L. Yao , T. Ma , et al., “Indoleamine 2,3‐Dioxygenase‐1 Involves in CD8+T Cell Exhaustion in Glioblastoma via Regulating Tryptophan Levels,” International Immunopharmacology 142, no. Pt A (2024): 113062, 10.1016/j.intimp.2024.113062. 39244898

[356] T. C. Mitchell , O. Hamid , D. C. Smith , et al., “Epacadostat Plus Pembrolizumab in Patients With Advanced Solid Tumors: Phase I Results From a Multicenter, Open‐Label Phase I/II Trial (ECHO‐202/KEYNOTE‐037),” Journal of Clinical Oncology 36, no. 32 (2018): 3223–3230, 10.1200/JCO.2018.78.9602. 30265610 PMC6225502

[357] L. Cen , Y. Wu , M. He , et al., “Discovery and Optimization of Novel Apo‐IDO1 Inhibitors by a Pharmacophore‐Based Structural Simplification Strategy,” Journal of Medicinal Chemistry 68, no. 6 (2025): 6633–6655, 10.1021/acs.jmedchem.5c00034. 40042617

[358] L. Xie , K. Hu , Y. Duo , et al., “Off‐tumor IDO1 Target Engagements Determine the Cancer‐Immune Set Point and Predict the Immunotherapeutic Efficacy,” Journal for ImmunoTherapy of Cancer 9, no. 6 (2021): 002616. 10.1136/jitc-2021-002616 PMC8237741 34148865

[359] U. F. Rohrig , A. Reynaud , S. R. Majjigapu , P. Vogel , F. Pojer , and V. Zoete , “Inhibition Mechanisms of Indoleamine 2,3‐Dioxygenase 1 (IDO1),” Journal of Medicinal Chemistry 62, no. 19 (2019): 8784–8795, 10.1021/acs.jmedchem.9b00942. 31525930

[360] M. Platten , E. A. A. Nollen , U. F. Rohrig , F. Fallarino , and C. A. Opitz , “Tryptophan Metabolism as a Common Therapeutic Target in Cancer, Neurodegeneration and Beyond,” Nature Reviews Drug Discovery 18, no. 5 (2019): 379–401, 10.1038/s41573-019-0016-5. 30760888

[361] X. Ma , E. Bi , Y. Lu , et al., “Cholesterol Induces CD8+ T Cell Exhaustion in the Tumor Microenvironment,” Cell Metabolism 30, no. 1 (2019): 143–156, 10.1016/j.cmet.2019.04.002. 31031094 PMC7061417

[362] F. Li , Y. Feng , Z. Yin , and Y. Wang , “Mitochondrial Metabolism in T‐Cell Exhaustion,” International Journal of Molecular Sciences 26, no. 15 (2025): 7400. 40806529 10.3390/ijms26157400 PMC12347488

[363] Y. Lv , M. Li , L. Weng , et al., “Ginseng‐Derived Nanoparticles Reprogram Macrophages to Regulate Arginase‐1 Release for Ameliorating T Cell Exhaustion in Tumor Microenvironment,” Journal of Experimental & Clinical Cancer Research 42, no. 1 (2023): 322, 10.1186/s13046-023-02888-7. 38012650 PMC10683135

[364] R. Jin , J. Hao , J. Yu , P. Wang , E. R. Sauter , and B. Li , “Role of FABP5 in T Cell Lipid Metabolism and Function in the Tumor Microenvironment,” Cancers (Basel) 15, no. 3 (2023): 657. 36765614 10.3390/cancers15030657 PMC9913835

[365] S. Zhu , T. Zhang , L. Zheng , et al., “Combination Strategies to Maximize the Benefits of Cancer Immunotherapy,” Journal of Hematology & Oncology 14, no. 1 (2021): 156, 10.1186/s13045-021-01164-5. 34579759 PMC8475356

[366] Y. Lu , S. Ma , Y. T. Chan , Y. Wu , Y. Feng , and N. Wang , “Epigenetic Orchestration of Cancer‐Immune Dynamics: Mechanisms, Technologies, and Clinical Advancements,” *Journal of Advanced Research* (Early View), 10.1016/j.jare.2025.08.057. PMC13131514 40939889

[367] H. Wang , X. Niu , Z. Jin , et al., “Immunotherapy Resistance in Non‐Small Cell Lung Cancer: from Mechanisms to Therapeutic Opportunities,” Journal of Experimental & Clinical Cancer Research 44, no. 1 (2025): 250, 10.1186/s13046-025-03519-z. 40849659 PMC12374485

[368] B. Wang , M. Tang , Q. Chen , et al., “Delivery of mRNA Encoding Interleukin‐12 and a Stimulator of Interferon Genes Agonist Potentiates Antitumor Efficacy through Reversing T Cell Exhaustion,” ACS Nano 18, no. 24 (2024): 15499, 10.1021/acsnano.4c00063. 38832815

[369] X. Ma , Q. Wang , C. Sun , et al., “Targeting TCF19 Sensitizes MSI Endometrial Cancer to Anti‐PD‐1 Therapy by Alleviating CD8+ T Cell Exhaustion via TRIM14‐IFN‐β Axis,” Cell Reports 42, no. 8 (2023): 112944, 10.1016/j.celrep.2023.112944. 37566545

[370] D. M. Pardoll , “The Blockade of Immune Checkpoints in Cancer Immunotherapy,” Nature Reviews Cancer 12, no. 4 (2012): 252, 10.1038/nrc3239. 22437870 PMC4856023

[371] W. Zou , J. D. Wolchok , and L. Chen , “PD‐L1 (B7‐H1) and PD‐1 Pathway Blockade for Cancer Therapy: Mechanisms, Response Biomarkers, and Combinations,” Science Translational Medicine 8, no. 328 (2016): 328rv4, 10.1126/scitranslmed.aad7118. PMC4859220 26936508

[372] R. Offringa , L. Kotzner , B. Huck , and K. Urbahns , “The Expanding Role for Small Molecules in Immuno‐Oncology,” Nature Reviews Drug Discovery 21, no. 11 (2022): 821–840, 10.1038/s41573-022-00538-9. 35982333

[373] W. Chen , J. M. N. Teo , S. W. Yau , et al., “Chronic Type I Interferon Signaling Promotes Lipid‐Peroxidation‐Driven Terminal CD8+ T Cell Exhaustion and Curtails Anti‐PD‐1 Efficacy,” Cell Reports 41, no. 7 (2022): 111647, 10.1016/j.celrep.2022.111647. 36384131

[374] R. Leshem , K. N. Sefton , C. W. Wong , et al., “Combined PARP14 Inhibition and PD‐1 Blockade Promotes Cytotoxic T Cell Quiescence and Modulates Macrophage Polarization in Relapsed Melanoma,” Journal for ImmunoTherapy of Cancer 13, no. 1 (2025): 010683, 10.1136/jitc-2024-010683. PMC11772928 39870492

[375] M. Hashimoto , S. S. Ramalingam , and R. Ahmed , “Harnessing CD8 T Cell Responses Using PD‐1–IL‐2 Combination Therapy,” Trends in Cancer 10, no. 4 (2024): 332–346, 10.1016/j.trecan.2023.11.008. 38129234 PMC11006586

[376] J. Bae , L. Liu , C. Moore , et al., “IL‐2 Delivery by Engineered Mesenchymal Stem Cells Re‐Invigorates CD8+ T Cells to Overcome Immunotherapy Resistance in Cancer,” Nature Cell Biology 24, no. 12 (2022): 1754–1765, 10.1038/s41556-022-01024-5. 36474070

[377] L. Zhang , X. Guo , X. Sun , et al., “Analysis of Tumor‐infiltrating Exhausted T Cells Highlights IL‐6 and PD1 Blockade as a Combined Immunotherapy Strategy for Non‐Small Cell Lung Cancer,” Frontiers in Immunology 16 (2025): 1486329, 10.3389/fimmu.2025.1486329. 40040705 PMC11876966

[378] H. Yin , N. Pu , Q. Chen , et al., “Gut‐Derived Lipopolysaccharide Remodels Tumoral Microenvironment and Synergizes with PD‐L1 Checkpoint Blockade via TLR4/MyD88/AKT/NF‐κB Pathway in Pancreatic Cancer,” Cell Death & Disease 12, no. 11 (2021): 1033, 10.1038/s41419-021-04293-4. 34718325 PMC8557215

[379] K. M. Viramontes , E. N. Neubert , J. M. DeRogatis , and R. Tinoco , “PD‐1 Immune Checkpoint Blockade and PSGL‐1 Inhibition Synergize to Reinvigorate Exhausted T Cells,” Frontiers in Immunology 13 (2022): 869768, 10.3389/fimmu.2022.869768. 35774790 PMC9237324

[380] K. Mortezaee , “B7‐H3 Immunoregulatory Roles in Cancer,” Biomedicine & Pharmacotherapy 163 (2023): 114890, 10.1016/j.biopha.2023.114890. 37196544

[381] I. Salewski , J. Henne , L. Engster , et al., “CDK4/6 Blockade Provides an Alternative Approach for Treatment of Mismatch‐Repair Deficient Tumors,” Oncoimmunology 11, no. 1 (2022): 2094583. 35845723 10.1080/2162402X.2022.2094583 PMC9278458

[382] J. G. Matous , J. P. Snook , N. A. Contreras , et al., “Shp‐1 Regulates the Activity of Low‐Affinity T Cells Specific to Endogenous Self‐Antigen During Melanoma Tumor Growth and Drives Resistance to Immune Checkpoint Inhibition,” Journal for ImmunoTherapy of Cancer 13, no. 4 (2025): 010879, 10.1136/jitc-2024-010879. PMC12007028 40246583

[383] H. Sheng , Y. Huang , Y. Xiao , et al., “ATR Inhibitor AZD6738 Enhances the Antitumor Activity of Radiotherapy and Immune Checkpoint Inhibitors by Potentiating the Tumor Immune Microenvironment in Hepatocellular Carcinoma,” Journal for ImmunoTherapy of Cancer 8, no. 1 (2020): 000340, 10.1136/jitc-2019-000340. PMC7254123 32461345

[384] B. Cheng , J. Wu , K. Chen , et al., “Association of 5α‐Reductase Inhibitor Prescription with Immunotherapy Efficacy in Metastatic Renal Cell Carcinoma: A Multicenter Retrospective Analysis,” Journal for ImmunoTherapy of Cancer 13, no. 2 (2025): 011154, 10.1136/jitc-2024-011154. PMC11865730 40010773

[385] Y. Naito , S. Koyama , K. Masuhiro , et al., “Tumor‐Derived Semaphorin 4A Improves PD‐1–Blocking Antibody Efficacy by Enhancing CD8 + T Cell Cytotoxicity and Proliferation,” Science Advances 9, no. 20 (2023): ade0718, 10.1126/sciadv.ade0718. PMC10198637 37205755

[386] M. Hashimoto , J. D. Konda , S. Perrino , M. C. Fernandez , A. M. Lowy , and P. Brodt , “Targeting the IGF‐Axis Potentiates Immunotherapy for Pancreatic Ductal Adenocarcinoma Liver Metastases by Altering the Immunosuppressive Microenvironment,” Molecular Cancer Therapeutics 20, no. 12 (2021): 2469–2482, 10.1158/1535-7163.MCT-20-0144. 34552012 PMC8677570

[387] P. Icard , M. Prieto , A. Coquerel , et al., “Why and How Citrate May Sensitize Malignant Tumors to Immunotherapy,” Drug Resistance Updates 78 (2025): 101177, 10.1016/j.drup.2024.101177. 39612545

[388] A. M. Amitrano , B. J. Berry , K. Lim , et al., “Optical Control of CD8+ T Cell Metabolism and Effector Functions,” Frontiers in Immunology 12 (2021): 666231, 10.3389/fimmu.2021.666231. 34149701 PMC8209468

[389] Y. Liu , B. Debo , M. Li , Z. Shi , W. Sheng , and Y. Shi , “LSD1 Inhibition Sustains T Cell Invigoration with a Durable Response to PD‐1 Blockade,” Nature Communications 12, no. 1 (2021): 6831, 10.1038/s41467-021-27179-7. PMC8613218 34819502

[390] W. Sheng , Y. Liu , D. Chakraborty , B. Debo , and Y. Shi , “Simultaneous Inhibition of LSD1 and TGFβ Enables Eradication of Poorly Immunogenic Tumors with Anti–PD‐1 Treatment,” Cancer Discovery 11, no. 8 (2021): 1970–1981, 10.1158/2159-8290.CD-20-0017. 33687985 PMC8598400

[391] S. Karan , E. Jung , C. Boone , and N. F. Steinmetz , “Synergistic Combination Therapy Using Cowpea Mosaic Virus Intratumoral Immunotherapy and Lag‐3 Checkpoint Blockade,” Cancer Immunology, Immunotherapy 73, no. 3 (2024): 51, 10.1007/s00262-024-03636-2. 38349406 PMC10864561

[392] F. Ju , Y. Luo , C. Lin , et al., “Oncolytic Virus Expressing PD‐1 Inhibitors Activates a Collaborative Intratumoral Immune Response to Control Tumor and Synergizes with CTLA‐4 or TIM‐3 Blockade,” Journal for ImmunoTherapy of Cancer 10, no. 6 (2022): 004762. 10.1136/jitc-2022-004762 PMC9189843 35688558

[393] T. Tian , R. Liang , G. Erel‐Akbaba , et al., “Immune Checkpoint Inhibition in GBM Primed with Radiation by Engineered Extracellular Vesicles,” ACS Nano 16, no. 2 (2022): 1940–1953, 10.1021/acsnano.1c05505. 35099172 PMC9020451

[394] U. Horzum , H. Yanik , E. Z. Taskiran , and G. Esendagli , “Effector Th1 Cells under PD ‐1 and CTLA ‐4 Checkpoint Blockade Abrogate the Upregulation of Multiple Inhibitory Receptors and By‐Pass Exhaustion,” Immunology 167, no. 4 (2022): 640–650, 10.1111/imm.13560. 36053975

[395] W. Y. Du , H. Masuda , K. Nagaoka , et al., “Janus Kinase Inhibitor Overcomes Resistance to Immune Checkpoint Inhibitor Treatment in Peritoneal Dissemination of Gastric Cancer in C57BL/6 J Mice,” Gastric Cancer 27, no. 5 (2024): 971–985, 10.1007/s10120-024-01514-5. 38805119 PMC11335826

[396] K. Wang , P. Coutifaris , D. Brocks , et al., “Combination Anti‐PD‐1 and Anti‐CTLA‐4 Therapy Generates Waves of Clonal Responses That Include Progenitor‐Exhausted CD8+ T Cells,” Cancer Cell 42, no. 9 (2024): 1582–1597, 10.1016/j.ccell.2024.08.007. 39214097 PMC11387127

[397] H. Li , D. P. Zandberg , A. Kulkarni , et al., “Distinct CD8+ T Cell Dynamics Associate with Response to Neoadjuvant Cancer Immunotherapies,” Cancer Cell 43, no. 4 (2025): 757–775, 10.1016/j.ccell.2025.02.026. 40086437 PMC12359303

[398] M. McLaughlin , E. C. Patin , M. Pedersen , et al., “Inflammatory Microenvironment Remodelling by Tumour Cells after Radiotherapy,” Nature Reviews Cancer 20, no. 4 (2020): 203–217, 10.1038/s41568-020-0246-1. 32161398

[399] L. F. Spurr , C. A. Martinez , W. Kang , et al., “Highly Aneuploid Non‐Small Cell Lung Cancer Shows Enhanced Responsiveness to Concurrent Radiation and Immune Checkpoint Blockade,” Nature Cancer 3, no. 12 (2022): 1498–1512, 10.1038/s43018-022-00467-x. 36443406

[400] S. C. Formenti , N. P. Rudqvist , E. Golden , et al., “Radiotherapy Induces Responses of Lung Cancer to CTLA‐4 Blockade,” Nature Medicine 24, no. 12 (2018): 1845–1851, 10.1038/s41591-018-0232-2. PMC6286242 30397353

[401] P. J. Saylor , S. V. Kozin , A. Matsui , et al., “The Radiopharmaceutical Radium‐223 Has Immunomodulatory Effects in Patients and Facilitates Anti‐Programmed Death Receptor‐1 Therapy in Murine Models of Bone Metastatic Prostate Cancer,” Radiotherapy and Oncology 192 (2024): 110091, 10.1016/j.radonc.2024.110091. 38224917 PMC10905770

[402] B. Palakurthi , S. R. Fross , I. H. Guldner , et al., “Targeting CXCL16 and STAT1 Augments Immune Checkpoint Blockade Therapy in Triple‐Negative Breast Cancer,” Nature Communications 14, no. 1 (2023): 2109, 10.1038/s41467-023-37727-y. PMC10101955 37055410

[403] Y. Guan , S. G. Kraus , M. J. Quaney , M. A. Daniels , J. B. Mitchem , and E. Teixeiro , “FOLFOX Chemotherapy Ameliorates CD8 T Lymphocyte Exhaustion and Enhances Checkpoint Blockade Efficacy in Colorectal Cancer,” Frontiers in Oncology 10 (2020): 586, 10.3389/fonc.2020.00586. 32391270 PMC7190812

[404] Y. Song , L. Bugada , R. Li , et al., “Albumin Nanoparticle Containing a PI3Kγ Inhibitor and Paclitaxel in Combination with α‐PD1 Induces Tumor Remission of Breast Cancer in Mice,” Science Translational Medicine 14, no. 643 (2022): abl3649, 10.1126/scitranslmed.abl3649. PMC9589917 35507675

[405] X. Bai , Y. Zhou , Y. Yokota , et al., “Adaptive Antitumor Immune Response Stimulated by Bio‐Nanoparticle Based Vaccine and Checkpoint Blockade,” Journal of Experimental and Clinical Cancer Research 41, no. 1 (2022): 132. 35392977 10.1186/s13046-022-02307-3 PMC8991500

[406] D. N. Khalil , N. Suek , L. F. Campesato , et al., “In Situ Vaccination With Defined Factors Overcomes T Cell Exhaustion in Distant Tumors,” Journal of Clinical Investigation 129, no. 8 (2019): 3435, 10.1172/JCI128562. 31329159 PMC6668692

[407] J. Hsu , R. N. Donahue , M. Katragadda , et al., “A T Cell Receptor β Chain–Directed Antibody Fusion Molecule Activates and Expands Subsets of T Cells to Promote Antitumor Activity,” Science Translational Medicine 15, no. 724 (2023): adi0258, 10.1126/scitranslmed.adi0258. PMC11421222 38019931

[408] C. Raffin , L. T. Vo , and J. A. Bluestone , “Treg Cell‐Based Therapies: Challenges and Perspectives,” Nature Reviews Immunology 20, no. 3 (2020): 158–172, 10.1038/s41577-019-0232-6. PMC7814338 31811270

[409] L. Gattinoni , C. A. Klebanoff , and N. P. Restifo , “Paths to Stemness: Building the Ultimate Antitumour T Cell,” Nature Reviews Cancer 12, no. 10 (2012): 671–684, 10.1038/nrc3322. 22996603 PMC6352980

[410] N. Jain , Z. Zhao , J. Feucht , et al., “TET2 guards against Unchecked BATF3‐induced CAR T Cell Expansion,” Nature 615, no. 7951 (2023): 315–322, 10.1038/s41586-022-05692-z. 36755094 PMC10511001

[411] R. C. Lynn , E. W. Weber , E. Sotillo , et al., “c‐Jun Overexpression in CAR T Cells Induces Exhaustion Resistance,” Nature 576, no. 7786 (2019): 293–300, 10.1038/s41586-019-1805-z. 31802004 PMC6944329

[412] Y. Zhao , J. Chen , M. Andreatta , et al., “IL‐10‐Expressing CAR T Cells Resist Dysfunction and Mediate Durable Clearance of Solid Tumors and Metastases,” Nature Biotechnology 42, no. 11 (2024): 1693–1704, 10.1038/s41587-023-02060-8. 38168996

[413] J. E. Jaspers , J. F. Khan , W. D. Godfrey , et al., “IL‐18‐secreting CAR T Cells Targeting DLL3 Are Highly Effective in Small Cell Lung Cancer Models,” Journal of Clinical Investigation 133, no. 9 (2023): 166028. 10.1172/JCI166028 PMC10145930 36951942

[414] H. Feng , L. Qiu , Z. Shi , et al., “Modulation of Intracellular Kinase Signaling to Improve TIL Stemness and Function for Adoptive Cell Therapy,” Cancer Medicine 12, no. 3 (2023): 3313–3327, 10.1002/cam4.5095. 36028997 PMC9939193

[415] Y. Wang , H. Zhang , G. Du , et al., “Enforced Expression of Runx3 Improved CAR‐T Cell Potency in Solid Tumor via Enhancing Resistance to Activation‐induced Cell Death,” Molecular Therapy 31, no. 3 (2023): 701–714, 10.1016/j.ymthe.2022.12.009. 36523165 PMC10014350

[416] Q. Fu , Y. Zheng , W. Fang , et al., “RUNX‐3‐expressing CAR T Cells Targeting Glypican‐3 in Patients with Heavily Pretreated Advanced Hepatocellular Carcinoma: A Phase I Trial,” EClinicalMedicine 63 (2023): 102175, 10.1016/j.eclinm.2023.102175. 37680942 PMC10480529

[417] C. He , Y. Zhou , Z. Li , et al., “Co‐Expression of IL‐7 Improves NKG2D‐Based CAR T Cell Therapy on Prostate Cancer by Enhancing the Expansion and Inhibiting the Apoptosis and Exhaustion,” Cancers (Basel) 12, no. 7 (2020): 1969. 32698361 10.3390/cancers12071969 PMC7409228

[418] S. R. McCutcheon , A. M. Swartz , M. C. Brown , et al., “Transcriptional and Epigenetic Regulators of Human CD8+ T Cell Function Identified through Orthogonal CRISPR Screens,” Nature Genetics 55, no. 12 (2023): 2211, 10.1038/s41588-023-01554-0. 37945901 PMC10703699

[419] X. Zhang , C. Zhang , M. Qiao , et al., “Depletion of BATF in CAR‐T Cells Enhances Antitumor Activity by Inducing Resistance Against Exhaustion and Formation of central Memory Cells,” Cancer Cell 40, no. 11 (2022): 1407–1422, 10.1016/j.ccell.2022.09.013. 36240777

[420] Y. Zhang , Q. Zhuang , F. Wang , et al., “Co‐expression IL‐15 Receptor Alpha with IL‐15 Reduces Toxicity via Limiting IL‐15 Systemic Exposure during CAR‐T Immunotherapy,” Journal of Translational Medicine 20, no. 1 (2022): 432, 10.1186/s12967-022-03626-x. 36167591 PMC9516829

[421] C. R. Good , M. A. Aznar , S. Kuramitsu , et al., “An NK‐like CAR T Cell Transition in CAR T Cell Dysfunction,” Cell 184, no. 25 (2021): 6081–6100. 34861191 10.1016/j.cell.2021.11.016 PMC8827167

[422] I. Y. Jung , V. Narayan , S. McDonald , et al., “BLIMP1 and NR4A3 Transcription Factors Reciprocally Regulate Antitumor CAR T Cell Stemness and Exhaustion,” Science Translational Medicine 14, no. 670 (2022): abn7336, 10.1126/scitranslmed.abn7336. PMC10257143 36350986

[423] S. Liang , R. Zheng , B. Zuo , et al., “SMAD7 Expression in CAR‐T Cells Improves Persistence and Safety for Solid Tumors,” Cellular & Molecular Immunology 21, no. 3 (2024): 213–226, 10.1038/s41423-023-01120-y. 38177245 PMC10901810

[424] J. Si , X. Shi , S. Sun , et al., “Hematopoietic Progenitor Kinase1 (HPK1) Mediates T Cell Dysfunction and Is a Druggable Target for T Cell‐Based Immunotherapies,” Cancer Cell 38, no. 4 (2020): 551–566, 10.1016/j.ccell.2020.08.001. 32860752

[425] W. Ouyang , S. W. Jin , N. Xu , et al., “PD‐1 Downregulation Enhances CAR‐T Cell Antitumor Efficiency by Preserving a Cell Memory Phenotype and Reducing Exhaustion,” Journal for ImmunoTherapy of Cancer 12, no. 4 (2024): 008429, 10.1136/jitc-2023-008429. PMC11015237 38589248

[426] L. Cherkassky , A. Morello , J. Villena‐Vargas , et al., “Human CAR T Cells with Cell‐Intrinsic PD‐1 Checkpoint Blockade Resist Tumor‐Mediated Inhibition,” Journal of Clinical Investigation 126, no. 8 (2016): 3130, 10.1172/JCI83092. 27454297 PMC4966328

[427] F. Zou , L. Lu , J. Liu , et al., “Engineered Triple Inhibitory Receptor Resistance Improves Anti‐Tumor CAR‐T Cell Performance via CD56,” Nature Communications 10, no. 1 (2019): 4109, 10.1038/s41467-019-11893-4. PMC6739330 31511513

[428] T. Yoshikawa , Z. Wu , S. Inoue , et al., “Genetic Ablation of PRDM1 in Antitumor T Cells Enhances Therapeutic Efficacy of Adoptive Immunotherapy,” Blood 139, no. 14 (2022): 2156, 10.1182/blood.2021012714. 34861037

[429] R. Nguyen , E. Doubrovina , C. M. Mousset , et al., “Cooperative Armoring of CAR and TCR T Cells by T Cell–Restricted IL15 and IL21 Universally Enhances Solid Tumor Efficacy,” Clinical Cancer Research 30, no. 8 (2024): 1555, 10.1158/1078-0432.CCR-23-1872. 37910044 PMC11018485

[430] M. S. Hussein , Q. Li , R. Mao , Y. Peng , and Y. He , “TCR T Cells Overexpressing c‐Jun Have Better Functionality with Improved Tumor Infiltration and Persistence in Hepatocellular Carcinoma,” Frontiers in Immunology 14 (2023): 1114770, 10.3389/fimmu.2023.1114770. 37215108 PMC10192869

[431] G. Kadyrzhanova , M. Tamai , S. Sarkar , R. S. Kalra , and H. Ishikawa , “Aging Impairs CD8 T Cell Responses in Adoptive T‐cell Therapy against Solid Tumors,” Frontiers in Immunology 16 (2025): 1484303, 10.3389/fimmu.2025.1484303. 39925817 PMC11803149

[432] N. Sailer , I. Fetzer , M. Salvermoser , et al., “T‐Cells Expressing a Highly Potent PRAME‐Specific T‐Cell Receptor in Combination with a Chimeric PD1‐41BB Co‐Stimulatory Receptor Show a Favorable Preclinical Safety Profile and Strong Anti‐Tumor Reactivity,” Cancers 14, no. 8 (2022): 1998. 35454906 10.3390/cancers14081998 PMC9030144

[433] B. C. Cianciotti , Z. I. Magnani , A. Ugolini , et al., “TIM‐3, LAG‐3, or 2B4 Gene Disruptions Increase the Anti‐Tumor Response of Engineered T Cells,” Frontiers in Immunology 15 (2024): 1315283, 10.3389/fimmu.2024.1315283. 38510235 PMC10953820

[434] D. C. Palmer , B. R. Webber , Y. Patel , et al., “Internal Checkpoint Regulates T Cell Neoantigen Reactivity and Susceptibility to PD1 Blockade,” Med 3, no. 10 (2022): 682. 36007524 10.1016/j.medj.2022.07.008 PMC9847506

[435] A. Jimenez‐Reinoso , M. Molero‐Abraham , C. Cirauqui , et al., “CD4 + Tumor‐Infiltrating Lymphocytes Secreting T Cell‐Engagers Induce Regression of Autologous Patient‐derived Non‐small Cell Lung Cancer Xenografts,” Oncoimmunology 13 (2024): 2392897, 10.1080/2162402X.2024.2392897. 39206095 PMC11352715

[436] M. Kalaitsidou , O. R. Moon , M. Sykorova , et al., “Signaling via a CD28/CD40 Chimeric Costimulatory Antigen Receptor (CoStAR™), Targeting Folate Receptor Alpha, Enhances T Cell Activity and Augments Tumor Reactivity of Tumor Infiltrating Lymphocytes,” Front Immunol 14 (2023): 1256491. 38022678 10.3389/fimmu.2023.1256491 PMC10664248

[437] C. H. June , R. S. O'Connor , O. U. Kawalekar , S. Ghassemi , and M. C. Milone , “CAR T Cell Immunotherapy for Human Cancer,” Science 359, no. 6382 (2018): 1361–1365, 10.1126/science.aar6711. 29567707

[438] E. W. Weber , M. V. Maus , and C. L. Mackall , “The Emerging Landscape of Immune Cell Therapies,” Cell 181, no. 1 (2020): 46–62, 10.1016/j.cell.2020.03.001. 32243795 PMC8900215

[439] M. Poorebrahim , J. Melief , Y. Pico de Coana , S. L. Wickström , A. Cid‐Arregui , and R. Kiessling , “Counteracting CAR T Cell Dysfunction,” Oncogene 40, no. 2 (2021): 421–435, 10.1038/s41388-020-01501-x. 33168929 PMC7808935

[440] Q. Deng , G. Han , N. Puebla‐Osorio , et al., “Characteristics of Anti‐CD19 CAR T Cell Infusion Products Associated with Efficacy and Toxicity in Patients With Large B Cell Lymphomas,” Nature Medicine 26, no. 12 (2020): 1878–1887, 10.1038/s41591-020-1061-7. PMC8446909 33020644

[441] N. J. Haradhvala , M. B. Leick , K. Maurer , et al., “Distinct Cellular Dynamics Associated with Response to CAR‐T Therapy for Refractory B Cell Lymphoma,” Nature Medicine 28, no. 9 (2022): 1848–1859, 10.1038/s41591-022-01959-0. PMC9509487 36097221

[442] S. Arcangeli , C. Bove , C. Mezzanotte , et al., “CAR T Cell Manufacturing from Naive/Stem Memory T Lymphocytes Enhances Antitumor Responses While Curtailing Cytokine Release Syndrome,” Journal of Clinical Investigation 132, no. 12 (2022): 150807. 10.1172/JCI150807 PMC9197529 35503659

[443] Y. Chen , C. Sun , E. Landoni , L. Metelitsa , G. Dotti , and B. Savoldo , “Eradication of Neuroblastoma by T Cells Redirected With an Optimized GD2‐Specific Chimeric Antigen Receptor and Interleukin‐15,” Clinical Cancer Research 25, no. 9 (2019): 2915–2924, 10.1158/1078-0432.CCR-18-1811. 30617136

[444] S. Nair , J. B. Wang , S. T. Tsao , et al., “Functional Improvement of Chimeric Antigen Receptor Through Intrinsic Interleukin‐15Rα Signaling,” Current Gene Therapy 19, no. 1 (2019): 40–53, 10.2174/1566523218666181116093857. 30444200

[445] D. Sommermeyer , M. Hudecek , P. L. Kosasih , et al., “Chimeric Antigen Receptor‐Modified T Cells Derived from Defined CD8+ and CD4+ Subsets Confer Superior Antitumor Reactivity in Vivo,” Leukemia 30, no. 2 (2016): 492, 10.1038/leu.2015.247. 26369987 PMC4746098

[446] S. A. Doydora , D. Franklin , P. Sun , et al., “Alum and Rainfall Effects on Ionophores in Runoff From Surface‐Applied Broiler Litter,” Journal of Environmental Quality 44, no. 5 (2015): 1657, 10.2134/jeq2015.02.0099. 26436282

[447] J. A. Kraemer , M. L. Erb , C. A. Waddling , et al., “A Phage Tubulin Assembles Dynamic Filaments by an Atypical Mechanism to Center Viral DNA Within the Host Cell,” Cell 149, no. 7 (2012): 1488, 10.1016/j.cell.2012.04.034. 22726436 PMC3401054

[448] X. Pan , J. Wang , L. Zhang , G. Li , and B. Huang , “Metabolic Plasticity of T Cell Fate Decision,” Chinese Medical Journal 137, no. 7 (2024): 762, 10.1097/CM9.0000000000002989. 38086394 PMC10997312

[449] J. G. Baldwin , C. Heuser‐Loy , T. Saha , et al., “Intercellular Nanotube‐Mediated Mitochondrial Transfer Enhances T Cell Metabolic Fitness and Antitumor Efficacy,” Cell 187, no. 23 (2024): 6614, 10.1016/j.cell.2024.08.029. 39276774 PMC11623344

[450] H. Tian , G. Wang , Q. Wang , et al., “Complement C1q Binding Protein Regulates T Cells' Mitochondrial Fitness to Affect Their Survival, Proliferation, and Anti–Tumor Immune Function,” Cancer Science 113, no. 3 (2022): 875, 10.1111/cas.15261. 34978120 PMC8898709

[451] X. Zhu , J. Chen , W. Li , et al., “Hypoxia‐Responsive CAR‐T Cells Exhibit Reduced Exhaustion and Enhanced Efficacy in Solid Tumors,” Cancer Research 84, no. 1 (2024): 84, 10.1158/0008-5472.CAN-23-1038. 37874330

[452] Y. Shi , I. S. Kotchetkov , A. Dobrin , et al., “GLUT1 Overexpression Enhances CAR T Cell Metabolic Fitness and Anti‐tumor Efficacy,” Molecular Therapy 32, no. 7 (2024): 2393, 10.1016/j.ymthe.2024.05.006. 38720457 PMC11286825

[453] J. A. Guerrero , D. D. Klysz , Y. Chen , et al., “GLUT1 Overexpression in CAR‐T Cells Induces Metabolic Reprogramming and Enhances Potency,” Nature Communications 15, no. 1 (2024): 8658, 10.1038/s41467-024-52666-y. PMC11456602 39370422

[454] D. Alizadeh , R. A. Wong , X. Yang , et al., “IL15 Enhances CAR‐T Cell Antitumor Activity by Reducing mTORC1 Activity and Preserving Their Stem Cell Memory Phenotype,” Cancer Immunology Research 7, no. 5 (2019): 759, 10.1158/2326-6066.CIR-18-0466. 30890531 PMC6687561

[455] C. Yan , L. Zheng , S. Jiang , et al., “Exhaustion‐Associated Cholesterol Deficiency Dampens the Cytotoxic Arm of Antitumor Immunity,” Cancer Cell 41, no. 7 (2023): 1276, 10.1016/j.ccell.2023.04.016. 37244259

[456] M. Shao , X. Teng , X. Guo , et al., “Inhibition of Calcium Signaling Prevents Exhaustion and Enhances Anti‐Leukemia Efficacy of CAR‐T Cells via SOCE‐Calcineurin‐NFAT and Glycolysis Pathways,” Advanced Science 9, no. 9 (2022): 2103508, 10.1002/advs.202103508. 35032108 PMC8948559

[457] L. Fultang , S. Booth , O. Yogev , et al., “Metabolic Engineering Against the Arginine Microenvironment Enhances CAR‐T Cell Proliferation and Therapeutic Activity,” Blood 136, no. 10 (2020): 1155, 10.1182/blood.2019004500. 32573723 PMC7565134

[458] Y. Wang , C. Tong , H. Dai , et al., “Low‐Dose Decitabine Priming Endows CAR T Cells with Enhanced and Persistent Antitumour Potential via Epigenetic Reprogramming,” Nature Communications 12, no. 1 (2021): 409, 10.1038/s41467-020-20696-x. PMC7814040 33462245

[459] J. Zeng , Y. Sun , Y. Fang , et al., “Unleashing the Potential of a Low CpG Passer Transposon for Superior CAR‐T Cell Therapy,” Frontiers in Immunology 16 (2025): 1541653, 10.3389/fimmu.2025.1541653. 39981247 PMC11840574

[460] M. Zhu , Y. Han , T. Gu , et al., “Class I HDAC Inhibitors Enhance Antitumor Efficacy and Persistence of CAR‐T Cells by Activation of the Wnt Pathway,” Cell Reports 43, no. 4 (2024): 114065, 10.1016/j.celrep.2024.114065. 38578828

[461] M. Luu , Z. Riester , A. Baldrich , et al., “Microbial Short‐Chain Fatty Acids Modulate CD8+ T Cell Responses and Improve Adoptive Immunotherapy for Cancer,” Nature Communications 12, no. 1 (2021): 4077, 10.1038/s41467-021-24331-1. PMC8249424 34210970

[462] J. Zhang , J. Li , Y. Hou , et al., “Osr2 Functions as a Biomechanical Checkpoint to Aggravate CD8+ T Cell Exhaustion in Tumor,” Cell 187, no. 13 (2024): 3409, 10.1016/j.cell.2024.04.023. 38744281

[463] L. Miao , Z. Zhang , Z. Ren , F. Tang , and Y. Li , “Obstacles and Coping Strategies of CAR‐T Cell Immunotherapy in Solid Tumors,” Frontiers in Immunology 12 (2021): 687822, 10.3389/fimmu.2021.687822. 34093592 PMC8170155

[464] S. Ghassemi , J. S. Durgin , S. Nunez‐Cruz , et al., “Rapid Manufacturing of Non‐Activated Potent CAR T Cells,” Nature Biomedical Engineering 6, no. 2 (2022): 118–128, 10.1038/s41551-021-00842-6. PMC8860360 35190680

[465] M. J. Dickinson , P. Barba , U. Jager , et al., “A Novel Autologous CAR‐T Therapy, YTB323, With Preserved T‐Cell Stemness Shows Enhanced CAR T‐Cell Efficacy in Preclinical and Early Clinical Development,” Cancer Discovery 13, no. 9 (2023): 1982, 10.1158/2159-8290.CD-22-1276. 37249512 PMC10481129

[466] X. Dong , J. Ren , Z. Amoozgar , et al., “Anti‐VEGF Therapy Improves EGFR‐vIII‐CAR‐T Cell Delivery and Efficacy in Syngeneic Glioblastoma Models in Mice,” Journal for ImmunoTherapy of Cancer 11, no. 3 (2023): 005583, 10.1136/jitc-2022-005583. PMC10008211 36898734

[467] N. Zheng , J. Fang , G. Xue , et al., “Induction of Tumor Cell Autosis by Myxoma Virus‐Infected CAR‐T and TCR‐T Cells to Overcome Primary and Acquired Resistance,” Cancer Cell 40, no. 9 (2022): 973–985, 10.1016/j.ccell.2022.08.001. 36027915 PMC9489043

[468] E. A. Chong , C. Alanio , J. Svoboda , et al., “Pembrolizumab for B‐Cell Lymphomas Relapsing after or Refractory to CD19‐Directed CAR T‐Cell Therapy,” Blood 139, no. 7 (2022): 1026, 10.1182/blood.2021012634. 34496014 PMC9211527

[469] E. W. Weber , K. R. Parker , E. Sotillo , et al., “Transient Rest Restores Functionality in Exhausted CAR‐T Cells Through Epigenetic Remodeling,” Science 372, no. 6537 (2021): aba1786. 10.1126/science.aba1786 PMC8049103 33795428

[470] I. Diaz‐Cano , L. Paz‐Ares , and I. Otano , “Chapter Six ‐ Adoptive Tumor Infiltrating Lymphocyte Transfer as Personalized Immunotherapy,” in International Review of Cell and Molecular Biology, eds. F. Aranda , P. Berraondo , and L. Galluzzi , Vol. 370 (Elsevier, 2022), 163–192. 10.1016/bs.ircmb.2022.04.003 35798505

[471] C. Wrzesinski , C. M. Paulos , A. Kaiser , et al., “Increased Intensity Lymphodepletion Enhances Tumor Treatment Efficacy of Adoptively Transferred Tumor‐Specific T Cells,” Journal of Immunotherapy 33, no. 1 (2010): 1–7, 10.1097/CJI.0b013e3181b88ffc. 19952961 PMC3247626

[472] F. Chen , Z. Zou , J. Du , et al., “Neoantigen Identification Strategies Enable Personalized Immunotherapy in Refractory Solid Tumors,” Journal of Clinical Investigation 129, no. 5 (2019): 2056, 10.1172/JCI99538. 30835255 PMC6486339

[473] A. Harari , M. Graciotti , M. Bassani‐Sternberg , and L. E. Kandalaft , “Antitumour Dendritic Cell Vaccination in a Priming and Boosting Approach,” Nature Reviews Drug Discovery 19, no. 9 (2020): 635–652, 10.1038/s41573-020-0074-8. 32764681

[474] K. Reinhard , B. Rengstl , P. Oehm , et al., “An RNA Vaccine Drives Expansion and Efficacy of Claudin‐CAR‐T Cells Against Solid Tumors,” Science 367, no. 6476 (2020): 446–453, 10.1126/science.aay5967. 31896660

[475] J. Chiffelle , D. Barras , R. Petremand , et al., “Tumor‐Reactive T Cell Clonotype Dynamics Underlying Clinical Response to TIL Therapy in Melanoma,” Immunity 57, no. 10 (2024): 2466–2482, 10.1016/j.immuni.2024.08.014. 39276771

[476] S. Yunger , B. Geiger , N. Friedman , M. J. Besser , and S. Adutler‐Lieber , “Modulating the Proliferative and Cytotoxic Properties of Patient‐Derived TIL by a Synthetic Immune Niche of Immobilized CCL21 and ICAM1,” Frontiers in Oncology 13 (2023): 1116328, 10.3389/fonc.2023.1116328. 36937426 PMC10020329

[477] Z. Liu , Q. Meng , J. Bartek , et al., “Tumor‐Infiltrating Lymphocytes (TILs) from Patients with Glioma,” Oncoimmunology 6, no. 2 (2017): 1252894. 10.1080/2162402X.2016.1252894 PMC5353900 28344863

[478] L. G. Radvanyi , C. Bernatchez , M. Zhang , et al., “Specific Lymphocyte Subsets Predict Response to Adoptive Cell Therapy Using Expanded Autologous Tumor‐Infiltrating Lymphocytes in Metastatic Melanoma Patients,” Clinical Cancer Research 18, no. 24 (2012): 6758–6770, 10.1158/1078-0432.CCR-12-1177. 23032743 PMC3525747

[479] S. Krishna , F. J. Lowery , A. R. Copeland , et al., “Stem‐Like CD8 T Cells Mediate Response of Adoptive Cell Immunotherapy Against human Cancer,” Science 370, no. 6522 (2020): 1328–1334, 10.1126/science.abb9847. 33303615 PMC8883579

[480] A. Kurzay , S. Fresnillo Salo , A. Rahbech , et al., “ProS1‐MerTK Signaling in CD4 T Cells: Implications for TIL Expansion and Functionality,” Oncoimmunology 14, no. 1 (2025): 2532662. 40653768 10.1080/2162402X.2025.2532662 PMC12269690

[481] P. Karagiannis , S. Iriguchi , and S. Kaneko , “Reprogramming Away From the Exhausted T Cell State,” Seminars in Immunology 28, no. 1 (2016): 35–44, 10.1016/j.smim.2015.10.007. 26589493

[482] Z. Sun , A. Xu , Z. Wu , et al., “Effect of Hypoxia‐Induced mIL15 Expression on Expansion and Memory Progenitor Stem‐Like TILs In Vitro,” Frontiers in Immunology 15 (2024): 1450245, 10.3389/fimmu.2024.1450245. 39650651 PMC11621077

[483] D. Xiong , L. Zhang , and Z. J. Sun , “Targeting the Epigenome to Reinvigorate T Cells for Cancer Immunotherapy,” Mil Med Res 10, no. 1 (2023): 59. 38044445 10.1186/s40779-023-00496-2 PMC10694991

[484] M. Philip , L. Fairchild , L. Sun , et al., “Chromatin States Define Tumour‐Specific T Cell Dysfunction and Reprogramming,” Nature 545, no. 7655 (2017): 452–456, 10.1038/nature22367. 28514453 PMC5693219

[485] T. J. DuCote , X. Song , K. J. Naughton , et al., “EZH2 Inhibition Promotes Tumor Immunogenicity in Lung Squamous Cell Carcinomas,” Cancer Research Communications 4, no. 2 (2024): 388–403, 10.1158/2767-9764.CRC-23-0399. 38265267 PMC10863487

[486] A. N. Henning , R. Roychoudhuri , and N. P. Restifo , “Epigenetic Control of CD8+ T Cell Differentiation,” Nature Reviews Immunology 18, no. 5 (2018): 340–356, 10.1038/nri.2017.146. PMC6327307 29379213

[487] Y. Kagoya , M. Nakatsugawa , Y. Yamashita , et al., “BET Bromodomain Inhibition Enhances T Cell Persistence and Function in Adoptive Immunotherapy Models,” Journal of Clinical Investigation 126, no. 9 (2016): 3479, 10.1172/JCI86437. 27548527 PMC5004946

[488] P. D. Langridge and G. Struhl , “Epsin‐Dependent Ligand Endocytosis Activates Notch by Force,” Cell 171, no. 6 (2017): 1383–1396, 10.1016/j.cell.2017.10.048. 29195077 PMC6219616

[489] M. D. Hellmann , P. A. Janne , M. Opyrchal , et al., “Entinostat Plus Pembrolizumab in Patients With Metastatic NSCLC Previously Treated With Anti–PD‐(L)1 Therapy,” Clinical Cancer Research 27, no. 4 (2021): 1019–1028, 10.1158/1078-0432.CCR-20-3305. 33203644 PMC7887114

[490] H. Song , H. Wang , M. Gong , et al., “Augmentation of Antitumor Function of Tumor‐Infiltrating Lymphocytes Against Triple‐Negative Breast Cancer by PD‐1 Blockade,” Cell Biology International 46, no. 2 (2022): 278–287, 10.1002/cbin.11729. 34854515

[491] C. Zhang , Y. Sun , S. Li , et al., “Autophagic Flux Restoration Enhances the Antitumor Efficacy of Tumor Infiltrating Lymphocytes,” Journal for ImmunoTherapy of Cancer 10, no. 10 (2022): 004868. 10.1136/jitc-2022-004868 PMC9621197 36307150

[492] X. Che , S. Zheng , Y. Sun , et al., “Multi‐Engineered T Cell Vaccine Boosting TCR‐T Cell Therapy Enhances Anti‐Tumor Function and Eradicates Heterogeneous Solid Tumors,” Molecular Therapy 33, no. 9 (2025): 4529–4551, 10.1016/j.ymthe.2025.05.036. 40450522 PMC12432877

[493] Y. Chen , D. Ouyang , Y. Wang , et al., “EBV Promotes TCR‐T‐Cell Therapy Resistance by Inducing CD163+M2 Macrophage Polarization and MMP9 Secretion,” Journal for ImmunoTherapy of Cancer 12, no. 6 (2024): 008375. 10.1136/jitc-2023-008375 PMC11184188 38886114

[494] R. Abu Eid , K. M. Friedman , M. Mkrtichyan , et al., “Akt1 and ‐2 Inhibition Diminishes Terminal Differentiation and Enhances Central Memory CD8 + T‐cell Proliferation and Survival,” Oncoimmunology 4, no. 5 (2015): 1005448, 10.1080/2162402X.2015.1005448. PMC4485779 26155399

[495] C. B. Nava Lauson , S. Tiberti , P. A. Corsetto , et al., “Linoleic Acid Potentiates CD8+ T Cell Metabolic Fitness and Antitumor Immunity,” Cell Metabolism 35, no. 4 (2023): 633–650, 10.1016/j.cmet.2023.02.013. 36898381

[496] A. Nguyen , D. Brown , R. Krishnan , et al., “HDACi‐Dependent Microenvironmental Normalization Overcomes Tumor Burden–Induced T‐cell Exhaustion,” Clinical Cancer Research 29, no. 20 (2023): 4289–4305, 10.1158/1078-0432.CCR-22-2181. 37561398

[497] E. Vercher , A. Covo‐Vergara , E. Conde , et al., “Human T Cells Engineered With an HLA‐A2‐Restricted Murine T‐cell Receptor Targeting Glypican 3 Effectively Control Human Hepatocellular Carcinoma in Mice,” Hepatology 82, no. 2 (2025): 326–343, 10.1097/HEP.0000000000001175. 39601444 PMC12266797

[498] S. C. Lu , M. J. Hansen , J. R. Hemsath , B. J. Parrett , B. N. Zell , and M. A. Barry , “Modulating Oncolytic Adenovirus Immunotherapy by Driving Two Axes of the Immune System by Expressing 4‐1BBL and CD40L,” Human Gene Therapy 33, no. 5‐6 (2022): 250, 10.1089/hum.2021.197. 34731019 PMC11981553

[499] S. Basnet , M. Van der Heijden , D. C. A. Quixabeira , et al., “Overcoming Effector T Cell Exhaustion in Ovarian Cancer Ascites with a Novel Adenovirus Encoding for a MUC1 Bispecific Antibody Engager and IL‐2 Cytokine,” Molecular Therapy 32, no. 9 (2024): 3114, 10.1016/j.ymthe.2024.06.029. 38910324 PMC11403222

[500] V. Arias , T. V. Kudling , J. H. A. Clubb , et al., “Boosting Anti‐Tumor Immunity With TILT‐517 Oncolytic Adenovirus and Checkpoint Blockade in Renal Cell Carcinoma,” Molecular Therapy Oncology 33, no. 2 (2025): 200979, 10.1016/j.omton.2025.200979. 40589567 PMC12208612

[501] N. M. Durham , K. Mulgrew , K. McGlinchey , et al., “Oncolytic VSV Primes Differential Responses to Immuno‐Oncology Therapy,” Molecular Therapy 25, no. 8 (2017): 1917–1932, 10.1016/j.ymthe.2017.05.006. 28578991 PMC5542805

[502] E. J. West , K. J. Scott , E. Tidswell , et al., “Intravenous Oncolytic Vaccinia Virus Therapy Results in a Differential Immune Response Between Cancer Patients,” Cancers 14, no. 9 (2022): 2181, 10.3390/cancers14092181. 35565310 PMC9103071

[503] A. Morales‐Molina , M. A. Rodriguez‐Milla , A. Gimenez‐Sanchez , A. J. Perise‐Barrios , and J. Garcia‐Castro , “Cellular Virotherapy Increases Tumor‐Infiltrating Lymphocytes (TIL) and Decreases Their PD‐1^+^ Subsets in Mouse Immunocompetent Models,” Cancers 12, no. 7 (2020): 1920. 32708639 10.3390/cancers12071920 PMC7409201

[504] I. Liikanen , S. Basnet , D. C. A. Quixabeira , et al., “Oncolytic adenovirus Decreases the Proportion of TIM‐3 + Subset of Tumor‐Infiltrating CD8 + T Cells With Correlation to Improved Survival in Patients with Cancer,” Journal for ImmunoTherapy of Cancer 10, no. 2 (2022): 003490, 10.1136/jitc-2021-003490. PMC8867324 35193929

[505] S. Liu , F. Li , Q. Ma , et al., “OX40L‐Armed Oncolytic Virus Boosts T‐Cell Response and Remodels Tumor Microenvironment for Pancreatic Cancer Treatment,” Theranostics 13, no. 12 (2023): 4016, 10.7150/thno.83495. 37554264 PMC10405835

[506] E. Riva , S. Carboni , W. di Berardino‐Besson , et al., “Bimodal Effect of NKG2A Blockade on Intratumoral and Systemic CD8 T Cell Response Induced by Cancer Vaccine,” Cancers (Basel) 16, no. 11 (2024): 2036. 38893156 10.3390/cancers16112036 PMC11171001

[507] J. Huang , M. Zheng , Z. Zhang , et al., “Interleukin‐7‐Loaded Oncolytic Adenovirus Improves CAR‐T Cell Therapy for Glioblastoma,” Cancer Immunology, Immunotherapy 70, no. 9 (2021): 2453–2465, 10.1007/s00262-021-02856-0. 33543339 PMC10991970

[508] A. Morales‐Molina , S. Gambera , A. Leo , and J. Garcia‐Castro , “Combination Immunotherapy Using G‐CSF and Oncolytic Virotherapy Reduces Tumor Growth in Osteosarcoma,” Journal for ImmunoTherapy of Cancer 9, no. 3 (2021): 001703, 10.1136/jitc-2020-001703. PMC7978281 33737338

[509] M. Wei , S. Zuo , Z. Chen , et al., “Oncolytic Vaccinia Virus Expressing a Bispecific T‐cell Engager Enhances Immune Responses in EpCAM Positive Solid Tumors,” Frontiers in Immunology 13 (2022): 1017574, 10.3389/fimmu.2022.1017574. 36451817 PMC9702515

[510] J. Chen , B. R. Madina , E. Ahmadi , et al., “Cancer Immunotherapy With Enveloped Self‐Amplifying mRNA CARG‐2020 That Modulates IL‐12, IL‐17 and PD‐L1 Pathways to Prevent Tumor Recurrence,” Acta Pharmaceutica Sinica B 14, no. 1 (2024): 335–349, 10.1016/j.apsb.2023.08.034. 38261838 PMC10792965

[511] A. H. Twing , S. Gokhale , B. Slostad , et al., “Impact of Transcatheter Aortic Valve Implantation among Patients With Co‐Existing Mild to Moderate Mitral Regurgitation,” The American Journal of Cardiology 177 (2022): 84–89, 10.1016/j.amjcard.2022.04.049. 35732551

[512] W. Wang , X. Li , R. Hu , et al., “BET Inhibitor in Combination With BCG Vaccine Enhances Antitumor Efficacy and Orchestrates T Cell Reprogramming for Melanoma,” Cell Reports Medicine 6, no. 3 (2025): 101995, 10.1016/j.xcrm.2025.101995. 40107246 PMC11970395

[513] M. Coppey , S. Dasgupta , and T. G. Spiro , “Resonance Raman Spectroscopic Evidence That Carp Deoxyhemoglobin Remains in a T‐Like Quaternary Structure at High pH: Implications for Cooperativity,” Biochemistry 25, no. 8 (1986): 1940–1944, 10.1021/bi00356a016. 3707921

[514] M. H. Qazilbash , N. Y. Saini , S. C. Cha , et al., “A Randomized Phase 2 Trial of Idiotype Vaccination and Adoptive Autologous T‐Cell Transfer in Patients With Multiple Myeloma,” Blood 139, no. 9 (2022): 1289–1301, 10.1182/blood.2020008493. 34521108 PMC8900281

[515] Y. Liu , J. Pagacz , D. J. Wolfgeher , K. D. Bromerg , J. V. Gorman , and S. J. Kron , “Senescent Cancer Cell Vaccines Induce Cytotoxic T Cell Responses Targeting Primary Tumors and Disseminated Tumor Cells,” Journal for ImmunoTherapy of Cancer 11, no. 2 (2023): 005862. 10.1136/jitc-2022-005862 PMC9933761 36792123

[516] J. L. Tanyi , S. Bobisse , E. Ophir , et al., “Personalized Cancer Vaccine Effectively Mobilizes Antitumor T Cell Immunity in Ovarian Cancer,” Science Translational Medicine 10, no. 436 (2018): aao5931. 10.1126/scitranslmed.aao5931 29643231

[517] A. Paladhi , S. Daripa , I. Mondal , and S. K. Hira , “Targeting Thymidine Phosphorylase Alleviates Resistance to Dendritic Cell Immunotherapy in Colorectal Cancer and Promotes Antitumor Immunity,” Frontiers in Immunology 13 (2022): 988071, 10.3389/fimmu.2022.988071. 36090972 PMC9449540

[518] R. Wang , H. Li , S. He , et al., “Spatiotemporal Nano‐Regulator Unleashes Anti‐Tumor Immunity by Overcoming Dendritic Cell Tolerance and T Cell Exhaustion in Tumor‐Draining Lymph Nodes,” Advanced Materials 37, no. 5 (2025): 2412141, 10.1002/adma.202412141. 39663685

[519] P. Liu , L. Zhao , G. Kroemer , and O. Kepp , “PD‐L1+ Macrophages Suppress T Cell‐Mediated Anticancer Immunity,” Oncoimmunology 13, no. 1 (2024): 2338951. 38590800 10.1080/2162402X.2024.2338951 PMC11000604

[520] S. P. Lau , N. van Montfoort , P. Kinderman , et al., “Dendritic Cell Vaccination and CD40‐Agonist Combination Therapy Licenses T Cell‐Dependent Antitumor Immunity in a Pancreatic Carcinoma Murine Model,” Journal for ImmunoTherapy of Cancer 8, no. 2 (2020): 000772, 10.1136/jitc-2020-000772. PMC7373331 32690771

[521] J. Chen , Y. Gao , J. Zhong , et al., “Lnc‐H19‐Derived Protein Shapes the Immunosuppressive Microenvironment of Glioblastoma,” Cell Reports Medicine 5, no. 11 (2024): 101806, 10.1016/j.xcrm.2024.101806. 39481387 PMC11604490

[522] Y. Xiang , M. Tian , J. Huang , et al., “LMP2‐mRNA Lipid Nanoparticle Sensitizes EBV‐Related Tumors to Anti‐PD‐1 Therapy by Reversing T Cell Exhaustion,” Journal of Nanobiotechnology 21, no. 1 (2023): 324, 10.1186/s12951-023-02069-w. 37679769 PMC10486025

[523] K. Qiu , X. Duan , M. Mao , et al., “mRNA‐LNP Vaccination‐Based Immunotherapy Augments CD8+ T Cell Responses against HPV‐Positive Oropharyngeal Cancer,” NPJ Vaccines 8, no. 1 (2023): 144, 10.1038/s41541-023-00733-8. 37773254 PMC10542330

[524] P. Czerwinska , M. Rucinski , N. Wlodarczyk , et al., “Therapeutic Melanoma Vaccine with Cancer Stem Cell Phenotype Represses Exhaustion and Maintains Antigen‐Specific T Cell Stemness by Up‐Regulating BCL6,” Oncoimmunology 9, no. 1 (2020): 1710063. 32002306 10.1080/2162402X.2019.1710063 PMC6959432

[525] C. Gao , R. Luo , C. H. T. Kwong , et al., “Cancer Vaccine from Intracellularly Gelated Tumor Cells Functionalized with CD47 Blockage and Damage‐associated Molecular Pattern Exposure,” Cell Reports Medicine 6, no. 5 (2025): 102092, 10.1016/j.xcrm.2025.102092. 40345180 PMC12147843

[526] X. Zhang , G. Liu , X. Shi , et al., “Sequential Administration of Anti‐PD‐1 and Anti‐Tim‐3 Combined With an SA‐GM‐CSF‐Anchored Vaccine Overcomes Adaptive Immune Resistance to Reject Established Bladder Cancer,” Journal of Cancer 12, no. 7 (2021): 2000, 10.7150/jca.44769. 33753998 PMC7974521

[527] T. Tada , T. D. Norton , R. Leibowitz , and N. R. Landau , “Checkpoint Inhibitor‐expressing Lentiviral Vaccine Suppresses Tumor Growth in Preclinical Cancer Models,” Journal for ImmunoTherapy of Cancer 12, no. 4 (2024): 008761, 10.1136/jitc-2023-008761. PMC11043704 38658032

[528] A. E. Fan , H. Sultan , T. Kumai , et al., “STAT5 Activation Enhances Adoptive Therapy Combined With Peptide Vaccination by Preventing PD‐1 Inhibition,” Molecular Cancer Therapeutics 24, no. 3 (2025): 419–430, 10.1158/1535-7163.MCT-24-0505. 39582348 PMC11879759

[529] F. Baharom , R. A. Ramirez‐Valdez , K. K. S. Tobin , et al., “Intravenous Nanoparticle Vaccination Generates Stem‐Like TCF1+ Neoantigen‐Specific CD8+ T Cells,” Nature Immunology 22, no. 1 (2021): 41–52, 10.1038/s41590-020-00810-3. 33139915 PMC7746638

[530] H. Liu , K. D. Moynihan , Y. Zheng , et al., “Structure‐Based Programming of Lymph‐node Targeting in Molecular Vaccines,” Nature 507, no. 7493 (2014): 519–522, 10.1038/nature12978. 24531764 PMC4069155

[531] S. Pant , Z. A. Wainberg , C. D. Weekes , et al., “Lymph‐Node‐Targeted, mKRAS‐Specific Amphiphile Vaccine in Pancreatic and Colorectal Cancer: The Phase 1 AMPLIFY‐201 Trial,” Nature Medicine 30, no. 2 (2024): 531–542, 10.1038/s41591-023-02760-3. PMC10878978 38195752

[532] S. Spranger , M. Javorovic , M. Burdek , et al., “Generation of Th1‐Polarizing Dendritic Cells Using the TLR7/8 Agonist CL075,” The Journal of Immunology 185, no. 1 (2010): 738–747, 10.4049/jimmunol.1000060. 20511554

[533] K. I. Woroniecka , K. E. Rhodin , C. Dechant , et al., “4‐1BB Agonism Averts TIL Exhaustion and Licenses PD‐1 Blockade in Glioblastoma and Other Intracranial Cancers,” Clinical Cancer Research 26, no. 6 (2020): 1349–1358, 10.1158/1078-0432.CCR-19-1068. 31871298 PMC7073290

[534] G. Leem , J. Park , M. Jeon , et al., “4‐1BB Co‐Stimulation Further Enhances Anti‐PD‐1‐Mediated Reinvigoration of Exhausted CD39 + CD8 T Cells from Primary and Metastatic Sites of Epithelial Ovarian Cancers,” Journal for ImmunoTherapy of Cancer 8, no. 2 (2020): 001650, 10.1136/jitc-2020-001650. PMC7745695 33335029

[535] A. Malyshkina , E. Littwitz‐Salomon , K. Sutter , et al., “Chronic Retroviral Infection of Mice Promotes Tumor Development, but CD137 Agonist Therapy Restores Effective Tumor Immune Surveillance,” Cancer Immunology, Immunotherapy 68, no. 3 (2019): 479–488, 10.1007/s00262-019-02300-4. 30635687 PMC11028158

[536] Z. C. Schmiechen , H. A. Nanda , A. L. Burrack , et al., “IL‐15 Complex Enhances Agonistic Anti‐CD40^+^ Anti‐PDL1 by Correcting the T‐bet to Tox Ratio in CD8^+^ T Cells Infiltrating Pancreatic Ductal Adenocarcinoma,” Cancer Immunology Research 13, no. 6 (2025): 847–886, 10.1158/2326-6066.CIR-24-0758. 40072469 PMC12134750

[537] T. Jiang , Z. Yang , Q. Su , et al., “Bivalent OX40 Aptamer and CpG as Dual Agonists for Cancer Immunotherapy,” ACS Applied Materials & Interfaces 17, no. 5 (2025): 7353–7362, 10.1021/acsami.4c18550. 39841045

[538] D. J. Messenheimer , S. M. Jensen , M. E. Afentoulis , et al., “Timing of PD‐1 Blockade Is Critical to Effective Combination Immunotherapy With Anti‐OX40,” Clinical Cancer Research 23, no. 20 (2017): 6165–6177, 10.1158/1078-0432.CCR-16-2677. 28855348 PMC5641261

[539] D. A. Knee , B. Hewes , and J. L. Brogdon , “Rationale for Anti‐GITR Cancer Immunotherapy,” European Journal of Cancer 67 (2016): 1–10, 10.1016/j.ejca.2016.06.028. 27591414

[540] A. E. Mahne , S. Mauze , B. Joyce‐Shaikh , et al., “Dual Roles for Regulatory T‐cell Depletion and Costimulatory Signaling in Agonistic GITR Targeting for Tumor Immunotherapy,” Cancer Research 77, no. 5 (2017): 1108–1118, 10.1158/0008-5472.CAN-16-0797. 28122327

[541] A. Debesset , C. Pilon , S. Meunier , et al., “TNFR2 blockade Promotes Antitumoral Immune Response in PDAC by Targeting Activated Treg and Reducing T Cell Exhaustion,” Journal for ImmunoTherapy of Cancer 12, no. 11 (2024): 008898, 10.1136/jitc-2024-008898. PMC11580249 39562007

[542] S. Yasmin‐Karim , P. T. Bruck , M. Moreau , et al., “Radiation and Local Anti‐CD40 Generate an Effective In Situ Vaccine in Preclinical Models of Pancreatic Cancer,” Frontiers in Immunology 9 (2018): 2030, 10.3389/fimmu.2018.02030. 30245691 PMC6137176

[543] J. Busselaar , S. Tian , H. van Eenennaam , and J. Borst , “Helpless Priming Sends CD8^+^ T Cells on the Road to Exhaustion,” Frontiers in Immunology 11 (2020): 592569, 10.3389/fimmu.2020.592569. 33123174 PMC7573232

[544] J. Duraiswamy , R. Turrini , A. Minasyan , et al., “Myeloid Antigen‐Presenting Cell Niches Sustain Antitumor T Cells and License PD‐1 Blockade via CD28 Costimulation,” Cancer Cell 39, no. 12 (2021): 1623–1642, 10.1016/j.ccell.2021.10.008. 34739845 PMC8861565

[545] A. E. Oja , B. Piet , D. van der Zwan , et al., “Functional Heterogeneity of CD4^+^ Tumor‐Infiltrating Lymphocytes With a Resident Memory Phenotype in NSCLC,” Frontiers in Immunology 9 (2018): 2654, 10.3389/fimmu.2018.02654. 30505306 PMC6250821

[546] R. H. Vonderheide and M. J. Glennie , “Agonistic CD40 Antibodies and Cancer Therapy,” Clinical Cancer Research 19, no. 5 (2013): 1035–1043, 10.1158/1078-0432.CCR-12-2064. 23460534 PMC3590838

[547] L. M. Snell , B. L. MacLeod , J. C. Law , et al., “CD8^+^ T Cell Priming in Established Chronic Viral Infection Preferentially Directs Differentiation of Memory‐Like Cells for Sustained Immunity,” Immunity 49, no. 4 (2018): 678–694, 10.1016/j.immuni.2018.08.002. 30314757 PMC8060917

[548] M. E. Goebeler and R. C. Bargou , “T Cell‐Engaging Therapies — BiTEs and Beyond,” Nature Reviews Clinical Oncology 17, no. 7 (2020): 418–434, 10.1038/s41571-020-0347-5. 32242094

[549] A. Berezhnoy , B. J. Sumrow , K. Stahl , et al., “Development and Preliminary Clinical Activity of PD‐1‐Guided CTLA‐4 Blocking Bispecific DART Molecule,” Cell Reports Medicine 1, no. 9 (2020): 100163, 10.1016/j.xcrm.2020.100163. 33377134 PMC7762776

[550] N. van de Donk , C. O'Neill , M. E. M. de Ruijter , C. P. M. Verkleij , and S. Zweegman , “T‐cell Redirecting Bispecific and Trispecific Antibodies in Multiple Myeloma Beyond BCMA,” Current Opinion in Oncology 35, no. 6 (2023): 601–611, 10.1097/CCO.0000000000000983. 37501530 PMC10566598

[551] C. H. Chang , Y. Wang , R. Li , et al., “Combination Therapy With Bispecific Antibodies and PD‐1 Blockade Enhances the Antitumor Potency of T Cells,” Cancer Research 77, no. 19 (2017): 5384–5394, 10.1158/0008-5472.CAN-16-3431. 28819027

[552] C. Krupka , P. Kufer , R. Kischel , et al., “Blockade of the PD‐1/PD‐L1 Axis Augments Lysis of AML Cells by the CD33/CD3 BiTE Antibody Construct AMG 330: Reversing a T‐Cell‐Induced Immune Escape Mechanism,” Leukemia 30, no. 2 (2016): 484–491, 10.1038/leu.2015.214. 26239198

[553] T. Osada , S. P. Patel , S. A. Hammond , K. Osada , M. A. Morse , and H. K. Lyerly , “CEA/CD3‐Bispecific T Cell‐Engaging (BiTE) Antibody‐Mediated T Lymphocyte Cytotoxicity Maximized by Inhibition of Both PD1 and PD‐L1,” Cancer Immunology, Immunotherapy 64, no. 6 (2015): 677–688, 10.1007/s00262-015-1671-y. 25742933 PMC11029757

[554] T. T. Junttila , J. Li , J. Johnston , et al., “Antitumor Efficacy of a Bispecific Antibody That Targets HER2 and Activates T Cells,” Cancer Research 74, no. 19 (2014): 5561–5571, 10.1158/0008-5472.CAN-13-3622-T. 25228655

[555] J. P. Pei , Y. Wang , L. P. Ma , et al., “AXL Antibody and AXL‐ADC Mediate Antitumor Efficacy via Targeting AXL in Tumor‐Intrinsic Epithelial‐Mesenchymal Transition and Tumor‐Associated M2‐Like Macrophage,” Acta Pharmacologica Sinica 44, no. 6 (2023): 1290–1303, 10.1038/s41401-022-01047-6. 36650292 PMC10203350

[556] J. Rios‐Doria , J. Harper , R. Rothstein , et al., “Antibody–Drug Conjugates Bearing Pyrrolobenzodiazepine or Tubulysin Payloads Are Immunomodulatory and Synergize With Multiple Immunotherapies,” Cancer Research 77, no. 10 (2017): 2686–2698, 10.1158/0008-5472.CAN-16-2854. 28283653

[557] S. R. Woo , M. B. Fuertes , L. Corrales , et al., “STING‐Dependent Cytosolic DNA Sensing Mediates Innate Immune Recognition of Immunogenic Tumors,” Immunity 41, no. 5 (2014): 830–842, 10.1016/j.immuni.2014.10.017. 25517615 PMC4384884

[558] S. R. Woo , L. Corrales , and T. F. Gajewski , “The STING Pathway and the T Cell‐Inflamed Tumor Microenvironment,” Trends in Immunology 36, no. 4 (2015): 250–256, 10.1016/j.it.2015.02.003. 25758021 PMC4393801

[559] T. Ohkuri , A. Ghosh , A. Kosaka , et al., “STING Contributes to Antiglioma Immunity via Triggering Type I IFN Signals in the Tumor Microenvironment,” Cancer Immunology Research 2, no. 12 (2014): 1199–1208, 10.1158/2326-6066.CIR-14-0099. 25300859 PMC4258479

[560] O. Demaria , A. De Gassart , S. Coso , et al., “STING Activation of Tumor Endothelial Cells Initiates Spontaneous and Therapeutic Antitumor Immunity,” Proceedings of the National Academy of Sciences 112, no. 50 (2015): 15408–15413, 10.1073/pnas.1512832112. PMC4687570 26607445

[561] Y. T. Wu , Y. Fang , Q. Wei , et al., “Tumor‐Targeted Delivery of a STING Agonist Improves Cancer Immunotherapy,” Proceedings of the National Academy of Sciences 119, no. 49 (2022): 2214278119, 10.1073/pnas.2214278119. PMC9894229 36442099

[562] R. A. Bukhalid , J. R. Duvall , K. Lancaster , et al., “XMT‐2056, a HER2‐Directed STING Agonist Antibody–Drug Conjugate, Induces Innate Antitumor Immune Responses by Acting on Cancer Cells and Tumor‐Resident Immune Cells,” Clinical Cancer Research 31, no. 9 (2025): 1766–1782, 10.1158/1078-0432.CCR-24-2449. 40029253 PMC12010966

[563] L. C. Tsao , J. S. Wang , X. Ma , et al., “Effective Extracellular Payload Release and Immunomodulatory Interactions Govern the Therapeutic Effect of Trastuzumab Deruxtecan (T‐DXd),” Nature Communications 16, no. 1 (2025): 3167, 10.1038/s41467-025-58266-8. PMC11965298 40175391

[564] L. Rausch and A. Kallies , “Molecular Mechanisms Governing CD8 T Cell Differentiation and Checkpoint Inhibitor Response in Cancer,” Annual Review of Immunology 43, no. 1 (2025): 515–543, 10.1146/annurev-immunol-082223-044122. 40279308

[565] D. T. Utzschneider , M. Charmoy , V. Chennupati , et al., “T Cell Factor 1‐Expressing Memory‐Like CD8+ T Cells Sustain the Immune Response to Chronic Viral Infections,” Immunity 45, no. 2 (2016): 415–427, 10.1016/j.immuni.2016.07.021. 27533016

[566] W. Tian , G. Qin , M. Jia , et al., “Hierarchical Transcriptional Network Governing Heterogeneous T Cell Exhaustion and Its Implications for Immune Checkpoint Blockade,” Frontiers in Immunology 14 (2023): 1198551, 10.3389/fimmu.2023.1198551. 37398674 PMC10311999

[567] V. van der Heide , E. Humblin , A. Vaidya , and A. O. Kamphorst , “Advancing Beyond the Twists and Turns of T Cell Exhaustion in Cancer,” Science Translational Medicine 14, no. 670 (2022): abo4997, 10.1126/scitranslmed.abo4997. PMC10000016 36350991

[568] M. Z. Qiu , C. Wang , Z. Wu , et al., “Dynamic Single‐cell Mapping Unveils Epstein‒Barr Virus‐Imprinted T‐Cell Exhaustion and On‐Treatment Response,” Signal Transduction and Targeted Therapy 8, no. 1 (2023): 370, 10.1038/s41392-023-01622-1. 37735150 PMC10514267

[569] C. Chen , J. Liu , Y. Chen , et al., “Application of ATAC‐seq in Tumor‐Specific T Cell Exhaustion,” Cancer Gene Therapy 30, no. 1 (2023): 1–10, 10.1038/s41417-022-00495-w. 35794339 PMC9842510

[570] Z. Liu , Z. Yang , J. Wu , et al., “A Single‐Cell Atlas of Human Tumor Microenvironments,” Cell 188, no. 11 (2025): 3081.40147443

[571] P. Yang , K. Jin , Y. Yao , et al., “Spatial Integration of Multi‐omics Single‐cell Data with SIMO,” Nature Communications 16, no. 1 (2025): 1265, 10.1038/s41467-025-56523-4. PMC11787318 39893194

[572] T. Zhong , S. Sun , M. Zhao , B. Zhang , and H. Xiong , “The Mechanisms and Clinical Significance of CD8^+^ T Cell Exhaustion in Anti‐tumor Immunity,” Cancer Biol Med 22, no. 5 (2025): 460–480. 40492696 10.20892/j.issn.2095-3941.2024.0628 PMC12240197

[573] W. P. Lin , H. Li , and Z. J. Sun , “T Cell Exhaustion Initiates Tertiary Lymphoid Structures and Turbocharges Cancer‐Immunity Cycle,” EBioMedicine 104 (2024): 105154, 10.1016/j.ebiom.2024.105154. 38749300 PMC11108856

[574] M. K. Blake , P. O'Connell , and Y. A. Aldhamen , “Fundamentals to Therapeutics: Epigenetic Modulation of CD8^+^ T Cell Exhaustion in the Tumor Microenvironment,” Frontiers in Cell and Developmental Biology 10 (2022): 1082195, 10.3389/fcell.2022.1082195. 36684449 PMC9846628

[575] J. A. Belk , W. Yao , N. Ly , et al., “Genome‐wide CRISPR Screens of T Cell Exhaustion Identify Chromatin Remodeling Factors That Limit T Cell Persistence,” Cancer Cell 40, no. 7 (2022): 768–786, 10.1016/j.ccell.2022.06.001. 35750052 PMC9949532

[576] J. Zhao , Y. Li , J. Zhu , H. Li , and X. Jin , “Ubiquitination in Hepatocellular Carcinoma Immunity,” Journal of Translational Medicine 23, no. 1 (2025): 574, 10.1186/s12967-025-06592-2. 40410880 PMC12102898

[577] Y. Liu , H. Ni , J. Li , et al., “LARP4‐mediated Hypertranslation Drives T Cell Dysfunction in Tumors,” Nature Immunology 26, no. 9 (2025): 1488–1500, 10.1038/s41590-025-02232-5. 40696044

[578] Z. Z. Yang , D. M. Grote , B. Xiu , et al., “TGF‐β Upregulates CD70 Expression and Induces Exhaustion of Effector Memory T Cells in B‐cell Non‐Hodgkin's Lymphoma,” Leukemia 28, no. 9 (2014): 1872–1884, 10.1038/leu.2014.84. 24569779 PMC4145058

[579] M. A. Salkeni and A. Naing , “Interleukin‐10 in Cancer Immunotherapy: from Bench to Bedside,” Trends in Cancer 9, no. 9 (2023): 716–725, 10.1016/j.trecan.2023.05.003. 37321942 PMC10524969

[580] B. Palermo , O. Franzese , G. Frisullo , et al., “CD28/PD1 co‐expression: Dual Impact on CD8+ T Cells in Peripheral Blood and Tumor Tissue, and Its Significance in NSCLC Patients' Survival and ICB Response,” Journal of Experimental & Clinical Cancer Research 42, no. 1 (2023): 287, 10.1186/s13046-023-02846-3. 37898752 PMC10612243

[581] Z. Zhang , M. Langenbach , S. Sagar , et al., “Efficacy of CTLA‐4 Checkpoint Therapy Is Dependent on IL‐21 Signaling to Mediate Cytotoxic Reprogramming of PD‐1^+^CD8^+^ T Cells,” Nature Immunology 26, no. 1 (2025): 92–104, 10.1038/s41590-024-02027-0. 39702858 PMC12853267

[582] S. Hwang , D. A. Cobb , R. Bhadra , B. Youngblood , and I. A. Khan , “Blimp‐1–Mediated CD4 T Cell Exhaustion Causes CD8 T Cell Dysfunction during Chronic Toxoplasmosis,” Journal of Experimental Medicine 213, no. 9 (2016): 1799–1818, 10.1084/jem.20151995. 27481131 PMC4995081

[583] K. Yang , D. B. Blanco , G. Neale , et al., “Homeostatic Control of Metabolic and Functional Fitness of Treg Cells by LKB1 Signalling,” Nature 548, no. 7669 (2017): 602–606, 10.1038/nature23665. 28847007 PMC5804356

[584] K. Nakagawara , M. Ando , T. Srirat , et al., “NR4A Ablation Improves Mitochondrial Fitness for Long Persistence in Human CAR‐T Cells against Solid Tumors,” Journal for ImmunoTherapy of Cancer 12, no. 8 (2024): 008665. 10.1136/jitc-2023-008665 PMC11331892 39151930

[585] J. Corria‐Osorio , S. J. Carmona , E. Stefanidis , et al., “Orthogonal Cytokine Engineering Enables Novel Synthetic Effector States Escaping Canonical Exhaustion in Tumor‐Rejecting CD8+ T Cells,” Nature Immunology 24, no. 5 (2023): 869, 10.1038/s41590-023-01477-2. 37081150 PMC10154250

[586] A. Hyrenius‐Wittsten , Y. Su , M. Park , et al., “SynNotch CAR Circuits Enhance Solid Tumor Recognition and Promote Persistent Antitumor Activity in Mouse Models,” Science Translational Medicine 13, no. 591 (2021): abd8836. 10.1126/scitranslmed.abd8836 PMC8594452 33910981

[587] A. P. Teixeira and M. Fussenegger , “Synthetic Gene Circuits for Regulation of Next‐Generation Cell‐Based Therapeutics,” Advanced Science 11, no. 8 (2024): 2309088, 10.1002/advs.202309088. 38126677 PMC10885662

[588] H. Sabit , T. M. Pawlik , F. Radwan , et al., “Precision Nanomedicine: Navigating the Tumor Microenvironment for Enhanced Cancer Immunotherapy and Targeted Drug Delivery,” Molecular Cancer 24, no. 1 (2025): 160, 10.1186/s12943-025-02357-z. 40457437 PMC12131435

[589] Q. He , J. Chen , J. Yan , et al., “Tumor Microenvironment Responsive Drug Delivery Systems,” Asian Journal of Pharmaceutical Sciences 15, no. 4 (2020): 416–448. 32952667 10.1016/j.ajps.2019.08.003 PMC7486519

[590] Y. Zhang , J. Wang , G. Qing , et al., “Controlling T Cell–Tumor Cell Interaction with a Biomimetic Physical Barrier for Cancer Immunotherapy,” Proceedings of the National Academy of Sciences 122, no. 28 (2025): 2500589122, 10.1073/pnas.2500589122. PMC12280959 40627401

